# Partially local three-way alignments and the sequence signatures of mitochondrial genome rearrangements

**DOI:** 10.1186/s13015-017-0113-0

**Published:** 2017-08-23

**Authors:** Marwa Al Arab, Matthias Bernt, Christian Höner zu Siederdissen, Kifah Tout, Peter F. Stadler

**Affiliations:** 1 Bioinformatics Group, Department of Computer Science, Interdisciplinary Center for Bioinformatics, Universität Leipzig, Härtelstraße 16–18, 04107 Leipzig, Germany; 2 Swarm Intelligence and Complex Systems, Department of Computer Science, Universität Leipzig, Augustusplatz 10, 04109 Leipzig, Germany; 3 Faculty of Sciences I, Lebanese University, Hadath, Beirut, Lebanon; 4 Helmholtz Centre for Environmental Research-UFZ, Permoserstraße 15, 04318 Leipzig, Germany; 5 Competence Center for Scalable Data Services and Solutions Dresden/Leipzig, German Centre for Integrative Biodiversity Research (iDiv), and Leipzig Research Center for Civilization Diseases, Universität Leipzig, Härtelstraße 16–18, 04107 Leipzig, Germany; 6 Max Planck Institute for Mathematics in the Sciences, Inselstraße 22, 04103 Leipzig, Germany; 7 Fraunhofer Institut for Cell Therapy and Immunology, Perlickstraße 1, 04103 Leipzig, Germany; 8 Department of Theoretical Chemistry, University of Vienna, Währinger Straße 17, 1090 Vienna, Austria; 9 Center for non-coding RNA in Technology and Health, Grønegårdsvej 3, 1870 Frederiksberg C, Denmark; 10 Santa Fe Institute, 1399 Hyde Park Rd., 87501 Santa Fe, USA

**Keywords:** Dynamic programming, Genome rearrangements, Breakpoints, Mitogenomes

## Abstract

**Background:**

Genomic DNA frequently undergoes rearrangement of the gene order that can be localized by comparing the two DNA sequences. In mitochondrial genomes different mechanisms are likely at work, at least some of which involve the duplication of sequence around the location of the apparent breakpoints. We hypothesize that these different mechanisms of genome rearrangement leave distinctive sequence footprints. In order to study such effects it is important to locate the breakpoint positions with precision.

**Results:**

We define a partially local sequence alignment problem that assumes that following a rearrangement of a sequence *F*, two fragments *L*, and *R* are produced that may exactly fit together to match *F*, leave a gap of deleted DNA between *L* and *R*, or overlap with each other. We show that this alignment problem can be solved by dynamic programming in cubic space and time. We apply the new method to evaluate rearrangements of animal mitogenomes and find that a surprisingly large fraction of these events involved local sequence duplications.

**Conclusions:**

The partially local sequence alignment method is an effective way to investigate the mechanism of genomic rearrangement events. While applied here only to mitogenomes there is no reason why the method could not be used to also consider rearrangements in nuclear genomes.

**Electronic supplementary material:**

The online version of this article (doi:10.1186/s13015-017-0113-0) contains supplementary material, which is available to authorized users.

## Background

The small genomes of animal mitochondria, usually harbouring only 13 protein-coding genes as well as their own ribosomal and transfer RNAs, are subject to frequent rearrangements of the gene order. There does not seem to exist a unique molecular mechanism, however. Inversions [1] can be explained by inter-mitochondrial recombination [2, 3]. Similarly, *transposition* [4] and *inverse transposition* [5] may also be the result of nonhomologous recombination events [6, 7]. In a *tandem duplication random loss* (TDRL) event [8, 9], on the other hand, part of the mitogenome, which contains one or more genes, is duplicated in tandem; subsequently, one of the redundant copies of the genes is lost at random. Transpositions can also be explained by a TDRL mechanism, and there is at least evidence that rearrangements involving the inversion of genes can be explained by a duplication-based mechanism, where the duplicate is inverted [10]. It remains an open question how variable the rates and the relative importance of different rearrangement mechanisms are over longer evolutionary time-scales and in different clades. While TDRLs leave a clearly identifiable trace in the mitogenomic sequence, namely the usually rapidly decaying pseudogenized copies of redundant genes [10], little is known about the impact of other rearrangement mechanisms. It has been observed, however, that lineages with frequent rearrangements also show elevated levels of nucleotide sequence variation [11].

**Fig. 1:**
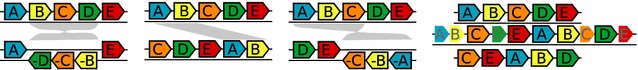
Elementary rearrangement events discussed for mitogenomes. From left to right: inversion, transposition, inverse transposition, tandem duplication random loss. Pseudogenisation leading to eventual gene loss is indicated by *symbols without borders* (Adapted from [12] ©Elsevier)

Mitogenome rearrangements are usually analyzed at the level of signed permutations that represent the order and orientation of the genes, see Fig. 1. Inferred rearrangement scenarios, i.e., sequences of a minimal number of rearrangement operations that explain the differences between two gene orders, of course depend strongly on the operations that are considered [13]. While initially only inversions were considered, more recently algorithms have also been developed for shortest TDRL rearrangement scenarios [14]. In contrast to inversions and transpositions, TDRLs are strongly asymmetric, i.e., when a single TDRL suffices to obtain gene order $\pi '$ from $\pi$, multiple TDRLs are needed to go from $\pi '$ back to $\pi$. Parsimonious TDRL scenarios requiring more than one step are rarely unique [15] and usually a large number of TDRLs with different loss patterns are equivalent in circular genomes [16]. As a consequence, it is impossible to precisely reconstruct a TDRL rearrangement event from gene order information only. It is indispensable to analyze the underlying sequence data to detect duplication remnants. This raises the question whether reversals or transpositions also leave characteristic sequences behind that provide information on the rearrangement type independent of the analysis of gene order data. This would provide more detailed and more reliable data on the relative frequency of different rearrangement mechanisms.

**Fig. 2:**
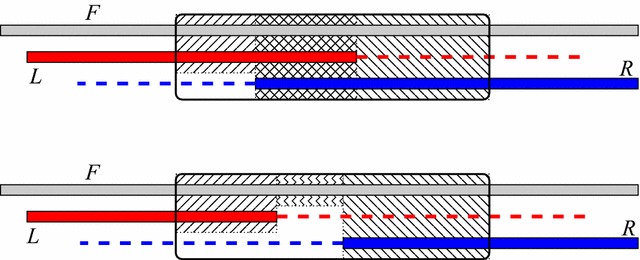
Overview of the problem: we assume that the prefix of *L* and the suffix of *R* is known to be homologous to the reference. It thus suffices to consider a “core region” indicated by the *black frame*. In this region we consider alignments in which *L* and *R* overlap (above) and alignments in which a gap remains between *L* and *R* (below). In the first case, the overlapping, hashed, region is scored as three-way alignment and deletions of a prefix of *R* and a suffix of *L* do not contribute to the score. In the second case, the gap between *L* and *R* is penalized, however. *Dashed lines* indicate the non-aligned parts of the sequences *L* and *R*, resp

It is easy to determine the approximate location breakpoints in relation to annotated genes by the comparison of gene orders. Tools such as CREx [17] also infer the putative type of rearrangement operations by assuming a most parsimonious rearrangement scenario. The exact localization of breakpoints in the genomic sequence is a necessary prerequisite for any detailed investigation into sequence patterns that might be associated with genome rearrangements. Let us now focus on a given breakpoint and suppose, for the moment, that we know which of two mitogenomes represents the ancestral gene order; it will serve as the reference. We denote the sequence of the reference mitogenome in the vicinity of the breakpoint by *F*. The homologous sequence in the derived genome is then split into two non-contiguous parts, which we denote by *L* and *R*. In practice, *F* will be chosen to contain parts of the two genes *a* and *b* that are adjacent to the breakpoint in the reference. *L* and *R* then contain the corresponding parts of *a* and *b* as well flanking sequences extending towards the new neighbors of *a* and *b* in the derived genome. Without loosing generality, we fix the notation so that *R* and *L* are homologous to the right and left part of *F*, respectively. See Fig. 2 for a graphical representation.

If rearrangements always were a simple “cut-and-paste” operation, it would suffice to find the concatenation of a suffix of *R* and a prefix of *L* that best matches *F*. This simple model, however, does not account for TDRLs. We therefore have to allow that *R* and *L* partially overlap. It is also conceivable that recombination-based rearrangements lead to the deletion of a part of the ancestral sequence or the insertion of some unrelated nucleotides. To account for this possibility, we have to allow that a part of *F* in the immediate vicinity of the breakpoint does not appear in either *R* or *L*. The alternative scenarios are outlined in Fig. 2.

Currently, only a few approaches use sequence data for genome rearrangement analysis. Parsimonious rearrangement scenarios w.r.t. cut-and-join operations and indels that minimize the difference in the size distribution of the breakpoint regions from expected values are computed in [18]. For next generation sequencing data differences in the alignments of the reads to a reference genome can be used to uncover rearrangements [19]. Alignments of synteny blocks can be extended into the breakpoint region in order to delineate its position as precisely as possible [20]. This is approach is most closely related to our methods. However, it does not handle duplications and their possible remnants.

The computational problem of pinpointing the breakpoint position and identifying potentially duplicated or deleted sequence fragments can be understood as a specific mixture of local and global alignments of the three sequences *F*, *R*, and *L*, which we will formalize below. Three-way alignment algorithms have a long tradition in bioinformatics [21, 22], where they have been used as components in progressive multiple sequence alignment procedures [23, 24].

The exact solution for multiple sequence alignment has exponential running time in the number of sequences. A such, exact alignments are only possible for a small number of sequences of short length. Therefore, heuristics and approximation methods have been applied in tools such as MAFFT [25], CLUSTAL [26], PASTA [27] and HAlign [28]. Furthermore parallel computing has been introduced to reduce the actual running time for large scale alignments [29]. In our study, however, since we align only the three sequences for the small breakpoint region, using dynamic programming to yield an exact alignment remains feasible. On the other hand, the combination of local and global alignments in multi-way alignments, has not received much attention. In the following section we show that an efficient dynamic programming algorithm can be devised for this task. It is then applied to a set of well-documented mitochondrial genome rearrangements in animals. This focus on mitogenomes is not a fundamental limitation of our method but rather guided by readily available data and extensive literature on well-described individual cases.

## Theory

The alignment of the reference sequence *F* and its two offspring *L* and *R* is global at the “outer end” (here terminal deletions are scored), but local toward the breakpoint region (here terminal deletions in *R* and *L*, resp., remain unscored. Although the problem is symmetric, we follow the usual algorithmic design of dynamic programming algorithms for sequence alignments and consider partial solutions that are restrictions to prefixes of *F*, *L*, and *R*. In the following we denote by $m:=|F|$, $n:=|L|$, and $p:=|R|$ the respective length of the input sequences and by $S_{i,j,k}$ the maximal score of an alignment of the prefixes *F*[1..*i*], *L*[1..*j*], and *R*[1..*k*]. As usual, an index 0 refers to the empty prefix. We restrict our attention to additive scores defined on the input alphabet augmented by the gap character ($\texttt {`-'}$). We write $\gamma (a,b,c)$ for the score of the three-way parts and $\sigma (a,b)$ for the pairwise part, i.e., regions in which a suffix of *L* or a prefix of *R* remains unaligned. More details will be given at the end of this section.

The general step in the recursion for *S* is essentially the same as for global three-way alignments [21, 22], with a single modification introduced by local alignment of the left end of *R*. As in the familiar Smith–Waterman algorithm [30] we have to add the possibility that *R* is still un-aligned to the set of choices. This state is encoded by $k=0$. Thus

1 $$\begin{aligned} S_{i,j,k} = \max {\left\{ \begin{array}{ll} S_{i-1,j-1,k-1} + \gamma (F_i,L_j,R_k) \\ S_{i-1,j-1,k} + \gamma (F_i,L_j,\texttt {'-'}) \\ S_{i-1,j,k-1} + \gamma (F_i,\texttt {'-'},R_k) \\ S_{i-1,j,k} + \gamma (F_i,\texttt {'-'},\texttt {'-'}) \\ S_{i,j-1,k-1} + \gamma (\texttt {'-'},L_j,R_k) \\ S_{i,j-1,k} + \gamma (\texttt {'-'},L_j,\texttt {'-'}) \\ S_{i,j,k-1} + \gamma (\texttt {'-'},\texttt {'-'},R_k) \\ S_{i,j,0} \quad , k > 0 \end{array}\right. } \end{aligned}$$

The initialization at the edges and faces of the three-dimensional “matrix” deserves some separate discussion. Entries of the form $S_{i,0,0}$ correspond to the insertion of a prefix of *F*. Since gaps relative to *R* are not scored but need to be paid for in the alignment with *L*, we have $S_{i,0,0} = i\times g$, where *g* is the uniform gap score. By the same argument $S_{0,j,0}=j\times g$. The attempt to place *R* to the left of *L* and *F* incurs costs relative to both *L* and *F*, thus $S_{0,0,k} = k \times 2g$. The last case makes the alignment local w.r.t. the right end of *R*, i.e., allows penalty-free deletions of any prefix of *R*.

The alignment of *F* and *L* in the absence of *R* is global towards the left, hence the face with $k=0$ is computed according to the Needleman–Wunsch algorithm [31], i.e.,

2 $$\begin{aligned} S_{i,j,0} = \max {\left\{ \begin{array}{ll} S_{i-1,j-1,0} + \sigma (F_i,L_j) \\ S_{i-1,j,0} + \sigma (F_i,\texttt {'-'}) \\ S_{i,j-1,0} + \sigma (\texttt {'-'},L_j) \end{array}\right. } \end{aligned}$$

The $j=0$ plane describes alignments of *F* and *R* before the beginning of *L*. It also follows the Needleman–Wunsch scheme but has a more elaborate scoring considering triples of sequences since the insertions relative to *L* are explicitly penalized here:

3 $$\begin{aligned} S_{i,0,k} = \max {\left\{ \begin{array}{ll} S_{i-1,0,k-1} + \gamma (F_i,\texttt {'-'},R_k) \\ S_{i-1,0,k} + \gamma (F_i,\texttt {'-'},\texttt {'-'}) \\ S_{i,0,k-1} + \gamma (\texttt {'-'},\texttt {'-'},R_k) \end{array}\right. } \end{aligned}$$

The $i=0$ plane, finally, describes the alignment of *L* and *R* without *F*. Since the gaps are penalized at the beginning of *F*, the scoring function $\gamma$ is used in this plane. The cells are filled according to the Needleman–Wunsch recursions in the following manner:

4 $$\begin{aligned} S_{0,j,k}=\max {\left\{ \begin{array}{ll} S_{0,j-1,k-1} + \gamma (\texttt {'-'},L_j,R_k) \\ S_{0,j-1,k} + \gamma (\texttt {'-'},L_j,\texttt {'-'}) \\ S_{0,j,k-1} + \gamma (\texttt {'-'},\texttt {'-'},R_k) \end{array}\right. } \end{aligned}$$

The matrix *S* computes not quite the solution to our problem however. Thus we introduce a two-dimensional scoring matrix *M* that holds the optimal scores of an alignment of *F* and *R* that continues after the end of the aligned part of *L*, i.e., after the breakpoint position in *L*. It satisfies the recursion

5 $$\begin{aligned} M_{i,k} = \max {\left\{ \begin{array}{ll} M_{i-1,k-1} + \sigma (F_i,R_k) \\ M_{i-1,k} + \sigma (F_i,\texttt {'-'}) \\ M_{i,k-1} + \sigma (\texttt {'-'},{R}_k) \\ \mathop {\max \limits _{j}} S_{i,j,k} \\ \mathop {\max \limits _{i'<i}} \mathop {\max \limits _{j}} S_{i',j,0} \end{array}\right. } \end{aligned}$$

The first three cases are the Needleman–Wunsch style extension of the pairwise alignment. The next case corresponds to the situation that *L* ends with position *j* in the three way alignment. In the final case the three sequences do not overlap. Our model stipulates that gaps between the left end of the *FL* alignment and the right end of *FR* alignment are not penalized, hence there is no gap cost contribution for the interval $F[i'+1..i]$ on the reference sequence.

Since the alignment of *F* and *R* is global at its right end, the traceback starts from the lower right corner of *M*, i.e., the entry $M_{mp}$. If there is an overlap region, the traceback moves from *M* to *S* at an index triple (*i*, *j*, *k*) with $k>0$. In the case of a gap region the transition is directly to the *FL* surface, i.e., at $k=0$. The traceback terminates when it reaches $S_{0,0,0}$.

The algorithm uses $O(n^3)$ space and time for input sequences of length *n*. To achieve this time complexity, notice that $\tilde{m}_i := \max _{i'<i}\max _j S_{i',j,0}$ can be pre-computed in quadratic time for each *i* and memoized in a linear array.^1^ As a further optimization, we may use $\tilde{m}_i = \max ( \max _j S_{i-1,j,0}, m_{i-1})$.

The scoring model must satisfy the usual constraints for local similarity based alignments: gap and mismatch scores must be negative, at least on average, as otherwise the option to remove free end gaps would never be taken. Furthermore, mismatch scores must be larger than indel scores to avoid the proliferation of insertions followed by deletions or vice versa. Here we use the sum of pairs model

6 $$\begin{aligned} \gamma (a,b,c)=\left( \sigma (a,b)+\sigma (a,c)+\sigma (b,c)\right) /w(a,b,c) \end{aligned}$$

with pairwise similarities $\sigma (\,.\,,\,.\,)$ defined on the alphabet comprising the four nucleotides and the gap character $\texttt {`-'}$. This scoring model satisfies the required constraints, provided the pairwise scores $\sigma (\,.\,,\,.\,)$ are suitable for pairwise local sequence alignments [33]. We use match and mismatch scores $\sigma (a,a)=\alpha$ and $\sigma (a,b)=\beta$ for $a\ne b\in \mathcal {A}$ that are independent of the letter of the alphabet. The gap scores are also specified in a sequence-independent manner: $\sigma (a,\texttt {`-'})=\sigma (\texttt {`-'},a)=\delta$ and $\sigma (\texttt {`-'},\texttt {`-'})=0$. The pairwise scoring scheme is thus specified by $\{\alpha ,\beta ,\gamma \}$. In addition we use a “sum-of-pair weight” $w(a,b,c)=W$ if $a,b,c\in \mathcal {A}$ are all letters, and $w(a,b,c)=1$ if at least one of *a*, *b*, and *c* is a gap character. This extra parameter modifies the relative weight of the overlap region compared to the parts of the alignments in which only *L* or only *R* is aligned to the reference *F*.

## Methods

### Parametrization of the scoring function

In order to test whether the dynamic programming scheme outlined in the previous section is indeed capable of determining the breakpoint location with sufficient accuracy we used synthetic data. We separately prepared test data with a gap relative to *F* and test data with an overlap of *R* and *L*. A random suffix or prefix of the same length was appended to the *R* and *L* sequences, thereby complementing them to mimic a scenario in which input sequences comprise two annotation items flanking the approximate location of the breakpoint. Both the designed gap and overlap lengths were fixed at 10 nucleotides. We used both perfect sequences and sequences in which *L* and *R* were mutated with a position-wise probability of 15 or 30%, respectively.

**Fig. 3:**
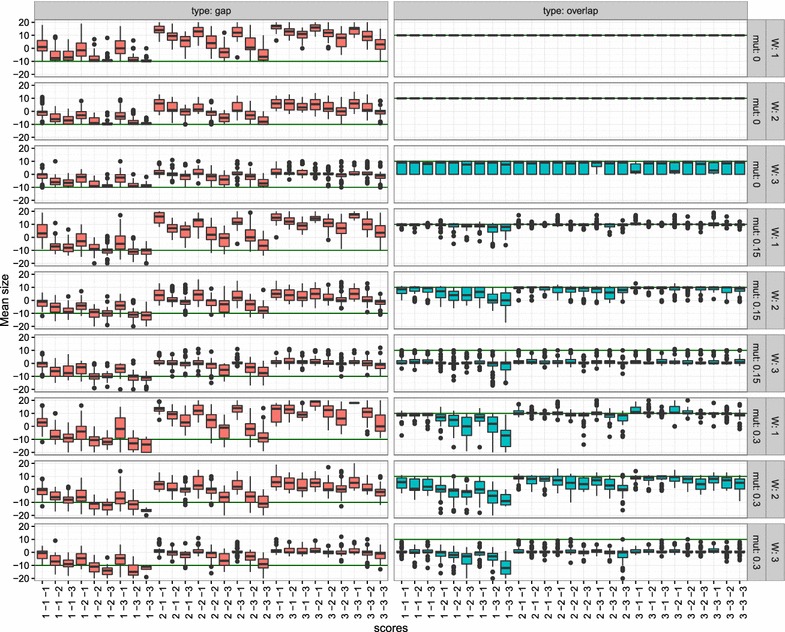
Variation of overlap and gap sizes in simulated data as a function of alignment parameters (score: match, mismatch, gap), sum of pairs weight *W*, and sequence divergence mut between *F* and *R* or *L*, respectively. The ground truth is indicated by solid lines at +10 of for the gap scenario and $+10$ for the overlap case

In order to survey the influence of different alignment parameters we scanned all combinations of match scores in $\{1,2,3\}$ and gap/mismatch scores in $\{-1,-2,-3\}$. The overlap is defined as the number of alignment columns from the first to the last position at which all three sequences, *F*, *L*, and *R*, are aligned (Fig. 3). The size of the gap between or overlap of *R* and *L* was estimated from a sample of 50 simulated instances for each parameter combination. In addition we investigate the effect of the sum of pairs weight parameter $W\in \{1,2,3\}$

We first consider the test cases with overlap. In the absence of artificial mutation the expected overlap size matches the expected value for $W=1$. For large values of *W* the observed overlap is reduced to $5.9\pm 4.4$. This is not unexpected since large values of *W* down-weight the triple matches in the overlap region relative to indels in one of the sequences. The overlap size decreases with increasing sequence variation. Again, larger values of *W* aggravate the effect. For $W=1$ most pairwise scoring models yield good estimates of the true overlap size. Exceptions are scoring schemes [given as triple (match, mismatch, gap)] with small relative match scores such as $(1, -2, -2)$, $(1, -2, -3)$, $(1, -3, -2)$, $(1, -3, -3)$, which produce overlaps that are too short or even gaps in the presence of elevated levels of sequence variation. If match scores are too large, overlaps tend to be overestimated. This is the case for the scoring schemes $(3, -1, -1)$, $(3, -2, -1)$, and $(3, -3, -1)$.

In the test cases with a gap between *L* and *R* we find that gap sizes do not depend strongly on the sequence divergence. In general, gap sizes tend to be underestimated, in particular in scoring functions with small gap penalties. This is explained by the fact that there is a non-negligible chance that the first few nucleotides of the random suffix of *R* or the last few nucleotides of the random prefix of *L* also match with *F*. Best results were obtained for $(1, -1, -3)$, $(1, -1, -2)$ for $W=1$. Larger values of *W* do not lead to improvements.

Combining the results for the gapped and the overlapping examples, the scoring functions $(1, -1, -3)$, $(1, -1, -2)$, and possibly $(2, -3, -3)$ perform best. Throughout the remainder of this contribution we use $W=1$ and the pairwise alignment scores $(1,-1,-2)$.

### Mitochondrial rearrangement data

We apply our alignment method to a collection of 152 unique animal mitochondrial gene order pairs that contain exactly the 37 canonical genes: atp6, atp8, cob, cox1, cox2, cox3, nad1, nad2, nad3, nad4, nad4l, nad5, nad6, rrnL, rrnS, trnA, trnC, trnD, trnE, trnF, trnG, trnH, trnI, trnK, trnL1, trnL2, trnM, trnN, trnP, trnQ, trnR, trnS1, trnS2, trnT, trnV, trnW, trnY. The list of accession numbers was taken from [34] and the gene orders extracted from the annotations in RefSeq release 73 [35]. Rearrangement analysis for all pairs of unique gene orders has been done with CREx [17]. For our analysis only pairs of unique gene orders are considered that are separated by a single rearrangement event, i.e., we retain 144 genome pairs of which 124 are predicted by CREx [17] as transpositions, 14 as inversions and six as TDRL. Since transposition and inversion are symmetric both directions are considered, i.e. 62 and 7 pairs in each direction, respectively. For the analysis of the breakpoint regions for each pair of unique gene orders a pair of representative sequences has been chosen that is most closely related according to the NCBI taxonomy database [36].

For each predicted breakpoint we extract the reference sequence from one genome and the two predicted fragments from the other, where the former genome exhibits the putative ancestral state of the rearrangement and the latter the putative derived state. All sequences are retrieved from RefSeq release 73 [35]. The reference sequence *F* is the concatenation of the last 60 nt of the left gene, the intergenic region if available, and the first 60 nt of the right gene. The left query *L* is formed of the last 60 nt of the left query gene and the intergenic region with respect to the next gene (previous gene in case of inversion). The right query *R* is formed by the intergenic region with respect to the previous gene (next gene in case of inversion). In case of inversion the reverse complement of the corresponding query is used in the alignment. For each triple (*F*, *L*, *R*) the alignment is computed as described above and the length of the overlap of *L* and *R* or the length of the gap between *L* and *R* is recorded. However, we exclude 191 alignments that contain stretches of intergenic region longer than 40 nt in the reference sequence and *R* or *L* are very short i.e less than 10 nt in *F* from the statistical analysis. In total, we removed 233 of 459 alignments. Most of these cases are due to annotation errors in RefSeq or because we did not consider the control region for the computation of the gene orders. A complete set of the corresponding breakpoint alignments is compiled in Additional files 2 and 3 in human and machine readable form, respectively. Furthermore, the rearrangement events considered here are compiled in Additional file 1.

**Fig. 4:**
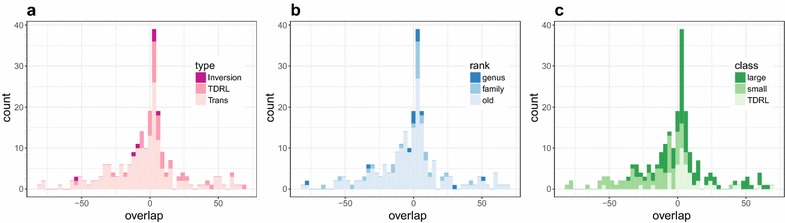
Distribution of overlap sizes for the breakpoints types of pairs of animal species (**a**) as classified by CREx. **b** Species pairs are grouped by the phylogenetic age of their last common ancestor according to the NCBI taxonomy. **c** Grouping w.r.t. to the structure of the overlap: larger and smaller overlap chosen as reference *F* for symmetric rearrangments, resp., and asymmetric rearrangements, i.e., TDRLs

We only included the 49 pairs of gene orders with symmetric rearrangements in the comparison of the average overlap sizes where the alignments for both choices of the reference were retained. This data set comprises three inversions and 46 transpositions, corresponding to 186 alignments.

## Results and discussion

Figure 4 summarizes the distribution of overlap sizes. Several patterns are clearly visible. First, there is a substantial fraction of alignments with long gaps. The main cause of these long gaps is a very low nucleic acid sequence similarity with an average of only 39% between the gene portions (60 nt from start or end). This explains 10 of the 15 cases. Some of the remaining cases are explained by annotation errors such as the misannotation of *trnY* in *Luvarus imperialis*. In four alignments the long gaps are caused by the long intergenic region in reference sequence *F*.

The distribution of the overlap sizes for the different rearrangement types is shown in Fig. 4a. For TDRLs we expect to see overlaps of *R* and *L* that are interpreted as remnants of the duplication event. Indeed, of the 44 alignments of breakpoint regions that are due to TDRL in the CREx data, 37 have a non-zero overlap. The mean overlap length is 7.5 nt, also including instances with a gap. In principle, the overlap can vanish due to a complete deletion of the duplicate and due to sequence divergence between the functional and the non-functional copy that eventually erases all detectable sequence similarity. The latter explanation is plausible only for species pairs with large phylogenetic distance.

**Fig. 5:**
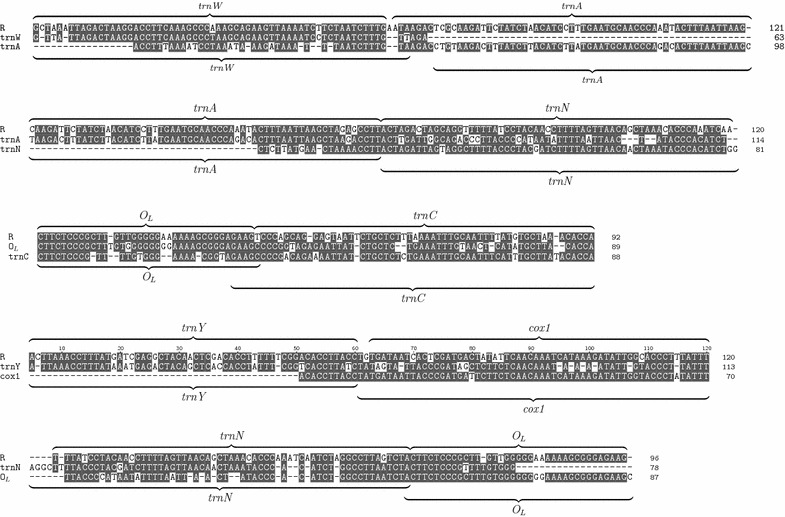
Breakpoint alignments for the mitochondrial genome rearrangement separating the reference gene order of *Phaeognathus hubrichti* (NC_006344) from *Hydromantes brunus* (NC_006345). This is a clear example of a TDRL. Alignments are displayed with TeXshade [37]

There is no *a priori* theoretical prediction for the expected overlap in the case of inversions and transpositions. Among the seven alignments classified as inversions by CREx there is no or only a marginal overlap of a few nucleotides. Note that overlaps of 2–3 nt can be easily explained by random matches rather than as a remnant of the genome rearrangement. Thus, our data indicates that the molecular mechanism generating inversions neither duplicates not deletes sequence in the breakpoint region. Also, there seems to be no common sequence pattern associated with these breakpoints.

**Table 1:** The rearranged pairs with average overlap size $\bar{\ell }>10$ nt and their rearrangement type as specified in the literature

Species	Accessions	$\bar{\ell }$	Literature Type	References
*Maulisia mauli* Normichthys operosus	*NC_011007*NC_011009	127	TDRL	[10]
*Phaeognathus hubrichti* Hydromantes brunus	NC_006344NC_006345	66	no duplication TDRL	[38, 39]
*Galaxias maculatus* Galaxiella nigrostriata	NC_004594NC_008448	62	duplication deletion	[10]
*Jordanella floridae* Xenotoca eiseni	NC_011387NC_011381	59	duplication deletion	[40]
*Olisthops cyanomelas* Chlorurus sordidus	NC_009061NC_006355	27	duplication	[41]
*Parachanna insignis* Odontobutis platycephala	NC_022480NC_010199	25	TDRL	[42]
*Platax orbicularis* Luvarus imperialis	NC_013136NC_009851	20	annotation error trnY	[43]
*Ischikauia steenackeri* Chanodichthys mongolicus	NC_008667NC_008683	20	annotation error trnI	[44]
*Albula glossodonta* Pterothrissus gissu	NC_005800NC_005796	20	TDRL	[45]
*Galago senegalensis* Otolemur crassicaudatus	NC_012761NC_012762	19	annotation error trnI	[46]
*Batrachoseps wrighti* Batrachoseps attenuatus	NC_006333NC_006340	19	duplication deletion	[39]
*Diplophos taenia* Chauliodus sloani	NC_002647NC_003159	19	TDRL	[47]
*Chlorurus sordidus* Xenotoca eiseni	NC_006355NC_011381	18	duplication deletion	[41]
*Chauliodus sloani* Myctophum affine	NC_003159NC_003163	10	duplication deletion	[48]

Transpositions can be generated by TDRL or a recombination. The 177 alignments of the breakpoint regions of gene orders separated by a transposition show an average overlap of 5.2 nt. Only for less than half of the alignments (83) an overlap $\ge$0 has been found. This can be explained in part by annotation errors. The main reason, however, is that both possible choices for the ancestral state, i.e., the reference sequence *F* are included for each pair. Only one of them is expected to generate an overlap (see below). Thus many of the cases that CREx predicts as transposition are really generated by a TDRL on a molecular level. By design, CREx prefers to predict transpositions over TDRLs as the more parsimonious alternative if both are possible explanations. To be precise, CREx prefers to predict transpositions over TDRLs as the more parsimonious alternative for rearrangements detected in linear nodes of the strong interval tree [49] of the two input gene orders [17]. Table 1 summarizes example cases from the literature where we detected large overlaps. All these rearrangements are explained by duplication mechanism or TDRL in the literature. However three cases are not originally rearranged, but errors in the RefSeq annotation lead to spurious rearrangements.

**Fig. 6:**
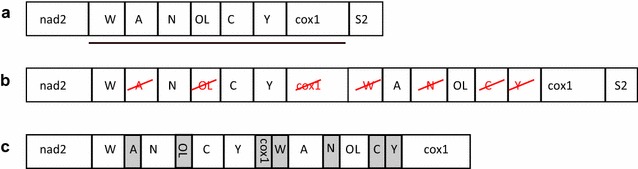
Rearrangement of *Hydromantes brunus*. **a** Ancestral gene order, the underlined genes are duplicated. **b** Duplication and deletion. **c** Current gene order with gene remnants in *gray boxes*

We consider *Hydromantes brunus* in some detail. In [38], the rearrangement in *H. brunus* is claimed to be without duplication based on an analysis of nucleotide skew to classify between rearrangement or partial genome duplication. The same rearrangement has been claimed to be a TDRL in [39]. The alignment of the corresponding breakpoints in Fig. 5 shows an average overlap of 74 nt which is a strong evidence of duplication mechanism. Thus we support the TDRL model [39] with regard to the molecular mechanism. Nevertheless our alignments show a different inferred intermediate state, as shown in Fig. 6. In [39] the duplicated genes are: part of nad2, trnW, trnA, trnN, O$_L$, trnC and trnY. We, however, suggest the duplication of trnW, trnA, trnN, O$_L$, trnC, trnY and cox1. The absence of the remnant of nad2 and the presence of the cox1 remnant support our model.

Although none of the inversions show large overlaps, there is no clear association between the size of the overlap or gap with TDRL or transposition according to the CREx classification. In fact, at least a substantial part of the transpositions is clearly the result of a rearrangement mechanism that generates sizable duplications of mitogenomic sequence.

To our surprise overlaps are also found for older rearrangements, see the Fig. 4b. From the largest overlaps shown in Table 1 at least three pairs are on order level (*J. floridae*-*X. eiseni*, *O. cyanomelas*-*C. sordidus*, *D. taenia*-*C. sloani*). Thus, deletion is not always immediate and duplication remnants can be detected sometimes also for ancient rearrangements.

Figure 4c summarizes the overlap sizes grouped into three classes. The first two correspond to breakpoints of symmetric rearrangements. Here we distinguish for each breakpoint the choice of the reference *F* that yields the larger or the smaller average overlap, respectively: 81% of the overlaps and 70% of the gaps fall into large and small class, respectively. The third class are the asymmetric rearrangements, i.e., TDRLs, which allow only one choice of *F*.

**Fig. 7:**
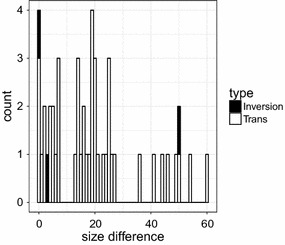
Difference in average overlap size between symmetric rearrangements in the two directions in absolute value

The difference in overlap size caused by the choice of the reference *F* is shown in Fig. 7 for the symmetric rearrangements. In 67% of the 49 cases the difference is larger than 10 nucleotides (note that the one large difference for an inversion is due to annotation errors). This relatively large difference is due to an overlap caused by duplication in one genome, which turns out to be a gap when this species is used as a reference. The comparison of the alignments for both choices of *F* thus makes it possible to deduce the direction of the symmetric rearrangements, i.e., the ancestral and the derived gene order. It also shows the importance of the choice of the correct reference sequence to allow to discriminate between rearrangements caused by a duplication mechanism and those that result from other mechanisms such as inversion and translocation. However, in phylogenetically old rearrangement events the sequences of duplicated genes may be totally degenerated; in these cases, it becomes difficult to specify the mechanism behind the rearrangement or its direction.

## Concluding remarks

We have introduced a specialized three-way alignment model to study the breakpoint regions of mitochondrial genome rearrangements in detail. We observed several unexpected features. In particular, a substantial fraction of rearrangements involves duplication of genomic DNA, many of which have not been recognized as TDRL-like events. This in particular pertains to many events that have been classified as transpositions. On the other hand, some apparent TDRL events do not produce overlaps. While it is possible that in the case of ancient events the genomic sequences have diverged beyond the point where duplicated DNA is still recognizable, it is also possible that some of the ostensible TDRLs in fact correspond to possibly multiple rearrangements of other types. Clearly, further investigations into the individual cases will be necessary to resolve this issue completely. The present study at the very least adds to the evidence that multiple rearrangement mechanisms are at work and indicates that their classification is by no means a trivial task.

Reports of mitogenomic rearrangement are usually not sufficiently precise. In most publications the (potential) molecular mechanisms are not analyzed at all. The term “translocation” is often used to express only that genes have been moved rather than to designate a specific type of rearrangement operation or a molecular mechanism. The term TDRL is used more or less interchangeably for the resulting rearrangement operation and the molecular mechanism generating it; the published analysis usually is limited to the rearrangement operation. For the other rearrangement operations that are assumed in mitochondrial genomes, i.e., inversions, transpositions, and inverse transpositions, the mechanisms still await elucidation in most cases. In this paper we have presented a method that can help to uncover the molecular mechanisms of genome rearrangement. In conjunction with the available methods for genome rearrangement analysis [13] and the consideration of tRNA remolding [50], which can mimic rearrangements, the analyses of breakpoint sequences will significantly improve our understanding of mitochondrial gene order evolution.

The alignment method introduced here is not limited to mitochondrial genomes. Since it is meaningfully applied only to a sequence interval of moderate size around a genomic breakpoint, it could also be applied to study the evolution of nuclear genomes and to investigate chromosomal rearrangements in a cancer research context.

The partially local alignment problem introduced here also poses theoretical questions, which we hope to answer in forthcoming research. Most obviously, it suggests to consider multiple alignments in which, for each input sequence and both of its ends, either a local or global alignment is requested. Given such a problem specification, how can one construct a corresponding dynamic programming algorithm, possibly with the additional constraint that the dynamic programming scheme also supports a probabilistic version. Beyond this question one may of course also ask whether there are interesting generalization of overlap alignments and combinations of local, global, and overlap alignments.

## Additional files

**Additional file 1.** Mitogenome rearrangements in animals. List of species and accession numbers that were chosen as representative of the gene order rearrangements detected by CREx.

**Additional file 2.** Breakpoint alignments. Human readable, annotated breakpoint alignments for all gene order rearrangements in animal mitochondria. PDF file.

**Additional file 3.** Breakpoint alignments. Machine readable breakpoint alignments for all gene order rearrangements in animal mitochondria. Stockholm alignment format.

## Footnotes

1 Memoization [32] is a technique from computer science to increase the efficiency of programs by storing intermediate results that are needed multiple times instead of recomputing them. Here this is also achieved with the *M* and *S* arrays.

## References

[1] Smith MJ, Bamfield DK, Doteval K, Gorski S, Kowbel DJ (1989). Gene arrangement in sea star mitochondrial DNA demonstrates a major inversion event during echinoderm evolution. Gene.

[2] Dowton M, Campbell NJH (2001). Intramitochondrial recombination-is it why some mitochondrial genes sleep around?. Trends Ecol Evol.

[3] Bacman SR, Williams SL, Moraes CT (2009). Intra- and inter-molecular recombination of mitochondrial DNA after invivo induction of multiple double-strand breaks. Nucleic Acids Res.

[4] Boore JL, Collins TM, Stanton D, Daehler LL, Brown WM (1995). Deducing the pattern of arthropod phylogeny from mitochondrial DNA rearrangements. Nature.

[5] Boore JL, Lavrov DV, Brown WM (1998). Gene translocation links insects and crustaceans. Nature.

[6] Lunt DH, Hyman BC (1997). Animal mitochondrial DNA recombination. Nature.

[7] Groth C, Petersen RF, Piškur J (2000). Diversity in organization and the origin of gene orders in the mitochondrial DNA molecules of the genus Saccharomyces. Mol Biol Evol.

[8] Moritz C, Brown WM (1986). Tandem duplications of D-loop and ribosomal RNA sequences in lizards mitochondrial DNA. Science.

[9] Boore JL, Sankoff D, Nadeau JH (2000). Comparative genomics: empirical and analytical approaches to gene order dynamics, map alignment and the evolution of gene families, vol. 1., Computational biology series. The duplication/random loss model for gene rearrangement exemplified by mitochondrial genomes of deuterostome animals.

[10] Jühling F, Pütz J, Bernt M, Donath A, Middendorf M, Florentz C, Stadler PF (2012). Improved systematic tRNA gene annotation allows new insights into the evolution of mitochondrial tRNA structures and into the mechanisms of mitochondrial genome rearrangements. Nucleic Acids Res..

[11] Xu W, Jameson D, Tang B, Higgs PG (2006). The relationship between the rate of molecular evolution and the rate of genome rearrangement in animal mitochondrial genomes. J Mol Evol.

[12] Bernt M, Braband A, Schierwater B, Stadler PF (2013). Genetic aspects of mitochondrial genome evolution. Mol Phylog Evol.

[13] Fertin G, Labarre A, Rusu I, Tannier E, Vialette S (2009). Combinatorics of genome rearrangements.

[14] Chaudhuri K, Chen K, Mihaescu R, Rao S. On the tandem duplication-random loss model of genome rearrangement. In: Proceedings of the seventeenth annual ACM-SIAM symposium on discrete algorithm. Philadelphia: Society for Industrial and Applied Mathematics; 2006. p. 564–70.

[15] Bernt M, Chen K-Y, Chen M-C, Chu A-C, Merkle D, Wang H-L, Chao K-M, Middendorf M (2011). Finding all sorting tandem duplication random loss operations. J Discret Algorithms.

[16] Hartmann T, Chu AC, Middendorf M, Bernt M. Combinatorics of tandem duplication random loss mutations on circular genomes. In: IEEE/ACM Transactions on Computational Biology and Bioinformatics. 2016. 10.1109/TCBB.2016.2613522 28114075

[17] Bernt M, Merkle D, Rasch K, Fritzsch G, Perseke M, Bernhard D, Schlegel M, Stadler PF, Middendorf M (2007). CREx: inferring genomic rearrangements based on common intervals. Bioinformatics.

[18] Bulteau L, Fertin G, Tannier E (2016). Genome rearrangements with indels in intergenes restrict the scenario space. BMC Bioinform.

[19] Lemaitre C, Tannier E, Gautier C, Sagot M-F (2008). Precise detection of rearrangement breakpoints in mammalian chromosomes. BMC Bioinform.

[20] Medvedev P, Stanciu M, Brudno M (2009). Computational methods for discovering structural variation with next-generation sequencing. Nat Methods.

[21] Gotoh O (1986). Alignment of three biological sequences with an efficient traceback procedure. J Theor Biol.

[22] Konagurthu AS, Whisstock J, Stuckey PJ (2004). Progressive multiple alignment using sequence triplet optimization and three-residue exchange costs. J. Bioinf. Comp. Biol..

[23] Hirosawa M, Hoshida M, Ishikawa M, Toya T (1993). MASCOT: multiple alignment system for protein sequences based on three-way dynamic programming. Comput Appl Biosci.

[24] Kruspe M, Stadler PF (2007). Progressive multiple sequence alignments from triplets. BMC Bioinform.

[25] Katoh K, Standley DM (2013). MAFFT multiple sequence alignment software version 7: improvements in performance and usability. Mol Biol Evol.

[26] Larkin MA, Blackshields G, Brown N, Chenna R, McGettigan PA, McWilliam H, Valentin F, Wallace IM, Wilm A, Lopez R (2007). Clustal W and Clustal X version 2.0. Bioinformatics.

[27] Mirarab S, Nguyen N, Guo S, Wang L-S, Kim J, Warnow T (2015). Pasta: ultra-large multiple sequence alignment for nucleotide and amino-acid sequences. J Comput Biol.

[28] Zou Q, Hu Q, Guo M, Wang G (2015). HAlign: fast multiple similar DNA/RNA sequence alignment based on the centre star strategy. Bioinformatics.

[29] Chen X, Wang C, Tang S, Yu C, Zou Q (2017). CMSA: a heterogeneous CPU/GPU computing system for multiple similar RNA/DNA sequence alignment. BMC Bioinform.

[30] Smith TF, Waterman MS (1981). Identification of common molecular subsequences. J Mol Biol.

[31] Needleman SB, Wunsch CD (1970). A general method applicable to the search for similarities in the aminoacid sequences of two proteins. J Mol Biol.

[32] Michie D (1968). Memo functions and machine learning. Nature.

[33] Nguyen K, Guo X, Yi P (2016). Multiple biological sequence alignment: scoring functions. Algorithms and applications.

[34] Bernt M. Gene order rearrangement methods for the reconstruction of phylogeny. PhD thesis, Fakultt für Mathematik und Informatik der Universitt Leipzig. 2010.

[35] Pruitt KD, Tatusova T, Maglott DR (2005). NCBI reference sequence (RefSeq): a curated non-redundant sequence database of genomes, transcripts and proteins. Nucleic Acids Res.

[36] Benson DA, Karsch-Mizrachi I, Lipman DJ, Ostell J, Sayers EW (2008). GenBank. Nucleic Acids Res.

[37] Beitz E (2000). TeXshade: shading and labeling of multiple sequence alignments using LaTeX2e. Bioinformatics.

[38] Fonseca MM, Froufe E, Harris DJ (2006). Mitochondrial gene rearrangements and partial genome duplications detected by multigene asymmetric compositional bias analysis. J Mol Evol.

[39] Müller RL, Boore JL (2005). Molecular mechanisms of extensive mitochondrial gene rearrangement in plethodontid salamanders. Mol Biol Evol.

[40] Tatarenkov A, Mesak F, Avise JC (2017). Complete mitochondrial genome of a self-fertilizing fish *Kryptolebias marmoratus* (Cyprinodontiformes, Rivulidae) from Florida. Mitochondrial DNA A.

[41] Mabuchi K, Miya M, Satoh TP, Westneat MW, Nishida M (2004). Gene rearrangements and evolution of tRNA pseudogenes in the mitochondrial genome of the parrotfish (teleostei: Perciformes: Scaridae). J Mol Evol.

[42] Ki J-S, Jung S-O, Hwang D-S, Lee Y-M, Lee J-S (2008). Unusual mitochondrial genome structure of the freshwater goby odontobutis platycephala: rearrangement of trnas and an additional non-coding region. J Fish Biol.

[43] Yamanoue Y, Miya M, Matsuura K, Yagishita N, Mabuchi K, Sakai H, Katoh M, Nishida M (2007). Phylogenetic position of tetraodontiform fishes within the higher teleosts: Bayesian inferences based on 44 whole mitochondrial genome sequences. Mol Phylogenet Evol.

[44] Saitoh K, Sado T, Mayden R, Hanzawa N, Nakamura K, Nishida M, Miya M (2006). Mitogenomic evolution and interrelationships of the cypriniformes (actinopterygii: Ostariophysi): the first evidence toward resolution of higher-level relationships of the worlds largest freshwater fish clade based on 59 whole mitogenome sequences. J Mol Evol.

[45] Inoue JG, Miya M, Tsukamoto K, Nishida M (2001). Complete mitochondrial DNA sequence of conger myriaster (teleostei: Anguilliformes): novel gene order for vertebrate mitochondrial genomes and the phylogenetic implications for anguilliform families. J Mol Evol.

[46] Matsui A, Rakotondraparany F, Munechika I, Hasegawa M, Horai S (2009). Molecular phylogeny and evolution of prosimians based on complete sequences of mitochondrial DNAs. Gene.

[47] Satoh TP, Miya M, Mabuchi K, Nishida M (2016). Structure and variation of the mitochondrial genome of fishes. BMC Genom.

[48] Poulsen JY, Byrkjedal I, Willassen E, Rees D, Takeshima H, Satoh TP, Shinohara G, Nishida M, Miya M (2013). Mitogenomic sequences and evidence from unique gene rearrangements corroborate evolutionary relationships of myctophiformes (neoteleostei). BMC Evol Biol.

[49] Heber S, Stoye J. Finding all common intervals of $$k$$ permutations. In: Amihood A, Landau GM, editors. Combinatorial pattern matching. Lect Notes Comp Sci. vol. 2089. Berlin : Springer; 2001. p. 207–18.

[50] Sahyoun AH, Hölzer M, Jühling F, Höner zu Siederdissen C, Al-Arab M, Tout K, Marz M, Middendorf M, Stadler PF, Bernt M (2015). Towards a comprehensive picture of alloacceptor tRNA remolding in metazoan mitochondrial genomes. Nucleic Acids Res.

