# Supplementary material for: Partially local three-way alignments and the sequence signatures of mitochondrial genome rearrangements
Source: Algorithms Mol Biol. 2017 Aug 23;12:22. doi: 10.1186/s13015-017-0113-0 (PMC5569537; doi:10.1186/s13015-017-0113-0)
Supplement: Supplementary file 2 — Additional file 2. Breakpoint alignments. Human readable, annotated breakpoint alignments for all gene order rearrangements in animal mitochondria. PDF file. [file 13015_2017_113_MOESM2_ESM.pdf]

1 Trans

1.1 NC_011007-NC_011009

Avg ovsize: 127

LCA: Platytroctidae-family

1.1.1 trnS2-trnD 131

```
                                   trnS2                                                         trnD
R      GGAGTTGAGCCCCCATATGCTGGTTTCAAGCCAGCCGCATAACCGCTCTGCCACTTTCTTCTATCAAGAACCCTAGTAAAATAGCACATTACACTGCCTTGTCGAGACAGAGTTGTGGGTTAGAGCCC   128
trnS2  GGAGTTGAGCCCCCATATGCTGGTTTCAAGCCAGCCGCATAACCGCTCTGCCACTTTCTTCTATCA-GAACCCTAGTTAAA-ATCACATTACACTGCCTTATCGAGACAGATTTGTGGGTTAAAACCC   126
trnD   -GAGTTGAGCCCCCATATGCTGGTTTCAAGTCAGCCGCATAGCCGCTCTGCCACTTTCTTCTATCA-GAACCCTAGTAAAA-ATCACATTACACTGCCTTGTCGAGACAGATTTGTGGGTTAAAACCC   125
                                   trnS2
                                                                                                trnD

       trnD
R      CGCG--   132
trnS2  CGCG--   130
trnD   CGCGGG   131
       trnD
```

1.1.2 trnD-cox2 131

```
       [trnD]                                                                        [cox2]
R      AATAGCACATTACACTGCCTTGTCGAGACAGAGTTGTGGGTTAGAGCCCCGCGGGTCCTGAGCTTTAAGCTAGAATGGCACATCCCTCACAATTAGGATTCCAAGACGCGGCCTCACCAGTAATAGAA  128
trnD   AAAATCACATTACACTGCCTTGTCGAGACAGATTTGTGGGTTAAAACCCCGCGGGTCCTGAGCTTTAAGCTA-A-TGGCACATCCCTCACAATTAGGGTCCCAAAACGCGGCCTCACCAGTAATAGAA  126
cox2   -AAATCACATTACACTGCCTTATCGAGACAGATTTGTGGGTTAAAACCCCGCGGGTCCTGAGCTTTAAGCTAGAATGGCACATCCCTCACAATTAGGATTCCAAGACGCGGCCTCACCAGTAATAGAA  127
       [trnD]                                                                        [cox2]
```

```
       [cox2]
R      GAGC-T  133
trnD   GAGC--  130
cox2   GAGCTT  133
       [cox2]
```

1.1.3 cox1-trnS2 121

```
       [cox1]                                                                   [trnS2]
R      GATGCCCTCCCCC-CTACCACACATTCGAAGAGCCAGCTTTCGTCCAAGTACAATCAAACTGACGAGAAAGGGAGGAGTTGAGCCCCCATATGCTGGTTTCAAGCCAGCCGCATAACCGCTC-  121
cox1   GATGCCCTCCCCC-CTACCACACATTCGAGGAGCCAGCTTTTGTCCAAGTTCAATCAAACTGACGAGAAAGGGAGGAGTTGAGCCCCCATATGCTGGTTTCAAGTCAGCCGCATAGCCGCTC-  121
trnS2  -ATGCCCTCCCCCTCTACCACAC--TCGAAGAGCCAGCTTTTGTCCAAGTTCAATCAAGCTGACGAGAAAGGGAGGAGTTGAGCCCCCATATGCTGGTTTCAAGCCAGCCGCATAACCGCTCT  120
       [cox1]                                                                   [trnS2]
```

1.2 NC_006344-NC_006345

Avg ovsize: 74

LCA: Plethodontinae-subfamily

1.2.1 OL-trnC 95

OL *trnC*

```
R     CTTCTCCCGCTT-GTTGGGGGAAAAAAGCGGGAGAAGTCCCAGCAG-GAGTAATTCTGCTCTTTAAAATTTGCAATTTTATGTGCTAA-ACACCA   92
OL    CTTCTCCCGCTTTGTGGGGGGGGAAAAGCGGGAGAAGCCCCGGTAGAGAATTAT-CTGCTC--TGAAATTTGTAACT-CATATGCTTA--CACCA   89
trnC  CTTCTCCCG-TT--TTGTGGG--AAAA-CGGTAGAAGCCCCGACAGAAAATTAT-CTGCTCTCTGAAATTTGCAATTTCATTTGCTTATACACCA   88
```

OL

trnC

1.2.2 trnA-trnN 80

trnA *trnN*

```
R     CAAGATTCTATCTAACATCCTTTGAATGCAACCCAAATACTTTAATTAAGCTAGAGCCTTACTAGACTAGCAGGTTTTTATCCTACAACCTTTTAGTTAACAGCTAAACACCCAAATCAA-   120
trnA  TAAGACTTTATCTTACATCTTATGAATGCAACCCAGACACTTTAATTAAGCTAAGACCTTACTTGATTGGCAGACCCTTACCCCATAATATTTTAATTAAC---T--ATACCCACATCT--   114
trnN  ---------------------------------------CTCTTATGAA-CTAAAACCTTACTAGATTAGTAGGCTTTTACCCTACGATCTTTTAGTTAACAACTAAATACCCACATCTGG    81
```

trnA

trnN

1.2.3 trnN-OL 77

trnN *OL*

```
R     ----T-TTATCCTACAACCTTTTAGTTAACAGCTAAACACCCAAATCAATCTAGGCCTTAGTCTACTTCTCCCGCTT-GTTGGGGGAAAAAAGCGGGAGAAG-   96
trnN  AGGCTTTTACCCTACGATCTTTTAGTTAACAACTAAATACCC-A--C-ATCT-GGCCTTAATCTACTTCTCCCGTTTTGTGGG-------------------   78
OL    ------TTACCCCATAATATTTTAATT-A-A-CT--ATACCC-A--C-ATCT-GGCCTTAATCTACTTCTCCCGCTTTGTGGGGGGGGAAAAGCGGGAGAAGC   87
```

trnN

OL

1.2.4 trnY-cox1 70

trnY *cox1*

```
R     AA--GGACTTAAACCTTTATGATCGAGGCTACAACTCGACACCTTTTTCGGACACCTTACCTGTGATAATCACTCGATGACTATATTCAACAAATCATAAAGATATTGGCACCCTTTATTT-   120
trnY  AAAAGGA-TTAAACCTTTATAAATGAGACTACAGCTCACCACCTATTT-CGGTCACCTTATCTATAGTA-TTACCCGATAGCTCTTCTCAACAAAT-A-A-A-ATATT-GTACCCT-TATTT-   113
cox1  ---------------------------------------------------ACACCTTACCTATGATAATTACCCGATGATTCTTCTCAACAAATCATAAAGATATTGGTACCCTATATTTA    71
```

trnY

cox1

1.2.5 trnW-trnA 50

trnW *trnA*

```
R     GCTAAATTAGACTAAGGACCTTCAAAGCCCAAAGCAGAAGTTAAAATCTTCTAATCTTTGAATAAGACTCGCAAGATTCTATCTAACATCCTTTGAATGCAACCCAAATACTTTAATTAAG-   121
trnW  G-TTA-TTAGACTAAGGACCTTCAAAGCCCTAAGCAGAAGTTAAAATCCTCTAATCTTTG--TTAGA-------------------------------------------------------    63
trnA  -----------------ACCTTTAAAATCCTAAATA-AAGATAAA-T--T-TAATCTTTG--TAAGACCTGTAAGACTTTATCTTACATCTTATGAATGCAACCCAGACACTTTAATTAAGC    98
```

trnW

trnA

1.3 NC_004594-NC_008448

Avg ovsize: 62
LCA: Galaxiidae-family

1.3.1 cox3-trnG 118

```
                              cox3                                                        trnG
R     GCACTTCGTTGACGTTGTCTGATTATTCTTGTATGTCTCAATTTATTGATGAGGCTCATAATCTTTCTAGTATTAAG-TTAGTATAAGTGACTTCCAATCACACGGTCTTGGTTAGAGTC  119
cox3  -CATTTCGTTGATGTCGTGTGACTTTTCCTTTATGCCTCTATTTATTGATGAGGCTCATAGTCTTTCTAGTACTAATATTAGTATAAGTGACTTCCAATCACGAAGTCTTGATTA-AGCC  118
trnG  --ATTTCGTTGATGTCGTGTGACTTTTCCTTTATGCCTCTATTTATTGATGAGGCTCATAGTCTTTCTAGTATTAATATTAGTATAAGTGACTTCCAATCACGCAGTCTTGGTTAAACCC  118
                              cox3
                                                                                          trnG
```

1.3.2 trnR-nad4l 112

```
                              trnR                                                        nad4l
R     TCTAAGAGAAGACCTCTGATTTCGGCTCAGACAATTATGGTTTAAGTCCATAACCGCCTTATGACTCCAGTACACTTTACCTTCACTTCAGCCTTTGTCCTAGGGCTAATAGGATTAG-C  119
trnR  TCCAAAACAAGACCTCTGATTTCGACTCAGAAAATTATGGTTTAAATCCATAACCCCCTTATGACCCCCATACATTTTACCTTCTCCTCAGCTCTTGTTCAAGGACTAACAGG-------  113
nad4l -CCAAAACAAGACCTCTGATTTCGACTCAGAAAATTATGGTTTAAATCCATAACCCCCTTATGACCCCCACCCTTTTTACCTTCTCTTCAGCTTTTATTCTAGGATTAATAGGTCTGGCC  119
                              trnR
                                                                                          nad4l
```

1.3.3 nad4-trnH -42

```
                                   nad4                                                       trnH
R     ACCTGCTCCCTGTAGTGCTGCTGGTGGCCAAGCCTGAGTTGATGTGGGG-TTGATCT-TTTTGTAGATATAGTTTAGCCAAAACATCAGATTGTGATTCTGAAAACAGGGGTTAAAC-CCCC   119
nad4  A-------------------------------------------------------------------------------------------------------------------------     1
trnH  -------------------------------------------GGGGGCCTGA-CTATTCTGTATATATAGTTTAATCAAAATATTAGATTGTGATTCTAAAGATAGGGGTTAAACTCCCC    78
      nad4
                                                                                             trnH
```

1.4 NC_011387-NC_011381

Avg ovsize: 59

LCA: Cyprinodontoidei-suborder

1.4.1 trnQ-trnM 66

```
                                trnQ                                                     trnM
R     GGACTTGAACCCTACCTGAAGAGATCAAAACTCTTAGTGCTTCCACTACACCACTTCCTAGTAAAGTCAGCTAAACAAGCTTTTGGGCCCATACCCCAAACATGTTGGTTAAAATCCT-   118
trnQ  GGGTTTGAACCCCACCTGAAGAGATCAAAACTCTTAGTGCTTCCACTACACTACTTCCTAGTAAAGTCAGCTAAATAAGCTTTTGGGCCCATACCCTAAACATGTTGGTTAAAATCCT-   118
trnM  ----------------------------------------------------ACTTCCTAGTAAAGTCAGCTAAATAAGCTTTTGGGCCCATACCCCAAACATGTTGGTTAAAATCCTT    67
                                trnQ
                                                                                         trnM
```

1.4.2 trnM-nad2 63

```
                                 trnM                                                  nad2
R     AGCTAAACAAGCTTTTGGGCCCATACCCCAAACATGTTGGTTAAAATCCTTCCTTTACTAATGAACCCTTTTATCCTTTCCACCCTTCTTCTAGGACTCGGCCTTGGAACAACCATCAC-   119
trnM  AGCTAAATAAGCTTTTGGGCCCATACCCCAAACATGTTGGTTAAAATCCTTCCTTTACTAATGA--------------------------------------------------------    64
nad2  -GCTAAATAAGCTTTTGGGCCCATACCCTAAACATGTTGGTTAAAATCCTTCCTTTACTAATGAACCCCTACATTTTATTTATTTTACTTTTTGCTCTAGGCCTCGGGACAACCACTACC   119
                                 trnM
                                                                                       nad2
```

1.4.3 nad1-trnI 48

```
                                 nad1                                                             trnI
R     CTCGTCATTTGACACCTCTCCCTCCCAATCGCCTTATCTGGCCTGCCCCCTCAACTTT-AGACGCT-GGAGCTGTGCCTG-AATCAAAGGGCCACTTTGATAGAGTGAATCATGAGGGTTAGAATCC   124
nad1  CTTGTAATCTGACATCTCTCATTACCAATTGCCTTCTTTGGTCTTCCCCCCCAACTTTAATATTTACAGAGATATGCCTGAAAT-AAAGGATCCCCTTGATAGAGTGCC-CCTTAG-----------   114
trnI  --------------------------------------------------------------------GAGATGTGCCTGAAATAAAAGGATCATTTTGATAGAATGCCTCATGAGGGTTTAAGTCC    59
                                 nad1
                                                                                                  trnI
```

1.5 NC_013571-NC_006345

Avg ovsize: 35

LCA: Tetrapoda-superclass

1.5.1 trnY-cox1 69

trnY *cox1*

```
R     AAGAGGCCTTATCCTCTGTGC-TTAGATTTACAG-TCTAATGCTTTACTCAGCCATTTTACCTATGTTCATTAACCGCTGACTATTCTCTACCAATCACAAAGACATCGGAACCCTATATCT-   120
trnY  AAAAGGATTAAACCTTTATAAATGAGAC-TACAGCTCACCACCTATT-TCGGTCACCTTATCTATAGT-ATTACCCGATAGCTCTTCTCAACAAAT-A-AA--ATATT-GTACCCT-TATTT-   113
cox1  -----------------------------------------------------CACCTTACCTATGATAATTACCCGATGATTCTTCTCAACAAATCATAAAGATATTGGTACCCTATATTTA    70
```

trnY

cox1

1.5.2 trnA-trnN 63

trnA *trnN*

```
R     CAAGACTACATCTTACATCTAGTGAGTGCAAATCAATTGCTTTA-AT-TAAGCTAAATCCTCAACTAGAT-TGATAGGATTCAAACCTATAAATTTTTAGTTAACAGCTAAACACCCTAAACTG-   121
trnA  ---GCTTA-AT-T-A-A---AGTGTCTGGGT-TGCATT-CATAAGATGTAAGATAAAGTCTT-AC-AGGTCTTATATAGTATTACCCGAT-AGCTCTTC-TCAACAAATAAA-------------    96
trnN  -------------------------------------------------AA-CTAAAACCTT-ACTAGAT-TAGTAGGCTTTTACCCTACGATCTTTTAGTTAACAACTAAATACCCACATCTGG    73
```

trnA

trnN

1.5.3 trnW-trnA -27

trnW *trnA*

```
R     TAGGATAATCAGTCCAAGAGCCTTCAAAGCCCTAAGTAGACCCACAAA-GTT-TAA-CTTCTGATAAGGACTGCAAGACTACATCT-TACATCTAGTGAGTGCAAATCAATTGCTTTAATTAAG   120
trnW  ---G-TTATTAGACTAAGGACCTTCAAAGCCCTAAGCAGAAGTTAAAATCCTCTAATCTT-TGTT-AGAATAAAAATTTTACATCTACAAATATAGT---------------------------    91
trnA  ----------------------------------------------------------------------------------------------------------------------------     0
```

trnW

1.6 NC_009061-NC_006355

Avg ovsize: 28

LCA: Labroidei-suborder

1.6.1 trnQ-trnM 55

```
                                      trnQ                                                      trnM
R     GGGCTCGAACCCCTCCTGGAGAGATCAAAGCTCTCAGTGCCTCCACTACACCACTTCCTAGTACGGTCAGCTAATTTAAGCTTCTGGGCCCATAC-CCCCGCCATGATGGTTAA-AATCC   118
trnQ  GGGCTCGAACCCTTCCTGAAGAGATCAAAACTCTTGGTGCTTCCACTACACCACTTCCTAGCAAAACAAGGAAACA-ATTCCATTGG--A-ATACACCCAAACAAGTTGATTAA------   110
trnM  -----------------------------------------------------------AGCAAAGTAAGCTAATAGAAGCTATTGGGCCCATAC-CCCAAAAATGTTGACTAATACTCA    60
                                      trnQ
                                                                                            trnM
```

1.6.2 trnM-nad2 23

```
                                      trnM                                                      nad2
R     GCTAATTTAAGCTTCTGGGCCCATAC-CCCCGCCATGATGGTTAAAATCCTTCCCTTACTG-ATGAACCCCTATATTCTAGGCACCCT-CTTGCTTGGGCTCGGCCTAGGTACAACCCTAAC-   119
trnM  GCTAATAGAAGCTATTGGGCCCATAC-CCCAAAAATGTTGACTAA------------------------------------------------------------------------------    44
nad2  ----------------------ATACACCCAAACAAGTTGATTAACTTCAA-CGATTGCTTCATGAGCCCCTATATTCTCTCGATCTTTCTTT-TCGGACTCGGCCTAGGAACCACAATTACA    99
                          trnM
                                                                                            nad2
```

1.6.3 trnI-trnQ 6

```
R      CTGAAATAAAGGGCCACTTTGATAGAGTGAATAATGAGGGTTAAAGTCCCTCCACCTCCTTAGGAAAAGGGGGCTCGAACCCCTCCTGGAGAGATCAAAGCTCTCAGTGCCTCCACTA-   118
trnI   CTGAATCAAAGGGTCACTTTGATAGAGTGAAGCATGGGGGTTAAAATCCTCCCAACTCCTT----------------------------------------------------------    61
trnQ   -------------------------------------------------------CCCCATAGAAAAAAGGGGCTCGAACCCTTCCTGAAGAGATCAAAACTCTTGGTGCTTCCACTAC    64
```

1.7 NC_022480-NC_010199

Avg ovsize: 25

LCA: Perciformes-order

1.7.1 trnL1-nad5 41

```
R      TAACAGTCATCCC-TTGGTCTTAGGAACCAAAAACTCTTGGTGCAAATCCAAGTAGCAGCTATGCACCCAACCCCTCTTTATAATAACAACCAGCCTGATCCTTATCTTTACCACTC-T-GTT   119
trnL1  -AATAGCCCTTCCATTGGTCTTAGGAACCAAAAACTCTTGGTGCAAATCCAAGTAGCAGCTA------------------------------------------------------------    61
nad5   ---------------------TAGTCACCAATGCC-CTTGGTTCAAATCCAAGTAGTAGCCATGCCCCCCTTACACATTACCTTAACATCCACCCTAA-CAATAACCCTCCTGCTCTTAGCC    99
```

1.7.2 trnH-trnS1 19

```
R      GTTTAACTAAAACATTAGATTGTGATTCTAAAAACAGGGGTTTAAGCCCCCTTGTCCACCGAAAGAGGCTTGCCCGCAACAGAGACTGCTAATCCCTGTCACTTTGGTTGAACTCCAAA-   119
trnH   GTTTAATAAAAACATTAGATTGTGATTCTAAAGACAGAAGTTAAAACCTCCTTATCCAC-------------------------------------------------------------    59
trnS1  ----------------------------------------TTAAAACCCGC--GCCCAC-GAGAGAGGCCTGCTGGCAACGAAGACTGCTAATCCTCATCCCCTCGGTTGAAATCCGAAG    77
```

1.7.3 nad4-trnH 16

```
R      ACATCCTTCCCCTCATACTACTCATCCTAAAACCAGAACTTATCTGATGCTGAACCTTTTGTAGACATAG-TTTAACTAAAACATTAGATTGTGATTCTAAAAACAGGGGTTTAAGCC-CC   119
nad4   ATCTCCTCCCCCTGCTTCTCCTGATTACAAAACCCGAGCTAATTTGTGGCTGAATTATATGTAGGTACAACTTTAA---------------------------------------------    76
trnH   ------------------------------------------------------------GTAGATATAG-TTTAATAAAAACATTAGATTGTGATTCTAAAGACAGAAGTTAAAACCTCC    60
```

1.8 NC_013136-NC_009851

Avg ovsize: 20

LCA: Acanthuroidei-suborder

1.8.1 trnY-cox1 59

```
     trnY                                                          cox1
R    AGGACTCAAACCTCTGTCTGTGGGGCTACAATCCACCGCTTAAA-CCTCAGCCATCCTACCTGTGGCAATCACACGTTGATTTTTCTCGACTAATCACAAAGACATCGGCACCCTCTATCT-   120
trnY -GGACTCGAACCTCTGTCTATGGGGCTACAATCCACCGCTTAAAAACTCAGCCATCCTACC-------------------------------------------------------------   60
cox1 --GACTCGAACCTCTGTCTATGGGGCTACAATCCACCGCTTAAAAACTCAGCCATCCTACCTGTGGCAATCACACGTTGATTCTTCTCGACTAATCACAAAGACATCGGCACCCTCTATCTA   120
     trnY
                                                                   cox1
```

1.8.2 trnC-trnY 2

```
     trnC                                                          trnY
R    -GGCAGGCAATTAGCCTACTACTTAAGATTTGCAATCTAATATGTTAAACACCTCGGAGCTTGGTAGAAAGAGGACTCAAACCTCTGTCTGTGGGGCTACAATCCACCGCTTAA-ACCTCA   119
trnC CGGCAAGCAGTTAGCTTGCGACTTCAGATTTGCAATCTGATATGTTTA-CACCTCAAGGCTTGGTAAGAAGAGGACTCGAACCTCTGTCTATGGGGCTACAATCCACCGCTTAAAAACTCA   120
trnY -----------------------------------------------------------------------------------------------------------------------CA   2
     trnC
                                                                                                                            trnY
```

1.8.3 nad2-trnW 1

```
     nad2                                                          trnW
R    TACTGCCTCCATTACCCTCCTACCACTCACCCCTACTCTTACCGCCCTACCAACCCTCTAAGAGACTTAGGATAACACCTAGACCAAGAGCCTTCAAAGCCCTCAGTGGGAGTGAGAAT-   119
nad2 CATGGCCACCATCTCACTGTTACCCCTCGCCCCCGCAGCAATTGCACTACTGACCCTCTA------------------------------------------------------------   60
trnW -----------------------------------------------------------A-GAGACTTAGGCTAACATCTAGACCAAGGGCCTTCAAAGCCCTCAGTGGGAGTGAGAATC   60
     nad2
                                                                   trnW
```

1.9 NC_008667-NC_008683

Avg ovsize: 20
LCA: Cyprinoidea-superfamily

1.9.1 trnI-trnQ 59

trnI *trnQ*

```
R     GAATGCCTAGGGACCACTTTGATAGAGTGGCCTATAGGGGCTAAAATCCCCTCAGTTCTTAGAAAGAAGGGGATCGAACCCATGCCCAAGAGATCAAAACTCTTAGTGCTTCCTCTA-   117
trnI  GAATGCCTAGGGACCACTTTGATAGAGTGGCCTATAGGGGCTAAAATCCCCTCAGTTCTT----------------------------------------------------------    60
trnQ  -AATGCCTAGGGACCACTTTGATAGAGTGGCCTATAGGGGCTAAAATCCCCTCAGTTCTTAGAAAGAAGGGGATCGAACCCATGCCCAAGAGATCAAAACTCTTAGTGCTTCCTCTAC   117
```

trnI

trnQ

1.9.2 nad1-trnI 2

nad1 *trnI*

```
R     CTAGTACTATGACACATTGCCCTGCCAATCGCACTAGCAGGCCTCCCCCCACAACTATAATTCAGGAACTGTGCCCGAATGCCTAGGGACCACTTTGATAGAGTGGCCTATAGGGGCTAAAAT-   123
nad1  CTAGTACTATGACACATTGCCCTGCCAATCGCACTAGCGGGCCTTCCCCCACAACTATAGTTCAGGAACTGTGCCCGAATGCCTAGGGACCACTTTGATAGAGTGGCCTATAGGGGCTAAAAT-   123
trnI  -------------------------------------------------------------------------------------------------------------------------ATC     3
```

nad1

trnI

1.9.3 rrnS-trnV -1

```
                                      rrnS                                                         trnV
R     GACAAGGGGAGGCAAGTCGTAACATGGTAAGTGTACCGGAAGGTGCACTTGGATTAAACCCAGGGCGTGGCTGAGTTAGTCAAGCATCTCACTTACACCGAGAAGACATCCATGCAAGT-   119
rrnS  GACAAGGGGAGGCAAGTCGTAACATGGTAAGTGTACCGGAAGGTGCACTTGGATTAAACC------------------------------------------------------------    60
trnV  -------------------------------------------------------------AGGGCGTGGCTGAGTTAGTCAAGCATCTCACTTACACCGAGAAGACATCCATGCAAGTT    59
                                      rrnS
                                                                                                   trnV
```

1.10 NC_005800-NC_005796

Avg ovsize: 20

LCA: Albuliformes-order

1.10.1 trnL1-nad5 59

```
                                      trnL1                                                         nad5
R      AACAGCTGATCCGTTGGTCTTAGGAACCAAAGACTCTTGGTGCAAATCCAAGTAGAGGCTATGCATTTATCAACTTTAGGTTTTAATTCGAGT-TCCTTAACTGTATTTGTACTACTCCT-   119
trnL1  AATAGCTCATCCGTTGGTCTTAGGAACCAAAAACTCTTGGTGCAACTCCAAGTAGAGGCT-------------------------------------------------------------    60
nad5   -ATAGCTCATCCGTTGGTCTTAGGAACCAAAAACTCTTGGTGCAACTCCAAGTAGAGGCTATGCACTCAACAACCCTCATCTTCAATTCAAGCCTCCT-AATTATCTTCGCGCTCCTTATT   119
                                      trnL1
                                                                                                    nad5
```

1.10.2 trnS1-trnL1 3

trnS1 *trnL1*

```
R      CTATGAGGCGTTGGGAACTGCTAATTC-TTAGTATCATGGTTAGAATCCATGGCTCGCTCGAGCCCCTATAGGATAACAGCTGATCCGTTGGTCTTAGGAACCAAAGACTCTTGGTGCAAA   120
trnS1  CT-GGAAGCACTGGGGACTGCTAATCCCTCAGCACCACGGCTAAACTCCGTGGCTCACTCG-GCCCCTAAAGGATAATAGCTCATCCGTTGGTCTTAGGAACCAAAAACTCTTGGTGCAA-   118
trnL1  ---------------------------------------------------------------------------------------------------------------------CAAA     4
```

trnS1

trnL1

1.10.3 nad4-trnH -2

nad4 *trnH*

```
R      ATCTGATTCCAGTAATGCTTTTGGCTTTAAAGCCTGAGTTAGTGTGGGGTTGGTGTCTATGCAGACATAGTTTAAACAAAATGCTAGATTGTGATTCTAGCGATAGGAGATAGAACCTC-   119
nad4   ACTTAATTCCAGTATTACTTCTAGTACTGAAGCCCGAACTAATATGAGGCTGATGCATAT------------------------------------------------------------    60
trnH   --------------------------------------------------------------AGATATAGTTTAAAAAAGACGTTAGATTGTGATTCTAAAAATAGGAGATAAAACCTCT    58
```

nad4

trnH

1.11 NC_012761-NC_012762

Avg ovsize: 19

LCA: Lorisiformes-infraorder

1.11.1 trnI-trnQ 59

```
trnI (R, columns 1–60) | trnQ (R, columns 61–117)
R     TCTGACAAAAGAGTTACTTTGATAGAGTAAAACATAGAGGTGCAAACCCTCTTATTTCTAGGACAGCAGGCCTTGAACCTACCCCTGAGAACTCAAAATTCTCCGTGCTACCTA-CT  116
trnI  TCTGACAATAGAATTACTTTGATAGAGTAAAACATAGAGGTGCGAACCCTCTTATTTCTA---------------------------------------------------------  60
trnQ  -CTGACAATAGAATTACTTTGATAGAGTAAAACATAGAGGTGCGAACCCTCTTATTTCTAGGATAATAGGACTTGAACCCACTCTTAAGAACTCAAAATTCTCCGTGCTACCTATCA  116
trnI | trnQ
```

1.11.2 nad1-trnI 2

```
nad1 | trnI
R     CTATGCATATGATATGTTTCACTACCCATTATCTTAGCCAGCATTCCCCCCCAAACATAGAAATATGTCTGACAAAAGAGTTACTTTGATAGAGTAAAACATAGAGGTGCAAACCCT  117
nad1  ATATGCATATGACACGCCTCACTACCCATCTCCTTGGCCAACATCCCCCCTCAAACATAGAAATATGTCTGACAATAGAATTACTTTGATAGAGTAAAACATAGAGGTGCGAACCC-  116
trnI  ------------------------------------------------------------------------------------------------------------------CC-  2
nad1
trnI
```

1.11.3 trnL2-nad1 -2

```
trnL2 | nad1
R      CCTGGCAATTGCATAAAACTTAAAACTTTAGAAACAGAGGTTCAACTCCTCTTCTTAACATATGTTTATAATAAACCTCCTGCTACTTATTATCCCCATTATTCTAGCCATAGCTTTCCT-  120
trnL2  CCCGGTAATTGCATAAAACTTAAAACTTTACCACCAGAGGTTCAACTCCTCTTCTTAACA-------------------------------------------------------------  60
nad1   --------------------------------------------------------------TGTTTGTTCTAAACCTCCTACTACTCATCATTCCCATTATCCTGGCCATAGCCTTCCTC  59
trnL2
nad1
```

1.12 NC_006333-NC_006340

Avg ovsize: 19

LCA: Batrachoseps-genus

1.12.1 trnA-trnN 51

```
                                  trnA                                                    trnN
R     CAAGACTTTATTCCACATCTTCTGAATGCAACCCAGACACTTTAATTAAGCTAAAACCCTC-CTAGATTAGTAGGCTTTTATCCTACTACCTTTTAGTTAACAGCTAAATACCTAATATCA-   120
trnA  CGAGACTCTACCCCACATCTTCTGAATGCAACCCAGACACTTTAATTAAGCTAAAGCCTTCACAA-ACTA-CAC-CTCATATTCTTGTA--ATTTAATAAACAACTAA--------------   103
trnN  ---------------------------------------------------------CTTCCCTAGATTAACAGGATTTTATCCTACTACAATTTAGTTAACAGCTAAACACCAGATTTCAG    65
                                  trnA
                                                                                          trnN
```

1.12.2 trnY-cox1 7

```
                                  trnY                                                    cox1
R     -AAAA-GGACTACACCTTTATAATTGAGGCTACAACTCAGCACCTACTTCGGCCATCTTACCTATGATAGTTACGCGATGACTTTTTTCAACAAACCACAAAGATATCGGCACCCTTTACCT-   120
trnY  GGAAAGGGAC--CACCTTTATAAATGAGGCTACAACTCAACACCTATTTCGGCCACCTTACCT-----------------------------------------------------------    61
cox1  -------------------------------------------------------CTTACCTGTGATAATTACTCGATGACTATTTTCAACAAACCATAAAGATATCGGCACCTTATATTTA    67
                                  trnY
                                                                                          cox1
```

1.12.3 trnC-trnY 1

```
                        trnC                                                   trnY
R     GTAGAAG-T-CATTCTGCTCTTTAAAATTTGCAATTTTATGTGCT-GTACACCACGAAACTTGGCAAGAAAAG-GACTACACCTTTATAATTGAGGCTACAACTCAGCACCTACTTCGGCC--   117
trnC  -TGGCAGATACACTCTGCTCCTTAAAATTTGCAATTTCATGTGCTACTACACTAC-A------------------------------------------------------------------    55
trnY  --------------------------------------------------------ACACT-GGTAAGGAAAGGGAC--CACCTTTATAAATGAGGCTACAACTCAACACCTATTTCGGCCAC    64
                        trnC                                                   trnY
```

1.13 NC_002647-NC_003159

Avg ovsize: 19

LCA: Stomiatiformes-order

1.13.1 trnY-cox1 51

```
                        trnY                                                   cox1
R     AGGAGTTTAACCTCTGTTTATGGGGCTACA-ATCCACCGCTTAACACTCAGC-CAC-CCTACCTGTGGCAATCACACGCTGATTTTTCTCAACCAATCACAAAGACATTGGCACCCTGTA-TTT   120
trnY  AGGACTTAAACCTCTCTTTATGGGGCTACA-ACCCACCACTTAAATCTCAGC-CAC-CCTACCT------------------------------------------------------------    61
cox1  -------------CTGCTTATAGG-CTACACATCC-CAACTCGGGTTT-AGCCCTCACCCACCTGTGGCAATTACACGCTGATTCTTCTCCACAAACCACAAAGACATTGGCACCCTTTACTTA   108
                        trnY                                                   cox1
```

1.13.2 trnN-trnC 4

```
                                     trnN                                                                                      trnC
R     CCTCGATCCTACAAAGTCTTAGTTAACAGCTAAGCGCTCCAACCAGCGAGCATCAGTCTA-CTCTCCCCGCCGCCGGGGCG-CGCGGGGAAAGCCCCGGCGGGCGGTTAGGCCTGCTTCTTCAGGTTT   126
trnN  CCTCGATCCCCCAAACTCTTAGTTAACAGCTAAGCGCCCCAACCAGCGAGCATCCATCTAACTGACC-AGCC--------------------------------------------------------    71
trnC  --------------------------------------------------------------------CGCCGCC-GGGCGTCGAC-GGAAAGCCCCGGCAGGCTGGAAA-CCTGCTTCTTCAGATTT    57
                                     trnN
                                                                                                                            trnC
```

```
          trnC
R     GCAATCTGACGTGTCACACCT--   147
trnN  -----------------------    71
trnC  GCAATCTGACGTGTCACACCTCA    80
           trnC
```

1.13.3 trnC-trnY 4

```
                                     trnC                                                      trnY
R     C-GGCGGGCGGTTAGGCCTGCTTCTTCAGGTTTGCAATCTGACGTGTCACACCTCGGGGCTTGGCAGGGAGAGGAGTTTAACCTCTGTTTATGGGGCTACAATCCACCGCTTAACACTC--   118
trnC  CCGGCAGGCTGGAA-ACCTGCTTCTTCAGATTTGCAATCTGACGTGTCACACCTCAGGGCTT-----------------------------------------------------------    61
trnY  ----------------------------------------------------------GCCTGGTAGGAAGAGGACTTAAACCTCTCTTTATGGGGCTACAACCCACCACTTAAATCTCAG    63
                                     trnC
                                                                                               trnY
```

1.14 NC_006355-NC_011381

Avg ovsize: 18

LCA: Percomorpha-order

1.14.1 nad1-trnI 48

nad1 (R) · *trnI* (R)

```
R     TTTATAATCTGACATCTGGCACTACTAATTGCATTCGCCGGACTTCCCCCACACCTTTAA----CA-GGAGCTGTGCCTG-AATCAAAGGGTCACTTTGATAGAGTGAAGCATGGGGTTAAAATCC   121
nad1  CTTGTAATCTGACATCTCTCATTACCAATTGCCTTCTTTGGTCTTCCCCCCCAACTTTAATATTTACAGAGATATGCCTGAAAT-AAAGGATCCCCTTGATAGAGTGCCCCTT--------------   112
trnI  -----------------------------------------------------------------ACGGAGATGTGCCTGAAATAAAAGGATCATTTTGATAGAATGCCTCATGAGGGTTTAAGTCC    62
```

nad1 (nad1) · *trnI* (trnI)

1.14.2 trnI-trnM 14

trnI (R) · *trnM* (R)

```
R     CTGA-ATCAAAGGGTCACTTTGATAGAGTGAAGCATG-GGGG-TTAAAATCCTCCCAACTCCTTCCACGGAGCAAAGTAAGCTAATAGAAGCTATTGGGCCCATACCCCAAAAATGTTGACTAA--TA   123
trnI  -TGAAATAAAAGGATCATTTTGATAGAATGCCTCATGAGGGT-TTAA---------------------------------------------------------------------------------    45
trnM  ---------------------------------CCTGAGGGGATTAAAA-C-TCTTAG-TACTTCCT---AGTAAAGTCAGCTAA-ATAAGCTTTTGGGCCCATACCCCAAACATGTTGGTTAAAATC    88
```

trnI (trnI) · *trnM* (trnM)

trnM (R)

```
R     CTC   126
trnI  ---    45
trnM  CTT    91
```

trnM (trnM)

1.14.3 trnM-trnQ -8

```
                                    trnM                                                                  trnQ
R     -GCTAATAGAAGCTATTGGGCCCATACCCCAAAAATGTTGACTAATACTCAGCCTTTGTTACTTTTCCCCATAGAAAAAAGGGGCTCGAACCCTTCCTGAAGAGATCAAAACTCTTGGTGCTTCCACT   127
trnM  AGCTAA-ATAAGCTTTTGGGCCCATACCCCAAACATGTTGGTTAAAATCCTTCCTTTACTAAT-----------------------------------------------------------------    62
trnQ  -----------------------------------------------------------------------TAGAAAGAAGGGGTTTGAACCCCACCTGAAGAGATCAAAACTCTTAGTGCTTCCACT    57
                                    trnM
                                                                                                          trnQ
```

```
      trnQ
R     A-   128
trnM  --    62
trnQ  AC    59
      trnQ
```

1.15 NC_003159-NC_003163

Avg ovsize: 10

LCA: Neoteleostei-subclass

1.15.1 trnE-cob 26

```
                                    trnE                                                            cob
R     CAGGACTCTAACCAAGACCAGCGACTTGAAAAACCGCCGTTGTTTTTCAACTACAAGAACCCA-ATGGCCAGCCTACGAAAAACCCACCCCCTTCTAAAATTGCCAACCACGCACTAGTAGA   122
trnE  CAGGATTTTAACCTGGACCAATGACATGAAAAGCCACCGTTGTGATTCAACTATAAGAACCCATCCGGGCT-C-TGCCACAAAC--------------------------------------    82
cob   ----------------------------------------------------------ACCCA-CCCGACA-C-TACGCAAAACCCACCCCCTCCTAAAATCGCAAACGATGCCC---T--A    57
                                    trnE
                                                                                                  cob
```

1.15.2 trnT-trnP 14

```
                                trnT                                                                 trnP
R     AACATGTCGAGCACCGGACTTGTAATCCGGGGGTCGGGGTTTAAATCCCCCCTAGTGCTCACCCC-GCC-C-GACTCAGAGAGGGGAGATTTTAACTCCCACCCTTAGCTCCCAGAGCTAA-GATCC   124
trnT  AGTATCCAGAGCGCCGGTTTTGTAACCCGGAGGCCGGAAGTTCAAATCTCCCCGAGCGCACAGCCTAGCC-CAGGCTC-------------------------------------------------    77
trnP  ---------------------------------------------------------------CT-GCCACAAACTCAAAACGACAAGACTTACACTCGCACTTCCGGCACCCAAAGCCGGTGCTCT    63
                                trnT                                                                 trnP
```

```
        trnP
R     TAAACTAG   132
trnT  --------    77
trnP  TAATTAAA    71
        trnP
```

1.15.3 nad5-nad6 -10

```
                                nad5                                                       nad6
R     AAAACCTACCTCCGCCTCTCCCTTCTAACACT-AGCTCTAGCCACCTTAATTTTTGTTTAAACGGCCCGCAAAGTCCCTCGACTTAACCCCCGGGTTAACTCAAGCACTCCTAAT-A   115
nad5  --TACCTA--TCC-C-TATTCTTTATAACCCTTTGC-CTAGCCACCCT---------------------------------------------------------------------    41
nad6  ----------------------------------------------------------TAAACGGCACGCAAGGCCCCTCGACTCAACCCCCGAGTTAACTCTAGAACCACAAATAA    59
                           nad5                                                         nad6
```

1.16 NC_008797-NC_007781

Avg ovsize: 8

LCA: Neogastropoda-infraorder

1.16.1 trnL1-trnL2 14

```
                                         trnL1                                                                   trnL2
R       -CA-AAGAATTATGCGATT-GATTTAGGATCAATAGACATAGGTTAAAATCCTTTATTTGATAGAAGGAATAGAGGTGGCAGA-TTAAGTGCATTAGGTTTAAGCCCTAAAGATAAAGATGAAAATTC   124
trnL1   GCAGATGAAGT--GC-ATTAGGTTTAAGCCCTAAATATAAAGATGAAATTTCTTT-------------------------------------------------------------------------    52
trnL2   -----------------------------------------GATTAAATTTAATTAACAATTA-AATGATCAAA-TTAGCAAAATTAA-TGCAATTGACTTAGGATCAATAGATATAGGTGAAATTCC    84
                                    trnL1                                                                      trnL2
```

```
        trnL2
R       T-    125
trnL1   --     52
trnL2   TT     86
        trnL2
```

1.16.2 rrnL-trnL1 10

```
                                       rrnL                                                                     trnL1
R       GAAAGGACCGGCGTAACATATAATATATTTTTATTAGATCAAATATAATTT-AACTTTGAAATCAAATTAGCAAA-GA-ATTA-TG-C-GATT-GATTTAGGATCAATAGACATAGGTTAAAA-TC-C   119
rrnL    GCCAGGTCA-G-TTT-C-TATCTT-TAATTATAT-AGAT-AAGTTTAGTACGAAAGGACCAATTT-ATCATAAAACGATATTACTTACTGATTAAATTTA----------------------------    92
trnL1   ------------------------------------------------------------------------------------------ATTAGGTTTAAGCCCTAAATATAAAGATGAAATTTCTT    38
                                          rrnL
                                                                                                                  trnL1
```

1.16.3 trnL2-nad1 2

trnL2 / *nad1*

```
R     GCA-GATTAAGTGCATTAGGTTTAAGCCCTAAAGATAAAGATGAAAATTCTTTCCTTTATAATGTATCTAAGAATTATTTCCTGTTTGTTTACTTATGTTTGTATTTTACTAGCGGTCGC-   119
trnL2 GCAAAATTAA-TGCAATTGACTTAGGATCAATAGATATAGGTGAAATTCCTTTATTTGATAAT----------------------------------------------------------    62
nad1  -------------------------------------------------------------ATGTATGTTTCTATCATYTCAACAGTATTTACCTATATCTGTATTTTATTGGCAGTCGCT    60
```

trnL2

nad1

1.17 NC_005961-NC_006355

Avg ovsize: 6

LCA: Teleostomi-superclass

1.17.1 trnM-nad2 41

trnM / *nad2*

```
R     AGTCAGCTAAGCAAGCTATTGGGCCCATAC-CCCAAAAATGTT-A-TAC-AC--CAACCTCTACTACACACACCATGACCACAACCTCATGAACAATAATAACACTAAACATCACACTAGGAACC-CT   121
trnM  GCTAA--TA-G-AAGCTATTGGGCCCATAC-CCCAAAAATGTTGACTAATACT-CAGCCTTTGTTAC-------------------------------------------------------------    61
nad2  --------------------------ATACACCCAAACAAGTTGA-TTA-ACTTCAACGATTGCTTCAT-GAGC-CC-CTATATTCTCTCGATCTTTCTTTC-GGA--C-TCGGCCTAGGAACCACA    93
```

trnM

nad2

nad2

```
R     CTTAGC   127
trnM  ------    61
nad2  ATTACA    99
```

nad2

1.17.2 trnQ-trnA 8

```
                         trnQ                                                          trnA
R     -A-CTCGAACCCGCACCTGAA-AGATCAAAACTTCCAGTACTACCTCATTGTACTACCCCTTACTAATAGAACTCGC-GACAACTCATC-ACATCTCCTGCATGCAACACAGACACTTTAATTAAGCC   123
trnQ  GGGCTCGAACCCTT-CCTGAAGAGATCAAAACTCTTGGTGCT-TC-CA--CTAC-ACCACTTCCTAGCAAAAC-------------------------------------------------------    67
trnA  -----------------------------------------------------------------AATAAAACTTGCAGGGGTCTCACCTACATCTTCTGCTTGCAAAGCAGACACTTTAATTAAG-C    62
                          trnQ
                                                                                   trnA
```

1.17.3 trnW-trnQ -30

```
                       trnW                                                         trnQ
R     GGTTATACACCCATTAAACCTGAGGCCTTCAAAGCCCCCAAAAAACAATTTTAGCTTCTGCTACACTAAAGAAGCAGGACTCGAACCCGCACCTGAA-AGATCAAAACTTCCAGTACTACCTC-AT   124
trnW  ----ATA-ACC--TCAGACCAAGGGCCTTCAAAGCCC-----------------------------------------------------------------------------------------    30
trnQ  -------------------------------------------------------------------AAAAAAG--GGGCTCGAACCCTT-CCTGAAGAGATCAAAACTCTTGGTGCTTCCACTAC    56
                trnW
                                                                                     trnQ
```

1.18 NC_020000-NC_006405

Avg ovsize: 5

LCA: Anura-order

1.18.1 trnL1-nad5 14

trnL1 *nad5*

```
R      AATAGTCTATCCATTGGTCTTAGGCGCCAAAATTTCTTGGTGCAACTCCAAG-TGAAAGCTATGAATTTAATAATTTTTAATTCCTCTATTCTCCTATCCCTTATT-TCCTTAC--TTT-ACCC   119
trnL1  AATAGCCCTTCCCCTGGTCTTAGGCTCCAGTATCTCTTGGTGCAAATCCAAG-TAAAAGCTA--------------------------------------------------------------   61
nad5   ------------------------------------------------CAAGTTAAAAGTCATGGACCTCCTCCTGGTCACCTCATC-ATGC-CTTATT-ATAACCATCATTACAATTTCACTC   73
```

trnL1

nad5

1.18.2 trnS1-trnL1 7

trnS1 *trnL1*

```
R      ACTGGGTAAACGAGAACTGCTAATTACTCGCCCCCGCGGTTCAATTCCGCGGCTTTCCCG--GCTTTTAAAGGAAAATAGTCTATCCATTGGTCTTAGGCGCCAAAATTTCTTGGTGCA-AC   119
trnS1  TCTGGGAAAACGAGAACTGCTAATTCCTCGCGTCCATGGTTCAATTCCATGGCTTGCTCGAAACTTTTA-----------------------------------------------------   69
trnL1  --------------------------------------------------------------GCTTTTAAAGGAAAATAGCCCTTCCCCTGGTCTTAGGCTCCAGTATCTCTTGGTGCAAAT   60
```

trnS1

trnL1

1.18.3 cob-trnT -5

cob *trnT*

```
R      -TCTATTCCT-TATCCTTATTCCGGCTGGGGGTTGAATAGAAAACAAACTA-TTAA-CTAACTAGCCTTAG-TAGCTTA-ATTAAAGCATCGGCCTTGTAAGCCGAAGAATGGAGACTAACACTC-   119
cob    ATCT-TTGCTCCATTCTTC-CCCATCATTGGACT-CTTAGAAAACAAGTTACTTCAGCT-------------------------------------------------------------------   56
trnT   ----------------------------------------------------------------GCCC-AGATAGCTTATATTAAAGCATCGGTCTTGTAAACCGAAGATTGTAGATTAA-ACCCT   60
```

cob

trnT

1.19 NC_006355-NC_022477

Avg ovsize: 4

LCA: Perciformes-order

1.19.1 trnQ-nad2 12

```
                                    trnQ
R     GGGCTCGAACCCTTCCTGAAGAGATCAAAACTCTTGGTGCTTCCACTACACCACTTCCTAGCAAAACAAG-GAAACAA--TTCCATTGGAATACACCCAAACAAGTTGATTAACTTCAACGATTGCTT   125
trnQ  GGATTCGAACCCAACCTAAAGAGATCAAAACTCTCAGTGCTTCCTCTACACCACTTCCTAGTAAAGTCAGCTAAAAAAGCTTTCAGGCCCATAC-CCTGAACATGTTGGTTAA----AACCCTTCCCT   123
nad2  --------------------------------------------------------------------------------------------------------------------------------     0
                                    trnQ

                  nad2
R     CATGAGCCCCTATATTCT--CTCGATCTTTCTTTTCGGACTCGGCCTAGGAACCACAATTAC-   185
trnQ  TACTA-CACCTCTATCCT---------------------------------------------   140
nad2  ------CCCCT-TATCCTGGCTCTA-CT-ACTATTTAGCCTCGGCCTAGGCACCATCCTCACA    54
                  nad2
```

1.19.2 trnM-trnQ 2

```
                                    trnM                                               trnQ
R     GCTAATAGAAGCTATTGGGCCCATACCCCAAAAATGTTGACTAATACTCAGCCTTTGTTACTTTTCCCC-AT-AGAAAAAAGGGGCTCGAACCCTTCCTGAAGAGATCAAAACTCTTGGTGCTTCCAC   126
trnM  GCTAA-ATAAGCTTTCGGGCCCATCCCCCGAACATGTCGGCCAACAATC--CCTCCTTTACT------------------------------------------------------------------    59
trnQ  ------------------------------------------------------------CTTTAAACCTATTAGAAAGAAGGGATTCGAACCCAACCTAAAGAGATCAAAACTCTCAGTGCTTCCTC    68
                                    trnM                                               trnQ
```

```
      trnQ
R     TA-   128
trnM  ---    59
trnQ  TAC    71
      trnQ
```

1.19.3 nad1-trnI 0

```
      nad1                                                          trnI
R     TTTATAATCTGACATCTGGCACTACTAATTGCATTCGCCGGACTTCCCCCACACCTTTAACAGGAGCTGTGCCTGAATCAAAGGGTCACTTTGATAGAGTGAAGCATGGGGGTTAAAATC-C   121
nad1  TTAATAGTATGACACCTCGCCCTCCCAATCGCATTCTCAGGCTTCCACCCCAAATATAATA------------------------------------------------------------    62
trnI  --------------------------------------------------------------GGAGCCGTGCCTGAACCAAAGGATCACTTTGATAGAGTGAATCACGAGGGTTAAAACCCC    60
      nad1
                                                                    trnI
```

1.20 NC_022670-NC_002735

Avg ovsize: 3

LCA: Atelocerata-superclass

1.20.1 cob-trnS2 8

cob *trnS2*

```
R      TTT-TATTTTATTCATTATCATGCCTATCACCTCAAAAACATGAGATAAAATACTTGATTAAGTTAATTAAGCTTTAGAAAGCGAGTACCTTGAAAGTACTAGAA-GATAGTTAAATTCTA   119
cob    TTTATATATT-TT-ATTTTAAACCCAATAATTTCCTATCTTTGAGATT-A-TA-AT--TTAAATTAA-TAA--------------------------------------------------    63
trnS2  ---------------------------------------------------------------TTAACTAAATGTT--AA-GTAAATATCTTGAAAATATTATAAAGA-AGTTTGATTCTT    54
```

cob *trnS2*

1.20.2 trnS2-nad1 5

trnS2 *nad1*

```
R      AGCTTTA-GA-AAGCGAGTACCTTGAAAGTACTAGAA-GATAGTTAAATTCTATC-ATTAACTTACCATA-CACTTAT-TTTAATGGTTATAA-AATAAGATATATTACTATACCAACAATAAATAAT   121
trnS2  --CTAAATGTTAAGTAAATATCTTGAAAATATTATAAAGA-AGTTTGATTCT-TCTAGAAATTTAGCTTATCTTTTATATATAA--------------------------------------------    80
nad1   -------------------------------------------------------------------------------TTTAAAAATTTCAACAGTTA-A-ACATAACTCCAACAACAAAGTATAA-    46
```

trnS2 *nad1*

nad1

```
R      AGACAATTCAAAGAAACAG   140
trnS2  -------------------    80
nad1   A-ATAAGT-ATATTTA-A-    61
```

nad1

1.20.3 trnI-trnM -2

```
                               trnI                                                        trnM
R     -AGATGCCTGA-ATAAAGGATTACTTTGATAGAGTAAATCATGCACAATTATGCTCTTACTATAAATAATTAGATAAGCTAATATTAAGCTTATAGGTTCATACCCTATCAATAGAAGTAAAAC---C   123
trnI  TAG-TGCCTGATAAAAAGGAGTATCCTGATAGGGTAAATTATGCACA-CTATGC-CTT----------------------------------------------------------------------   55
trnM  ------------------------------------------------------------TATATATAA-AAGATAAGCTAATA--A-GCTACTGGGCCCATACCCCTTCTATGTAAGTAAATCTTAC   64
                              trnI                                                        trnM
```

```
      trnM
R     T-   124
trnI  --    55
trnM  TC    66
      trnM
```

1.21 NC_012689-NC_022670

Avg ovsize: 2

LCA: Neoptera-subclass

1.21.1 trnI-nad2 6

```
                                trnI                                                        nad2
R     ---TGCCTGACAAAAGGATTATTTCGATAAAATAAATCATATATATAAATTATATCTTTATTAATTAATAAATTTTATTACAA-ATTCAACTTGTCCTGA-TTTAAT-A-C--AACAGTAATTTTC-C   118
trnI  AGATGCCTGAATAAAGGATTACTTTGATAGAGTAAATCATGCACA-A--TTATG-CTCT-T--ACT-ATAAAT-------------------------------------------------------   65
nad2  -------------------------------------------------------------------ATAAATATTAAAACAATAAT-AACAAGAC-TAACTTTAAGAACCCTTATAGTAATATCATC   59
                                trnI                                                        nad2
```

```
         nad2
R         C    119
trnI      -     65
nad2      A     60
         nad2
```

1.21.2 cob-trnM 2

```
                                   cob                                                              trnM
R     TTCGCATACT-TTCTAATATTAGAACTACCA-TT-AAAATATTGAGATAAACTATTATATTAATTTAAAAAAGATAAGCTAAT--TAAGCTATTAGACTCATACTCTAACTATAGAAATATCAATTTT   123
cob   TT-TTATTTTATTC-ATTATCATGCCTATCACCTCAAAAACATGAGATAAAATACTTGATTA------------------------------------------------------------------   60
trnM  ------------------------------------------------------------TAAAT-AAT-TAGATAAGCTAATATTAAGCTTATAGGTTCATACCCTATCAATAGAAGTA-AAACCTT   65
                                   cob                                                              trnM
```

1.21.3 trnM-trnS2 -1

```
                                 trnM                                                          trnS2
R      AGCTAAT--TAAGCTATTAGACTCATACTCTAACTATAGAAATATCAATTTTCTTCTTTTTAAGTTAATTA-GTTAAATATAAACTTATATCTTGAAAATA-TAAAATA-AATAATAATT-TATT   119
trnM   -GCTAATATTAAGCTTATAGGTTCATACCCTATCAATAGAAGTA-AAACCTTCTTCTAATTA---------------------------------------------------------------   60
trnS2  ---------------------------------------------------------------GTTAATTAAGCTTTAGA-AAGCGAGTACCTTGAAAGTACTAGAAGATAGTTA-AATTCTA-T   59
                                 trnM                                                          trnS2
```

1.22 NC_006917-NC_015076

Avg ovsize: 1

LCA: Jenkinsia-genus

1.22.1 trnQ-trnM 3

```
                 trnQ                                                        trnM
R     GGACTCGAACCCATCCTCTAGAGATCAAAACTCTAGGTGCTTCCACTACACCACTTTCT-AGTAGGGTCAGCTAAATAAGCTTTTGGGCCCATACCCCAAACATGTTGGTTAGA-ATCCT   118
trnQ  GGATTTGAACCCAGCCCGCAGAGATCAAAACTCTGAGTGCTCCCGCTACACCACTCTCTGAAT---------------------------------------------------------    63
trnM  ------------------------------------------------------------AATATGGTAAGCTAAAAAAGCTTTCGGGCCCATACCCCGAATATGTTGGTTAAACCCCCT    60
                 trnQ                                                        trnM
```

1.22.2 nad1-trnI 3

```
                 nad1                                                        trnI
R     TCTTGTCCTCTGGCACATTGCCCTCCCCGTCGGGGCCGCTGGTCTCCCACCCCAGTTCTAGGAGTTATGCCTGAATGTCTAAGGACCACTTTGATAGGGTGCGTTATGAGGGTTAAAGT-   119
nad1  T-TCGTCGTCTGACACCTTTCCTTCATTGTCAGCACTGCCGCACTTCCCCCTCAATT-TAGGA---------------------------------------------------------    61
trnI  ------------------------------------------------------------GGAACTGTGCCTGACCGCTTAAGGGGCACTTTGATAGAGTGACTAAAGGGGGTTGAACTC    60
                 nad1                                                        trnI
```

1.22.3 trnI-trnQ -1

```
                                 trnI                                                          trnQ
R     GAATGTCTAAGGACCACTTTGATAGGGTGCGTTATGAGGGTTAAAGTCCCTCTGACTCCTCATTAGAAAGAAGGGACTCGAACCCATCCTCTAGAGATCAAAACTCTAGGTGCTTCCACTA-   121
trnI  GACCGCTTAAGGGGCACTTTGATAGAGTGACTAAAGGGGGTTGAACTCCCCCCAGCTCCT--------------------------------------------------------------    60
trnQ  -------------------------------------------------------------ATTAGAAAGCCGGGATTTGAACCCAGCCCGCAGAGATCAAAACTCTGAGTGCTCCCGCTAC    61
                                 trnI                                                          trnQ
```

1.23 NC_007690-NC_007689

Avg ovsize: 1

LCA: Crinozoa-subphylum

1.23.1 atp6-cox3 4

```
                                  atp6                                                              cox3
R     TGTATTCAAGCTTATGTTTTTACTACATTAATTCATTTTTATTTACAGGA-AAATCTTTAAAAATTTTTAAAATGAATCATCAACATCCATATCATTTGGTAGATCAAAGTCCATGACCTTTGATAGC   127
atp6  TGTATACAGGCTTATGTTTTTACAGCTTTGGTTCATTTTTATTTACAAGAGAAAT-TTTA--GTTTTTTA----------------------------------------------------------    67
cox3  ------------------------------------------------------------------TTTA--ATGAGTTATCAACATTCTTATCATTTGGTTGAGCAGAGTCCTTGGCCTATAATGGC    60
                                   atp6                                                            cox3
```

```
      cox3
R     TTC-   130
atp6  ----    67
cox3  TTCA    64
      cox3
```

1.23.2 trnR-nad4l 3

trnR *nad4l*

```
R      GTTTAATAAAAATTTTTGGTTTCGGCCCTTATGCTTTTGGTTAAAGTCCAAACTCTTTTTG-TGAAGTTTTTTTTATTTTTATGTGTTTTATTTTTTTCTTAGGTTGTGTT-GGTGTAAT-   119
trnR   GTTTAAGTAAAACTTTTGGTTTCGGCCCGTTTATTTTTGGTTATAGTCCAAAC-CTTT----------------------------------------------------------------   57
nad4l  -------------------------------------------------------TTTTAGATGTGGTTTTTGTTTGTTTTTCTTTGTTCTGTGTTTTTTTTGGG-TGTTTTAGGTGTTTTG   66
```

trnR *nad4l*

1.23.3 nad4l-cox2 -3

nad4l *cox2*

```
R      GTTGTTTCTCGTTTTTGTGGTAGTGATAATGTTTTTTCTTTAAAATTGCTTCGTATATAGAGAAAAATGGCAACTTGGTTACAGTTAGGCTTTCAGGATGCATCTTCTCCTTTAATGGAGGAGCT-   125
nad4l  GTAGTTTCTCGAAGGTTTGGTACTGATAGAGTTTTTTCTTTGAAATTATTGCGAGTTTAG------------------------------------------------------------------   60
cox2   ---------------------------------------------------------------ATAATGGCAACTTGGCTGCAGTTTGGTTTTCAGGATGCTTCTTCTCCATTAATGGAGGAGTTG   63
```

nad4l *cox2*

1.24 NC_010971-NC_005961

Avg ovsize: 0

LCA: Typhlopoidea-superfamily

1.24.1 trnQ-trnM 5

```
                                 trnQ                                                    trnM
R      GGAGTTGAACCTAAACCAAGAAGCTCAAAACTTCTCGTACTACCAC-TA-TACTACCTCTTAGTAAAGTCAGCTAAATAAGCTCTCGGGCCCATACCCCGAAAATGTTGAACA-CAACCTT   118
trnQ   --ACTCGAACCCGCACCTGAAAGATCAAAACTTCCAGTACTACCTCATTGTACTACCCCTTACTAA-------------------------------------------------------    64
trnM   -------------------------------------------------------------AGTAGAGTCAGCTAAGCAAGCTATTGGGCCCATACCCCAAAAATGTTATACACCAACCTC    60
                                 trnQ
                                                                                         trnM
```

1.24.2 trnI-trnQ 2

```
                                 trnI                                                    trnQ
R      TGCCTGAGCCTTAAGGATTGCATTGATAGTGCAAATACAGAG--TTAACCCTCTCACCTCCTAAGAGT-CCAGGAGTTGAACCTAAACCAAGAAGCTCAAAACTTCTCGTACTACCAC-TA   117
trnI   TGCCTGAG--AAAAGGACTACCTTGATAAAGTAAAAACAGAGCATTGAACCTCTCACTTCCT-----------------------------------------------------------    60
trnQ   ------------------------------------------------------------CTAA-AGAAGCAGGACTCGAACCCGCACCTGAAAGATCAAAACTTCCAGTACTACCTCATT    60
                                 trnI
                                                                                         trnQ
```

1.24.3 trnW-trnA -8

```
                                 trnW                                                    trnA
R      --TT-TAGGT-TATATAAACCAGGAGCCTTCAAAGCTCCAAAAAAGACATTGGCTTATCTCCTGAGAGTTTGCAACACGTCACATCTCCTGCATGCAACACAGACACTTTAATTAAGCTAAA   118
trnW   GGTTATACACCCAT-TAAACCTGAGGCCTTCAAAGCCCCCAAAAA-ACAAT--TTTAGCTTCTG----------------------------------------------------------    60
trnA   ------------------------------------------------------------------------CAACTCATCACATCTCCTGCATGCAACACAGACACTTTAATTAAGC--CA    48
                                 trnW
                                                                                         trnA
```

1.25 NC_001922-NC_004448

Avg ovsize: 0
LCA: Alligatorinae-subfamily

1.25.1 trnG-nad3 2

```
                    trnG                                                 nad3
R     AAACAATACAAGTGACTTCCAATCACTAGACCCCCCAATTATCCCACCAGGGGAAAAGCAATTAACCTATTTATCATACTCACAATATCCTCAATCACCGTTTCAATCGTAGTCGCCCT-   119
trnG  ACAT--------------------------------------------------------------------------------------------------------------------     4
nad3  --ATAATACAAGTGACTTCCAATCACTAAACCCC-TAA-CACACAACAAGGGGAAAAGCAATCAACCTTCTTACCATATTCATAGTAACCTCCATCACCGCCGCAGCCGTAATCACTATA   116
      trnG                                                                                                 nad3
```

1.25.2 atp6-cox3 1

```
                    atp6                                                 cox3
R     ATAATCCAAGCATACGTCTTCGTCCTCTTATTATCCCTATATTTACAAGAAAATTCATAATGTCACACCAAACACACCTCTTCCATATAGTCAACCCAAGTCCCTGACCCCTCGCCGG-   118
atp6  ATAATCCAAGCCTACGTCTTCGTCCTCCTATTATCCCTATACCTTCAAGAAAACACGTAA-----------------------------------------------------------    60
cox3  -----------------------------------------------------------ATGTCACACCAAACACACTCCTTTCACATAGTCCACCCCAGCCCCTGACCCCTCGCCGGG    60
                    atp6                                                 cox3
```

1.25.3 cox3-trnG -4

```
R     GACACTTTGTAGACGTCGTCTGACTTTTCCTCTATATCTCGATCTACTGATGAGGATCGTGCTTTTCTAGTACAAACAATACAAGTGACTTCCAATCACTAGACCCCCCAATTATCCCA 119
cox3  GACATTTTGTAGATGTCGTCTGACTCTTCCTTTACATCTCAATCTACTGATGAGGTTCATGCTCTTCTAGTATAAATAATACAAGTGACTTCCAATCACTAAACCCC------------ 107
trnG  ---------------------------------------------------------------------------------------------------------------TGATTCTC 8
```

(R annotations: cox3, trnG; lower annotations: cox3, trnG)

1.26 NC_011569-NC_010268

Avg ovsize: -4

LCA: Aulorhynchidae-family

1.26.1 trnS1-trnL1 9

```
R      CTTGCTAGCAACGGATACTGCTAATCTCCGTAACTTTGGTTGGAACCCAATGCTCACTCAGCCCACCCACTTCTGTAGGATAACAGCTTATCCATTGGACTTAGGATCCAAAGACTCTTGGTGCAAA- 127
trnS1  CTCGCTAGCAACGAAAACTGCTAATTTTCGCAACTTTGGTTGAACCCCAAAGCTCACTC-GACCTCACAC---------------------------------------------------------- 69
trnL1  -------------------------------------------------------------CCCTCATACTTCTGTAGGATAACAGCGCATCCATTGGACTTAGGATCCAAAAACTCTTGGTGCAAAT 67
```

(R annotations: trnS1, trnL1; lower annotations: trnS1, trnL1)

1.26.2 nad4-trnH 3

```
                                nad4                                               trnH
R      ACCTCATACCACTTATTCTCCTTGTACTTAAGCCTGAACTAGTGTGAGGTTGAGCCACATGTAAATGTAGTTTAAGCAAAATATTAGATTGTGATTCTAAAGATAGAAGCTAA-AACCTT   119
nad4   ACCTAATTCCTCTACTTCTCTTGGTGTTTAAACCAGAACTAATTTGAGGTTGAACTACATGT----------------------------------------------------------    62
trnH   -----------------------------------------------------------TGTAGACGTAGTTTAACAAAAATATTAGATTGTGATTCTAAAGATAGGGGTTAATCTGCCC    61
                                nad4
                                                                                   trnH
```

1.26.3 trnH-trnS1 -24

```
                                trnH
R      GTTTAAGCAAAATATTAGATTGTGATTCTAAAGATAGAAGCTAAA-ACCTTCTCATTCACCCTTAAAATTTCACCCAAAACGTTTAGTGAGTGTAGTACA-ACACTAGATTTCATCTAAAAATAGGAG   126
trnH   -TTTAACAAAAATATTAGATTGTGATTCTAAAGATAGGGGTTAATCTGCCCCTCGTCCACCAT-AA--------------------------------------------------------------    64
trnS1  ------------------------------------------------------------------------------------------GGGTA-TAAATACACCAGGAAAAACCTGAAAATAGGGG    37
                                trnH

                                            trnS1
R      CTAAATCTCCTCACTCC-CAGAGAGAGGCTTGCTAGCAACGGATACTGCTAATCTCCGTAACTTTGGTTGGAACCCAAT-   204
trnH   --------------------------------------------------------------------------------    64
trnS1  TGTAATCTCCCCAACCCACCGAGAGAGGCTCGCTAGCAACGAAAACTGCTAATTTTCGCAACTTTGGTTGAACCCCAAAG   117
                                           trnS1
```

1.27 NC_010777-NC_008063

Avg ovsize: -4

LCA: Araneomorphae-suborder

1.27.1 trnI-trnQ 3

```
                                    trnI                                                  trnQ
R     AGTAAGGTGCCTGATTAAAGGGTTAATTTGATAGATTAAATAACGAACAGGTATATTTTA-CCAATTTAAAAATCAAATTTTTAAGTG--TTT-CACTTTGTTA   100
trnI  -TTATG-TGCCTGAATAAAGGGTTAATTTGATAGATTAAACAATGAA-A--TATAT------------------------------------------------    51
trnQ  -----------------------------------------------------TATTTAAATCTCTTAAAAAATCAAATTTTTTTGTGCATATACACTTT--AA    49
                                  trnI
                                                                                    trnQ
```

1.27.2 nad6-cob 3

```
                                    nad6                                                          cob
R     TCGCTATGTTAATTGTAGTATGAGTTTCTAATTA-TAATAAGGGCCCCGTTTGAACTAATTTTGAAAGTATTGATACGAAAAAAAAACGTAGTTCTAAGAGTTTTAAATGGGTCATT-G--G-T   119
nad6  GCAGTA-AT-AATTGTAGTA--ATTTGATTAAGAGGAATAAAGGAAGGGGCAGTACGGATTTAGAAA---------------------------------------------------------    63
cob   ----------------------------------------------------------------AAATTCTT--TACGTAAGAAAAGTGTA-TT-T-AGAGTAATAAATAATATATTAGTAGAT    55
                                     nad6
                                                                                                   cob
```

1.27.3 rrnS-trnI -18

```
                                           rrnS                                                          trnI
R     -----TAA-TAAATAGA-TCTTTATTTAATTAATGATTAATTTTA-A-CTGAATTAATTTATTTTCAATAGTAAGGTGCCTGATTAAAGGGTTAATTTGATAGATTAAATAACG-A----A-CAG   110
rrnS  ATTTCTAAGTTAAGTGAATTTTCATTTAAAAAAGTAT-A-TTTAAGAGCTGTACTAA--------------------------------------------------------------------    55
trnI  ---------------------------------------------------------------------------GTGCCTGAATAAAGGGTTAATTTGATAGATTAAACAATGAAATATATCAA    50
                                  rrnS
                                                                                                    trnI
```

1.28 NC_005961-NC_010971

Avg ovsize: -5
LCA: Typhlopoidea-superfamily

1.28.1 trnW-trnQ 2

```
                             trnW                                                      trnQ
R     GGTTATACACCCAT-TAAACCTGAGGCCTTCAAAGCCCCCAAAAAACAATTTTAGCTTCTGCTACACTAAAGAAGC-AGGACTCGAACCCGCACCTGAAAGATCAAAACTTCCAGTACTACCTC-AT   124
trnW  --TT-TAG-GTTATATAAACCAGGAGCCTTCAAAGCTCC-AAAAAAGACATTG-GCTTAT-CT-C-CT-----------------------------------------------------------    59
trnQ  ------------------------------------------------------------------CTAA-GAGTCCAGGAGTTGAACCTAAACCAAGAAGCTCAAAACTTCTCGTACTACCACTAT    60
                                  trnW
                                                                                   trnQ
```

1.28.2 trnI-trnM -1

```
                              trnI                                                   trnM
R     TGCCTGAGAA--AAGGACTACCTTGATAAAGTAAAAACAGAGCATTGAACCTCTCACTTCCTAGTAGAGTCAGCTAAGCAAGCTATTGGGCCCATACCCCAAAAATGTTATACACCAACCT--   119
trnI  TGCCTGAGCCTTAAGGATTGCATTGATAGTGCAAATACAGAG--TTAACCCTCTCACCTCCT-------------------------------------------------------------    60
trnM  ---------------------------------------------------------------GTAAAGTCAGCTAAATAAGCTCTCGGGCCCATACCCCGAAAATGTTGAACA-CAACCTTT    59
                              trnI
                                                                                     trnM
```

1.28.3 trnQ-trnA -16

```
                              trnQ                                                    trnA
R     --ACTCGAACCCGCACCTGAAAGATCAAAACTTCCAGTACTACCTCATTGTACTACCCCTTACTAATAGAACTCGCGACAACTCATCACATCTCCTGCATGCAACACAGACACTTTAATTAAGC----   122
trnQ  GGAGTTGAACCTAAACCAAGAAGCTCAAAACTTCTCGTACTACCAC-TA-TACTACCTCTTA------------------------------------------------------------------    60
trnA  ------------------------------------------------------------------------------CAACACGTCACATCTCCTGCATGCAACACAGACACTTTAATTAAGCTAAA    50
                             trnQ
                                                                                                        trnA
```

```
      trnA
R      C   123
trnQ   -    60
trnA   A    51
      trnA
```

1.29 NC_022670-NC_012689

Avg ovsize: -6

LCA: Neoptera-subclass

1.29.1 cob-trnS2 5

```
                                cob                                                    trnS2
R      TTT-TATTTTATTC-ATTATCATGCCTATCACCTCAAAAACATGAGATAAAATACTTGATTAAGTTAATTAAGCTTTAGA-AAGCGAGTACCTTGAAAGTACTAGA-AGATAGTTAAATTCT-A   119
cob    TTCGCATACT-TTCTAATATTAGAACTACCA-TTAAAATA-TTGAGATAAACTATTATATTAATTTA---------------------------------------------------------    64
trnS2  --------------------------------------------------------------AGTTAATTA-GTTAAATATAAACTTATATCTTGAAAATATAAAATAAATAA-TAATTTATTT    60
                                cob
                                                                                    trnS2
```

1.29.2 trnM-nad2 4

```
                                 trnM                                                     nad2
R     -GCTAATATTAAGCTTATAGGTTCATACCCTATCAATAGAAGTA-AAACCTTCTTCTAATTAATAAATATTAAAACAATAAT-AACAAG-ACTAACTTTAAGAACCCTTATAGTAATA-TCATC   119
trnM  AGCTAAT-T-AAGCTATTAGACTCATACTCTAACTATAGAAATATCAATTTTCTTCTTTTTA---------------------------------------------------------------    60
nad2  ----------------------------------------------------------ATTAATAAATTTTATTACAA-ATTCAACTTGTCCTGA-TTTAATA---C-AACAGTAATTTTCCCT    60
                                 trnM
                                                                                    nad2
```

1.29.3 trnI-trnM -29

```
                               trnI                                                           trnM
R     AGATGCCTGAATAAAGGATTACTTTGATAGAGTAAATCATGCACAATTATGCTCTTACTATAAATAATTAGATAAGCTAATATTAAGCTTATAGGTTCATACCCTATCAATAGAAGTAA-AAC-CT-   124
trnI  ---TGCCTGACAAAAGGATTATTTCGATAAAATAAATCAT---------------------------------------------------------------------------------------    37
trnM  ---------------------------------------------------------------------AGATAAGCTAAT-T-AAGCTATTAGACTCATACTCTAACTATAGAAATATCAATTTTC    56
                      trnI
                                                                                                trnM
```

1.30 NC_009851-NC_006340

Avg ovsize: -10

LCA: Teleostomi-superclass

1.30.1 trnA-trnN 30

```
                                   trnA                                                          trnN
R     CGGGACATTACCCCACATCTCCTGCATGCAAAACAGACACTTTAATTAAGCTAAAGCCTTTCTAGATAGGCAGGCCTC-GATCCTGCAA-ACTCTTAGTTAACAGCTAAGCGCTCA-A-ACCAG  120
trnA  CGAGACTCTACCCCACATCTTCTGAATGCAACCCAGACACTTTAATTAAGCTAAAGCCTT-C-ACAAAC-TACACCTCATATTCTTGTA-A-T-TTAATAAACAACTAA---------------  103
trnN  -------------------------------------------------------------------------------TATCCTACTACAAT-TTAGTTAACAGCTAAACAC-CAGATTTCAG   43
                                   trnA                                                                        trnN
```

1.30.2 trnY-trnW 0

1.30.3 nad2-trnY -62

```
                                    nad2                                  trnY
R     -CATGGCCACCATCTCACTGTTACCCCTCGCCCCCGCAGCAATTGCACTACTGACCCTCTAAGAGACTTAGGCTAAC   76
nad2  ACA--------------------------------------------------------------------------    3
trnY  -----------------------------------------------------------------ATTTCGGCC-AC   11
      nad2                                                                  trnY
```

1.31 NC_016755-NC_006286

Avg ovsize: -11

LCA: Laterata-suborder

1.31.1 trnE-cob 14

```
                    trnE                                            cob
R     -CTGGC--TTATATCCAAAACCTGCGACCCGAAAAACCGCTGTTGTTATTCAACTATTAA-AGCAATGAC--CTC-CAACATACGAAAACAACACCCAATCTTAAAAATCGTAAATAGCTCCTTCA-T   120
trnE  GCCGGCAATTCTA-CCGGA-CCTGCGGTCTGAAAAACCGCCGTTGTG-TTCAACTACAAAAACC----------------------------------------------------------------   61
cob   --------------------------------------------------CAACTACAAGCACCAATAATGGCCCACATCACACGCAAGCATCATCCAATCTTTAAAATGGTCAACAGCACATTTATT   78
                    trnE                                            cob
```

1.31.2 nad5-nad6 -22

```
                    nad5                                            nad6
R     TT-CTATTTATCAATTTTCTTAATCACT-TCCCTATGTTTTCTCGTCCTCATGTGAACCTAACCGCACAAAGACTTCCCCGCACCATCCCCCGAGTTAACTCTAACACAACAAATA-   114
nad5  GGACGAAT-AA-AATCTTATTTATCATTATTCCTA----------------------------------------------------------------------------------   33
nad6  ---------------------------------------------------------CCTAAATACCCGAATAGCGCCCCGTTCCCCACCTAAAGATAACCATAATACCACAAAAAG   60
                    nad5                                            nad6
```

1.31.3 nad6-trnE -25

```
                    nad6                                            trnE
R     CGCAATACCAACCAAACCTGCCACAAA-ACACACCACAAGAAAACTTACAAA-ATATATCATCACTTCAATCTGGCTTATATCCAAAACCTGCGACCCGAAAAACCGCTGTTGTTATTCAA---   119
nad6  CGCAACACCAACAATAGCTATCAAAAATGCCCACCCCAA-AAGA-TAAGTAATATATATCAT--------------------------------------------------------------   60
trnE  ---------------------------------------------------------------------------------------ACCTGCGGTCTGAAAAACCGCCGTTGT-GTTCAACTA   36
                    nad6                                            trnE
```

1.32 NC_008327-NC_023228

Avg ovsize: -11
LCA: Soleoidei-suborder

1.32.1 trnI-trnQ 3

trnI *trnQ*

```
R     TGAAGCTAAAGGGCCACTTTGATAGAGTGAACCATGGGGGTTAAAATCCCCCTAACTCCTTAGAAAGAAGGGAATCGACCCCTTACTGAAGAGATCAAAACTCTTCGTGCTCCCCTTA-   118
trnI  TCTGAATAAAGAGTCACTTTGATAGAGTGAAGCACGTAGATGAAAACCCTACTAGCTCCTTA-AAA-----------------------------------------------------    65
trnQ  ---------------------------------------------------------------AAAGGAGG-ATTCGAACCTCAACTTAAGAGATCAAAACTCTTTGTGCACTCCATAA    55
```

trnI

trnQ

1.32.2 trnQ-trnM 1

trnQ *trnM*

```
R     GGAATCGACCCCTTACTGAAGAGATCAAAACTCTTCGTGCTCCCCTTA-CACCACTTCCTAGTGA-GGTCAGCTAAT-CAAGCTGTAGGGCCCATACCCCTAAAATGTAGGTTAAACCCCT   118
trnQ  G-ATTCGAACCTCAACTTAAGAGATCAAAACTCTTTGTGCACTCCATAACACTATTTCCTA------------------------------------------------------------    60
trnM  ------------------------------------------------------------AGTTACGGTAAGCTAAAACAAGCTATTGGGCCCATACCCCAGCCATATAGGTTAAAACCCT    61
```

trnQ

trnM

1.32.3 nad1-trnI -37

```
                                    nad1                                                         trnI
R     ATAGTTATTTGGAAT-CTAGCTATTCCCACTGCATTCGCCGGCTTACCCCCGCAGCTCTAACAAGGAGCTATGCCTGAAGCTAAAGGGCCACTTTGATAGAGTGAACCATGGGGGTTAAAATC---   122
nad1  TTCGTGATA-GTAACCCTAATAATTC----------------------------------------------------------------------------------------------------    25
trnI  ---------------------------------------------------------------AGGAGCTATGTCTGAA--TAAAGAGTCACTTTGATAGAGTGAAGCACGTAGATGAAAACCCTA    61
                 nad1
                                                                                                trnI
```

1.33 NC_022477-NC_006355

Avg ovsize: -11

LCA: Perciformes-order

1.33.1 trnM-nad2 50

```
                                    trnM                                                          nad2
R     GCTAA-ATAAGCTTTCGGGCCCATCC-CCCGAACATGTCGGCCAACAATCCTCCTTTACTAA-TGAACCCCCTTATCCTGGCTCTA-CTA-CTATTTAGCCTCGGCCTAGGCACCATCCTCAC-   119
trnM  GCTAATAGAAGCTATTGGGCCCATAC-CCCAAAAATGTTGACTAATACTCAG-CCTTTGTTAC-TTTCCCC-----------------------------------------------------    69
nad2  ----------------------ATACACCCAAACAAGTTGATTAACT-TCAA-CGATTGCTTCATGAGCCCCTATATTCT--CTCGATCTTTCTTTCGGACTCGGCCTAGGAACCACAATTACA    99
                                    trnM
                                                                                                nad2
```

1.33.2 trnQ-trnI -36

```
                                       trnQ
R     GGATTCGAACCCAACCTAAAGAGATCAAAACTCTCAGTGCTTCCTCTACACCACTTCCTAGTAAAGTCAGCTAAAAAAGCTTTCAGGCCCATAC-CCTGAACATGTTGGTTAAAACCCTTCCCTTACT   127
trnQ  GGGCTCGAACCCTTCCTGAAGAGATCAAAACTCTTGGTGCTTCCACTACACCACTTCCTAGCAAAA-CAAGGAAACAA--TTCCATTGGAATACACCCAAACAAGTTGATTAA---------------   110
trnI  --------------------------------------------------------------------------------------------------------------------------------     0
                                       trnQ

                                                     trnI
R     ACACCTCTATCCTAAACCCCTGGAGCCGTGCCTGAACCAAAGGATCACTTTGATAGAGTGAATCACGAGGGTTAAAA-CCC   207
trnQ  ---------------------------------------------------------------------------------   110
trnI  ---------------------GGAGCTGTGCCTGAATCAAAGGGTCACTTTGATAGAGTGAAGCATGGGGGTTAAAATCCT    60
                                                     trnI
```

1.33.3 nad1-trnQ -49

```
                                       nad1                                                                                 trnQ
R     TTAATAGTATGACACCTCGCCCTCCCAATCGCATTCTCAGGCCTTCCACCCCAAATATAATATAACAAACCACACAAACAAAAACCAACCCCACCCACCCTTTAAACCTATTAGAAAGAAGGGATTCG   128
nad1  TTTATAATCTGACATCTGGCACTACTAATTGCATTCGCCGGACTTCCCCCACACCTTTAACA------------------------------------------------------------------    62
trnQ  ---------------------------------------------------------------------------------------------------------------TAGAAAAAAGGGGCTCG    17
                                       nad1
                                                                                                                            trnQ

                           trnQ
R     AACCCAACCTAAAGAGATCAAAACTCTCAGTGCTTCCTCTA-   169
nad1  ------------------------------------------    62
trnQ  AACCCTTCCTGAAGAGATCAAAACTCTTGGTGCTTCCACTAC    59
                           trnQ
```

1.34 NC_009851-NC_003159

Avg ovsize: -13
LCA: Neoteleostei-subclass

1.34.1 trnY-trnW 0

1.34.2 trnN-trnC -17

```
R     CCTCGATCCTGCAAACTCTTAGTTAACAGCTAAGCGCTCAAACCAGCGAGCATCCATCTACCTTTCCCCCGCCTGATAAAACTAGGAGGCGGGGAAAGCCCCGGCAAGCAGTTAGCTTGCGACTTCA   128
trnN  CCTCGATCCCCCAAACTCTTAGTTAACAGCTAAGCGCCCCAACCAGCGAGCATCCATCTAACTGACC-------------------------------------------------------------    67
trnC  ------------------------------------------------------------------------------------GGCGTCGACGGAAAGCCCCGGCAGGCTGGAAACCTGCTTCTTCA    44
```

trnN (upper brace over R), trnC (upper brace over R); trnN (lower brace under trnN), trnC (lower brace under trnC)

```
R     GATTTGCAATCTGATATGTTTACACCT--   155
trnN  -----------------------------    67
trnC  GATTTGCAATCTGACGTGTC-ACACCTCA    72
```

trnC (upper brace over R); trnC (lower brace under trnC)

1.34.3 nad2-trnY -22

nad2 trnY

```
R     -C-ATGGCCACCATCTCACTGTTAC-CCCTCGCCCCCGCAGCAATTGCACTACTGACCCTCTAAGAGACTTAG-GCTAAC    76
nad2  TCTATCGCTGCCCT-TG-G-GTTGCTCCCTGTAACCC-CAGCTAT-----------------------------------    41
trnY  -------------------------------------------------------------------ACTTAAATCTCAG    13
```

nad2 trnY

1.35 NC_012762-NC_008683

Avg ovsize: -19

LCA: Teleostomi-superclass

1.35.1 trnI-nad1 0

1.35.2 rrnS-trnV -7

rrnS trnV

```
R     ATTACTAGAGGAGATAAGTCGTAACAAGGTAAGCATACTGGAAAGTGTGCTTGGATCACCAAAGTGTAGCTTAACAACTAAAGCATCCGGCTTACACCCGGAAGATTTCAGCCCATGA-    118
rrnS  -G-ACAAGGGGAGGCAAGTCGTAACATGGTAAGTGTACCGGAAGGTGCACTTGGATTA-------------------------------------------------------------     56
trnV  -----------------------------------------------------------------GTGGCTGAGTTAGTCAAGCATCTCACTTACACCGAGAAGACATCCATGCAAGTT     54
```

rrnS trnV

1.35.3 trnL2-trnI -50

```
R      CCCGGTAA-TTGCATAAAACTTAA-AACTTTACCACCAGAGGTTCAACTCCTCTTCTTAACACATGTTTGTTCTAAACCTCCTACTACTCATCATTCCCATTATCCTGGCCATAGCC--TTC 118
trnL2  AT-GGTAAATTGCGAAAGGCCTAAGCCCTTTAA-ACCAGAGGTTCAAATCCTCTTC------------------------------------------------------------------ 54
trnI   ----------------------------------------------------------------------------------------------------------TGTCCGAAGCCCGATC 16
```

1.36 NC_010268-NC_010199

Avg ovsize: -20

LCA: Percomorpha-order

1.36.1 trnL1-nad5 39

```
R      AACAGCGCATCCATTGGACTTAGGATCCAAAAACTCTTGGTGCAAATCCAAGTGGAAGTAATGATAAAAG-ACCC-T-ACTCTAATCATACACTCT-GGATTCATCATAGTATTTACCATCTT 119
trnL1  AATAGCCCTTCCATTGGTCTTAGGAACCAAAAACTCTTGGTGCAAATCCAAGTAGCAGC---------------------------------------------------------------- 59
nad5   --------------------TAGTCACCAATGCC-CTTGGTTCAAATCCAAGTAGTAGCCATGCCCCCCTTACACATTACCTTAA-CATCCACCCTAACAATAACCCTCCTGCT-CTTAGC-C 99
```

1.36.2 trnH-trnL1 -38

```
                                             trnH                                                                                trnL1
R      -TTTAACAAAAATATTAGATTGTGATTCTAAAGATAGGGGTTAATCTGCCCCTCGTCCACCATAACTCCACCTATACCCTAAATTTAAATTTTAACCCTCATACTTCTGTAGGATAACAGCGCATCCA   127
trnH   GTTTAATAAAAACATTAGATTGTGATTCTAAAGACAGAAGTTAAAAC-CTCCTTATCCACCCCAA---------------------------------------------------------------    64
trnL1  -------------------------------------------------------------------------------------------------------CTCCTAAAGGATAATAGCCCTTCCA    25
                                             trnH                                                                                trnL1

                   trnL1
R      TTGGACTTAGGATCCAAAAACTCTTGGTGCAAA-   160
trnH   ----------------------------------    64
trnL1  TTGGTCTTAGGAACCAAAAACTCTTGGTGCAAAT    59
                   trnL1
```

1.36.3 trnS1-trnH -62

```
                                  trnS1                                                                                          trnH
R      CTCGCTAGCAACGAAAACTGCTAATTTTCGCAACTTTGGTTGAACCCCAAAGCTCACTCGACCTCACACACACATTTCAATTTAATATCCTCATACTAAATAAACTAGACCCTAAAAATAAGTGTAGA   128
trnS1  CCTGCTGGCAACGAAGACTGCTAATCCTCATCCCCTCGGTTGAAATCCGAAGCTCACTCAA-------------------------------------------------------------------    61
trnH   -----------------------------------------------------------------------------------------------------------------------------GTAGA     5
                                  trnS1                                                                                          trnH
```

```
R      CGTAGTTTAACAAAAATATTAGATTGTGATTCTAAAGATAGGGGTT-AATCTGCC  182
trnS1  -------------------------------------------------------  61
trnH   TATAGTTTAATAAAAACATTAGATTGTGATTCTAAAGACAGAAGTTAAAACCTCC  60
```

1.37 NC_008063-NC_010777

Avg ovsize: -23

LCA: Araneomorphae-suborder

1.37.1 trnI-cob -5

```
R     -TTATG-TGCCTGAATAAAGGGTTAATTTGATAGATTAAACAATGAAATATATCAAATTCTT--TACGTAAGAAAAGTGTA-TT-TA-GAGTAATAAATAATATATTAGTAGA  106
trnI  AGTAAGGTGCCTGATTAAAGGGTTAATTTGATAGATTAAATAACGAA------------------------------------------------------------------  47
cob   ----------------------------------------------------TGAAAGTATTGATACGAAAAAAAACGTAGTTCTAAGAGTTTTAAATGGGTCATTGGTA--  59
```

1.37.2 rrnS-trnQ -16

```
R     ATTTCTAAGTTAAGTGAATTTTCATTTAAAAAA-GTATA-TTTAAGAGCTGTACTAAACTCAACTTGTTAGATTATTTAAATCTCTTAAAAAATCAAATTTTTTTGTGCATATACACT-TTA-   119
rrnS  ---T--AA-TAAATAGA-TCTTTATTTAATTAATGATTAATTTTA-A-CTGAATTAA------------------------------------------------------------------    48
trnQ  -------------------------------------------------------------------------TATTTT-ACCAATTTAAAAATCAAATTTTTAAGTGTTTC-ACTTTGTTAT    48
```

rrnS (R, positions 1–57); trnQ (R, positions 60–123); rrnS (rrnS); trnQ (trnQ)

1.37.3 nad6-trnI -49

```
R     GCAGTA-AT-AATTGTAGTAATTTGATTAAGAGGAATAAAGGAAGGGGCAGTACGGATTTAGAAATTATGTGCCTGAATAAAGGGTTAATTTGATAGATTAAACAATGAAATATATCAA   117
nad6  TCGCTATGTTAATTGTAGTA---------------------------------------------------------------------------------------------------    20
trnI  ---------------------------------------------------------------------GTGCCTGATTAAAGGGTTAATTTGATAGATTAAATAACG--A-A---CAG    44
```

nad6 (R); trnI (R); nad6 (nad6); trnI (trnI)

1.38 NC_007781-NC_008797

Avg ovsize: -23

LCA: Neogastropoda-infraorder

1.38.1 trnW-trnQ 3

```
                        trnW                                                  trnQ
R      -TAAAGATTTTTCTTTATATTAAGCTTTGAAGGCTCATAGTTTGTATTAACTTAAAACCTTACCAGGAGGAACTGAACCTCTCCTAAGAAATCAAAATTCTTTGTGCCCTTACACCG-   116
trnW   CTAAAGAATCTTCTTTATTTCAGGCTTTGAAGGCCTGCAGTTT-TAATAACTTAAGACCTT---------------------------------------------------------    60
trnQ   ----------------------------------------------------------CTTATCAGGAGGATGTGAACCTCTCTTAAGAAATCAAAATTCTTAGTGCCCTAACACCGC    60
                        trnW
                                                                              trnQ
```

1.38.2 trnL1-nad1 1

```
                        trnL1                                                        nad1
R      GCAGATGAAG--TGC-ATTAGGTTTAAGCCCTAAATATAAAGATGAAATTTCTTTTCTTTATA-ATGTATGTTTCTATCATYTCAACAGTATTTACCTATATCTGTATTTTATTGGCAGTCGC-   119
trnL1  -CA-AAGAATTATGCGATT-GATTTAGGATCAATAGACATAGGTTAAAATCCTTTATTTGATAGA-----------------------------------------------------------    62
nad1   ----------------------------------------------------------------ATGTATCTAAGAATTATTTCCTGTTTGTTTACTTATGTTTGTATTTTACTAGCGGTCGCT    60
                        trnL1
                                                                                     nad1
```

1.38.3 trnL2-trnL1 -22

```
                        trnL2                                                        trnL1
R      GCAAAATTAA-TGCAATTGACTTAGGATCAATAGATATAGGTGAAATTCCTTTATTTGATAATTATAAAGGTGGCAGATGAAGTGC-ATTAGGTTTAAGCCCTAAATATAAAGATGAAATTTCT   122
trnL2  GCAG-ATTAAGTGCATTAGGTTTAAGCCCTAAAGATAAAGATGAAAATTCTTTCCTTTATA---------------------------------------------------------------    60
trnL1  -----------------------------------------------------------------------------------TGCGATT-GATTTAGGATCAATAGACATAGGTTAAAATCCT    40
                        trnL2
                                                                                                 trnL1
```

1.38.4 rrnL-trnL2 -76

```
                                      rrnL                                                                         trnL2
R      GCCAGGTCAGTTTCTATC-TTTAAT-TATATAGATAAGTTTAGTACGAAAGGACCAATTTATCATAAAACGATATTACTTACTGATTAAATTTAATTAACAATTAAATGATCAAATTAGCAAAATTAA   126
rrnL   GAAAGGACCGGC-GTAACATATAATATATTTTTATTAGATCA--------------------------------------------------------------------------------------    41
trnL2  ----------------------------------------------------------------------------------------------------------------------GC-AGATTAA     9
                         rrnL                                                                                                   trnL2
```

```
                         trnL2
R      -TGCAATTGACTTAGGATCAATAGATATAGGTGAA-ATTCCT   166
rrnL   ------------------------------------------    41
trnL2  GTGCATTAGGTTTAAGCCCTAAAGATAAAGATGAAAATTCTT    51
                         trnL2
```

1.39 NC_012762-NC_014174

Avg ovsize: -24

LCA: Amniota-clade

1.39.1 trnI-nad1 0

1.39.2 trnQ-trnM -21

```
                               trnQ                                                        trnM
R     G-ACTTGAACCCAC-TCTTAAGAACTCAAAATTCTCCGTGC-TACCTATCACACCCTATCCTACAAACAGTAAGGTCAGCTAAATAAGCTATCGGGCCCATACCCCGAAAATGTTGGTTTATACCCT-  124
trnQ  GGAGTTGAACCAACATCAAAA-AATTCAAAATTTT--ATGCATTTCTATTATAC--------------------------------------------------------------------------   51
trnM  ---------------------------------------------------------------------------TCTGCTACACAAGCAATTGGGCCCATACCCCAAAAACGATAACATATATCTTC   53
                           trnQ                                                                        trnM
```

1.39.3 trnL2-trnI -51

```
                               trnL2                                               trnI
R      -CCCGGTAATTGCATAAAACTTAAAAC-TTTACCACCAGAGGTTCAACTCCTCTTCTTAACACATGTTTGTTCTAAACCTCCTACTACTCATCATTCCCATTATCCTGGCCATAGCCTTC-  118
trnL2  GCTCGGTTATTGCAAGAGGCCTAAAACCTTTA--ACCAGAAGTTCAAATCTTCTCCACAACAAATAT------------------------------------------------------   65
trnI   ----------------------------------------------------------------------------------------------------------------------TCT    3
                               trnL2
                                                                                                                          trnI
```

1.40 NC_007689-NC_007690

Avg ovsize: -24

LCA: Crinozoa-subphylum

1.40.1 trnR-cox2 -2

```
                                  trnR                                                                   cox2
R      GTTTAAGTAAAACTTTTGGTTTCGGCCCGTTTATTTTTGGTTATAGTCCAAAC-CTTTCTTTTATAATGGCAACTTGGCTGCAGTTTGGTTTTCAGGATGCTTCTTCTCCATTAATGGAGGAG-TT   124
trnR   GTTTAATAAAAATTTTTGGTTTCGGCCCTTATGCTTTTGGTTAAAGTCCAAACTCTTT-TT-----------------------------------------------------------------    60
cox2   ---------------------------------------------------------------AAAATGGCAACTTGGTTACAGTTAGGCTTTCAGGATGCATCTTCTCCTTTAATGGAGGAGCTT    63
                                  trnR                                                                   cox2
```

1.40.2 atp6-nad4l -4

```
                                  atp6                                                                         nad4l
R      TGTATACAGGCTTATGTTTTTACAGCTTTGGTTCATTTTTATTTACAAGAGAAAT-TTTA-GT-TTTTTAGATGTGGTTTTTGTTTGTTTTTCTTTGTTCTGTGTTTTTTTT-GGGTGTTTTAGGTGT   124
atp6   TGTATTCAAGCTTATGTTTTTACTACATTAATTCATTTTTATTTACAGGA-AAATCTTTAAAAATTTTTAAA--------------------------------------------------------    71
nad4l  ----------------------------------------------------------------------------GTTTTTTTTATTTTTTATGTGTTTTATTTTTTTCTTAGGTTGTGTT-GGTGT    51
                                  atp6                                                                         nad4l
```

```
       nad4l
R      TTT-   127
atp6   ----    71
nad4l  AATA    55
       nad4l
```

1.40.3 nad4l-cox3 -67

```
                                      nad4l                                                                                                    cox3
R      GTAGTTTCTCGAAGGTTTGGTACTGATAGAGTTTTTTCTTTGAAATTATTGCGAGTTTAGGTTTCTGTCTGGGGGGTGTTTGTTTTGTTATTCTTTTGTATGAGTTGTTTTTGGGTGGGTGTAATTTA   128
nad4l  GTTGTTTCTCGTTTTTGTGGTAGTGATAATGTTTTTTCTTTAAAATTGCTTCGTATATAG--------------------------------------------------------------------   60
cox3   -------------------------------------------------------------------------------------------------------------------------------A   1
                                      nad4l                                                                                                    cox3
```

```
                                      cox3
R      ATGAGTTATCAACATTCTTATCATTTGGTTGAGCAGAGTCCTTGGCCTATAATGGCTTC-   187
nad4l  ------------------------------------------------------------   60
cox3   ATGAATCATCAACATCCATATCATTTGGTAGATCAAAGTCCATGACCTTTGATAGCTTCT   61
                                      cox3
```

1.41 NC_002735-NC_022670

Avg ovsize: -24

LCA: Atelocerata-superclass

1.41.1 trnS2-trnM -10

```
                                        trnS2                                                                                 trnM
R      --CTAAATGTTAAGTAAATATCTTGAAAATATTATAAAGA-AGTTTGATTCT-TCTAGAAATTTAGCTTATCTTTTATATATAACCCCCAGTATATATAA-AAGATAAGCTAATA--A-GCTACTGGG   120
trnS2  AGCTTTA-GA-AAGCGAGTACCTTGAAAGTACTA-GAAGATAGTTAAATTCTATC-ATTAACTTACCATA-CACTTATTT-TAA--------------------------------------------   78
trnM   ----------------------------------------------------------------------------------------------AAATAATTAGATAAGCTAATATTAAGCTTATAGG   34
                                       trnS2                                                                              trnM
```

```
                               trnM
R       CCCATACCCCTTCTATGTAAGTAAATCTTACT     152
trnS2   --------------------------------      78
trnM    TTCATACCCTATCAATAGAAGTAAAACCT--T      64
                               trnM
```

1.41.2 trnI-trnS2 -27

```
                               trnI                                                 trnS2
R       TAG-TGCCTGATAAAAAGGAGTATCCTGATAGGGTAAATTATGCACACT-ATGC-CTTGTCTTCTGAATTTCTTAACTAAATGTTAAGTAAATATCTTGAAAATATTATAAAGA-AGTTTGATTC--T     122
trnI    -AGATGCCTGA-ATAAAGGATTACTTTGATAGAGTAAATCATGCACAATTATGCTCTT----------------------------------------------------------------------      56
trnS2   -------------------------------------------------------------------------------------AAGCGAGTACCTTGAAAGTACTAGAA-GATAGTTAAATTCTAT      42
                               trnI
                                                                                                 trnS2
```

1.41.3 cob-nad1 -36

```
                               cob                                                        nad1
R       TTTATATATT-TT-ATTTTAAACCCAATAATTTCCTATCTTTGAGATTATA-ATTTAAATTAATAACACTTTAAAAATTTCAACAGTTAAACATAACTCCAACAACAAAGTATA-A-AA-T-AAGTAT     121
cob     TTT-TATTTTATTCATTATCATGCCTATCACCTCAAAAACATGAGATAAAATACTTG-ATTA------------------------------------------------------------------      60
nad1    --------------------------------------------------------------------------------------------------CCAACAATAAATAATAGACAATTCAAAGAA      30
                               cob
                                                                                                              nad1
```

```
         nad1
R      ATTTA   126
cob    -----    60
nad1   ACAGG    35
         nad1
```

1.42 NC_001922-NC_007012

Avg ovsize: -25

LCA: Teleostomi-superclass

1.42.1 trnH-trnL1 -13

```
                              trnH                                                                    trnL1
R      AGTTTAAACAAAACATTAGAATGTGAACCTAAAAATAGGAGACTACTTCCCCTTACTCACCTTTTCCCACTTTCATAGGATAACTAGGACATCCCCTGGCCTTAGGAGCC-AATAATCTTGGTGCAAC   127
trnH   -GTTTAAAAAAAACATTAGATTGTGATTCTAAAAACAAAAGTTAAAATCTTTTTATTTACC-------------------------------------------------------------------    60
trnL1  --------------------------------------------------------------------------AAAGGATAAC-AGCTCATCCATCGGTCTTAGGAACCGAAAACCCTTGGTGCAAC    53
                              trnH
                                                                                                     trnL1
```

```
R      -   127
trnH   -    60
trnL1  T    54
     trnL1
```

1.42.2 trnS1-trnH -14

```
                                      trnS1                                                                     trnH
R      CCG-CGCAGGAA---CTGCTAATTCCTAGCCCCCAGGACTAACAG-CC-TGGCCCTCTCGTCGTGGGCTCGGTAAGTATAGTTTAAACAAAACATTAGAATGTGAACCTAAAAATAGGAGACTACTTC   122
trnS1  CGGAAGCACTAAGGACTGCTAATCCTTAAGTCCCATGGTTCA-AGTCCATGGCTCACT----------------------------------------------------------------------    57
trnH   ------------------------------------------------------------------------TAAATATAGTTTAAAAAAAACATTAGATTGTGATTCTAAAAACAAAAGTTAAAATC    56
                                 trnS1
                                                                                                                trnH
```

```
       trnH
R      CC-   124
trnS1  ---    57
trnH   TTT    59
       trnH
```

1.42.3 nad4-trnS1 -49

```
                                     nad4                                                                                trnS1
R      TTACACACACTACCA-TCAATTATCCTCATCA-CAAACCCACGACTTATTTACCATGACTAACACCACCCACTCAATATTTAACCGAGAGAGATCCGCGCAGGAACTGCTAATTCCTAGCCCCCAGGA   126
nad4   --ACCTA-A-TCCCTGTC-CTTCTCCTAGTCCTTAAACCCGA-ACTTATAT-----------------------------------------------------------------------------    45
trnS1  ----------------------------------------------------------------------------------------------------AGGA-CTGCTAATCCTTAAGTCCCATGG    27
                                nad4
                                                                                                                    trnS1
```

```
                trnS1
R       CTAACAG-CC-TGGCCCT   142
nad4    ------------------    45
trnS1   TTCA-AGTCCATGG-C--    41
                trnS1
```

1.43 NC_009851-NC_013136

Avg ovsize: -25
LCA: Acanthuroidei-suborder

1.43.1 trnY-trnW 0

1.43.2 nad2-trnY -5

```
                                  nad2                                       trnY
R      CATGGCCACCATCTCACTGTTACCCCTCGCCCCCGCAGCAATTGCACTACTGACCCTCTAAGAGACTTAGGC-TAAC   76
nad2   TACTGCCTCCATTACCCTCCTACCACTCACCCCTACTCTTACCGCCCTACCAACCCTCTA-----------------   60
trnY   -----------------------------------------------------------------CTTAAACCTCAG   12
                                  nad2
                                                                            trnY
```

1.43.3 trnC-cox1 -72

```
trnC
R     CGGCAAGCAGTTAGCTTGCGACTTCAGATTTGCAATCTGATATGTTT-ACACCTCAAGGCTTGGTAAGAAGAGGACTCGAACCTCTGTCTATGGGGCTACAATCCACCGCTTAAAAACTCAGCCATCC   127
trnC  -GGCAGGCAATTAGCCTACTACTTAAGATTTGCAATCTAATATGTTAAACACCTCGGAGCT-------------------------------------------------------------------    60
cox1  --------------------------------------------------------------------------------------------------------------------------------     0
trnC
```

```
cox1
R     TACCTGTGGCAATCACACGTTGATTCTTCTCGACTAATCACAAAGACATCGGCACCCTCTATCT-   191
trnC  -----------------------------------------------------------------    60
cox1  -----GTGGCAATCACACGTTGATTTTTCTCGACTAATCACAAAGACATCGGCACCCTCTATCTA    60
cox1
```

1.44 NC_008975-NC_007178

Avg ovsize: -26

LCA: Old World tree frogs-family

1.44.1 trnT-trnP 21

```
trnT                                                        trnP
R     CTTAAC-CTAAAGCATTGGTCTTGTAAGCCAAAGATTGTAAACTAAACCTTACCCAGAGCTTCAAGAC-AAAAGGAATTTAACCTTC-ATCTCGACCCCCAAAGCCGGCATTCTATTTAAA-   119
trnT  -TTAACACCAAAGCATTGGTCTTGTAAACCAAAGATTGTAGACT-AA---TATCT--A-C-TCAGGACTAAAATTTATTTAA----------------------------------------    73
trnP  -------------------------------------------------------------TCAGAAC-AAGAAGAGTTTAA-CTTCTATCTTCGACCCCCAAAGCCGACATTCTAATTAAAC    60
trnT
trnP
```

1.44.2 nad5-trnL1 -36

```
                                         nad5                                                              trnL1
R       --T-ACCTGTCAACATTATTAATTACTCTTCTACTGACACTTATTTTTGTACAGTTTTTTAGAACTCATGAGAACACCTAATGCAG-CTTTTAAAGGAAAAAAGTCTTCCTCTGGCCTTAGGAGCCAG   124
nad5    GCTTACCTTGCGACTCTCTTCATCACGCTTGCCCTAGCACT---------------------------------------------------------------------------------------   41
trnL1   ----------------------------------------------------------------------------CCAATTCTGGCTTTTAAAGGAAAAAAGAATTCCCCTGGCCTTAGGCGCCAG   51
                         nad5                                                                                    trnL1
```

```
           trnL1
R       CATCTCTTGGTGCAAAT-   141
nad5    ------------------   41
trnL1   CACTTCTTGGTGCAAGTC   69
           trnL1
```

1.44.3 trnL1-trnT -64

```
                               trnL1                                                          trnT
R       AAAAAGTCTTCCTCTGGCCTTAGGAGCCAGCATCTCTTGGTGCAAATCCAAGTAGAAGCTCAGCCCTGGTAGCTTAAC-CTAAAGCATTGGTCTTGTAAGCCAAAGATTGTAAACTAA-ACCT   121
trnL1   ---------------------------------------------------------------------------------------------------------------------------   0
trnT    ----------------------------------------------------------------CCTGATAGCTTAACACCAAAGCATTGGTCTTGTAAACCAAAGATTGTAGACTAATATC-   58
                                                                                                      trnT
```

1.45 NC_010268-NC_011569

Avg ovsize: -28

LCA: Aulorhynchidae-family

1.45.1 nad4-trnS1 1

```
                                     nad4                                                       trnS1
R      ACCTAATTCCTCTACTTCTCTTGGTGTTTAAACCAGAACTAATTTGAGGTTGAACTACATGTGGGTA-TAAATACACCAGGAAAAACCTGAAAATAGGGGTGTAATCTCCCCAACCCACCGAGAGAGG  127
nad4   ACCTCATACCACTTATTCTCCTTGTACTTAAGCCTGAACTAGTGTGAGGTTGAGCCACAT--------------------------------------------------------------------  60
trnS1  -----------------------------------------------------------TGAGTGTAGTACA-ACACTAGATTTCATCTAAAAATAGGAGCTAAATCTCCTCACTCC-CAGAGAGAGG  67
                                     nad4                                                       trnS1
```

```
                        trnS1
R      CTCGCTAGCAACGAAAACTGCTAATTTTCGCAACTTTGGTTGAACCCCAAA-  178
nad4   ----------------------------------------------------  60
trnS1  CTTGCTAGCAACGGATACTGCTAATCTCCGTAACTTTGGTTGGAACCCAATG  119
                        trnS1
```

1.45.2 trnH-trnL1 -30

```
                                      trnH                                                     trnL1
R      -TTTAACAAAAATATTAGATTGTGATTCTAAAGATAGGGGTTAATCTGCCCCTCGTCCACCAT-AACTCCACCTATACCCTAAATTTAAATTTTAACCCTCATACTTCTGTAGGATAACAGCGCATCC  126
trnH   GTTTAAGCAAAATATTAGATTGTGATTCTAAAGATAGAAGCTAA-AACCTTCTCATTCACCCTTAA--------------------------------------------------------------  65
trnL1  ------------------------------------------------------------------------------------------------CCCACCCACTTCTGTAGGATAACAGCTTATCC  32
                                      trnH                                                     trnL1
```

```
                        trnL1
R      ATTGGACTTAGGATCCAAAAACTCTTGGTGCAAA-   160
trnH   -----------------------------------   65
trnL1  ATTGGACTTAGGATCCAAAGACTCTTGGTGCAAAT   67
                        trnL1
```

1.45.3 trnS1-trnH -55

```
                                   trnS1                                                                                trnH
R      CTCGCTAGCAACGAAAACTGCTAATTTTCGCAACTTTGGTTGAACCCCAAAGCTCACTCGACCTCACACACACATTTCAATTTAATATCCTCATACTAAATAAACTAGACCCTAAAAATAAGTGTAGA   128
trnS1  CTTGCTAGCAACGGATACTGCTAATCTCCGTAACTTTGGTTGGAACCCAATGCTCACTCAGCC-CACCC-----------------------------------------------------------   68
trnH   ----------------------------------------------------------------------------------------------------------------------------TAAA   4
                                   trnS1                                                                                trnH
```

```
                                trnH
R      CGTAGTTTAACAAAAATATTAGATTGTGATTCTAAAGATAG-GGGTTAATCTGCC   182
trnS1  -------------------------------------------------------   68
trnH   TGTAGTTTAAGCAAAATATTAGATTGTGATTCTAAAGATAGAAGCTAAAACCTTC   59
                                trnH
```

1.46 NC_010199-NC_005796

Avg ovsize: -30

LCA: Elopocephala-genus

1.46.1 trnS1-trnL1 3

trnS1 / trnL1

```
R      CCTGCTGGCAACGAAGACTGCTAATCC-TCATCCCCTCGGTTGAAATCCGAAGCTCACTCAATGCTCCTAAAGGATAATAGCCCTTCCATTGGTCTTAGGAACCAAAAACTCTTGGTGCAAA  121
trnS1  -CTGGAAGCACTGGGGACTGCTAATCCCTCAGCACCACGGCTAAACTCCGTGGCTCACTCG--GCCCCTAAAGGATAATAGCTCATCCGTTGGTCTTAGGAACCAAAAACTCTTGGTGCAA-  118
trnL1  ----------------------------------------------------------------------------------------------------------------------CAAA    4
```

trnS1

trnL1

1.46.2 trnH-nad5 -39

trnH

```
R     GTTTAATAAAAACATTAGATTGTGATTCTAAAGACAGAAGTTAAAACCTCCTTATCCACCCCAAAACAATAGCTCCTGAAACACTTCTTAAACCCAGTGTAGTCACCAATGCC-CTTGGTTCAAATCC  127
trnH  GTTTAAAAAAGACGTTAGATTGTGATTCTAAAAATAGGAGATAAAACCTCTTTATCCACC--------------------------------------------------------------------   60
nad5  ---------------------------------------------------------------------------------------------------TAGGAACCAAAAACTCTTGGTGCAACTCC   29
```

trnH

nad5

```
R     AAGTAGTAGCCATGCCCCCTTACACATTACCTTAACATCCACCCTAACAATAACCCTCCTGCTC-TTAGC  197
trnH  -----------------------------------------------------------------------   60
nad5  AAGTAGAGGCTATGCACTCAACAACCCTCATCTTCAATTCAAGCCTCCTAATTATCTTCGCGCTCCTTATT  100
```

nad5

1.46.3 nad4-trnS1 -54

```
                                                      nad4                                               trnS1
R      ATCTCCTCCCCCTGCTTCTCCTGATTAC-AAAACCCGAGCTAATTTGTGGCTGAATTATATGTAGGTACAACTTTAAAAGCCCTCCCCAAGAATAGACTTAAAACCCGCGCCCACGAGAGAGGCCTGC  127
nad4   ACTTAATTCCAGTATTACTTCT-AGTACTGAAGCCCGAACTAATATGAGGCTGATGCATAT-------------------------------------------------------------------   60
trnS1  -------------------------------------------------------------------------------------------------------------------GAGAGAGGC-TGG   12
                                                      nad4                                               trnS1
```

```
                   trnS1
R      TGGCAACGAAGACTGCTAATC-CTCATCCCCTCGGTTGAAATCCGAA  173
nad4   -----------------------------------------------   60
trnS1  AAGCACTGGGGACTGCTAATCCCTCAGCACCACGGCTAAACTCCGTG   59
                   trnS1
```

1.47 NC_006355-NC_009061

Avg ovsize: -31
LCA: Labroidei-suborder

1.47.1 trnI-trnM -13

```
                               trnI                                              trnM
R      CTGAATCAAAGGGTCACTTTGATAGAGTGAAGCATGGGGGTTAAAATCCTCCCAACTCCTTCCACGGAGCAAAGTAAGCTAATAGAAGCTATTGGGCCCATACCCCAAAAATGTTGACTAATACTC-  126
trnI   CTGAAATAAAGGGCCACTTTGATAGAGTGAATAATGAGGGTTAAAGTCCCTCCACCTCCT-------------------------------------------------------------------   60
trnM   -------------------------------------------------------------------------GTCAGCTAATTTAAGCTTCTGGGCCCATACCCCCGCCATGATGGTTAAAATCCT   54
                               trnI                                                 trnM
```

1.47.2 trnM-trnQ -14

```
      trnM                                                                  trnQ
R     GCTAATAGAAGCTATTGGGCCCATACCCCAAAAATGTTGACTAATACTCAGCCTTTGTTACTTTTCCCCATAGAAAAAAGGGGCTCGAACCCTTCCTGAAGAGATCAAAACTCTTGGTGCTTCCACTA   128
trnM  GCTAATTTAAGCTTCTGGGCCCATACCCCCGCCATGATGGTTAAAATCCTTCCCTT------------------------------------------------------------------------   56
trnQ  ----------------------------------------------------------------------TAGGAAAAGGGGGCTCGAACCCCTCCTGGAGAGATCAAAGCTCTCAGTGCCTCCACTA   58
      trnM                                                                    trnQ
```

```
R     -   128
trnM  -    56
trnQ  C    59
      trnQ
```

1.47.3 trnQ-nad2 -67

```
      trnQ                                                                                                                        nad2
R     GGGCTCGAACCCTTCCTGAAGAGATCAAAACTCTTGGTGCTTCCACTACACCACTTCCTAGCAAAACAAGGAAACAATTCCATTGGAATACACCCAAACAAGTTGATTAACTTCAACGATTGCTTCAT   128
trnQ  GGGCTCGAACCCCTCCTGGAGAGATCAAAGCTCTCAGTGCCTCCACTACACCACTTCCTA--------------------------------------------------------------------   60
nad2  -------------------------------------------------------------------------------------------------------------------------------T   1
      trnQ                                                                                                                        nad2
```

```
                                        nad2
R       GAGCCCCTATATTCTCTCGATCTTTCTTT-TCGGACTCGGCCTAGGAACCACAATTAC-   185
trnQ    -----------------------------------------------------------    60
nad2    GAACCCCTATATTCTAGGCACCCT-CTTGCTTGGGCTCGGCCTAGGTACAACCCTAACA    59
                                        nad2
```

1.48 NC_001922-NC_015305

Avg ovsize: -33

LCA: Tetrapoda-superclass

1.48.1 trnL1-nad5 3

```
                                  trnL1                                                                    nad5
R       AACTAGGACATCCCCTGGCCTTAGGAGCCAATA-ATCTTGGTGCAACCCCAAGTGAAAATACCA-TGCAA--CA-GCCGACCCTATCT-C--TTACCTTC-TT-C-ACCCTACCTGCTTTTATCCTT-   116
trnL1   AAACA-GCCCTCCACTGGCCTTAGGAGCCAGCACCTCTTGGTGCAAGTCCAAGT-AAAA---------------------------------------------------------------------    57
nad5    --------------------------------------------------------AAATCCCAGTACGAGTCATGCCATCCCTATATACAATTACTTTTGTGACAACAATATCTGCAATCG-CCTTA    71
                                 trnL1
                                                                                                  nad5
```

```
         nad5
R       -TTACT   121
trnL1   ------    57
nad5    ATTATT    77
         nad5
```

1.48.2 trnS1-trnH -12

```
                              trnS1                                                    trnH
R       ---CC-GCGCA--G-GAACTGCTAATTCCTAGCCCCCAGGACTAACAGCCTGGCCCTCTCGTCGTGGGCTCGGTAAGTATAGTTTAAACAA-AACATTAGAATGTGAACCTAAAAATAGGAGACTACT   120
trnS1   TGCCCGGCATAGTGAGAACTGCTAATTCCTCACCACATGGTTCGACT-CCATGCTCGCTCG-------------------------------------------------------------------    60
trnH    -------------------------------------------------------------------------TGAGTGTAATT-AAACAATAATGCTAGATCGTGACTCTAAAATTGAAAGTTAAAA    54
                              trnS1
                                                                                                  trnH
```

```
         trnH
R       TCC-C   124
trnS1   -----    60
trnH    TCCTT    59
         trnH
```

1.48.3 cob-trnT -92

```
                              cob                                                                             trnT
R       -TACCAATAGCAGGCCTCTTAGAGAACAAAATAGTTGAACCCACCTATGTTACCCCTAAGTCCCTCCATCCCAACAAGACCAAACCTCACCACACTCCCG-TGGCTAAAGGAAAAGCGCTGGCCTTGT   126
cob     CTA-----------------------------------------------------------------------------------------------------------------------------     3
trnT    -----------------------------------------------------------------------------------------------TCCTGATAGCTTAACTAA-AGCACTGGTCTTGT    32
        cob
                                                                                                              trnT
```

```
                      trnT
R     AAGACAGA-AGTGGA-CGGAACC-AAC   150
cob   ---------------------------     3
trnT  AAGCCAGAGATTGCAGCCTGACCTCTG    59
                      trnT
```

1.49 NC_005961-NC_014174

Avg ovsize: -34

LCA: Toxicofera-clade

1.49.1 trnQ-trnA -11

```
                                    trnQ                                                                          trnA
R     --ACTCGAACCCGCACCTGAAAGATCAAAACTTCCAGTACTACCTCATTGTACTACCCCTTACT-AA-TAGAACTCGCGACAACTCATC-ACATCTCCTGCATGCAACACAGACACTTTAATTAAGCC   123
trnQ  GGAGTTGAACCAACATCAAAAAATTCAAAATTTTATGCATTT-CT-ATTATACTACCCACTACTTAACTA----------------------------------------------------------    68
trnA  ---------------------------------------------------------------------------------AACT-ATCAACATCTTCTGAATGCAACTCAGATACTTTAATTAAGCT    46
                                   trnQ
                                                                                                            trnA
```

```
R     -   123
trnQ  -    68
trnA  A    47
      trnA
```

1.49.2 trnW-trnQ -28

trnW trnQ

```
R     GGTTATACACCCATTAAACCTGAGGCCTTCAAAGCCCCCAAAAAACAATTTTAGCTTCTGCTACACTAAAGAAGCAGGACTCGAACCCGCACCTGAAAGATCAAAACTTCCAGTACTAC-CTCAT  124
trnW  GGATATA-T----TTAAACCAATAGCCTTCAAAGCCATTAAAAA---------------------------------------------------------------------------------   39
trnQ  ------------------------------------------------------------------------AGAAGGAGTTGAACCAACATCAAAAAATTCAAAATTTTATGCATTTCTATTAT   53
```

trnW

trnQ

1.49.3 nad1-trnI -63

nad1 trnI

```
R     CTATGCCTCCTACACATCTCCCTGCCAATCTCACTAGGTGGGACACCACCAACACACTAATCCACGGAAGCGTGCCTGAGAA--AAGGACTACCTTGATAAAGTAAAAACAGAGCATTGAACCTCT  124
nad1  ------------------------------------------------------------------------------------------------------------------------------    0
trnI  ---------------------------------------------------------------ATGGAAAAGTGCCCGAGAACTAAGGATTACTTTGATAAAGTAAAAACAGAG-ACACAATCTCT   62
```

trnI

1.50 NC_003159-NC_006340

Avg ovsize: -34

LCA: Teleostomi-superclass

1.50.1 trnA-trnN 20

```
                  trnA                                   trnN
R     CAGGACTTTATCCCACATCTTCTGAATGCAACCCAGAAACTTTTATTAAGCTAAGGCCCTCTAGATGAGAGGGCCTCG-ATCCCCCAAACTCTTAGTTAACAGCTAAGCGCCCCA-ACCAG  119
trnA  CGAGACTCTACCCCACATCTTCTGAATGCAACCCAGACACTTTAATTAAGCTAAAGCCTTC-ACAAACTACA-CCTCATATTCTTGTAA-T-TTAATAAACAACTAA--------------  103
trnN  ---------------------------------------------------------------------------------------AA-T-TTAGTTAACAGCTAAACACCAGATTTCAG   32
                  trnA
                                                                                                        trnN
```

1.50.2 trnN-trnY -32

```
                   trnN                                                             trnY
R     -CCTC-GATCCC-CCAAACTCTTAGTTAACAGCTAAGCGCCCCAACCAGCGAGCATCCATCTAACTGACCAGCCTGGTA-GGAAGAGGACTTAAACCTCTCTTTATGGGGCTACAACCCACCACTTAA  124
trnN  GGATTTTATCCTACTACAAT-TTAGTTAACAGCTAAACACC---------------------------------------------------------------------------------------   40
trnY  -------------------------------------------------------------------------CTGGTAAGGAAAGGGAC---CACCTTTATAAATGAGGCTACAACTCAACACCTAT   52
              trnN
                                                                                          trnY
```

```
       trnY
R     ATC-TC-A-   130
trnN  ---------    40
trnY  TTCGGCCAC    61
       trnY
```

1.50.3 trnY-trnC -90

```
                                 trnY
R     AGGAC-TTA-AACCTCTCTTTATGGGGCTACAACCCACCACTTAAATCTCAGCCACCCTACCTTATACCCTGTTACGCCCCTGAACCACCTCAACACATGCTCCCCCATAGATTAACCACACTTATCG   126
trnY  GGAAAGGGACCACCTTTATAAATGAGGCTACAACTCAACACCTAT-T-TCGGCCACCTTACCTT--ACACTGATATGCAC------------------------------------------------    76
trnC  --------------------------------------------------------------------------------------------------------------------------------     0
                                 trnY
```

```
                                                                         trnC
R     CCCACATAAGCAGGTCTCCTTTCGCCGCCGGGCGTCGACGGAAAGCCCCGGCAGGCTGGAAAC-CTGCTTCTTCAGATTTGCAATCTGACGTGTCAC-AC-CT-C   227
trnY  ---------------------------------------------------------------------------------------------------------    76
trnC  ------------------------------------------AAGCCCTGGCAGA-T--ACACTCTGCTCCTTAAAATTTGCAATTTCATGTGCTACTACACTAC    60
                                                                       trnC
```

1.51 NC_011381-NC_006355

Avg ovsize: -34

LCA: Percomorpha-order

1.51.1 trnI-trnQ 6

```
                                trnI                                                          trnQ
R     -TGAAATAAAAGGATCATTTTGATAGAATGCCTCATGAGGGTTTAAGTCCCTCCATTCTCCTTAGAAAGAAGGGGTTTGAACCCCACCTGAAGAGATCAAAACTCTTAGTGCTTCCACTA-   119
trnI  CTGAA-TCAAAGGGTCACTTTGATAGAGTGAAGCATGGGGTTAAAATCCTCCCAA-CTCCTT-----------------------------------------------------------    61
trnQ  ---------------------------------------------------------CCCCATAGAAAAAAGGGGCTCGAACCCTTCCTGAAGAGATCAAAACTCTTGGTGCTTCCACTAC    64
                                trnI
                                                                                              trnQ
```

1.51.2 trnM-trnI -3

```
R     AGCTAA-ATAAGCTTTTGGGCCCATACCCCAAACATGTTGGTTAAAATCCTTCCTTTACTAATGACGGAGATGTGCCTGAAATAAAAGGATCATTTTGATAGAATGCCTCATGAGGGTTTAAGTC--   124
trnM  -GCTAATAGAAGCTATTGGGCCCATACCCCAAAAATGTTGACTAATACTCAGCCTTTGTTACT----------------------------------------------------------------    62
trnI  ------------------------------------------------------------------GGAGCTGTGCCTGA-ATCAAAGGGTCACTTTGATAGAGTGAAGCATGGGGGTTAAAATCCT    60
```

1.51.3 nad1-trnM -106

```
R     CTTGTAATCTGACATCTCTCATTACCAATTGCCTTCTTTGGTCTTCCCCCCCAACTTTAATATTTACAGAGATATGCCTGAAATAAAGGATCCCCTTGATAGAGTGCCCCTTAGAATAAAAGAAGTTT   128
nad1  TTTATAATCTGACATCTGGCACTACTAATTGCATTCGCCGGACTTCCCCCACACCTTTAACA------------------------------------------------------------------    62
trnM  --------------------------------------------------------------------------------------------------------------------------------     0
```

```
R     ATAACCCCACCTGAGGGGATTAAAACTCTTAGTACTTCCTAGTAAAGTCAGCTAA-ATAAGCTTTTGGGCCCATACCCCAAACATGTTGGTTAAAATCCT   227
nad1  ----------------------------------------------------------------------------------------------------    62
trnM  ----------------------------------------AGCAAAGTAAGCTAATAGAAGCTATTGGGCCCATACCCCAAAAATGTTGACTAATACTCA    60
```

1.52 NC_014174-NC_005961

Avg ovsize: -35

LCA: Toxicofera-clade

1.52.1 trnQ-trnI -5

```
        trnQ (R, above)                                                   trnI (R, above)
R       GGAGTTGAACCAACATCAAAAAATTCAAAATTTTATGCATTTC-T-ATTATACTACCCACTACTTAACTAATGGAAAAGTGCCCGAGAACTAAGGATTACTTTGATAAAGTAAAAACAGAG-ACACAA   125
trnQ    --ACTCGAACCCGCACCTGAAAGATCAAAACTTCCAGTACTACCTCATTGTACTACCCCTTAC-TAA-------------------------------------------------------------    64
trnI    ------------------------------------------------------------------------GGAAGCGTGCCTGAGAA--AAGGACTACCTTGATAAAGTAAAAACAGAGCATTGAA    54
        trnQ (below)                                                      trnI (below)
```

```
        trnI
R       --TCTC   129
trnQ    ------    64
trnI    CCTCTC    60
        trnI
```

1.52.2 trnW-trnA -39

```
        trnW (R, above)                                                   trnA (R, above)
R       GG--ATATAT---TTAAACCAATAGCCTTCAAAGCCATTAAAAAGAGATAACCTCTTAGTTCCTGTGTATTAAAGCTTATTGAAACT-ATCAACATCTTCTGAATGCAACTCAGATACTTTAATTAAG   122
trnW    GGTTATACACCCATTAAACCTGAGGCCTTCAAAGCCCCCAAAAA------------------------------------------------------------------------------------    44
trnA    -----------------------------------------------------------------------------------AACTCATC-ACATCTCCTGCATGCAACACAGACACTTTAATTAAG    44
        trnW (below)                                                      trnA (below)
```

```
      trnA
R     C-T   124
trnW  ---    44
trnA  CCA    47
      trnA
```

1.52.3 nad1-trnQ -61

```
                                                      nad1                                                    trnQ
R     ACACTAGCAATATGCTTAATTTACTCAATAGTGCCCCTTGCACTAGCAGCCCTTCCATAGTGGAGAAGGAGTTGAACCAACATCAAAAAATTCAAAATTTTATGCATTTC-T-ATTA   115
nad1  ----------------------------------------------------------------------------------------------------------------------     0
trnQ  -------------------------------------------------------------GAAGCAGGACTCGAACCCGCACCTGAAAGATCAAAACTTCCAGTACTACCTCATT-    55
                                                                                                              trnQ
```

1.53 NC_007012-NC_001922

Avg ovsize: -36

LCA: Teleostomi-superclass

1.53.1 nad4-trnH -11

```
                                            nad4                                                          trnH
R     --AC-CTAA-TCCCTGTC-CTTCTCCTAGTCCTTAAACC-CGAACTTATATGAGGGGTGTGCCACTGTAAATATAGTTTAAAAAAAACATTAGATTGTGATTCTAAAAACAAAAGTTAAAATCTT-   119
nad4  TTACACACACTACCA-TCAATTATCCTCATCACAAACCCACGA-CTTATTT---------------------------------------------------------------------------    49
trnH  --------------------------------------------------------------CTCGGTAAGTATAGTTTAAACAAAACATTAGAATGTGAACCTAAAAATAGGAGACTACTTCCCC    64
                                  nad4
                                                                                                       trnH
```

1.53.2 trnS1-trnL1 -41

```
                                trnS1                                                 trnL1
R      CGGAAGCACTAAGGACTGCTAATCCTTAAGTCCCATGGTTCAAGTCCATGGCTCACTTAGCCCCTAAAGGATAAC-AGCTCATCCATCGGTCTTAGGAACCGAAAACCCTTGGTGCAA-C   118
trnS1  -G-AGGG-C-CAGG-CTGTTAGTCCT----------------------------------------------------------------------------------------------   21
trnL1  -------------------------------------------------------------------AGGATAACTAGGACATCCCCTGGCCTTAGGAGCCAATAATC-TTGGTGCAACC   52
              trnS1
                                                                                           trnL1
```

1.53.3 trnH-trnS1 -58

```
                                 trnH                                                  trnS1
R      -GTTTAAAAAAAACATTAGATTGTGATTCTAAAAACAAAAGTTAAAATCTTTTTATTTACCAAGAGAGGCCGGAAGCACTAAGGACTGCTAATCCTTAAGTCCCATGGTTCAAGTCCATGG   120
trnH   AGTTTAAACAAAACATTAGAATGTGAACCTAAAAATAGGAGACTACTTCCCCTTACTCACC------------------------------------------------------------   61
trnS1  -----------------------------------------------------------------------------------------------------------------------GG   2
                                trnH
                                                                                                                         trnS1
```

1.54 NC_012762-NC_012761

Avg ovsize: -37

LCA: Lorisiformes-infraorder

1.54.1 trnI-nad1 0

1.54.2 trnL2-trnI -48

trnL2 *trnI*

```
R      CCCGGTAATTGCATAAAACTTAAAACTTTACCACCAGAGGTTCAACTCCTCTTCTTAACACATGTTTGTTCTAAACCTCCTACTACTCATCATTCCCATTATCCTGGCCATAGCCTTC   118
trnL2  CCTGGCAATTGCATAAAACTTAAAACTTTAGAAACAGAGGTTCAACTCCTCTTCTTAACA----------------------------------------------------------    60
trnI   ------------------------------------------------------------------------------------------------------------CAAACCCT-C     9
```

trnL2

trnI

1.54.3 nad1-trnQ -65

nad1 *trnQ*

```
R      ATATGCATATGACACGCCTCACTACCCATCTCCTTGGCCAACATCCCCCCTCAAACATAGAAATATGTCTGACAATAGAATTACTTTGATAGAGTAAAACATAGAGGTGCGAACCCTCTTATTTCTAG   128
nad1   CTATGCATATGATATGTTTCACTACCCATTATCTTAGCCAGCATTCCCCCCCAAACATAG--------------------------------------------------------------------    60
trnQ   -----------------------------------------------------------------------------------------------------------------------------TAG     3
```

nad1

trnQ

trnQ

```
R      GATAATAGGACTTGAACCCACTCTTAAGAACTCAAAATTCTCCGTGCTACCTA-TC   183
nad1   --------------------------------------------------------    60
trnQ   GACAGCAGGCCTTGAACCTACCCCTGAGAACTCAAAATTCTCCGTGCTACCTACTA    59
```

trnQ

1.55 NC_005961-NC_023228

Avg ovsize: -40
LCA: Teleostomi-superclass

1.55.1 trnQ-trnA -10

```
                                   trnQ                                                         trnA
R     -ACTCGAACCCGCACCTGAA-AGATCAAAACTTCCAGTACTACCTCATTGTACTACCCCTTACTAATAGAACTCGCGACA-ACT-CATCACATCTCCTGCATGCAACACAGACACTTTAATTAAGCC   123
trnQ  GATTCGAACCT-CAACTTAAGAGATCAAAACTCTTTGTGC-ACTCCATAACACTA------------------------------------------------------------------------    53
trnA  -----------------------------------------------------------------ATAAAACTTGCGGGATACTACCCCACATCTTCTGCTTGCAAAGCAGACACTTTAATTAAGC-    61
                                trnQ                                                         trnA
```

1.55.2 nad1-trnI -55

```
                                 nad1                                                          trnI
R     CTATGCCTCCTACACATCTCCCTGCCAATCTCACTAGGTGGGACACCACCAACACACTAATCCACGGAAGC-GTGCCTGAGAAAAGGACT-ACCTTGATAAAGTAAA-AACAGAGCATTGAACCTCT-   124
nad1  TT------------------------------------------------------------------------------------------------------------------------------     2
trnI  ---------------------------------------------------------TTATCCCCAGGAGCTATGTCTGAATAAAG-AGTCACTTTGATAGAGTGAAGCACGTAG-ATGAAAACCCTA    69
      nad1                                                                                             trnI
```

1.55.3 trnW-trnQ -57

```
trnW (R: positions 2-58)  trnQ (R: positions 67-128)
R      -GGTTATACACCCATTAAACCTGAGGCCTTCAAAGCCCCCAAAAAACAATTTTAGCTTCTGCTACACTAAAGAAGCAGGACTCGAACCCGCACCTGAA-AGATCAAAACTTCCAGT--ACTACCTCAT   124
trnW   AGGTTAT-------------------------------------------------------------------------------------------------------------------------     7
trnQ   ----------------------------------------------------------------CAATAGGAAAGGAGGATTCGAACCT-CAACTTAAGAGATCAAAACTCTTTGTGCACT-CCATAA    62
trnW (lower: positions 1-8)  trnQ (lower: positions 65-128)
```

1.56 NC_005796-NC_010199

Avg ovsize: -40

LCA: Elopocephala-genus

1.56.1 trnH-trnS1 32

```
trnH  trnS1
R      GTTTAAAAAAGACGTTAGATTGTGATTCTAAAAATAGGAGATAAAACCTCTTTATCCACCGAGAGAGGC-TGGAAGCACTGGGGACTGCTAATCCCTCAGCACCACGGCTAAACTCCGT--   118
trnH   GTTTAATAAAAACATTAGATTGTGATTCTAAAGACAGAAGTTAAAACCTCCTTATCCAC--------------------------------------------------------------    59
trnS1  ---------------------------CCAAGAATAG-ACTTAAAACC-CGCGC-CCAC-GAGAGAGGCCTGCTGGCAACGAAGACTGCTAATCC-TCATCCCCTCGGTTGAAATCCGAAG    89
trnH  trnS1
```

1.56.2 trnS1-nad5 -34

```
                                trnS1
R      -CTGGAAGCACTGGGGACTGCTAATCCCTCAGCACCACGGCTAAACTCCGTGGCTCACTCGGCCCCTAAAGGATAATAGCTCATCCGTTGGTCTTAGGAACCAAAAACTCTTGGTGCAACTCCAAGTA   127
trnS1  CCTGCTGGCAACGAAGACTGCTAATCC-TCATCCCCTCGGTTGAAATCCGAAGCTCACTC-------------------------------------------------------------------    59
nad5   ----------------------------------------------------------------------------------------------TAGTCACCAATGCC-CTTGGTTCAAATCCAAGTA    33
                               trnS1
```

```
                                  nad5
R      GAGGCTATGCACTCAACAACCCTCATCTTCAATTCAAGCCTCCTAATTATCTTCGCGCTCCTTA--T   192
trnS1  -------------------------------------------------------------------    59
nad5   GTAGCCATGCCCCCCTTACACATTACCTTAACATCCACCCTAACAATAACCCTCCTGCTC-TTAGCC    99
                                 nad5
```

1.56.3 nad4-trnL1 -120

```
                                nad4
R      ACTTAATTCCAGTATTACTTCT-AGTACTGAAGCCCGAACTAATATGAGGCTGATGCATATGTAGATATA-GTTTAAAAAAGACGTTAGATTGTGATTCTAAAAATAGGAGATAAAACCTCTTTATCC   126
nad4   ATCTCCTCCCCCTGCTTCTCCTGATTACAAAA-CCCGAGCTAATTTGTGGCTGAATTATATGTAGGTACAACTTTAAAA-------------------------------------------------    78
trnL1  --------------------------------------------------------------------------------------------------------------------------------     0
                                nad4
```

```
                                                                                                                trnL1
R      ACCGAGAGAGGCTGGAAGCACTGGGGACTGCTAATCCCTCAGCACCACGGCTAAACTCCGTGGCTCACTCGGCCCCTAAAGGATAATAGCTCATCCGTTGGTCTTAGGAACCAAAAACTCTTGGTGCA   254
nad4   --------------------------------------------------------------------------------------------------------------------------------    78
trnL1  -----------------------------------------------------------------------GCTCCTAAAGGATAATAGCCCTTCCATTGGTCTTAGGAACCAAAAACTCTTGGTGCA    57
                                                                                                           trnL1
```

```
                trnL1
R      ACTCCAAGTAGAGGCT   270
nad4   ----------------    78
trnL1  A----------A---T    60
                trnL1
```

1.57 NC_012688-NC_022670

Avg ovsize: -41
LCA: Neoptera-subclass

1.57.1 trnM-trnQ 0

1.57.2 rrnS-trnM -20

```
                          rrnS                                                      trnM
R     TTATATTTTATATAAAATACTGAATTTTTTTAC-TCAAAATCAAAAGATTAATTTTAAAAATAAA-AGATAAGCTAATA--AAGCTTAAAGACTCATAATCTTTATATAGAAATTTCAATTTT   119
rrnS  A-A-ATTAT-TATAAGG-ACT-AATTGTTTAACCGCAA-------------------------------------------------------------------------------------    33
trnM  ----------------------------------------------------------AAATAATTAGATAAGCTAATATTAAGCTTATAGGTTCATACCCTATCAATAGAAGTAA-AACCTT    64
                rrnS                                                                          trnM
```

1.57.3 trnI-nad2 -103

```
                                    trnI
R     -G---CCTGATAAAAGGATTATTTTGATAGAATAATTCATG-AAAATAAAGTTTTT-CTTTCATTTAATAATTTCAATTTTTAAAATTTATTAAATTATTGCTAATAATATATATATTATTAATTAAT  122
trnI  AGATGCCTGAATAAAGGATTACTTTGATAGAGTAAATCATGCACAATTATGCTCTTACTATAAAT---------------------------------------------------------------   65
nad2  --------------------------------------------------------------------------------------------------------------------------------    0
                                  trnI
```

```
                        nad2
R     AAAATTATTGATCATATTAATTTTCTATTTTATTTTAATATTA-AGAACTTTAATTAC  179
trnI  ----------------------------------------------------------   65
nad2  ----------------------------------------TTATAGTAATATCATCA-   17
                                                  nad2
```

1.58 NC_006355-NC_023228

Avg ovsize: -44
LCA: Percomorpha-order

1.58.1 trnM-trnQ -3

```
                            trnM                                                          trnQ
R     GCTAATAGAAGCTATTGGGCCCATACCCCAAAAATGTTGACTAATACTC-AGCCTTTGTTACTTTTCCCC-ATAGAAAAAAGGGGCTCGAACCCTTCCTGAAGAGATCAAAACTCTTGGTGC-TTCCA  125
trnM  GCTAAAACAAGCTATTGGGCCCATACCCCAGCCATATAGGTTAAAACCCTATCCAT------------------------------------------------------------------------   56
trnQ  -----------------------------------------------------------TA-TTATCCCCAATAGGAAAGGAGGATTCGAACCTCAACTTAAGAGATCAAAACTCTTTGTGCACTCCA   68
                          trnM                                                          trnQ
```

```
       trnQ
R      CTA   128
trnM   ---    56
trnQ   TAA    71
       trnQ
```

1.58.2 nad1-trnI -9

```
R     TTTATAAT-CTGACATCTGGCACTACTAATTGCATTCGCCGGACTTCCCCCACACCTTTAACAGGAGCTGTGCCTGAATCAAAGGGTCACTTTGATAGAGTGAAGCATGGGGGTTAAAATCC--   121
nad1  TTCGTGATAGTAAC-CCTAATAATTCTAGTTATAACAGCAGGTATTCCTCCA------------------------------------------------------------------------    51
trnI  -------------------------------------------------------------CAGGAGCTATGTCTGAAT-AAAGAGTCACTTTGATAGAGTGAAGCACGTAGATGAAAACCCTA    62
```

Annotations: R — nad1 (columns 1–61), trnI (from column 64); nad1 row — nad1; trnI row — trnI.

1.58.3 trnQ-nad2 -120

```
R     GGGCTCGAACCCTTCCTGAAGAGATCAAAACTCTTGGTGCT-TCCACTACACCACTTCCTAGCAAAACAAGGAAACAATTCCATTGGAATACACCCAAACAAGTTGATTAACTTCAACGATTGCTTCA   127
trnQ  GA-TTCGAACCTCAACTTAAGAGATCAAAACTCTTTGTGCACTCCATAACACTATTTCCTA-------------------------------------------------------------------    60
nad2  --------------------------------------------------------------------------------------------------------------------------------     0
```

Annotations: R — trnQ (columns 1–61), nad2 (last columns); trnQ row — trnQ.

```
                                         nad2
R     TGAGCCCCTATATTCTCTCGATCTTTCTTTTCGGACTCGGCCTAGGAACCACAATTAC-   185
trnQ  -----------------------------------------------------------   60
nad2  -----------------------------------------------------ATTACA   6
                                                           nad2
```

1.59 NC_006286-NC_016755

Avg ovsize: -45
LCA: Laterata-suborder

1.59.1 trnE-nad6 -5

```
                                trnE                                                                   nad6
R     GCCGGCAATTCTA-CCGG-ACCTGCGGTCTGAAAAACCGCCGTTG-TGTTCAACTACAAAAACCCCCCTAAATACCCGAATAGCGCCCCGTTCCCCACCTAAAGATAACCATAATACCACAAAAA-   123
trnE  -CTGGC--TTATATCCAAAACCTGCGACCCGAAAAACCGCTGTTGTTATTCAACTATTAAAGC----------------------------------------------------------------   60
nad6  --------------------------------------------------------------------CTAACCGCACAAAGACTTCCCCGCACCATCCCCGAGTTAACTCTAACACAACAAATAG   59
                                trnE                                                                   nad6
```

1.59.2 nad5-trnE -58

nad5 trnE

```
R     GGACGA-ATA-AAATCTTATTTATCATTATTCCTACTAACCATGTTTATCCTACTATTCTAGACTTGACAATTTCCGCCGGCAATTCTACCGGACCTGCGGTCTGAAAAACCGCCGTTGTG-TTCAAC   125
nad5  TT-CTATTTATCAATTTTCTTAATCACT-TCCCTA---------------------------------------------------------------------------------------------   33
trnE  ---------------------------------------------------------------------------------------------ACCTGCGACCCGAAAAACCGCTGTTGTTATTCAAC   35
```

nad5

trnE

trnE

```
R     T   126
nad5  -    33
trnE  -    35
```

1.59.3 nad6-cob -72

nad6

```
R     CGCAACACCAACAATAGCTATCAAAAATGCCCACCCCAA-AAGA-TAAGTAATATATATCATAGAGCTTACTACCACAACGGTAGCACATCAGAAAATAAAATTCTGCTATCACTACAACTACAAGCA   126
nad6  CGCAATACCAACCAAACCTGCCACAAAA-CACACCACAAGAAAACTTAC-AAAATATATCAT------------------------------------------------------------------   60
cob   --------------------------------------------------------------------------------------------------------------------------------   0
```

nad6

cob

```
R     CCAATAATGGCCCACATCACACGCAAGCATCATCCAATCTTTAAAATGGTCAACAGCACATTTA-T   191
nad6  ------------------------------------------------------------------   60
cob   ------ATGACCTCCAACATACGAAAACAACACCCAATCTTAAAAATCGTAAATAGCTCCTTCATT   60
```

cob

1.60 NC_014174-NC_012762

Avg ovsize: -45
LCA: Amniota-clade

1.60.1 trnI-trnM 2

trnI *trnM*

```
R     GCCCGAGAACTAAGGATTACTTTGATAAAGTAAAA-ACAGAGACACAATCTCTC-TATTCCCATTAGAATCTGCTACACAAGCAATTGGGCCCATACCCCAAAAACGATAACATATAT-CTT  119
trnI  T-CTGACAA-T-AGAATTACTTTGATAGAGTAAAACATAGAGGTGCGAACCCTCTTATTTCTA-----------------------------------------------------------  60
trnM  -------------------------------------------------------------CAGTAAGGTCAGCTAAATAAGCTATCGGGCCCATACCCCGAAAATGTTGGTTTATACCCTT  61
```

trnI

trnM

1.60.2 trnL2-nad1 -65

trnL2 *nad1*

```
R      GCTCGGTTATTGCAAGAGGCCTAAAACCTTTA--ACCAGAAGTTCAAATCTTCTCCACAACAAATATCATGATTTTACAAATCATTAAAATCCTTAACCTAGTAGCCGTATTTATCTCAATTTTGAT  125
trnL2  CC-CGGTAATTGCATAAAACTTAAAA-CTTTACCACCAGAGGTTCAACTCCTCTTCTTAACA-----------------------------------------------------------------  60
nad1   -------------------------------------------------------------------------------------------------------------------------------  0
```

trnL2

1.60.3 trnQ-trnI -74

```
        trnQ (upper: R columns 1–53)                                   trnI (upper: R columns 75–128)
R       GGAGTTGAACCAACATC-AAAAAATTCAAAATTTTA--TGCATTTCTATTATACTACCCACTACTTAACTAATGGAAAAGTGCCCGAGAACTAAGGATTACTTTGATAAAGTAAAAACAGAGACACAA   125
trnQ    -GACTTGAACCCAC-TCTTAAGAACTCAAAATTCTCCGTGC-TACCTATCACAC--------------------------------------------------------------------------    51
trnI    --------------------------------------------------------------------------------------------------------------------------------     0
        trnQ (lower: trnQ columns 2–54)
```

```
        trnI
R       TCTC   129
trnQ    ----    51
trnI    TC-C     3
        trnI
```

1.61 NC_006355-NC_005961

Avg ovsize: -50

LCA: Teleostomi-superclass

1.61.1 trnM-trnQ -12

```
        trnM (upper: R columns 2–65)                                                 trnQ (upper: R columns 80–128)
R       -GCTAA-TA-G-AAGCTATTGGGCCCATACCCCAAAAATGTTGACTAATACTCAGCCTTTGTTACTTTTCCCCATAGAAAAAAG-GGG-CTCGAACCCTT-CCTGAAGAGATCAAAACTCTTGGTGCT   121
trnM    AGTCAGCTAAGCAAGCTATTGGGCCCATACCCCAAAAATGTT-A-TAC-AC-CAACCTCTACTAC---------------------------------------------------------------    61
trnQ    -----------------------------------------------------------------------------AAAGAAGCAGGACTCGAACCCGCACCTGAA-AGATCAAAACTTCCAGTACT    50
        trnM (lower: trnM columns 1–65)                                              trnQ (lower: trnQ columns 78–128)
```

trnQ

```
R       TCCAC-TA   128
trnM    --------    61
trnQ    ACCTCATT    58
```

trnQ

1.61.2 trnW-trnA -30

trnW *trnA*

```
R       ----ATA-ACC--TCAGACCAAGGGCCTTCAAAGCCCTAAGTGGGAGTGGAACTCTCCCAGACCCTGAATAAAACTTGCAGGGGTCTCACCTACATCTTCTGCTTGCAAAGCAGACACTTTAATTAAG   121
trnW    GGTTATACACCCATTAAACCTGAGGCCTTCAAAGCCC-------------------------------------------------------------------------------------------    37
trnA    -------------------------------------------------------------------AATAGAACTCGC-GACAACTCATC-ACATCTCCTGCATGCAACACAGACACTTTAATTAAG    59
```

trnW

trnA

```
R       ---   121
trnW    ---    37
trnA    CCA    62
```

trnA

1.61.3 trnQ-nad2 -108

trnQ

```
R       GGGCTCGAACCCT-TCCTGAAGAGATCAAAACTCTTGGTGCTTCC--A--CTAC-ACCACTTCCTAGCAAAACAAGGAAACAATTCCATTGGAATACACCCAAACAAGTTGATTAACTTCAACGATTG   122
trnQ    -A-CTCGAACCCGCACCTGAA-AGATCAAAACTTCCAGTACTACCTCATTGTACTACCCCTTACTA--------------------------------------------------------------    63
nad2    --------------------------------------------------------------------------------------------------------------------------------     0
```

trnQ

```
                                    nad2
R     CTTCATGAGCCCCTATATTCTCTCGATCTTTCTTTTCGGACTCGGCCTAGGAACCACAATTA-C-   185
trnQ  -----------------------------------------------------------------   63
nad2  ----------------------------------------------CTAGGAACC-CTCTTAGCC   18
                                                         nad2
```

1.62 NC_003163-NC_003159

Avg ovsize: -51

LCA: Neoteleostei-subclass

1.62.1 trnE-trnP -17

```
                                trnE                                                                         trnP
R     CAGGATTTTAACCTGGACCAATGACATGAAAAGCCACCGTTGTGATTCAACTATAAGAACCCATCCGGGCTCTGCCACAAACTCAAAACGACAAGACTTACACTCGCACTTCCGGCACCCAAAGCCGG   128
trnE  CAGGACTCTAACCAAGACCAGCGACTTGAAAAACCGCCGTTGTTTTTCAACTACAAGAACCCA-----------------------------------------------------------------   63
trnP  --------------------------------------------------------------------------------ACTCAGAGAGGGGAGATTTTAACTCCCACCCTTAGCTCCCAGAGCTAA   48
                                trnE                                                                         trnP
```

```
           trnP
R     TGCTCTTAA-TTA-A   141
trnE  ---------------   63
trnP  -GATCCTAAACTAGA   62
           trnP
```

1.62.2 nad5-cob -50

```
                                     nad5                                                       cob
R     TA--CCTA--TCC-C-TATTCTTTATAACCCTTTGC-CTAGCCACCCTCCTCACCACAAACCTTTAAGGCCCCCCCCCCCCTAATGACCCACCCGACACTACGCAAAACCCACCCCCTCCTAAAAATC   121
nad5  AAAACCTACCTCCGCCTCTCCCTTCTAACACTA-GCTCTAGCCACCTT--------------------------------------------------------------------------------    47
cob   --------------------------------------------------------------------------------------------------CTACGAAAAACCCACCCCCTTCTAAAAATT    30
                           nad5                                                                                          cob
```

```
           cob
R     G-CAA--ACG-A-T-GC-CCT   135
nad5  ---------------------    47
cob   GCCAACCACGCACTAGTAGAC    51
           cob
```

1.62.3 trnT-nad6 -87

```
                                  trnT
R     AGTATCCAGAGCGCCGGTTTTGTAACCCGGAGGCCGGAAGTTCAAATCTCCCCGAGCGCACAGCCTAGCCCAGGCTCTGCCACAAAAATTAACCTATTGGCCAAGGCCTGATCTGCCTGACCCCCCCC   128
trnT  AACATGTCGAGCACCGGACTTGTAATCCGGGGGTCGGGGGTTTTAAATCCCCCCTAGTGCTCACCC-CGCCC---------------------------------------------------------    70
nad6  --------------------------------------------------------------------------------------------------------------------------------     0
                                  trnT
```

```
                                                          nad6
R     CCGCCTTCTTTCTCACCGGCCTACCGGCCCTAAACGGCACGCAAGGCCCCTCGACTCAACCCCCGAGTTAACTCTAGAACCACAAAT-A   216
trnT  -----------------------------------------------------------------------------------------    70
nad6  ------------------------------TAAACGGCCCGCAAAGTCCCTCGACTTAACCCCCGGGTTAACTCAAGCACTCCTAATAA    59
                                                           nad6
```

1.63 NC_005796-NC_006405

Avg ovsize: -60

LCA: Teleostomi-superclass

1.63.1 trnL1-trnH 0

1.63.2 cob-trnT -8

```
R     -TAT-TCCTAGTATTAAATCCAC-TAGTAGGATGACTAGAGAATAAAGCACTTAAATGAGCTTGCCCTAG-TAGCTTATA-AAAAGCACCGGTCTTGTAATCCGAAGATTGAAGGTTAAAGTCC   119
cob   ATCTTTGCTCC-ATTC-TTCCCCATCATTGGACTCTTAGAAAACAAGTTACTTCA---------------------------------------------------------------------    53
trnT  ---------------------------------------------------------------GCCC-AGATAGCTTATATTAAAGCATCGGTCTTGTAAACCGAAGATTGTAGATTAAACCCT    60
```

1.63.3 nad4-trnL1 -174

```
R      -ACTTAATTCCAGTATTACTTCTAGTACTGAAGCCCGAACTAATATGAGGCTGATGCATATGTAGATATAGTTTAAAAAAGACGTTAGATTGTGATTCTAAAAATAGGAGATAAAACCTCTTTATCCA   127
nad4   TTCTTC-TT-CA-TACTGCACCT-GCACT---------------------------------------------------------------------------------------------------    25
trnL1  --------------------------------------------------------------------------------------------------------------------------------     0

R      CCGAGAGAGGCTGGAAGCACTGGGGACTGCTAATCCCTCAGCACCACGGCTAAACTCCGTGGCTCACTCGGCCCCTAAAGGATAATAGCTCATCCGTTGGTCTTAGGAACCAAAAACTCTTGGTGCAA   255
nad4   --------------------------------------------------------------------------------------------------------------------------------    25
trnL1  ---------------------------------------------------------------------------TAAAGGAAAATAGCCCTTCCCCTGGTCTTAGGCTCCAGTATCTCTTGGTGCAA    53
```

```
                trnL1
R      CTCCAAGTAGAGGCT   270
nad4   ---------------    25
trnL1  -----A--------T    55
                trnL1
```

1.64 NC_011381-NC_011387

Avg ovsize: -61

LCA: Cyprinodontoidei-suborder

1.64.1 trnM-trnI -5

```
                                  trnM                                                                    trnI
R     AGCTAAATAAGCTTTTGGGCCCATACCCCAAACATGTTGGTTAAAATCCTTCCTTTACTAATGACGGAGATGTGCCTGAAATAAAAGGATCATTTTGATAGAATGCCTCATGAGGGTTTAAGT-C-   124
trnM  AGCTAAACAAGCTTTTGGGCCCATACCCCAAACATGTTGGTTAAAATCCTTCCTTTACTA------------------------------------------------------------------    60
trnI  -----------------------------------------------------------------GGAGCTGTGCCTGAA-TCAAAGGGCCACTTTGATAGAGTGAATCATGAGGGTTAGAATCCC    60
                                  trnM                                                                    trnI
```

1.64.2 trnQ-nad2 -69

```
                                  trnQ                                                                                           nad2
R     GGGTTTGAACCCCACCTGAAGAGATCAAAACTCTTAGTGCTTCCACTACACTACTTCCTAGTAAAGTCAGCTAAATAAGCTTTTGGGCCCATACCCTAAACATGTTGGTTAAAATCCTTCCTTTACTA   128
trnQ  GGACTTGAACCCTACCTGAAGAGATCAAAACTCTTAGTGCTTCCACTACACCACTTCCTA--------------------------------------------------------------------    60
nad2  --------------------------------------------------------------------------------------------------------------------------------     0
                                  trnQ
```

nad2

```
R     ATGAACCCCTACATTTTATTTATTTTACTTTTTGCTCTAGGCCTCGGGACAACCA-CTAC   187
trnQ  ------------------------------------------------------------   60
nad2  -TGAACCCTTTTATCCTTTCCACCCTTCTTCTAGGACTCGGCCTTGGAACAACCATCACC   59
```

nad2

1.64.3 nad1-trnM -110

nad1

```
R     CTTGTAATCTGACATCTCTCATTACCAATTGCCTTCTTTGGTCTTCCCCCCCAACTTTAATATTTACAGAGATATGCCTGAAATAAAGGATCCCCTTGATAGAGTGCCCCTTAGAATAAAAGAAGTTT   128
nad1  CTCGTCATTTGACACCTCTCCCTCCCAATCGCCTTATCTGGCCTGCCCCCTCAACTTTA---------------------------------------------------------------------   59
trnM  --------------------------------------------------------------------------------------------------------------------------------   0
```

nad1

trnM

```
R     ATAACCCCACCTGAGGGGATTAAAACTCTTAGTACTTCCTAGTAAAGTCAGCTAAATAAGCTTTTGGGCCCATACCCCAAACATGTTGGTTAAAATCCT-   227
nad1  ----------------------------------------------------------------------------------------------------   59
trnM  -----------------------------------------GTAAAGTCAGCTAAACAAGCTTTTGGGCCCATACCCCAAACATGTTGGTTAAAATCCTT   59
```

trnM

1.65 NC_003159-NC_009851

Avg ovsize: -62

LCA: Neoteleostei-subclass

1.65.1 trnN-trnY -33

```
                                                   trnN                                                              trnY
R     CCTCGATCCCCCCAAACTCTTAGTTAACAGCTAAGCGCCCCAACCAGCGAGCATCCATCTAACTGACCA--GCCTGGTAGGAAGAGGACTTAAACCTCTCTTTATGGGGCTACAACCCACCACTTAAA-   125
trnN  CCTCGATCCTGCAAACTCTTAGTTAACAGCTAAGCGCTCAAACCAGCGAGCATCCATCTACCTTTCCCCCGCCTGATAAAACTAGGA-----------------------------------------    87
trnY  ------------------------------------------------------------------------------------------------------------------------ACTTAGGC     8
                                                   trnN                                                                                   trnY
```

```
      trnY
R     TCTCA   130
trnN  -----    87
trnY  TAACA    13
      trnY
```

1.65.2 nad2-trnW -58

```
                                 nad2                                                              trnW
R     TCTATCGCTGCCCTTGGGTTGCTCCCTGTAACCCCAGCTATCTTAGCCTGATTCACCTAGGGGCTTAGGATAA--TA-AGACCAAGAGCCTTCAAAGCTCTAAGCGAGAGTGAAAATCCC   117
nad2  ------------------------------------------------------------------------------------------------------------------------     0
trnW  ----------------------------------------------------------AGAGACTTAGGCTAACATCTAGACCAAGGGCCTTCAAAGCCCTCAGTGGGAGTGAGAATC--    60
                                                                                          trnW
```

1.65.3 trnY-trnC -96

```
                              trnY
R     AGGACTTAAACCTCTCTTTATGGGGCTACAACCCACCACTTA-AATCTCAGCCACCCTACCTTATACCCTGTTACGCCCCTGAACCACCTCAACACATGCTCCCCCATAGATTAACCACACTTATCGC 127
trnY  -GGACTCGAACCTCTGTCTATGGGGCTACAATCCACCGCTTAAAAACTCAGCCATCCTACC------------------------------------------------------------------- 60
trnC  -------------------------------------------------------------------------------------------------------------------------------- 0
                              trnY
```

```
                                                                  trnC
R     CCACATAAGCAGGTCTCCTTTCGCCGCCGGGCGTCGACGGAAAGCCCCGGCAGGCTGGAAACCTGCTTCTTCAGATTTGCAATCTGACGTGT-CACACCTC 227
trnY  ----------------------------------------------------------------------------------------------------- 60
trnC  -----------------------------GGAGGCGGGGGAAAGCCCCGGCAAGCAGTTAGCTTGCGACTTCAGATTTGCAATCTGATATGTTTACACCTC 72
                                                                  trnC
```

1.66 NC_004373-NC_011381

Avg ovsize: -69

LCA: Holacanthopterygii-superorder

1.66.1 nad1-trnI 48

```
                              nad1                                                            trnI
R     TTTATTATTTGACACCTGGCCCTCGGAGTTGCCCTTACGGGTC-TGCCTCCTG-C--C--TA--CAGGGTGTTGTGCCTGAATTAA-AGGGCCGCTTTGATAGAGCGTAACATGGGGGTTAGATTCC 118
nad1  CTTGTAATCTGACATCTCTCATTACCAATTGCCTTCTTTGGTCTTCCCCCCCAACTTTAATATTTACAGAGATATGCCTGAAATAA-AGGATCCCCTTGATAGAGTGCCCCTT-------------- 112
trnI  -----------------------------------------------------------------ACGGAGATGTGCCTGAAATAAAAGGATCATTTTGATAGAATGCCTCATGAGGGTTTAAGTCC 62
                              nad1
                                                                                              trnI
```

1.66.2 trnM-trnF 36

```
         trnM                                                                    trnF
R     -GCTAAGACAAGCTTTTGGGCCCATACCCCAAACATGTAGGTTTAACTCCTTCTTTTGCTATGGTTGTCCGAG-TA-GC-TCAACTAAAGCATAACATTGA-AGTTGTTAAGATGGGACCTAGCCC-C   122
trnM  AGCTAA-ATAAGCTTTTGGGCCCATACCCCAAACATGTTGGTTAAAATCCTTCCTTTACTATATTTA-CAGAGATATGCCTGAAATAAAGGATCCCCTTGATAGA-GT--------------------   105
trnF  ------------------------------------------------------------------------G-TA-GC-TTAATTAAAGTATAACACTGA-AGATGTTAAGATGGGCCCTAAAAAGC    52
         trnM                                                                      trnF
```

```
      trnF
R     CC   124
trnM  --   105
trnF  CC    54
      trnF
```

1.66.3 trnP-trnM -292

```
         trnP
R     AG-ATTTTAACTCTCACTACTAGCCCCCAAAACTAATATTCTAACGGTAAACTACCCTCTGCTATATGCAACGTGACCGCTACAGTACCCACACACATACATGTACACATCTATTTATATAGATATAC   127
trnP  GGGAGTTTAACCCCCACCCCTAGCTCCCAAAGCTAGAGCTCTAA-GTTAAACTATTCTTTG-------------------------------------------------------------------    60
trnM  --------------------------------------------------------------------------------------------------------------------------------     0
         trnP
```

```
R     ATATATACTTATGCATTTATATATCTATACCTATGTAGATACGTATACCCACATCTGTATCTACAACCATTTAGGCGTTTCTACACCTGTGCCTGTGTCCACCTGTATATAACTATGTATGTACAGAT   255
trnP  --------------------------------------------------------------------------------------------------------------------------------    60
trnM  --------------------------------------------------------------------------------------------------------------------------------     0
```

```
                                                                                                                                        trnM
R     ATATATATATATATATATATATATATGCAGCTGCATACATGCACACCCCCACGCTATACAACACGCGCCCTATGAGAGTAAGTCGCTACAACCCCGCACACGGTAAAATCAGCTAAGACAAGCTTTTGGG   383
trnP  ----------------------------------------------------------------------------------------------------------------------------------    60
trnM  ---------------------------------------------------------------------------------------------------ACAGTAAAGTCAGCTAA-ATAAGCTTTTGGG    30
                                                                                                                                       trnM
```

```
                  trnM
R     CCCATACCCCAAACATGTAGGTTTAACTCC--   413
trnP  --------------------------------    60
trnM  CCCATACCCCAAACATGTTGGTTAAAATCCTT    62
                   trnM
```

1.67 NC_006286-NC_008778

Avg ovsize: -70

LCA: Episquamata-order

1.67.1 nad5-trnE -51

```
                                nad5                                                          trnE
R     GGACGAATAAAATCTTATTTATCATTATTCCTACTAACCATGTTTATCCTACTATTCTAGACTTGACAATTTCCGCCGGCAATTCTACCGGACCTGCGGTCTGAAAAACCGCCGTTGTG-TTCAACT-   126
nad5  ------AT--C-TCTTA-TTATATTTACTGC-ACTAACCACAATTACCCTA-----------------------------------------------------------------------------    40
trnE  ------------------------------------------------------------------------------------------------------GAAAAATCCACGTTGTTATTCAACTA    26
                              nad5                                                                                       trnE
```

1.67.2 nad6-cob -74

nad6

```
R     CGCAACACCAACAATAGCTATCAAAAATGCCCACC-CC-AA-AAGATA-A-GTAATATATATCATAGAGCTTACTACCACAACGGTAGCACATCAGAA-AATAAAATTCTGCTATCACTACAACTACA   122
nad6  ACCAAC--CAA-AAGA-CCA-CACACAACCAAACCACCTAACAACATACAAATAAAATAAGACATTCA-CTTT-TACT-CCACTG-AGCATGTAAGTTCAATAAACTCATG-TAACACTGTAA-----   113
cob   --------------------------------------------------------------------------------------------------------------------------------     0
```

nad6

cob

```
R     AGCACCAATAATGGCCCACATCACACGCAAGCATCATCCAATCTTTAAAATGGTCAACAGCACATTTAT   191
nad6  ---------------------------------------------------------------------   113
cob   ---------------------------------------------------------------------     0
```

1.67.3 trnT-trnP -86

trnT *trnP*

```
R     -AGCTTACCAGATAAAGCGCCGGCCTTGTAAGCCGGAAACGAGCGCTCCGC-TCCTCAGGCTAAAACAATCCAAGCCTTACCACATCAAAGGAAAGGCTTTAAGCGCGCCTCATCTCCCGCCCCCAAA   126
trnT  TAGCTTA--ATACAAAGCATTGGCCTTGTAAGCCGAAGATG-GCAATCAACCTCC-------------------------------------------------------------------------    52
trnP  --------------------------------------------------------------------------------------------------------------------------------     0
```

trnT

trnP

```
R     ACGGGCATTTTAAACT   142
trnT  ----------------    52
trnP  -------------ACC     3
```

trnP

1.68 NC_005796-NC_005800

Avg ovsize: -70
LCA: Albuliformes-order

1.68.1 trnL1-trnH 0

1.68.2 trnS1-nad5 -74

```
                                   trnS1
R      CTG-GAAGCACTGGGGACTGCTAATCCCTCAGCACCACGGCTAAACTCCGTGGCTCACTCGGCCCCTAAAGGATAATAGCTCATCCGTTGGTCTTAGGAACCAAAAACTCTTGGTGCAACTCCAAGTA   127
trnS1  CTATGAGGCGTTGGGAACTGCTAATTC-TTAGTATCATGGTTAGAATCCATGGCTCGCTCG-------------------------------------------------------------------    60
nad5   --------------------------------------------------------------------------------------------------------------------------------     0
                                   trnS1
```

```
                                      nad5
R      GAGGCTATGCACTCAACAACCCTCATCTTCAATTCAAGCCTCCT-AATTATCTTCGCGCTCCTTAT-   192
trnS1  -------------------------------------------------------------------    60
nad5   -------TGCATTTATCAACTTTAGGTTTTAATTCGAGT-TCCTTAACTGTATTTGTACTACTCCTG    59
                                      nad5
```

1.68.3 nad4-trnL1 -137

```
                                   nad4
R      ACTTAATTCCAGTATTACTTCTAGTACTGAAGCCCGAACTAATATGAGGCTGATGCATATGTAGATATAGTTTAAAAAAGACGTTAGATTGTGATTCTAAAAATAGGAGATAAAACCTCTTTATCCAC   128
nad4   ATCTGATTCCAGTAATGCTTTTGGCTTTAAAGCCTGAGTTAGTGTGGGGTTGGTGTCTAT--------------------------------------------------------------------    60
trnL1  --------------------------------------------------------------------------------------------------------------------------------     0
                                   nad4
```

```
                                                                                                                                   trnL1
R      CGAGAGAGGCTGGAAGCACTGGGGACTGCTAATCCCTCAGCACCACGGCTAAACTCCGTGGCTCACTCGGCCCCTAAAGGATAATTAGCTCATCCGTTGGTCTTAGGAACCAAAAACTCTTGGTGCAAC   256
nad4   ------------------------------------------------------------------------------------------------------------------------------   60
trnL1  ----------------------------------------------------------------------GCCCCTATAGGATAACCAGCTGATCCGTTGGTCTTAGGAACCAAAGACTCTTGGTGCAA-   58
                                                                                                                     trnL1
```

```
           trnL1
R      TCCAAGTAGAGGCT   270
nad4   --------------   60
trnL1  ---A--T-------   60
        trnL1
```

1.69 NC_005439-NC_012434

Avg ovsize: -72

LCA: Panpulmonata-superfamily

1.69.1 cox2-trnY -61

```
                          cox2                                                trnY
R      ---T-TT--ATACCTATTGTAGTAGAAGCTGTTCACGTC-GATGACTTTTTTTTTGAAAGTGCTTAGCACAAAGGATTGTAAATCTTTCATTTGATATTCTATCTCAAAA   103
cox2   CACTCTTTCATGCCAATTGTTGTAGAAACGGC-CAGGTTGGAAGA-TTT------------------------------------------------------------   47
trnY   --------------------------------------------------------------------------------------------------------------   0
                        cox2
```

1.69.2 nad1-nad4l -75

```
R      -ATATTGTTCTTGTGTTTATTGTGTTGATTAATTATAATATATTTTAATTATATTTTTTAATAATTTTTTATGTTTCACTATTGTTTGTCTTTTTTTTGGTTCTATGTTACTTATTTAA  118
nad1   GGTTTTCTGCCTTT-TGGACTGTGT----------------------------------------------------------------------------------------------   24
nad4l  ----------------------------------------------------------------------------------------------------TTCTTTTTTACGTACATA-   18
```

1.69.3 nad4l-cob -80

```
R      CG-AG-G-AAGTCACATATTAATTGCGCTTACGCATTTTGCTAAAAATTCGTGATTCAGTTAATTTACCTAGACCTTTAAGTATTTCTATTTG  90
nad4l  AGTATAGTAAGTC--------------------------------------------------------------------------------  13
cob    ---------------------------------------------------------------------------------------------   0
```

1.70 NC_006340-NC_003159

Avg ovsize: -79

LCA: Teleostomi-superclass

1.70.1 trnN-trnC -25

```
                                 trnN                                                    trnC
R     GGATTTTATCCTACTACAAT-TTAGTTAACAGCTAAACACCAGATTTCAGGCTTTAATCTACTTATGTAAGCCCTGGCA-GAT--ACACTCTGCTCCTTAAAATTTGCAATTTCATGTGCTACTACAC  124
trnN  -CCT-CGATCCCCC-AAACTCTTAGTTAACAGCTAAGCGCC---------------------------------------------------------------------------------------  38
trnC  ------------------------------------------------------------------GAAAGCCCCGGCAGGCTGGAAAC-CTGCTTCTTCAGATTTGCAATCTGACGTGTCAC-AC-C  59
                        trnN                                                                               trnC
```

```
      trnC
R     T-A  126
trnN  ---   38
trnC  TCA   62
      trnC
```

1.70.2 trnY-trnN -61

```
                                  trnY
R     GGAAAGGGACCACCTTTATAAATGAGGCTACAACTCAACACCTAT-T-TCGGCCACCTTACCTTACA--CTGATATGCACAGCATTTTACCTAGTATGCGCGTAGCTACAAATTGTAGGCACGTCTTA  124
trnY  AGGACTTAA--ACCTCTCTTTATGGGGCTACAACCCACCACTTAAATCTCAGCCACCCTACCTTATACCCTGTTACGCCC------------------------------------------------  78
trnN  --------------------------------------------------------------------------------------------------------------------------------  0
                                  trnY
```

```
                                           trnN
R     CCATACTCTTCCCTAGATTAACAGGATTTTATCCTACTACAAT-TTAGTTAACAGCTAAACACCAGATTTCA--  195
trnY  --------------------------------------------------------------------------  78
trnN  -------------TAGATGAGAGGGCCTCGATCCCCCAA-ACTCTTAGTTAACAGCTAAGCGCCCCAAC-CAGC  59
                                          trnN
```

1.70.3 trnA-trnY -152

```
R     CGAGACTCTACCCCACATCTTCTGAATGCAACCCAGACACTTTAATTAAGCTAAAGCCTTCACAAACTACACCTCATATTCTTGTAATTTAATAAACAACTAATATTTATTTTTAGGCTATCTACTTC  128
trnA  CAGGACTTTATCCCACATCTTCTGAATGCAACCCAGAAACTTTTATTAAGCTAAGGCCCT--------------------------------------------------------------------  60
trnY  --------------------------------------------------------------------------------------------------------------------------------  0
```

```
R     TCCCGCTGTCTGGGGGGGAAGCGGGAGAAGCCCCGGAGAAGTCATGGGGCTCCTCGGCTTACGGGAAGTGTAAGCTTTATTACACTGGTAAGGAAAGGGACC-A--CCTTTATAAATGAGGCTACAAC  253
trnA  --------------------------------------------------------------------------------------------------------------------------------  60
trnY  ------------------------------------------------------------------------------------CTGGTA-GGAAGAGGACTTAAACCTCTCTTTATGGGGCTACAAC  43
```

```
R     TCAACACCTATTTCGGCCA  272
trnA  -------------------  60
trnY  CCACCACTTAAATCT-CAG  61
```

1.71 NC_014174-NC_007978

Avg ovsize: -79

LCA: Sauropsida-subclass

1.71.1 nad5-nad6 3

```
                              nad5                                                  nad6
R     TACCTATCAACATTTGTAATTACATCAATACTAGCCATAATTATTCAACTATATTACTAACTC-CGAACCGACCCAAAACATCAACC-ACACACAAGATCTAACACTACAAACAATGT   116
nad5  -ATC-AA-AACCTACTTAGGTTCATTCGCCCTATCCATCCTCA-TCACC-CTATTA--------------------------------------------------------------   51
nad6  -----------------------------------------------------TTAA-ACCGCTCGAATAGCCCCACAAGA-CAACCCACGCACCAACTCCAACACAACAAACAA---   60
                              nad5
                                                                               nad6
```

1.71.2 trnE-cob -8

```
                              trnE                                                   cob
R     TATCTAGACAACTAGAACCCCCAATACGAAAAATTGATGTTGT-TATT-CAACTATAAAAACATGACCATCCTACGAAAATTAAACCCAATCCTAAAAACT-ATTAACCACTCCCTAATTGA   119
trnE  G-GCTTTTCTCCAAG-ACTCGCGGCCTGAAAAGCCGCTGTTGTCAATTTCAACTACGAAAAC------------------------------------------------------------   60
cob   ----------------------------------------------------------------------CCTCCGAAAATCTCATCCACTCCTAAA-ACTCATTAACAACTCCTTAA-TC-   49
                              trnE
                                                                                      cob
```

1.71.3 trnP-trnF -234

```
                              trnP
R     AAGAGGTT-AACAACCATCTCTAACTCCCAAAGCTAGAATTTTATCTTAAACTAT-CTCTTAAACATATAAATTATACACACACCAAAAAATGTCAATCCCAAACAAAAATAAAATAATTTGGTCCAG   126
trnP  A-GGGCTTAAACCTCTATCTCCAGCTCCCAAAGCTGGTATTTTACACTAAACTATTCTCTGA------------------------------------------------------------------   61
trnF  --------------------------------------------------------------------------------------------------------------------------------   0
                              trnP
```

```
R     GGTTAACTCTAATTGTATTTATTTTTTTACGATATATATATATATATATATATATATATATATATATATATATATATAGTATATAGAGATATATTGTGTGGCCCAGGGTTAAACTCTAATTTTATATTTATATATGTG   254
trnP  --------------------------------------------------------------------------------------------------------------------------------------   61
trnF  --------------------------------------------------------------------------------------------------------------------------------------   0
```

```
                                                    trnF
R     TATCGGTCCAGGACTTGCCGTACAATTATTATAAAAGTTATTGTAGCTTAACATTAA-GCACAGTATTGAAAATACTGAAATGAGTGTAAACACTC-   349
trnP  -------------------------------------------------------------------------------------------------   61
trnF  ----------------------------------------TTGTAGCTTACTACCAAAGCATGGCACTGAAGATGCCAAGACGGCTGCCATT-CTCG   56
                                                    trnF
```

1.72 NC_006340-NC_006333

Avg ovsize: -87

LCA: Batrachoseps-genus

1.72.1 trnC-cox1 -34

```
                        trnC                                                                        cox1
R     -TGGCAGATACACTCTGCTCCTTAAAATTTGCAATTTCATGTGCTACTACACTACAGGCCTACAAATATAAGCTTGTATGCCTTACCTGTGATAATTACTCGATGACTATTTTCAACAAACCATAAAG   127
trnC  GTAGAAG-T-CATTCTGCTCTTTAAAATTTGCAATTTTATGTGCT-GTACACCAC-------------------------------------------------------------------------   52
cox1  -----------------------------------------------------------------------------------------TGATAGTTACGCGATGACTTTTTTCAACAAACCACAAAG   39
                        trnC                                                                         cox1
```

```
        cox1
R     ATATCGGCACCTTATATTT-   146
trnC  --------------------   52
cox1  ATATCGGCACCCTTTACCTA   59
        cox1
```

1.72.2 trnY-trnN -75

```
                               trnY
R     GGAAAGGGAC--CACCTTTATAAATGAGGCTACAACTCAACACCTATTTCGGCCACCTTACCTTACACTGATATGCACAGCATTTTACCTAGTATGCGCGTAGCTACAAATTGTAGGCACGTCTTACC   126
trnY  A-AAAGG-ACTACACCTTTATAATTGAGGCTACAACTCAGCACCTACTTCGGCCATCTTACCT-----------------------------------------------------------------    61
trnN  --------------------------------------------------------------------------------------------------------------------------------     0
                               trnY
```

```
                                    trnN
R     ATACTCTTCCCTAGATTAACAGGATTTTATCCTACTACAATTTAGTTAACAGCTAAACACCAGATTTCA-   195
trnY  ----------------------------------------------------------------------    61
trnN  ----------CTAGATTAGTAGGCTTTTATCCTACTACCTTTTAGTTAACAGCTAAATACCTAATATCAG    60
                                    trnN
```

1.72.3 trnA-trnY -153

```
                              trnA
R     CGAGACTCTACCCCACATCTTCTGAATGCAACCCAGACACTTTAATTAAGCTAAAGCCTTCACAAACTACACCTCATATTCTTGTAATTTAATAAACAACTAATATTTATTTTTAGGCTATCTACTTC   128
trnA  CAAGACTTTATTCCACATCTTCTGAATGCAACCCAGACACTTTAATTAAGCTAAAACCCTC-------------------------------------------------------------------    61
trnY  --------------------------------------------------------------------------------------------------------------------------------     0
                              trnA
```

```
                                                                                                                trnY
R     TCCCGCTGTCTGGGGGGGAAGCGGGAGAAGCCCCGGAGAAGTCATGGGGCTCCTCGGCTTACGGGAAGTGTAAGCTTTATTACACTGGTAAGGAAAGGGAC--CACCTTTATAAATGAGGCTACAACT   254
trnA  --------------------------------------------------------------------------------------------------------------------------------    61
trnY  --------------------------------------------------------------------------------------GGCAA-GAAAAGGACTACACCTTTATAATTGAGGCTACAACT    41
                                                                                                                trnY
```

```
       trnY
R      CAACACCTATTTCGGCCA   272
trnA   ------------------    61
trnY   CAGCACCTACTTCGGCCA    59
       trnY
```

1.73 NC_004373-NC_006355

Avg ovsize: -101

LCA: Holacanthopterygii-superorder

1.73.1 trnI-trnQ 4

```
       trnI                                                          trnQ
R      CTGAATTAAAGGGCCGCTTTGATAGAGCGTAACATGGGGGTTAGATTCCCCCCAACTCCTTAGAAAGGGAGGGCTCGAACCTCCCCCGGGGAGATCAAAACTCCCAGTGCTCCCACTA-   118
trnI   CTGAATCAAAGGGTCACTTTGATAGAGTGAAGCATGGGGGTTAAAATCCTCCCAACTCCTT----------------------------------------------------------    61
trnQ   ----------------------------------------------------------CCATAGAAAAAAGGGGCTCGAACCCTTCCTGAAGAGATCAAAACTCTTGGTGCTTCCACTAC    62
       trnI
                                                                     trnQ
```

1.73.2 trnM-trnF -10

trnM *trnF*

```
R     GCTAAGACAAGCTTTTGGGCCCATACCCCAAACATGTAGGTTTA-ACTCCTTCTTTTGCTATGGTTGTCCGAGTAGCTCAACTAAAGCATAACATTGAAGTTGTTAAGATGGGACCT-AGCCCCCC  124
trnM  GCTAATAGAAGCTATTGGGCCCATACCCCAAAAATGTTGACTAATACTCAGCC-TTTGTTAT----------------------------------------------------------------  61
trnF  ------------------------------------------------------------------------GTAGCTTAACAAAAGCATAACACTGAAGATGTTAAGATGGACCCTTAAAAGTCC  54
```

trnM

trnF

1.73.3 trnP-trnM -297

trnP

```
R     AGATTTTAACTCTC-ACTACTAGCCCCCAAAACTAATATTCTAACGGT-AAACTACCCTCTGCTATATGCAACGTGACCGCTACAGTACCCACACACATACATGTACACATCTATTTATATAGATATA  126
trnP  -GATTTAAAC-CTCCACCTCTAGCTCCCAAAGCTAGTATCCTGTCAGTTAAACGATTCTCTG------------------------------------------------------------------  60
trnM  --------------------------------------------------------------------------------------------------------------------------------  0
```

trnP

```
R     CATATATACTTATGCATTTATATATCTATACCTATGTAGATACGTATACCCACATCTGTATCTACAACCATTTAGGCGTTTCTACACCTGTGCCTGTGTCCACCTGTATATAACTATGTATGTACAGA  254
trnP  --------------------------------------------------------------------------------------------------------------------------------  60
trnM  --------------------------------------------------------------------------------------------------------------------------------  0
```

trnM

```
R     TATATATATATATATATATATATATGCAGCTGCATACATGCACACCCCCACGCTATACAACACGCGCCCTATGAGAGTAAGTCGCTACAACCCCGCACACGGTAAAATCAGCTAAGACAAGCTTTTGG  382
trnP  --------------------------------------------------------------------------------------------------------------------------------  60
trnM  -------------------------------------------------------------------------------------------------------AAAGTAAGCTAATAGAAGCTATTGG  25
```

trnM

```
R      GCCCATACCCCAAACATGTAGGTTTA-ACTCC   413
trnP   --------------------------------    60
trnM   GCCCATACCCCAAAAATGTTGACTAATACTCA    57
```

trnM (above), *trnM* (below)

1.74 NC_008123-NC_004373

Avg ovsize: -105

LCA: Ophidioidei-suborder

1.74.1 trnQ-trnM -54

```
R      GGATTCGAACCCATCCTATAGAGATCAAAACTCTATGTGCTCCCAATACACCACCTTCTAGC-GGAATAAGCTAATTAAGCTGTCGGGCCCATACCCCGGATACGTAGGTTAAAGCCCT   118
trnQ   GGGCTCGAACCTCCCCCGGGGAGATCAAAACTCCCAGTGCTCCCACTACACCACTTTCTAGCAGG------------------------------------------------------    65
trnM   -----------------------------------------------------------------------------------------------------------------------     0
```

trnQ, *trnM* (above), *trnQ* (below)

1.74.2 trnM-nad2 -89

```
R      AGCTAATTAAGCTGTCGGGCCCATACCCCGGATACGTAGGTTAAAGCCCTACTTCCGTTGGTGAACGTTCAGATGATATTCATTTTACTATCTTCTTTAATACTGGGCACAACCATTAC-   119
trnM   AG-GAGTTAAAC------------------------------------------------------------------------------------------------------------    11
nad2   -----------------------------------------------------------------------------------------------------ACTCGGCACTACCATTACC    19
```

trnM, *nad2* (above), *trnM*, *nad2* (below)

1.74.3 trnP-trnF -173

```
                                      trnP
R     GAGAATCTAACTCCCACCACTAACTCCCAAAGCTAGTATTCTTAAC--TAAAATACTTTTTGACGGTATCTAATGT-ACA--TACA-TCGATATTAACCCATATATATACATATATGTATAATAACCA   122
trnP  -AGATTTTAACTCTCACTACTAGCCCCCAAAACTAATATTC-TAACGGTAAACTACCCTCTG-CTATATGCAACGTGACCGCTACAGTACCCACACACATACATGTACACATCTATTTAT-ATAGATA   124
trnF  ------------------------------------------------------------------------------------------------------------------------------------   0
                                      trnP

R     TACATCTATATTAACCCATATATATA-C-ATA--TATGT--ATA-ATA-ACCATACATCTATAT-TA-ACCCATATA----TAT--ACATATATGTATAATAACCATACATCTATATTAACCCATATA   234
trnP  TACATATATACTTATGCATTTATATATCTATACCTATGTAGATACGTATACCC-ACATCTGTATCTACAACCATTTAGGCGTTTCTACACCTGTGCCT-GTGTCCA-CC-TGTATAT-AA-CTATGTA   246
trnF  ------------------------------------------------------------------------------------------------------------------------------------   0

R     TATACATATATGTATAATAACCATACATCTATATTAACCCATATATATACATATATGTATAATAACCATACATCTATATTAACCCATATATATACATATATGTATAATAACCATACATCTATATTAAC   362
trnP  TGTACAGATATATAT-ATATATATATATATATATGCAGCTGCATACATGCACA-------------------------------------------------------------------------------   298
trnF  ------------------------------------------------------------------------------------------------------------------------------------   0

                                                                                                                                  trnF
R     CCATATATATACATATATGTATAATAACCATACATCTATATTAACCCATATATATACATATATGTATAATAACCATACAGTCTATATTAACCATCAACGTAGCTTAAATAAAGCGCAACACTGAAGTT   490
trnP  ------------------------------------------------------------------------------------------------------------------------------------   298
trnF  ------------------------------------------------------------------------------------------------------GTAGCTCAACTAAAGCATAACATTGAAGTT   30
                                                                                                                          trnF

            trnF
R     GTTAAGATGGGCCCTAGAAAGC-T   513
trnP  ------------------------   298
trnF  GTTAAGATGGGACCTAGCCCCCCC    54
            trnF
```

1.75 NC_012434-NC_005439

Avg ovsize: -105

LCA: Panpulmonata-superfamily

1.75.1 nad1-cob -91

```
R     GGTTTTCTGCCT-TTTGGACTGTGTGCCGTATTACTATCAGCCAGTTCCGCTGTTTTGTAGCTTGCGTAAAATTCGACTCGTGGAAAGGATGGCTGGCTTGCCTAGCCCCATGAATTTTTC   120
nad1  A-TATTGTTCTTGTGTTTATTGTGT------------------------------------------------------------------------------------------------    24
cob   --------------------------------------------------------------------------------------------------------------------TTTGA     5
```

1.75.2 nad4l-trnY -104

```
R      AGTATAGTAAGTCTGAGGGGCAGAGACACTGTCAAAGCAATCCCCTGACGCGGCTGGTAAGATTTTGGTGGCTGAAATGAAACAGGCAGCTGGTTGTAAACCAGAGTATGGACGTAGAT   119
nad4l  CG-AG-G-AAGTC----------------------------------------------------------------------------------------------------------    10
trnY   ---------------------------------------------------------------------------------------------------------------------AT     2
```

1.75.3 cox2-nad4l -122

```
                                        cox2
R      CACTCTTTCATGCCAATTGTTGTAGAAACGGC-CAGGTTGGAAGA-TTTACTCTCCCTCTAAACCAAAATATATATGGATATAGACACAGTATTATTAATTCTAGAGGGAACAATGTGGTTAATCGTT   126
cox2   -----TTT-ATACCTATTGTAGTAGAAGCTGTTCACGTCG-ATGACTTT-------------------------------------------------------------------------------    42
nad4l  --------------------------------------------------------------------------------------------------------------------------------     0
                                    cox2
```

```
                                            nad4l
R      GACGTGGTAGATTTTTGACAGTACCCCTGGTCGTAGCATTAACTTTGCTGGTACTCTTCTTTTTTACGTACAT   199
cox2   -------------------------------------------------------------------------    42
nad4l  -------------------------------------------TTTTTTGGTTCTAT-GTTACTTATTTA-AT    28
                                                          nad4l
```

1.76 NC_006340-NC_009851

Avg ovsize: -115

LCA: Teleostomi-superclass

1.76.1 nad2-trnW -61

```
                                  nad2                                                          trnW
R      ACAACTATTATATCAATTATATTATTACCTATTACCCCCACAGTCCTTAATTTATTTTAAGGGTTTAGGATAA--TTTAAACCAAGGACCTTCAAAGCCCTAAACAGAAGTTAAAAACT   117
nad2   -CA--------------------------------------------------------------------------------------------------------------------     2
trnW   ----------------------------------------------------------------TTAGGCTAACATCTAGACCAAGGGCCTTCAAAGCCCTCAGTGGGAGTGAGAATC-    54
        nad2
                                                                                                trnW
```

1.76.2 trnY-trnN -76

```
                                        trnY
R     GGAAAGGGACCACCTTTATAAATGAGGCTACAACTCAACACCTA-TT--TCGGCCACCTTACCTTACACTGATATGCACAGCATTTTACCTAGTATGCGCGTAGCTACAAATTGTAGGCACGTCTTAC   125
trnY  GGACTCGAACCT-CTGTCT--ATGGGGCTACAATCCACCGCTTAAAAACTCAGCCATCCTACC-----------------------------------------------------------------   60
trnN  --------------------------------------------------------------------------------------------------------------------------------   0
                                        trnY

                                        trnN
R     CATACTCTTCCCTAGATTAACAGGATTTTATCCTACTACAAT-TTAGTTAACAGCTAAACAC-CAGATTTCA   195
trnY  ------------------------------------------------------------------------   60
trnN  -----------CTAGATAGGCAGGCCTCGATCCTGC-AAACTCTTAGTTAACAGCTAAGCGCTCAAACCAGC   60
                                        trnN
```

1.76.3 trnA-trnY -210

```
                                        trnA
R     CGAGACTCTACCCCACATCTTCTGAATGCAACCCAGACACTTTAATTAAGCTAAAGCCTTCACAAACTACACCTCATATTCTTGTAATTTAATAAACAACTAATATTTATTTTTAGGCTATCTACTTC   128
trnA  CGGGACATTACCCCACATCTCCTGCATGCAAAACAGACACTTTAATTAAGCTAAAGCCTT--------------------------------------------------------------------   60
trnY  --------------------------------------------------------------------------------------------------------------------------------   0
                                        trnA

                                                                                                                 trnY
R     TCCCGCTGTCTGGGGGGGAAGCGGGAGAAGCCCCGGAGAAGTCATGGGGCTCCTCGGCTTACGGGAAGTGTAAGCTTTATTACACTGGTAAGGAAAGGGACCACCTTTATAAATGAGGCTACAACTCA   256
trnA  --------------------------------------------------------------------------------------------------------------------------------   60
trnY  --------------------------------------------------------------------------------------------------------------------------------   0
```

trnY

```
R      ACACCTATTTCGGCCA   272
trnA   ----------------    60
trnY   --------------CA     2
```

trnY

1.77 NC_006345-NC_006344

Avg ovsize: -148
LCA: Plethodontinae-subfamily

1.77.1 trnW-trnN -58

trnW / *trnN*

```
R      G-TTA-TTAGACTAAGGACCTTCAAAGCCCTAAGCAGAAGTTAAAATCCTCTAATCTTTGTTAGAATAAAAATTTTACATCTACAAATATAGTATATCTCTTATGAACTAAAACCTTACTAGATTAGT   126
trnW   GCTAAATTAGACTAAGGACCTTCAAAGCCCAAAGCAGAAGTTAAAATCTTCTAATCTTTG--------------------------------------------------------------------    60
trnN   ----------------------------------------------------------------------------------------------------------------------CTAGACTAGC    10
```

trnW

trnN

trnN

```
R      AGGCTTTTACCCTACGATCTTTTAGTTAACAACTAAATACCCACATC-TG   175
trnW   --------------------------------------------------    60
trnN   AGGTTTTTATCCTACAACCTTTTAGTTAACAGCTAAACACCCAAATCAAT    60
```

trnN

1.77.2 trnY-trnA -187

```
trnY
R     AAAAGGA-TTAAACCTTTATAAATGAGACTACAGCTCACCACCTATT-TCGGTCACCTTATCTATAGTATTACCCGATAGCTCTTCTCAACAAATAAAATATTGTACCCTTATTTAGTATTGTACCTA   126
trnY  A--AGGACTTAAACCTTTATGATCGAGGCTACAACTCGACACCTTTTTCGGACACCTTACCT-----------------------------------------------------------------    61
trnA  --------------------------------------------------------------------------------------------------------------------------------     0
trnY

                                                                                                                              trnA
R     ACCTGTAATCGCATTATCTATCTTAATTTAGACTATCAGAAATGATTAAGTTTATATGATACACCATTTAAAATTATTCAATTAACCTTTAAAATCCTAAATAAAGATAAATTTAATCTTTGTAAGAC   254
trnY  --------------------------------------------------------------------------------------------------------------------------------    61
trnA  --------------------------------------------------------------------------------------------------------------------------TAAGAC     6
                                                                                                                              trnA

trnA
R     CTGTAAGACTTTATCTTACATCTTATGAATGCAACCCAGACACTTTAATTAAG-   307
trnY  ------------------------------------------------------    61
trnA  TCGCAAGATTCTATCTAACATCCTTTGAATGCAACCCAAATACTTTAATTAAGC    60
trnA
```

1.77.3 trnA-cox1 -201

```
trnA
R     TAAGACTTTATCTTACATCTTATGAATGCAACCCAGACACTTTAATTAAGCTA-AGACCTTACTTGATTGGCAGACCCTTACCCCATAATATTTTAATTAACTATACCCACATCTGGCCTTAATCTAC   127
trnA  CAAGATTCTATCTAACATCCTTTGAATGCAACCCAAATACTTTAATTAAGCTAGAG-CCTTA------------------------------------------------------------------    61
cox1  --------------------------------------------------------------------------------------------------------------------------------     0
trnA
```

```
R     TTCTCCCGCTTTGTGGGGGGGGAAAAGCGGGAGAAGCCCCGGTAGAGAATTATCTGCTCTGAAATTTGTAACTCATATGCTTACACCACAGGACTTAGTAAGAAGGATAACCTTTATAATACAACACC   255
trnA  --------------------------------------------------------------------------------------------------------------------------------    61
cox1  --------------------------------------------------------------------------------------------------------------------------------     0
```

cox1

```
R     TTACCTATGATAATTACCCGATGATTCTTCTCAACAAATCATAAAGATATTGGTACCCTATATTT-   320
trnA  ------------------------------------------------------------------    61
cox1  -------TGATAATCACTCGATGACTATATTCAACAAATCATAAAGATATTGGCACCCTTTATTTA    59
```

cox1

1.78 NC_001673-NC_006321

Avg ovsize: -148
LCA: Annelida-phylum

1.78.1 trnK-nad3 2

trnK *nad3*

```
R     -AGAGAGCTTGTAAATAGCACTTGACTTTTAATCAAGAGATAG-TATAATTATT-TCTAGTTAATGATCCTTACAGCCTTATCCTCAGCCATTGCACTATTAGTCCCTATTA-TTATTTTGGG   119
trnK  GAGAAAGCTTGCTAA--GCATTAGTCTTTTAAACTA-AAAAAGATCTCTTTGGTCTCT-----------------------------------------------------------------    55
nad3  --------------------------------------------------------CTA-TTA-TTAT--TTACA-T-TTATACTCAGAC-TAG---TAGTA-TCCTTCTTACTTATCCTT--    54
```

trnK

nad3

1.78.2 trnI-trnK -13

```
                          trnI                                                   trnK
R     AGCGCCGG-ATGAACGGATAACTCTGATGACGTTAATTAAGGAACAAGCTTCCCTGTATCTAACTAGAGAGCTTGTAAATAGCACTTGACTTTTAATC-AAGAGATAGTATAATT-AT-T-TCT   119
trnI  CGC-CGGGCATGAACGGACAGCTTTGATGTAGCTGAT-AACGAAGTA-CTTC------------------------------------------------------------------------   49
trnK  -----------------------------------------------------------------AGAAAGCTTGC-TA-AGCATTAGTCTTTTAAACTAAAAAGA-TCTCTTTGGTCTCTTT   56
                      trnI                                                               trnK
```

1.78.3 trnR-trnH -435

```
                          trnR
R     TAAGCA-TTAAAAAAAATGCGCGCCGATTTCGACTCGGCGAGAGCACAAAGCATTGTTTTTTTTACTTAGTTTATACTATACTCTATATATATATACGCATTTGTGTACTCTGATTGGGGGGGGGGGGT   127
trnR  GTGGCAATTTA---------------------------------------------------------------------------------------------------------------------   11
trnH  --------------------------------------------------------------------------------------------------------------------------------   0
        trnR

R     AATTTCACAAAAAGCTATAATCCGAAAAGGCCCGACCGGGCGAGAAAAAAAAAAAAAAAAAAAGAAAAAGTGGTGTTTTTAGGTTCTAATCCTTTAGAATGATGCCAATTTCGGAAAAACTCGACAGG   255
trnR  --------------------------------------------------------------------------------------------------------------------------------   11
trnH  --------------------------------------------------------------------------------------------------------------------------------   0

R     GACTTTTTAAATTTCGGTCCTTGCTAATATGGGCACGACGTATATTTGCGGTATTTACATAAGAAACGGCCTGTATCGAGCAAAATTTACAGTCTGTCGGGGGAAAAAATTTTAACCTAAAAAATTGT   383
trnR  --------------------------------------------------------------------------------------------------------------------------------   11
trnH  --------------------------------------------------------------------------------------------------------------------------------   0
```

trnH

```
R     TCGGCGTGGGGCCTTTTTTTTTTCAGTTTTTAACATTAAAAATTTTCTCGGAGTTCTAATCATAAAGGTAGGTTACAAAAACCCCCGAATTGTGGTTCCGGAAACGTCAAAAGAC-CCTTT   503
trnR  -------------------------------------------------------------------------------------------------------------------------   11
trnH  --------------------------------------------------------------TAAA-ATAGTTTAAAAAAAACCCTGTGTTGTGACCACAAAATTGTATACCTATTACTTT   58
```

trnH

1.79 NC_011128-NC_013257

Avg ovsize: -149

LCA: Endopterygota-infraclass

1.79.1 trnM-trnI 1

trnM *trnI*

```
R     -CTAAATTTAAGCTTTTGGGCTCATACCTCAAATATAAAG-T-AGATAATTCTTTTTTTTAATAAAGTGCCTGATTAAAGGATTATTCTGATAGGATAAATTAAGTAAAT-ATTTACCTT   116
trnM  GCTAAA-ATAAGCTATTGGGTTCATACCCCACTTATAAAGGTCCGACCCCTTTTCTTTTTA-----------------------------------------------------------   60
trnI  ------------------------------------------------------------AATGAAGTGCCTGACTAAAGGATTATTTTGATAGAATAAATCATGTATTTTATATACCTT   60
```

trnM

trnI

1.79.2 trnQ-nad2 -131

trnQ

```
R     ATAGAATTAAACTATAT-CTAAAAGTATCAAAAACTATTGTGCTTATTACACTAAAATATATTATTAATAAAAATATAAATTAATTATTTAGATTAAATTCTAATAAAAATTATTAAATTATAATTAT   127
trnQ  ATAGAATTAAACTATTTCCTAAAA-TATCAAAAATTTTTGTACATCATATACTAAAATGTA-------------------------------------------------------------------   60
nad2  --------------------------------------------------------------------------------------------------------------------------------   0
```

trnQ

```
                                     nad2
R     TTTTAATTTACAACTTAACTAACCCCCCTAAAATATTTTTTTTTTTTATTTTAATTTTTAGAAC  191
trnQ  ----------------------------------------------------------------  60
nad2  ----------------------------------------------------------------  0
```

1.79.3 rrnS-trnM -319

```
                                 rrnS
R     TA-AAATTTA-T-ATGTAAAATAAATTTTATAATAAATTCCCTAAATCATAAAATTTTCTTTAATAATAAATTAACTAAATAGATTTTTTTTTTTTTTTTTTTTATATTAAAATATTTAATAATCAA  125
rrnS  CACAAAAATATTCATGTAAAATTAAAATTA-AATAAATT---TATAGCC-AAAATCAAACTTTAA---------------------------------------------------------------  60
trnM  --------------------------------------------------------------------------------------------------------------------------------  0
                                 rrnS

R     TAATAAATTTTTAATAATTTCTTTTCCTTTCTTTTTATAATATTAATATTAAATACATAATTGCATATTAAATTTTTATAATTCATATATTTTATATTATATTTATATACCATTATATATTTGTTAAT  253
rrnS  --------------------------------------------------------------------------------------------------------------------------------  60
trnM  --------------------------------------------------------------------------------------------------------------------------------  0

R     AAATTAATAAATTATTAATATTAATAATAAATTAAATATTTAATATATATATATATTATATAAAGATAAATAAAACACTTAAATGTTTTATTTACCATTTTTAATAATATTTACATAAAAAAAAAATT  381
rrnS  --------------------------------------------------------------------------------------------------------------------------------  60
trnM  --------------------------------------------------------------------------------------------------------------------------------  0

                                 trnM
R     AAAA-ATAAGCTAAATTTAAGCTTTTGGGCTCATACCTCAAATATAAAG-T-AGATAATT  438
rrnS  ------------------------------------------------------------  60
trnM  AAAAGATAAGCTAAAAT-AAGCTATTGGGTTCATACCCCACTTATAAAGGTCCGACCCCT  59
                                 trnM
```

1.80 NC_006345-NC_013571

Avg ovsize: -163
LCA: Tetrapoda-superclass

1.80.1 trnW-trnN -79

```
                         trnW                                                                                    trnN
R     --GT-TATT-AGACTAAGGACCTTCAAAGCCCTAAGCAGAAGTTAAAATCCTCTAATCTTTGTTAGAATAAAAATTTTACATCTACAAATATAGTATATCTCTTATGAACTAAAACCTTACTAGATTA   124
trnW  TAGGATAATCAGTCCAAGAGCCTTCAAAGCCCTAAGTAGA----------------------------------------------------------------------------------------   40
trnN  -----------------------------------------------------------------------------------------------------------------------ACTAGATTG   9
                    trnW                                                                                                     trnN
```

```
                    trnN
R     GTAGGCTTTTACCCTACGATCTTTTAGTTAACAACTAAATACCCACATCT-G   175
trnW  ----------------------------------------------------   40
trnN  ATAGGATTCAAACCTATAAATTTTTAGTTAACAGCTAAACACCCTAAACTGG   61
                    trnN
```

1.80.2 trnA-cox1 -200

```
                           trnA
R     TAAGACTTTATCTTACATCTTATGAATGCAACCCAGACACTTTAATTAAGCTAAGACCTTACTTGATTGGCAGACCCTTACCCCATAATATTTTAATTAACTATACCCACATCTGGCCTTAATCTACT   128
trnA  CAAGACTACATCTTACATCTAGTGAGTGCAAATCAATTGCTTTAATTAAGCTAAATCCTCA-------------------------------------------------------------------   61
cox1  --------------------------------------------------------------------------------------------------------------------------------   0
                           trnA
```

cox1 (annotation spanning the preceding block is not shown on this line)

```
R     TCTCCCGCTTTGTGGGGGGGGAAAAGCGGGAGAAGCCCCGGTAGAGAATTATCTGCTCTGAAATTTGTAACTCATATGCTTACACCACAGGACTTAGTAAGAAGGATAACCTTTATAATACAACACCT   256
trnA  ---------------------------------------------------------------------------------------------------------------------------------   61
cox1  ---------------------------------------------------------------------------------------------------------------------------------   0
```

cox1

```
R     TACCTATGATAATTACCCGATGATTCTTCTCAACAAATCATAAAGATATTGGTACCCTATATTT-   320
trnA  -----------------------------------------------------------------   61
cox1  -----ATGTTCATTAACCGCTGACTATTCTCTACCAATCACAAAGACATCGGAACCCTATATCTC   60
```

cox1

1.80.3 trnY-trnA -211

trnY

```
R     AAAAGGATTAAACCTTTATAAATGAGACT-ACAG-CTCACCACCTATTTCGGTCACCTTATCTATAGTATTACCCGATAGCTCTTCTCAACAAATAAAATATTGTACCCTTATTTAGTATTGTACCTA   126
trnY  AAGAGGCCTTATCCTCTGTGCTT-AGATTTACAGTCTAA-----------------------------------------------------------------------------------------   38
trnA  --------------------------------------------------------------------------------------------------------------------------------   0
```

trnY

trnA

```
R     ACCTGTAATCGCATTATCTATCTTAATTTAGACTATCAGAAATGATTAAGTTTATATGATACACCATTTAAAATTATTCAATTAACCTTTAAAATCCTAAATAAAGATAAATTTAATCTTTGTAAGAC   254
trnY  --------------------------------------------------------------------------------------------------------------------------------   38
trnA  --------------------------------------------------------------------------------------------------------------------------TAAGGA   6
```

trnA

```
                                    trnA
R     CTGTAAGACTTTATCTTACATCTTATGAATGCAACCCAGACACTTTAATTAAG-   307
trnY  ------------------------------------------------------   38
trnA  CTGCAAGACTACATCTTACATCTAGTGAGTGCAAATCAATTGCTTTAATTAAGC   60
                                    trnA
```

1.81 NC_004373-NC_008123

Avg ovsize: -165

LCA: Ophidioidei-suborder

1.81.1 trnM-trnF -71

```
                         trnM                                                  trnF
R     GCTAAGACAAGCTTTTGGGCCCATACCCCAAACATGTAGGTTTAACTCCTTCTTTTGCTATGGTTGTCCGAGTAGCTCAACTAAAGCATAACATTGAAGTTGTTAAGATGGGACCTAGCCCCC-C   124
trnM  -----------------------------------------------------------------------------------------------------------------------------   0
trnF  -----------------------------------------------------------------------GTAGCTTAAATAAAGCGCAACACTGAAGTTGTTAAGATGGGCCCTAGAAAGCTC   54
                                                                                                     trnF
```

1.81.2 trnQ-nad2 -80

```
                         trnQ                                                                           nad2
R     GGGCTCGAACCTCCCCCGGGGAGATCAAAACTCCCAGTGCTCCCACTACACCACTTTCTAGCAGGCCCCCCCCCCTACGGGCGGTTAACTCCGCCACCTGCTCATGAGCCCATATATCATAACTGCCC   128
trnQ  GGATTCGAACCCATCCTATAGAGATCAAAACTCTATGTGCTCCCAATACACCACCTTCTA--------------------------------------------------------------------   60
nad2  --------------------------------------------------------------------------------------------------------------------------------   0
                         trnQ
```

```
            nad2
R     TCCTTATGAGCCTAGGACTCGGCACTACCATTAC-    162
trnQ  -----------------------------------    60
nad2  ------------TAATACTGGGCACAACCATTACA    23
                   nad2
```

1.81.3 trnP-trnM -345

```
R     -AGATTTTAACTCTCACTACTAGCCCCCAAAACTAATATTC-TAACGGTAAACTACCCTCTGCTATATGCAACGTGACCGCTACAGTACCCACACACATACATGTACACATCTATTTATATAGATATA    126
trnP  GAGAATCTAACTCCCACCACTAAACTCCCAAAGCTAGTATTCTTAAC--TAAAATACTTTTTG------------------------------------------------------------------    60
trnM  --------------------------------------------------------------------------------------------------------------------------------    0

R     CATATATACTTATGCATTTATATATCTATACCTATGTAGATACGTATACCCACATCTGTATCTACAACCATTTAGGCGTTTCTACACCTGTGCCTGTGTCCACCTGTATATAACTATGTATGTACAGA    254
trnP  --------------------------------------------------------------------------------------------------------------------------------    60
trnM  --------------------------------------------------------------------------------------------------------------------------------    0

R     TATATATATATATATATATATATATATGCAGCTGCATACATGCACACCCCCACGCTATACAACACGCGCCCTATGAGAGTAAGTCGCTACAACCCCGCACACGGTAAAATCAGCTAAGACAAGCTTTTGG    382
trnP  --------------------------------------------------------------------------------------------------------------------------------    60
trnM  --------------------------------------------------------------------------------------------------------------------------------    0

R     GCCCATACCCCAAACATGTAGGTTTAACTCC    413
trnP  -------------------------------    60
trnM  -----------------------TTAGCT--    6
```

Annotations: *nad2* (above R and below nad2 in the first alignment); *trnP* (above R and below trnP); *trnM* (above R, spanning the end of the third block and the fourth block, and below trnM).

1.82 NC_011009-NC_011007

Avg ovsize: -201
LCA: Platytroctidae-family

1.82.1 trnS2-cox2 -66

```
                                          trnS2
R     GGAGTTGAGCCCCCATATGCTGGTTTCAAGCCAGCCGCATAACCGCTCTGCCACTTTCTTCTATC-AGAACCCTAGTTAAAATCACATTACACTGCCTTATCGAGACAGATTTGTGGGTTAAAACCCC   127
trnS2 GGAGTTGAGCCCCCATATGCTGGTTTCAAGCCAGCCGCATAACCGCTCTGCCACTTTCTTCTATCAAGAACCC-------------------------------------------------------    73
cox2  --------------------------------------------------------------------------------------------------------------------------------     0
                                          trnS2
```

```
                                                     cox2
R     GCGGGTCCTGAGCTTTAAGCTAGAATGGCACATCCCTCACAATTAGGATTCCAAGACGCGGCCTCACCAGTAATAGAAGAGC-T   210
trnS2 ------------------------------------------------------------------------------------    73
cox2  -----------GCTTTAAGCTAGAATGGCACATCCCTCACAATTAGGATTCCAAGACGCGGCCTCACCAGTAATAGAAGAGCTT    73
                                                     cox2
```

1.82.2 cox1-trnD -72

```
                                          cox1
R     GATGCCCTCCCCCCTACCACACATTCGAGGAGCCAGCTTTTGTCCAAGTTCAATCAAACTGACGAGAAAGGGAGGAGTTGAGCCCCCATATGCTGGTTTCAAGTCAGCCGCATAGCCGCTCTGCCACT   128
cox1  GATGCCCTCCCCCCTACCACACATTCGAAGAGCCAGCTTTCGTCCAAGTACAATCAAACTGA------------------------------------------------------------------    62
trnD  --------------------------------------------------------------------------------------------------------------------------------     0
                                          cox1
```

```
                                        trnD
R     TTCTTCTATC-AGAACCCTAGTAAAA-ATCACATTACACTGCCTTGTCGAGACAGATTTGTGGGTTAAAACCCCGCGG   204
cox1  ----------------------------------------------------------------------------   62
trnD  ------TATCAAGAACCCTAGTAAAATAGCACATTACACTGCCTTGTCGAGACAGAGTTGTGGGTTAGAGCCCCGCGG   72
                                        trnD
```

1.82.3 trnD-trnS2 -465

```
                                   trnD
R      AAAATCACATTACACTGCCTTGTCGAGACAGATTTGTGGGTTAAAACCCCGCGGGTCCTGAGCTTTAAGCTAATGGCACATCCCTCACAATTAGGGTCCCAAAACGCGGCCTCACCAGTAATAGAAGA   128
trnD   AATAGCACATTACACTGCCTTGTCGAGACAGAGTTGTGGGTTAGAGCCCCGCGGGTCCTGAGCTTTAAGCTA--------------------------------------------------------   72
trnS2  --------------------------------------------------------------------------------------------------------------------------------   0
                                   trnD

R      GCCTCTCCGCTTCCATGACCACGAACTTGTAATCGTGCTTCTATTAGCACCTTCGTCCTTTACATTGTGGCAACGGTCTCAACTAAACCCACCAACAAATACACTCTGGCCCCCAAAGAAATTGAAAT   256
trnD   --------------------------------------------------------------------------------------------------------------------------------   72
trnS2  --------------------------------------------------------------------------------------------------------------------------------   0

R      CGTATGAACTATTCTCCCCTCAGTAATTCTTATCTGATTACTCTCCGCATACTGGACGAAATTAATGACCCACACCTCACCAGCAAAGCAACGGGTCACCAGTGGTACCAAAGCTACGAGTGTAAAAA   384
trnD   --------------------------------------------------------------------------------------------------------------------------------   72
trnS2  --------------------------------------------------------------------------------------------------------------------------------   0

R      CTACGAAGACCCAGCCTTAGACTCATACATGCCCACGGCCAGTTTCGACTCCTCGAAGCGGATCCCCCCGCCACAAACGTAGAATGACTCACGGATGCCCTCCCCCTCTACCACACTCGAAGAGCCAG   512
trnD   --------------------------------------------------------------------------------------------------------------------------------   72
trnS2  --------------------------------------------------------------------------------------------------------------------------------   0
```

```
                                                        trnS2
R      CTTTTGTCCAAGTTCAATCAAGCTGACGAGAAAGGGAGGAGTTGAGCCCCCATATGCTGGTTTCAAGCCAGCCGCATAACCGCTC-   597
trnD   --------------------------------------------------------------------------------------    72
trnS2  -------------------------ACGAGAAAGGGAGGAGTTGAGCCCCCATATGCTGGTTTCAAGCCAGCCGCATAACCGCTCT    61
                                                        trnS2
```

1.83 NC_010199-NC_010268

Avg ovsize: -217

LCA: Percomorpha-order

1.83.1 trnS1-trnL1 5

```
                                 trnS1                                                      trnL1
R      CCTGCTGGCAACGAAGACTGCTAATCCTCATCCCCTCGGTTGAAATCCGAAGCTCACTCAATGCTCCTAAAGGATAATAGCCCTTCCATTGGTCTTAGGAACCAAAAACTCTTGGTGCAAA-   121
trnS1  CTCGCTAGCAACGAAAACTGCTAATTTTCGCAACTTTGGTTGAACCCCAAAGCTCACTC---------------------------------------------------------------    59
trnL1  ------------------------------------------------------CCCTC-ATACTTCTGTAGGATAACAGCGCATCCATTGGACTTAGGATCCAAAAACTCTTGGTGCAAAT    67
                                 trnS1
                                                                                            trnL1
```

1.83.2 trnH-nad5 -126

```
                                 trnH
R      GTTTAATAAAAACATTAGATTGTGATTCTAAAGACAGAAGTTAAAAC-CTCCTTATCCACCCCAAAACAATAGCTCCTGAAACACTTCTTAAACCCAGTGTAGTCACCAATGCCCTTGGTTCAAATCC   127
trnH   -TTTAACAAAAATATTAGATTGTGATTCTAAAGATAGGGGTTAATCTGCCCCTCGTCCACCATAA---------------------------------------------------------------    64
nad5   --------------------------------------------------------------------------------------------------------------------------------     0
                                 trnH
```

```
                                  nad5
R      AAGTAGTAGCCATGCCCCCCTTACACATTACCTTAACATCCACCCTAACAATAACCCTCCTGCTCTTAGC   197
trnH   ----------------------------------------------------------------------    64
nad5   ---------------------------------------------------------------TCTTA--     5
                                                                        nad5
```

1.83.3 trnL1-trnH -532

```
                               trnL1
R      AATAGCCCTTCCATTGGTCTTAGGAACCAAAAACTCTTGGTGCAAATCCAAGTAGCAGCTACAACCACGCCCCTAACTTCCACCTTTTCCTTATACCTAAAAATACCAGGCCCACACCCAAGTACCAC   128
trnL1  AACAGCGCATCCATTGGACTTAGGATCCAAAAACTCTTGGTGCAAATCCAAGTGGAAG-TA-------------------------------------------------------------------    60
trnH   --------------------------------------------------------------------------------------------------------------------------------     0
                               trnL1

R      TAGTAATTATTCCGCCAGCCCAGCATATAAGCTACCCCAAAATCAAGCCGCTACCACTAACTGACCTAAATCTTATATTTTACACTCATTTAACCCAACATCTTAACTGGCTTATCATAAACAAATAT   256
trnL1  --------------------------------------------------------------------------------------------------------------------------------    60
trnH   --------------------------------------------------------------------------------------------------------------------------------     0

R      GTATAACTTAATTACACCCGTCTAAACGACATCATAATCACAAGAACCAGGCTAATCTTCTCCCAACACAACTATTGTAATAGACTAATTTTACATATGGGCTATAAAGAACAAAATACCTATTTACA   384
trnL1  --------------------------------------------------------------------------------------------------------------------------------    60
trnH   --------------------------------------------------------------------------------------------------------------------------------     0

R      TCTTTATTAATGAAACTTAAGCCTCAGACACTTAAAATATTACCCTTGCACCACCAAGATAAAAGCCTAGAGATTCGGATATCCGAGTATTCTAGGCTTTTATTATCGCCGGGCTACGCCACACGACT   512
trnL1  --------------------------------------------------------------------------------------------------------------------------------    60
trnH   --------------------------------------------------------------------------------------------------------------------------------     0
```

```
                                                                                    trnH
R      TATTCAATACTAATGTCCCACTAGAACACAACTTTTAACATATAAAATATACCCAACCCGTGTACCTATAATATTTTCCCCGTAGATATAGTTTAATAAAAACATTAGATTGTGATTCTAAAGACAGA   640
trnL1  --------------------------------------------------------------------------------------------------------------------------------    60
trnH   ---------------------------------------------------------------------------------GTAGACGTAGTTTAACAAAAATATTAGATTGTGATTCTAAAGATAGG    47
                                                                                    trnH
```

```
       trnH
R      AGTTAAAAC-CTC   652
trnL1  -------------    60
trnH   GGTTAATCTGCCC    60
       trnH
```

1.84 NC_010199-NC_022480

Avg ovsize: -229

LCA: Perciformes-order

1.84.1 nad4-trnS1 -61

```
                            nad4                                                                                     trnS1
R      ATCTCCTCCCCCTGCTTCTCCTGATTACAAAACCCGAGCTAATTTGTGGCTGAATTATATGTAGGTACAACTTTAAAAGCCCTCCCCAAGAATAGACTTAAAACCCGCGCCCACGAGAGAGGCCTGCT   128
nad4   ACATCCTTCCCCTCATACTACTCATCCTAAAACCAGAACTTATCTGATGCTGAA--------------------------------------------------------------------------    54
trnS1  -------------------------------------------------------------------------------------------------------------------AAAGAGGCTTGCC    13
                         nad4
                                                                                                                       trnS1
```

trnS1

```
R      GGCAACGAAGACTGCTAATCCTCATCCCCTCGGTTGAAATCCGAA-   173
nad4   ----------------------------------------------   54
trnS1  CGCAACAGAGACTGCTAATCCCTGTCACTTTGGTTGAACTCCAAAG   59
```

trnS1

1.84.2 trnH-nad5 -94

trnH

```
R      GTTTAATAAAAACATTAGATTGTGATTCTAAAGACAGAAGTTAAAACCTCCTTATCCACCCCAAAACAATAGCTCCTGAAACACTTCTTAAACCCAGTGTAGTCACCAATGCCCTTGGTTCAAATCCA   128
trnH   GTTTAACTAAAACATTAGATTGTGATTCTAAAAACAGGGGTTTAAGCCCCCTTGTCCACC--------------------------------------------------------------------   60
nad5   ----------------------------------------------------------------------------------------------------------------------------------   0
```

trnH

nad5

```
R      AGTAGTAGCCATGCCCCCCTTACACATTACCTTAACATCCACCCT-AACAATAACCCTCCTGCTCTTAGC   197
trnH   ----------------------------------------------------------------------   60
nad5   --------------------------TTATAATAACAACCAGCCTGATCCTTATCTTTACCACTCTGTTC   44
```

nad5

1.84.3 trnL1-trnH -534

trnL1

```
R      -AATAGCCCTTCCATTGGTCTTAGGAACCAAAAACTCTTGGTGCAAATCCAAGTAGCAGCTACAACCACGCCCCTAACTTCCACCTTTTCCTTATACCTAAAAATACCAGGCCCACACCCAAGTACCA   127
trnL1  TAACAGTCATCCC-TTGGTCTTAGGAACCAAAAACTCTTGGTGCAAATCCAAGTAGCAGCT------------------------------------------------------------------   60
trnH   -------------------------------------------------------------------------------------------------------------------------------   0
```

trnL1

```
R      CTAGTAATTATTCCGCCAGCCCAGCATATAAGCTACCCCAAAATCAAGCCGCTACCACTAACTGACCTAAATCTTATATTTACACTCATTTAACCCAACATCTTAACTGGCTTATCATAAACAAATA  255
trnL1  --------------------------------------------------------------------------------------------------------------------------------   60
trnH   --------------------------------------------------------------------------------------------------------------------------------    0

R      TGTATAACTTAATTACACCCGTCTAAACGACATCATAATCACAAGAACCAGGCTAATCTTCTCCCAACACAACTATTGTAATAGACTAATTTTACATATGGGCTATAAAGAACAAAATACCTATTTAC  383
trnL1  --------------------------------------------------------------------------------------------------------------------------------   60
trnH   --------------------------------------------------------------------------------------------------------------------------------    0

R      ATCTTTATTAATGAAACTTAAGCCTCAGACACTTAAAATATTACCCTTGCACCACCAAGATAAAAGCCTAGAGATTCGGATATCCGAGTATTCTAGGCTTTTATTATCGCCGGGCTACGCCACACGAC  511
trnL1  --------------------------------------------------------------------------------------------------------------------------------   60
trnH   --------------------------------------------------------------------------------------------------------------------------------    0

                                                                                                                    trnH
R      TTATTCAATACTAATGTCCCACTAGAACACAACTTTTAACATATAAAATATACCCAACCCGTGTACCTATAATATTTTCCCCGTAGATATAGTTTAATAAAAACATTAGATTGTGATTCTAAAGACAG  639
trnL1  --------------------------------------------------------------------------------------------------------------------------------   60
trnH   -----------------------------------------------------------------------------------TAGACATAGTTTAACTAAAACATTAGATTGTGATTCTAAAAACAG   45
                                                                                                                    trnH

          trnH
R      AAGTTAAAACCT-C  652
trnL1  --------------   60
trnH   GGGTTTAAGCCCCC   59
          trnH
```

1.85 NC_008778-NC_006286

Avg ovsize: -247

LCA: Episquamata-order

1.85.1 nad5-cob 10

```
                        nad5                                              cob
R     ------AT---CTCTTATT-ATATTTACTGC-ACTAACCACAATTACC-CTAACCTCTTTCAATACGCCT-TAATGACCCAACTAAAAACCAATCCTCTATTATCAACCCTAAACCATGCACTA-ATC   114
nad5  GGACGAATAAAATCTTATTTATCATTATTCCTACTAACCATGTTTATC-CTA-C--------------------------------------------------------------------------    52
cob   --------------------------------------------TATCACTA-CAACT-ACAA-GCACCAATAATGGCCCACATCACACGCAAGCATC-ATC--CAATCTTTAAA-ATGGTCAACAGC    77
                        nad5
                                                                                         cob
```

```
       cob
R     A-ACCT---   119
nad5  ---------    52
cob   ACATTTATT    86
       cob
```

1.85.2 nad6-trnP -57

```
                                  nad6
R     ACCAAC-C-AA-AAGACC-A-CACACAACCAAACCACCTAACAACATACAAATAAAATAAGACATTCA-CTT-TTACTCC-ACTG-AGCATGTAAGTTCAATAAACTCATG-TAACACTGTAAAACCC   118
nad6  CGCAACACCAACAATAGCTATCAAAAATGCCCACC-CC-AA-AAGATA-A-GTAATATATATCATAGAGCTTACTACCACAACGGTAGCACATCAGA-AAATAAAATTCTGCTATCACTACAA-----   117
trnP  --------------------------------------------------------------------------------------------------------------------------------     0
                                  nad6
```

```
                                       trnP
R     GATCAGAAGAAAGAAACTCTCACCCCTAGTCCCCAAAACTAGAATTCTACACTTAAACTAC   179
nad6  -------------------------------------------------------------   117
trnP  ----------------------------------------------------TTAAACTA-     8
                                                          trnP
```

1.85.3 trnT-trnE -696

```
      trnT
R     TAGCTTA--ATACAAAGCATTGGCCTTGTAAGCCGAAGATG-GCAATCAACCTCCCTACGACATTCTGCACCAACTACCTCAATTCCCCCTACATTTTCACACACTTCATATCTTCATTTTTTGAAAG   125
trnT  -AGCTTACCAGATAAAGCGCCGGCCTTGTAAGCCGGAAACGAGCGCTCCGC-TCC-------------------------------------------------------------------------    53
trnE  --------------------------------------------------------------------------------------------------------------------------------     0
      trnT

R     GCCCTTTCTTGAACGTCAGCTTGCTAAAATGTCCTAAAATCCGGCTTTTCCAAATTTTATGTCTATCGAGCAGTACAAACGCGCCGCCTCCTGAATTTGGCGTAAGTGATGGATTTTTTATAATTTCG   253
trnT  --------------------------------------------------------------------------------------------------------------------------------    53
trnE  --------------------------------------------------------------------------------------------------------------------------------     0

R     TATGGTTTTCAGCTTCGTTTTAAACCTTATATTTAAAGCACTCAACTAAGCGCTGGCTACCCCTATCGACTGCCAATCATGACCAGTCTCGTGGCTCACTCTTACAAGTTGCACCTATTTAATGACCT   381
trnT  --------------------------------------------------------------------------------------------------------------------------------    53
trnE  --------------------------------------------------------------------------------------------------------------------------------     0

R     TTCCAATACCTCTGGTTGTTAGGTCAGGCACTATCTTATAAGGTTACCAATCCCGTGGTCTTCACGACACATGGCGTTTGGGGGGATCTCATGGAGCTAACGGTCCTTAATCACGGTCACCCTGGTTT   509
trnT  --------------------------------------------------------------------------------------------------------------------------------    53
trnE  --------------------------------------------------------------------------------------------------------------------------------     0

R     AGGCGCTTTGGCCTTTCTCTCTTTTTTTAGGTGGAATTCAATAGCACGTTCGTCCGGGGCCAGCCCATTAGTTAAAATTGTCCATGTTACAATCGAACCCTCTACAGCCATGTTGAGTTATCTTTTAA   637
trnT  --------------------------------------------------------------------------------------------------------------------------------    53
trnE  --------------------------------------------------------------------------------------------------------------------------------     0

      trnE
R     TGGTCTCGGGACATACTCTATTTTAAAAAAAACATAAAAATTTATTTTTTCCCCCCCTCCCCCCAAAATCCACTACCTATTTTTATTTGGACTATCCAAAACCAAGGACACGAAAAATCCACGTTGTT   765
trnT  --------------------------------------------------------------------------------------------------------------------------------    53
trnE  ---------------------------------------------------------------------------------------------------------------GAAAAACCGCCGTTGTG    17
      trnE
```

```
            trnE
R       ATTCAACT-     773
trnT    ---------      53
trnE    -TTCAACTA      25
            trnE
```

1.86 NC_006343-NC_006335

Avg ovsize: -252
LCA: Plethodon-genus

1.86.1 cob-trnT 31

```
R     TT-TTACTTTTCCTTCTACTCTCATTAAGTGGATTAATAGAAAATAAACTTATA--AAAT-GATGCTATTGTAGTTTAATTAAACATTGGTCTTGTAAACCAAA-ATTGGGGGCTAATTAGCCC   119
cob   TTATTTATATTC-TTC--CTCCCACTAATAGGAGTAGCCGAAAACAAACTTATA--AAAT-GATA-----------------------------------------------------------    59
trnT  ----------------------------------TAATTGATA-GAAATTTACACCAAATCAATACTACCGCAGTTTAAGAAAACATCAGCCTTGTAAGCTGAAGAATGGGGATTTAAAATCTC    89
```

Annotations: *cob* (above, R), *trnT* (above, R), *cob* (below, trnT), *trnT* (below, trnT)

1.86.2 nad5-nad6 3

```
        nad5 (R: cols 1-61)                                          nad6 (R: cols 62-101)
R     ATATTTTTAATTTCTGCCCTCTTCGCACTTATTTTTATTCTCCCTAACAGCACGCAAAGACCCACAAAACACCCCCCAAGTAATCCCTAACACTACAAATA-   101
nad5  -TA--G-TAA-CACTTACCTCTAC-C-CTT-TTAATGGCCTCCC----------------------------------------------------------    36
nad6  -----------------------------------------CCCTCACAGCACGTAAAGATCCACGAGATACCCCACGAGTAATTTCTAATACCGTAAATAA    61
        nad5 (cols 2-44)                         nad6 (cols 42-102)
```

1.86.3 trnT-trnP -791

```
        trnT (R: cols 1-62)
R     GTTTAATTAAACATTGGTCTTGTAAAC-CAAAATTGGGGGCTAATTAGCCCCCCCCTAGCCTCCACTAATATAAAGTGTGTCTTAGCCTTATATAACTGAACTATATATTCTTCACCATCAACTATGA   127
trnT  GTTTAAGAAAACATCAGCCTTGTAAGCTGAAGAATGGGGATTTAAAATCTCCC---------------------------------------------------------------------------    53
trnP  --------------------------------------------------------------------------------------------------------------------------------     0
        trnT (cols 1-53)

R     ACGGATTGAATTACCTAACCTAGCCCAAACGGACACCGGCACACAATCTAATATGGGCTAATTATTTATGCCCCAGCCTTATATAACCGAACCATATATTATCCACCACCCGCTATGAACGGATTGAA   255
trnT  --------------------------------------------------------------------------------------------------------------------------------    53
trnP  --------------------------------------------------------------------------------------------------------------------------------     0

R     TTACCTAAACCTAACCTAAACGGACGCAACACAGTCTAAAATCTATAATATATACCCGCAGGTCATGCGAATATCTCTCAGCTCATTTATATAAATACACAGTAAATTACCTCCACAAATAAAATAAA   383
trnT  --------------------------------------------------------------------------------------------------------------------------------    53
trnP  --------------------------------------------------------------------------------------------------------------------------------     0

R     CAACAAATACTACTAAACTGTTATACCTCTCGAACAAACCACTATAATATAAGAAATAAATACCTTACCCTTACTTCCCCCCATAGTTTTTATTTACCTCCAGCATCTACCTAAATATACCAAATATT   511
trnT  --------------------------------------------------------------------------------------------------------------------------------    53
trnP  --------------------------------------------------------------------------------------------------------------------------------     0
```

```
R     GTCTTGCCACAGCCCCAACCTCATTCAATGTATAAACACACACATATCCCAGGTTTTCCTTATATACACAAATCTACCCCGGAAATTTAACACAAGCAGGCCATTTACCATAACTCAAACTTATAGTA  639
trnT  --------------------------------------------------------------------------------------------------------------------------------  53
trnP  --------------------------------------------------------------------------------------------------------------------------------  0

R     CAACAGACCCCCCCGCATAAACCAAACTTGATGGGTAAAAAAATGTGTAATTACCTTGCAAATTTAAAACCGTTTTACAAATAATATATAATCCTGAACAAAAAATCTACACTAAAATAAAAATTTCT  767
trnT  --------------------------------------------------------------------------------------------------------------------------------  53
trnP  --------------------------------------------------------------------------------------------------------------------------------  0

                                                                                                                   trnP
R     TAAAAACAGAAAAAAATATTAACCAAAAACAAACATCTCACCTCACTTCCCATAATCACTTTATAGCCCAATTTCAATTTTAAGTTAAAAATTCAAGCAAAAAAGATTTTCACTTTTGTCTCTGACAC  895
trnT  --------------------------------------------------------------------------------------------------------------------------------  53
trnP  ----------------------------------------------------------------------------ATATAAAAATAATT-TTTAAGCAGGAAGGATTTACACCTTCACCATTGACAC  51
                                                                                                                   trnP

              trnP
R     CCAAGGCCAAAATTCTAGAAACT--  918
trnT  -------------------------  53
trnP  CCAAGGCCAAAATTCTA-AATTTAA  75
              trnP
```

1.87 NC_022670-NC_012688

Avg ovsize: -255

LCA: Neoptera-subclass

1.87.1 trnM-nad2 6

```
                            trnM                                                             nad2
R     -GCTAATATTAAGCTTATAGGTTCATACCCTATCAATAGAAGTA-AAACCTTCTTCTAATTAATAAATATTAAA-ACAATAATAACAAGACTAACTT-TAAGAACCCTTATAGTAATATCAT--C-   119
trnM  AGCTAATA--AAGCTTAAAGACTCATAATCTTTATATAGAAATTTCAATTTTCTTCTTTTTA----------------------------------------------------------------   60
nad2  --------------------------------------------------------TAATTAATAAA-ATTATTGATCATATTAATTTT-CTATTTTATTTTAATA-TTA-AGAACTTTAATTACT   66
                            trnM
                                                                                         nad2
```

1.87.2 trnI-trnM 6

```
                          trnI                                                               trnM
R     AGATGCCTGAATAAAGGATTACTTTGATAGAGTAAATCATGCACAATTATGCTCTTACTAT-AAATAATTAGATAAGCTAATATTAAGCTTATAGGTTCATACCCTATCAATAGAAGTAA-AACCT--   124
trnI  ----GCCTGATAAAAGGATTATTTTGATAGAATAATTCATG-AAAATAAAGTTTTT-CTTTCATTTAATAA---------------------------------------------------------   65
trnM  -----------------------------------------------------------------TAA-AAGATAAGCTAATA--AAGCTTAAAGACTCATAATCTTTATATAGAAATTTCAATTTTC   60
                              trnI
                                                                                             trnM
```

1.87.3 rrnS-trnQ -777

```
                             rrnS
R     --AAATTAT-TATAAG-GACT-AATTGTTTAACCGCAACTGCTGGCACAAAATTAGTTAGACCTTAATCAATTAACTGGATCATAAATAATTACATTTCCAATTCTACAAATTGTGCCATACACCATT   123
rrnS  TTATATTTTATATAAAATACTGAATTTTTTTACT-CAA------------------------------------------------------------------------------------------   37
trnQ  --------------------------------------------------------------------------------------------------------------------------------   0
                rrnS
```

```
R     AAGAAAAAAATATACATATATAATTCATCAATTAACAATAAAAAGAGCAAGGATCAAACTCACCTTATACGTTATCAGCCATTATATAAAACGGATCTTAACAACCCCCTCCTATCCCTCTCCCAGGCA   251
rrnS  --------------------------------------------------------------------------------------------------------------------------------   37
trnQ  --------------------------------------------------------------------------------------------------------------------------------   0

R     TAGGGTACCATAGGGTACACGGGGTTAATAATTATTTCTACTATTATAAATAGTTACATTATTATCCACTAAGTATCAATATATATTCATAAATAGTTATTTATCATAGGGTTATTTAAAGGTATTCT   379
rrnS  --------------------------------------------------------------------------------------------------------------------------------   37
trnQ  --------------------------------------------------------------------------------------------------------------------------------   0

R     TCGTTAATTGTAACTCAGATAGGTAAATTCTCCAGGTCCATAGATGTACCACTCTCTTAATACACACATAGGCACTAAGGGGTATAAATAGTCTCATTACAGGCCACTTATAGGTCTCATCCCTTGGA   507
rrnS  --------------------------------------------------------------------------------------------------------------------------------   37
trnQ  --------------------------------------------------------------------------------------------------------------------------------   0

R     TGGGGGGTAGGGGGGGGGGTATTCACACTTAAGTATATAGTCTAGGATTAAATAGAATATAAATAATATTAATAATAATAATTAGTAATTAAATTAAATTCAAACGTTACATTACAAGATATAAAGAT   635
rrnS  --------------------------------------------------------------------------------------------------------------------------------   37
trnQ  --------------------------------------------------------------------------------------------------------------------------------   0

R     CTATATAATTCAGCTATCCCCCTAATAGTGTGTCACAACTATTAGTAGCATAAACCATAAATTCTTAAATTATCAAACATGTAACTTATTAAAATAAGATTTGAGCGGATCATTCACTTTCCTAATTT   763
rrnS  --------------------------------------------------------------------------------------------------------------------------------   37
trnQ  --------------------------------------------------------------------------------------------------------------------------------   0

                                trnQ
R     CTATACTAGTAAGAATCAAACTTACGCATTTGTGGTCAAATCACAACGTGCATCATACA-   822
rrnS  ------------------------------------------------------------   37
trnQ  -----------------------------------------------GTGCCCCATACAC   13
                                                        trnQ
```

1.88 NC_003979-NC_008831

Avg ovsize: -269

LCA: Harpacticoida-order

1.88.1 cox3-rrnL 2

cox3 *rrnL*

```
R      GATGTGGTGTGACTCTTTCTTTACTTAACTATTTATTGGTGGGGGAATTCTAGGGTCTA-AAAACTTAGGTTAAAAAAACCTGCCAAATTTCAAATTTAGTAATGAGTTTTACTCAG-TT-T-   119
cox3   GATGTGGTTTGGCTATTTCTGTATTTAGTTATTTATTGGTGAGG-AA-ACTCGG-TCTACTAA------------------------------------------------------------    60
rrnL   -------------------------------------------------------------AAACTTAGGTTAAATAAACCAATTAAATTTCAAATTTAATAATGAG-A-GAATCAGTTTTA    60
```

cox3

rrnL

1.88.2 trnS1-trnW -169

trnS1 *trnW*

```
R      AAGAGGTGTAAAGTTTAACTTCTAATTAAGCTAAAGCAGAAATGTTGCCTCTTTAGAAGAGTCCATGGGGGCAAGTTGAGCACGATAAAATAGGCTTAAATATTGAAAGGTTTGCGTGGGGCAGAACT   128
trnS1  A-------------------------------------------------------------------------------------------------------------------------------     1
trnW   --------------------------------------------------------------------------------------------------------------------------------     0
```

trnS1

trnW

```
R      TGAGGCGGAGCTCAAGTCTGTGAGTTGGGGGGGTAGCAAAGC   170
trnS1  ------------------------------------------     1
trnW   ------------------------------------------     0
```

1.88.3 trnW-cox1 -641

trnW

```
R      GCGTGGGGCAGAACTTGAGGCGGAGCTCAAGTCTGTGAGTTGGGGGGGTAGCAAAGCATTATTTGTTGCAAAGAAGAATGCTGCGCCGGCTCATAGAGTAGTGGGGGCATCGGGGGAGGAGGTTGAGT   128
trnW   --------------------------------------------------------------------------------------------------------------------------------     0
cox1   --------------------------------------------------------------------------------------------------------------------------------     0
```

```
R     TGCTGGTATGCCAAGTTGAGGTGCGGCTATCCTAGCTCTAGCGCTTGACGGCTGATTAACGCCCGGAAAAAAGAAAGAACAAAAGAAAAGTTAAAAATTTTCGCGATACCAAAGGACCCCCTGGGGGC   256
trnW  --------------------------------------------------------------------------------------------------------------------------------     0
cox1  --------------------------------------------------------------------------------------------------------------------------------     0

R     TGGCTAACCTCAGTACCAAAGTGCCAGCAAGAAAATGATCAAGCTTGAAAATAATAGATAATTTCAGAGGGGGTGATAAAAAAATGTGTGTACCAAAGAAAAAAAATTTTTGAGTTTAGATTAAAACC   384
trnW  --------------------------------------------------------------------------------------------------------------------------------     0
cox1  --------------------------------------------------------------------------------------------------------------------------------     0

R     AGATAGCGTCGCCAACAATTTCATGGCGAATGGGGGGAAAATTGGAGAGTGGGGGGGGGTCATGAAGTGCTGCAAGACTTTTTTAGCTGAAGCCCAGATTAGGCTTTAAAGCCTAAACCTGTTGAAAA   512
trnW  --------------------------------------------------------------------------------------------------------------------------------     0
cox1  --------------------------------------------------------------------------------------------------------------------------------     0

R     ACAGGTCAGGCCGGCAAAAGTCTTAAAGTGGCAAAACGAGAGAATTTAGGCTCTAAACCCCCGATTTTCGGGAGGGGCGTGGTTAAAGTTGAGCACGATAAAATAGGCTTAAATATTGAAATGTTTAG   640
trnW  --------------------------------------------------------------------------------------------------------------------------------     0
cox1  --------------------------------------------------------------------------------------------------------------------------------     0

                                  cox1
R     AATAAAATGGAGAGGGGCGACTAATCATAAAGATATTGGTACATTATACCTTATTG-CTGG-   700
trnW  --------------------------------------------------------------     0
cox1  -ATAAAGTGGAGAGGGGCTACGAACCACAAAGATATTGGTACTTTATA-CTTGTTGGCGGG    60
                                  cox1
```

1.89 NC_004448-NC_001922

Avg ovsize: -286

LCA: Alligatorinae-subfamily

1.89.1 trnG-cox3 0

1.89.2 cox3-nad3 -74

```
                                      cox3
R     GACATTTTGTAGATGTCGTCTGACTCTTCCTTTACATCTCAATCTACTGATGAGGTTCATGCTCTTCTAGTATAAATAATACAAGTGACTTCCAATCACTAAACCCCTAACACACAACAAGGGGAAAA   128
cox3  GACACTTTGTAGACGTCGTCTGACTTTTCCTCTATATCTCGATCTACTGATGAGGATCGT--------------------------------------------------------------------    60
nad3  --------------------------------------------------------------------------------------------------------------------------------     0
                                      cox3

                                        nad3
R     GCAATCAACCTTCTTACCATATTCATAGTAACCTCCATCACCGCCGCAGCCGTAATCACTAT-   190
cox3  ---------------------------------------------------------------    60
nad3  ------AACCTATTTATCATACTCACAATATCCTCAATCACCGTTTCAATCGTAGTCGCCCTA    57
                                          nad3
```

1.89.3 atp6-trnG -784

```
                                      atp6
R     ATAATCCAAGCCTACGTCTTCGTCCTCCTATTATCCCTATACCTTCAAGAAAACACGTAATGTCACACCAAACACACTCCTTTCACATAGTCCACCCCAGCCCCTGACCCCTCGCCGGGGCCATAGCC   128
atp6  ATAATCCAAGCATACGTCTTCGTCCTCTTATTATCCCTATATTTACAAGAAAATTCATAA--------------------------------------------------------------------    60
trnG  --------------------------------------------------------------------------------------------------------------------------------     0
                                      atp6

R     GCCATATTATTAACAACAGGCCTGACCTTCTGATTCCACTATGACTCTAGCCTTATTCTATTGCTCGGCCTAATCACCACTCTATTAGTAATACTCCAATGATGACGAGACATTATCCGAGAAAGCAC   256
atp6  --------------------------------------------------------------------------------------------------------------------------------    60
trnG  --------------------------------------------------------------------------------------------------------------------------------     0
```

```
R     CTACCTAGGACACCACACACCTGCAGTACAAAAAGGACTACGCTACGGCATAATCCTTTTTATCACATCAGAGGTCTTCTTCTTCCTGGGCTTCTTCTGAGCATTTTATCACTCAAGCCTTTCCCCCA   384
atp6  --------------------------------------------------------------------------------------------------------------------------------    60
trnG  --------------------------------------------------------------------------------------------------------------------------------     0

R     CCCCTGAGCTAGGGGGACAGTGACCCCCAGTCGGAATTACCACCCTTGACCCATTTGAAGTTCCCCTCCTAAACACAGCTGTCCTCCTTGCCTCTGGGGTAACAGTAACCTGGGCCCACCACAGCTTG   512
atp6  --------------------------------------------------------------------------------------------------------------------------------    60
trnG  --------------------------------------------------------------------------------------------------------------------------------     0

R     ATGGAAGCCAACCGAACACAAGCAATTCAGGCCCTAACACTCACCGTACTCCTTGGCCTATACTTCACCGCCCTTCAAGCCATAGAGTACTACGAAGCCCCCTTTACAATCGCAGACAGCACCTACGG   640
atp6  --------------------------------------------------------------------------------------------------------------------------------    60
trnG  --------------------------------------------------------------------------------------------------------------------------------     0

R     ATCAACATTCTTCGTTGCAACCGGCTTCCACGGCCTCCATGTTATTATTGGCTCAACATTTCTCATAGTCTGCCTATATCGACAGACAAAATATCACTTCACATCCAACCACCACTTCGGGTTCGAAG   768
atp6  --------------------------------------------------------------------------------------------------------------------------------    60
trnG  --------------------------------------------------------------------------------------------------------------------------------     0

                                                   trnG
R     CCGCTGCCTGATATTGACATTTTGTAGATGTCGTCTGACTCTTCCTTTACATCTCAATCTACTGATGAGGTTCATG   844
atp6  ----------------------------------------------------------------------------    60
trnG  ----------------------------------------------------------------------------     0
```

1.90 NC_006286-NC_008225

Avg ovsize: -289

LCA: Teleostomi-superclass

1.90.1 trnE-nad6 2

```
                                       trnE                                                                  nad6
R     GCCGGCAATTCT-ACC-GGACCTGCGGTCTGAAAAACCGCCGTTGTGTTCAACTACAAAAACCCCCCTAAATACCCGAATAGCGCCCCGTTCCCCACCTAAAGA-TAACC-ATAATACCACAA-AAA   123
trnE  GCCCGGGCT-CTAACCAGGACCAATGACTTGAAAAACCACCGTTG-ATTCAACTACAAGAAC-------------------------------------------------------------------   60
nad6  ------------------------------------------------------------ACCCACTCTAATCGCACGCA-AG-GCCCCGTAAGATCCCCCACGAGTAACCTCCAATACTAAAGCCAG   66
                                       trnE                                                              nad6
```

1.90.2 nad5-trnE -83

```
                                  nad5                                                                 trnE
R     GGACGAATAAAATCTTATTTATCATTATTCCTACTAACCATGTTTATCCTACTATTCTAGACTTGACAATTTCCGCCGGCAATTCTA-CC-GGACCTGCGGTCTGAAAAACCGCCGTTGTGTTCAACT   126
nad5  --------------------------------------------------------------------------------------------------------------------------------   0
trnE  -----------------------------------------------------------------------------------TCTAACCAGGACCAATGACTTGAAAAACCACCGTTG-ATTCAACT   44
                                                                                                                   trnE
```

```
R     -   126
nad5  -     0
trnE  A    45
     trnE
```

1.90.3 trnP-trnF -787

```
                                      trnP
R     CT---TTAAGCGCGCCTCATCTCCCGCCCCCAAAACGGGCATTTTAAACTAAACTATCCTCTGCAATCGCCTGGGCTCTGCCAGGCGTTTATGTCAATTATGCGTATATAGAGGTACAGTACAAACTA   125
trnP  GAGACTTAAACTC-CCACC-C-CCAGCTCCCAAAGCTGAGATTCTTCATTAAACTATCCTCTGCAA-CCCC---------------------------------------------------------    67
trnF  --------------------------------------------------------------------------------------------------------------------------------     0
                                      trnP

R     TGTATAATAATACATTAATTTCTTGTCCCCATAACAGTGATTTGCCAAAGGTAAACTCTTAATTATACTAAAGATAGACTATGTATATCGTGCATAATATCATCTAACCATGGGTGAACCATATCTAC   253
trnP  --------------------------------------------------------------------------------------------------------------------------------    67
trnF  --------------------------------------------------------------------------------------------------------------------------------     0

R     ATTCGTTCTTTACAGTACTGCCAGACAAACCATGAATCAGTTCTTGTCAATACGAATATTGTCCGGTATTGGGTTATTTCTTTATTTAGCTTCTCACGTGAGAATCATCAACCCTTGTCAGTTAAACA   381
trnP  --------------------------------------------------------------------------------------------------------------------------------    67
trnF  --------------------------------------------------------------------------------------------------------------------------------     0

R     CTCATTCCCTAGTCTCAGGCCCATTAATATAGGTTGCAACCCTTACTCACTTTTTCCAAGGCCTCTGGTTGTAAGATCAGGGTCCTCTAAATCCTCAATCACCTCTCCTCACTTTTTCCAAGGCCTCT   509
trnP  --------------------------------------------------------------------------------------------------------------------------------    67
trnF  --------------------------------------------------------------------------------------------------------------------------------     0

R     GGTTGATGGGTTAATTACACTTTGCCGTAAACTCATAGCATCCCTGCACTTTCCGGCAGCTGGTATCTTTTATTTCTCTATCTAGGCCTCTTACAATCACCAGTGGAGTAGCCCAATCTAATTGTAGG   637
trnP  --------------------------------------------------------------------------------------------------------------------------------    67
trnF  --------------------------------------------------------------------------------------------------------------------------------     0

R     TGGAAGTCACGGTGCACTGCATTACTGTACTATTTCATCTCTTGAGTAATGTGTATGAATGTGCGATAGACATATTTTTATTAAAAAAACGCATACCCTGTTTTGCTGGTCTCACTCTTCGCCATTTT   765
trnP  --------------------------------------------------------------------------------------------------------------------------------    67
trnF  --------------------------------------------------------------------------------------------------------------------------------     0
```

```
                                                                                                             trnF
R     CTTCCTATGTTTCCCTCTTGTCACCAAGGATTTCAAATTCATTTTACCTAGTTACAATAATAAATACACAATAAATTGACCAACAAACTGTTTCTGTAGCTTAACCAAAGCAGGGCACTGAAGATGCC   893
trnP  --------------------------------------------------------------------------------------------------------------------------------    67
trnF  ------------------------------------------------------------------------------------------TTACCGTAACTTAATTAAAGTTTAATATTGAAGCTATT    38
                                                                                                             trnF
```

```
          trnF
R     AAGATGGCACCCCATC-GAAC   913
trnP  ---------------------    67
trnF  AGGATGGGCCCTAAAAAGCCC    59
          trnF
```

1.91 NC_023228-NC_006355

Avg ovsize: -289

LCA: Percomorpha-order

1.91.1 trnM-nad2 23

```
                           trnM                                                          nad2
R     GCTAAAACAAGCTATTGGGCCCATAC-CCCAGCCATATAGGTTAAAACCCTATCCATAACAT-ATGAACCCC-CTAGCACTATTTATACT-CATCACAGCACTCTTATCCGGCACATTTATTAC-   120
trnM  GCTAATAGAAGCTATTGGGCCCATAC-CCCAAAAATGTTGACTAA--------------------------------------------------------------------------------    44
nad2  ----------------------ATACACCCAAACAAGTTGATTAACTTCA-A-CGATTGCTTCATGAGCCCCTATATT-CTCTCGATCTTTCTTTTC-GGACTCGGCCTAGGAACCACAATTACA    99
                    trnM
                                                                                         nad2
```

1.91.2 trnQ-trnI 1

```
                         trnQ                                                    trnI
R     TTA-TGGAGTGCACAAAGAGTTTTGATCTCTTAAGTTGAGGTTCGAATCCTCCTTTCCTATATATTATCCCCAGGAGCTATGTCTGAAT-AAAGAGTCACTTTGATAGAGTGAAGCACGTAGATGAAA   126
trnQ  GTAGTGGAA-GCACCAAGAGTTTTGATCTCTTCAGGAAGGGTTCG-AGCC-CCTTTTTTCTATCTTTTCCCC--------------------------------------------------------    69
trnI  -----------------------------------------------------------------------CAGGAGCTGTGCCTGAATCAAAGGGTCACTTTGATAGAGTGAAGCATGGGGGTTAAA    57
                          trnQ
                                                                                     trnI
```

```
       trnI
R     ACCCT   131
trnQ  -----    69
trnI  ATCCT    62
       trnI
```

1.91.3 nad1-trnQ -893

```
                         nad1
R     TTCGTGATAGTAACC-CTAATAATTCTAGTTATAACAGCAGGTATTCCTCCAATAGACTAATATTTTACATATATGTAATATTTTACATATATGTAATATTTTACATATATGTAATATTTTGCATATA   127
nad1  TTTATAATC-TGACATCTGGCACTACTAATTGCATTCGCCGGACTTCCCCCA----------------------------------------------------------------------------    51
trnQ  --------------------------------------------------------------------------------------------------------------------------------     0
                      nad1
```

```
R     TGTAATATTTTACATATATGTAATATTTTACATATATGTAATATTTTACATATATGTAATATTTTACATATATGTAATATTTTACATGTATGTAATATTTTACATGTATGTAATAGAACATAATACAT   255
nad1  --------------------------------------------------------------------------------------------------------------------------------    51
trnQ  --------------------------------------------------------------------------------------------------------------------------------     0
```

```
R     TCCACTCATAAACTTACTAATAGATGTATTAAACGCCCATAGGTATATCATATATTATATATCAGTATATCACCTACATAGTAAATTAATAACCATACATTGGATTTCAAGAATCAAGCTCCTTACAT  383
nad1  --------------------------------------------------------------------------------------------------------------------------------  51
trnQ  --------------------------------------------------------------------------------------------------------------------------------  0

R     TCTCAAGATCTTATCTTGAGAGAATAGTATTCTCTCTACTTTATGCTAGTATGCGGGATCCCATCATGTACACCTCCTACTTATCTAGTCAGTTCTTATTTTATAACATACCTTGCATGCTGTTAA  511
nad1  --------------------------------------------------------------------------------------------------------------------------------  51
trnQ  --------------------------------------------------------------------------------------------------------------------------------  0

R     TAAAGTTCTACACATACTTGACATGAAGTGTTCTCCAGGGGATAAGGGGTTTTCCTCTCTCCCTTGTTGACTCGACTAGTGTCTGCTTAGAAGAATATATCTGGGTAACTCATAACCAATTTCATCTA  639
nad1  --------------------------------------------------------------------------------------------------------------------------------  51
trnQ  --------------------------------------------------------------------------------------------------------------------------------  0

R     TTACTACAAAAGACATACAGACATTTATTGGAACACTACATAAGTCCTATAAACATACATTTTTACTGTCTTTATATCCGCCCGCCTTAATACAAACTATAGTCCTATACACAGTAATAGAAATCCCG  767
nad1  --------------------------------------------------------------------------------------------------------------------------------  51
trnQ  --------------------------------------------------------------------------------------------------------------------------------  0

R     GACTCTTTAATTTACCTCAGTGATTTCGACATAAAAAATTTTCCATCTTACATCTCTCAAAGAAGCAACAAATATTAAACAAAATTTACTAAGCCTAAAAATTTTTTGAGATTATTCGATTATAAAAA  895
nad1  --------------------------------------------------------------------------------------------------------------------------------  51
trnQ  --------------------------------------------------------------------------------------------------------------------------------  0
```

trnQ

```
R     AATCAAAAAACAAGCTACTAAAATATCACAAACCAAGTAACTTTGAAAATT-TAGGAAATAGTGTTATGGAGTGCACAAAGAGTTTTGATCTCTTAAGTTGAGGTTCGAAT--  1005
nad1  -----------------------------------------------------------------------------------------------------------------  51
trnQ  -------------------------------------------------TTCTAGGAAGTGGTGTAGTGGAA-GCACCAAGAGTTTTGATCTCTTCAGGAAGGGTTCGAGCCC  63
```

trnQ

1.92 NC_006321-NC_001673

Avg ovsize: -307

LCA: Annelida-phylum

1.92.1 trnK-trnH 6

```
                                        trnK                                                                    trnH
R     GAGAAAGCTTGCTA--AGCATTAGTCTTTTAAACTAAAAAAGATCTCTTTGGTCTCTTTCCCCGTTAAAATAGTTTAAAAAAAACCCTGTGTTGTG--ACCACAAAATTGTATA-CCTATTACTT   120
trnK  -AGAGAGCTTGTAAATAGCACTTGACTTTTAA---------------------------------------------------------------------------------------------    31
trnH  --------------------------TTTTAACATTAAAAATTT-TCT-CGGAGT-TCTAATCATAAAGGTAGGTTACAAAAACCCCCGAATTGTGGTTCCGGAAACGTCAAAAGACCCTT--TT    94
                    trnK                                                                       trnH
```

1.92.2 trnI-nad3 -54

```
                                        trnI                                                                       nad3
R     CGC-CGGGCATGAACGGACAGCTTTGATGTAGCTGA-TAACGAAGTA-CTTCTTCTAATGTCTTGTTTACACATATGAACCTATTATTATTTACATTTATACTCAGACTAGTAGTATCC-TTCTTACT   124
trnI  AGCGCCGG-ATGAACGGATAACTCTGATGACGTTAATTAAGGAACAAGCTTC----------------------------------------------------------------------------    51
nad3  ----------------------------------------------------------------------------------------------------------ACTATTAGTCCCTATTATTATT    22
                                 trnI                                                                                        nad3
```

```
       nad3
R     TATCCT   130
trnI  ------    51
nad3  TTGGGG    28
       nad3
```

1.92.3 trnR-trnK -875

```
                                                      trnR
       ______________________________________________________________________________________
R      GTGGCAATTTATGCATTTTTGGTTTCGGCCCAAAAAAATGGGAAAATCCCCAACCTTTTATGTATATATTATATATGTTTGTGTTATAAATATTTATTACGGCAAAAATATATATACTTTTATATAGT   128
trnR   TAAGCA-TTAA-----------------------------------------------------------------------------------------------------------------------   10
trnK   --------------------------------------------------------------------------------------------------------------------------------   0
       ___________
          trnR

R      ATTTTAACTTAAAACTAAAAAAATACAACTAAATAAGGTAAAGAACTTTTCACAAGAAACTAAAAAAAGTTTTTAAATTTTTTTTTTTTCTTTATGTATAATGTACTATATATATATGGGATGAATTAA   256
trnR   --------------------------------------------------------------------------------------------------------------------------------   10
trnK   --------------------------------------------------------------------------------------------------------------------------------   0

R      AATTTATAAATTTTATTTAATAAATTATATTAAATGAGTTAATTATATTAATAAAGAAATTTATGTTATATATTAAATATATACTCTATATTTTTTTTTTCACTTAAGGAAAAAAATATCTCCAAAAA   384
trnR   --------------------------------------------------------------------------------------------------------------------------------   10
trnK   --------------------------------------------------------------------------------------------------------------------------------   0

R      CACACATACGTGCAATACCCCCTATTTAGGTGCTAGTACAGGTGTAAAGCCATATTTTAGTAAATACATAAAATTTTTAAAAAATACCCTGTTTAGGTTCTACTATAGTCCCAGACGGCCTCTTCCGG   512
trnR   --------------------------------------------------------------------------------------------------------------------------------   10
trnK   --------------------------------------------------------------------------------------------------------------------------------   0

R      ACACACCCCCCTAAACAACAAAAAATACACAAATATTACACTTTAGCCTTCTAACCAAGGTTAAAGTCAACTTTTACACTTAATTTAACTTTATTAGTGTTTTCCCTCGTAAAAAACTTTTTTTTCCA   640
trnR   --------------------------------------------------------------------------------------------------------------------------------   10
trnK   --------------------------------------------------------------------------------------------------------------------------------   0

R      TCTGTACATTTCTGCGGAAAACTTTTTTTTTGCCTTTTTTTTATAAGGCCATTCAGGTTAAAAATTCGCCATTTTACCCCTGTGCACGAAACACCCTTAAAATGACAAATTTTGACACTTTTTTACAC   768
trnR   --------------------------------------------------------------------------------------------------------------------------------   10
trnK   --------------------------------------------------------------------------------------------------------------------------------   0
```

trnK

```
R     GTTTTTTACACGTTTTTACATATTTTTTTTCCCCTAATTTTTTTTTTACTTTAAAGTAAATAAAGGTTATTATATTTATTTTTATATTTACAAACAATATTATCACATATAGAGAGGAGAGAAAGCTTG  896
trnR  ---------------------------------------------------------------------------------------------------------------------------------   10
trnK  -----------------------------------------------------------------------------------------------------------------------AGAGAGCTTG   10
```

trnK

trnK

```
R     CTA--AGCATTAGTCTTTTAAACTA-AAAAAGATCTCTTTGGTCTCTT  941
trnR  ------------------------------------------------   10
trnK  TAAATAGCACTTGACTTTTAATCAAGAGATAG-TATAATTA-TTTCTA   56
```

trnK

1.93 NC_015076-NC_006917

Avg ovsize: -311
LCA: Jenkinsia-genus

1.93.1 trnI-trnM 1

trnI *trnM*

```
R     GACCGCTTAAGGGGCACTTTGATAGAGTGACTAAAGGGGGTTGAACTCCCCCCAGCTCCTAAA-TATGGTAAGCTAAAAAAGCTTTCGGGCCCATACCCCGAATATGTTGGTTA-AACCCCC  119
trnI  GAATGTCTAAGGACCACTTTGATAGGGTGCGTTATGAGGGTTAAAGTCCCTCTGACTCCTCA-----------------------------------------------------------   62
trnM  -------------------------------------------------------------AGTAGGGTCAGCTAAATAAGCTTTTGGGCCCATACCCCAAACATGTTGGTTAGAATCCTT   60
```

trnI

trnM

1.93.2 nad1-trnQ -4

```
                                      nad1                                                            trnQ
R     T-TCGTCGTCTGACACCTTTCCTTCATTGTCAGCACTGCCGCACTTCCCCCTCAATT-TAGGAGATTAGAAAGCCGGGATTTGAACCCAGCCCGCAGAGATCAAAACTCTGAGTGCTCCCGCTA-   122
nad1  TCTTGTCCTCTGGCACATTGCCCTCCCCGTCGGGGCCGCTGGTCTCCCACCCCAGTTCTA-----------------------------------------------------------------    60
trnQ  ----------------------------------------------------------------ATTAGAAAGAAGGGACTCGAACCCATCCTCTAGAGATCAAAACTCTAGGTGCTTCCACTAC    61
                                     nad1
                                                                                                      trnQ
```

1.93.3 trnQ-trnI -930

```
                                     trnQ
R     GGATTTGAACCCAGCCCGCAGAGATCAAAACTCTGAGTGCTCCCGCTACACCACTCTCTGAATTGTTACCGCCAGGCTCTGCCCCGCCCGGACGTCCTCCCTTTCTGGTGATTAATATATACTATGTA   128
trnQ  GGACTCGAACCCATCCTCTAGAGATCAAAACTCTAGGTGCTTCCACTACACCACTTTCT---------------------------------------------------------------------    59
trnI  --------------------------------------------------------------------------------------------------------------------------------     0
                                     trnQ

R     TTTACACCATAAATTTATTTCAGGTACTATCAAGAAACCATTAATACTAAGAAACACCAGGTACCCTATAAGATTTATCAACTTAGTGTGGAATAAATGAATATTTGATCATTAATCCCCATAATCTT   256
trnQ  --------------------------------------------------------------------------------------------------------------------------------    59
trnI  --------------------------------------------------------------------------------------------------------------------------------     0

R     ATATGAATGCATATTACTCTCTATCAACATCCCTAATTCTACAAATATAATGCGCAGTAAGAAATCAGCAACCCATATCTAATTGCATATCATGAATGATAGGGTCAGGGACTTACGGTGTGGGGGTT   384
trnQ  --------------------------------------------------------------------------------------------------------------------------------    59
trnI  --------------------------------------------------------------------------------------------------------------------------------     0

R     ACACAGAATGAACTATTACTGGCATCTGGTTCCTATTTCAGGGCCATACTAGTATATTCCCCCATCAACTGAATTAAATTTGCATAAGTTAATGGTGTAGGACTAACGGTTCTTTACTCCCCATGCCA   512
trnQ  --------------------------------------------------------------------------------------------------------------------------------    59
trnI  --------------------------------------------------------------------------------------------------------------------------------     0
```

```
R     AGCGTTCTCTATGCGACATCTGGTTCTTTTTATTTCCGGGTCACTTTCAAATTGCATTTGGCGACTCCTTCCTAATGTTATTAGCGAAGGTGGCACTACACTTTGCCTGAGTAAACAACTGTAACACT  640
trnQ  --------------------------------------------------------------------------------------------------------------------------------   59
trnI  --------------------------------------------------------------------------------------------------------------------------------    0

R     CTATAGACTAAAATAGAAGACTTGCATAAGTAATATCAAGTACATAAGGTTATTCCTTAATCATCTATCTACTCGTAGAATGCCCCGGGGTGTATGGTTAAAGGAATTTGCTCGGCAAACCCCCCCTA  768
trnQ  --------------------------------------------------------------------------------------------------------------------------------   59
trnI  --------------------------------------------------------------------------------------------------------------------------------    0

R     CCCCCCCTTAATCCGAAAGAACTTTGTTGTCTCCTGTCAAACCCCTAAACCAGGAAAAATTCAGACAGATCGTCACTGGGTTAAAATGTGAAATGAAGGTATGTGAGTATAATAATATAAAAAAATTC  896
trnQ  --------------------------------------------------------------------------------------------------------------------------------   59
trnI  --------------------------------------------------------------------------------------------------------------------------------    0

                                                                                                                       trnI
R     ATGCCCAAAGCCCCCTCTTTACCTGCAATTCTTCGGCCGAACCCCCCCCATTTTCCCCCTTGGGCCGAAGGATTCTTGTTTAAGGCGGAACTGTGCCTGACCGCTTAAGGGGCACTTTGATAGAGTGA  1024
trnQ  --------------------------------------------------------------------------------------------------------------------------------   59
trnI  -----------------------------------------------------------------------------------------------TGCCTGAATGTCTAAGGACCACTTTGATAGGGTGC   35
                                                                                                                           trnI

       trnI
R     CTAAAGGGGGTTGAACT-  1041
trnQ  ------------------    59
trnI  GTTATGAGGGTTAAAGTC    53
       trnI
```

1.94 NC_023228-NC_008327

Avg ovsize: -314

LCA: Soleoidei-suborder

1.94.1 trnI-trnM -10

trnI *trnM*

```
R     TCTGAATAAAGAGTCACTTTGATAGAGTGAAGCACGTAGATGAAAACCCTACTAGCTCCTTAAAAGTTACGGTAAGCTAAAACAAGCTATTGGGCCCATACCCCAGCCATATAGGTTAAAACCC--  124
trnI  TGAAGCTAAAGGGCCACTTTGATAGAGTGAACCATGGGGGTTAAAATCCCCCTAACTCCT------------------------------------------------------------------   60
trnM  ----------------------------------------------------------------------GGTCAGCTAAT-CAAGCTGTAGGGCCCATACCCCTAAAATGTAGGTTAAACCCCTA   55
```

trnI

trnM

1.94.2 trnQ-trnI -12

trnQ *trnI*

```
R     TTATGGAGTGCACAAAGAGTTTTGATCTCTTAAGTTGAGGTTCGAATC-CTCCTTTCCTATATATTATCCCCAGGAGCTATGTCTGAA--TAAAGAGTCACTTTGATAGAGTGAAGCACGTAGATGAA  125
trnQ  GTAAGGGGAGCACGAAGAGTTTTGATCTCTTCAGTAAGGGGTCGATTCCCTTCTTTC-TA--------------------------------------------------------------------   59
trnI  ------------------------------------------------------------------------AGGAGCTATGCCTGAAGCTAAAGGGCCACTTTGATAGAGTGAACCATGGGGGTTAA   56
```

trnQ

trnI

trnI

```
R     AACCCT  131
trnQ  ------   59
trnI  AATCC-   61
```

trnI

1.94.3 nad1-trnQ -922

nad1

```
R     TTCGTGATA-GTAACCCTAATAATTCTAGTTATAACAGCAGGTATTCCTCCAATAGACTAATATTTTACATATATGTAATATTTTACATATATGTAATATTTTACATATATGTAATATTTTGCATATA   127
nad1  ATAGTTATTTGGAATC-TAGCTATTC------------------------------------------------------------------------------------------------------   25
trnQ  --------------------------------------------------------------------------------------------------------------------------------   0
```

nad1

```
R     TGTAATATTTTACATATATGTAATATTTTACATATATGTAATATTTTACATATATGTAATATTTTACATATATGTAATATTTTACATGTATGTAATATTTTACATGTATGTAATAGAACATAATACAT   255
nad1  --------------------------------------------------------------------------------------------------------------------------------   25
trnQ  --------------------------------------------------------------------------------------------------------------------------------   0

R     TCCACTCATAAACTTACTAATAGATGTATTAAACGCCCATAGGTATATCATATATTATATATCAGTATATCACCTACATAGTAAATTAATAACCATACATTGGATTTCAAGAATCAAGCTCCTTACAT   383
nad1  --------------------------------------------------------------------------------------------------------------------------------   25
trnQ  --------------------------------------------------------------------------------------------------------------------------------   0

R     TCTCAAGATCTTATCTTGAGAGAATAGTATTCTCTCTCTACTTTATGCTAGTATGCGGGATCCCATCATGTACACCTCCTACTTATCTAGTCAGTTCTTATTTTATAACATACCTTGCATGCTGTTAA   511
nad1  --------------------------------------------------------------------------------------------------------------------------------   25
trnQ  --------------------------------------------------------------------------------------------------------------------------------   0

R     TAAAGTTCTACACATACTTGACATGAAGTGTTCTCCAGGGGATAAGGGGTTTTCCTCTCTCCCTTGTTGACTCGACTAGTGTCTGCTTAGAAGAATATATCTGGGTAACTCATAACCAATTTCATCTA   639
nad1  --------------------------------------------------------------------------------------------------------------------------------   25
trnQ  --------------------------------------------------------------------------------------------------------------------------------   0

R     TTACTACAAAAGACATACAGACATTTATTGGAACACTACATAAGTCCTATAAACATACATTTTTACTGTCTTTATATCCGCCCGCCTTAATACAAACTATAGTCCTATACACAGTAATAGAAATCCCG   767
nad1  --------------------------------------------------------------------------------------------------------------------------------   25
trnQ  --------------------------------------------------------------------------------------------------------------------------------   0

R     GACTCTTTAATTTACCTCAGTGATTTCGACATAAAAAATTTTCCATCTTACATCTCTCAAAGAAGCAACAAATATTAAACAAAATTTACTAAGCCTAAAAATTTTTTGAGATTATTCGATTATAAAAA   895
nad1  --------------------------------------------------------------------------------------------------------------------------------   25
trnQ  --------------------------------------------------------------------------------------------------------------------------------   0
```

trnQ

```
R     AATCAAAAAACAAGCTACTAAAATATCACAAACCAAGTAACTTTGAAAATTTAGGAAATAGTGTTATGGAGTGCACAAAGAGTTTTGATCTCTTAAGTTGAGGTTCGAAT--  1005
nad1  ----------------------------------------------------------------------------------------------------------------    25
trnQ  ----------------------------------------------------AGGAAGTGGTGTAAGGG-GAGCACGAAGAGTTTTGATCTCTTCAGTAAGGGGTCGATTCC    59
```

trnQ

1.95 NC_015076-NC_008683

Avg ovsize: -327

LCA: Otocephala-clade

1.95.1 rrnS-trnV 1

rrnS *trnV*

```
R     GCCTCAAGGGGAGGCAAGTCGTAACATGGTAAGTGTACCGGAAGGTGCACTTGGAAGAATCAGAGTGTGGCTGAGACAGATAGGCATCTCCCTTACACCGAGAATACACCCAAGCGA-TT  119
rrnS  GAC--AAGGGGAGGCAAGTCGTAACATGGTAAGTGTACCGGAAGGTGCACTTGGATTAAAC-----------------------------------------------------------   59
trnV  ------------------------------------------------------------CAGGGCGTGGCTGAGTTAGTCAAGCATCTCACTTACACCGAGAAGACATCCATGCAAGTT   60
```

rrnS

trnV

1.95.2 trnI-trnM -5

```
                                   trnI                                                    trnM
R     GACCGCTTAAGGGGCACTTTGATAGAGTGA-CTAAAGGGGGTTGAACTCCCCCCAGCTCCTAATATGGTAAGCTAAAAAAGCTTTCGGGCCCATACCCCGAATATGTTGGTTAAA-CCCCC   119
trnI  GAATGCCTAGGGACCACTTTGATAGAGTGGCCTATA-GGGGCTAAAATCCCCTCAGTTCTT------------------------------------------------------------    60
trnM  ------------------------------------------------------------------GGTCAGCTAATCAAGCTTTCGGGCCCATACCCCGAACATGACGGTTAAAGTCCCT    55
                                   trnI                                                    trnM
```

1.95.3 trnQ-trnI -979

```
                                   trnQ
R     GGATTTGAACCCA-GCCCGCAGAGATCAAAACTCTGAGTGCTCCCGCTACACCACTCTCTGAATTGTTACCGCCAGGCTCTGCCCCGCCCGGACGTCCTCCCTTTCTGGTGATTAATATATACTATGT   127
trnQ  GGGATCGAACCCATGCCCA-AGAGATCAAAACTCTTAGTGCTTCCTCTACACCACTTTCT-AA-----------------------------------------------------------------    61
trnI  --------------------------------------------------------------------------------------------------------------------------------     0
                                   trnQ

R     ATTTACACCATAAATTTATTTCAGGTACTATCAAGAAACCATTAATACTAAGAAACACCAGGTACCCTATAAGATTTATCAACTTAGTGTGGAATAAATGAATATTTGATCATTAATCCCCATAATCT   255
trnQ  --------------------------------------------------------------------------------------------------------------------------------    61
trnI  --------------------------------------------------------------------------------------------------------------------------------     0

R     TATATGAATGCATATTACTCTCTATCAACATCCCTAATTCTACAAATATAATGCGCAGTAAGAAATCAGCAACCCATATCTAATTGCATATCATGAATGATAGGGTCAGGGACTTACGGTGTGGGGGT   383
trnQ  --------------------------------------------------------------------------------------------------------------------------------    61
trnI  --------------------------------------------------------------------------------------------------------------------------------     0

R     TACACAGAATGAACTATTACTGGCATCTGGTTCCTATTTCAGGGCCATACTAGTATATTCCCCCATCAACTGAATTAAATTTGCATAAGTTAATGGTGTAGGACTAACGGTTCTTTACTCCCCATGCC   511
trnQ  --------------------------------------------------------------------------------------------------------------------------------    61
trnI  --------------------------------------------------------------------------------------------------------------------------------     0
```

```
R     AAGCGTTCTCTATGCGACATCTGGTTCTTTTTATTTCCGGGTCACTTTCAAATTGCATTTGGCGACTCCTTCCTAATGTTATTAGCGAAGGTGGCACTACACTTTGCCTGAGTAAACAACTGTAACAC   639
trnQ  --------------------------------------------------------------------------------------------------------------------------------    61
trnI  --------------------------------------------------------------------------------------------------------------------------------     0

R     TCTATAGACTAAAATAGAAGACTTGCATAAGTAATATCAAGTACATAAGGTTATTCCTTAATCATCTATCTACTCGTAGAATGCCCCGGGGTGTATGGTTAAAGGAATTTGCTCGGCAAACCCCCCCT   767
trnQ  --------------------------------------------------------------------------------------------------------------------------------    61
trnI  --------------------------------------------------------------------------------------------------------------------------------     0

R     ACCCCCCCTTAATCCGAAAGAACTTTGTTGTCTCCTGTCAAACCCCTAAACCAGGAAAAATTCAGACAGATCGTCACTGGGTTAAAATGTGAAATGAAGGTATGTGAGTATAATAATATAAAAAAATT   895
trnQ  --------------------------------------------------------------------------------------------------------------------------------    61
trnI  --------------------------------------------------------------------------------------------------------------------------------     0

                                                                                                                 trnI
R     CATGCCCAAAGCCCCCTCTTTACCTGCAATTCTTCGGCCGAACCCCCCCCATTTTCCCCCTTGGGCCGAAGGATTCTTGTTTAAGGCGGAACTGTGCCTGACCGCTTAAGGGGCACTTTGATAGAGTG  1023
trnQ  --------------------------------------------------------------------------------------------------------------------------------    61
trnI  --------------------------------------------------------------------------------------------------------------------------------     0

             trnI
R     ACTAAAGGGGGTTGAACT  1041
trnQ  ------------------    61
trnI  ------------------     0
```

1.96 NC_009584-NC_009585

Avg ovsize: -331

LCA: Ilisha-genus

1.96.1 trnT-trnP 1

```
                     trnT                                                    trnP
R     CTTAACCCAAAGCGCCGGTCTTGTAATCCGGAGATCGGAGGTTAAAATCCCCCCTAAGGCCAAAGAAGAAAGATTTTAACTTTCACCTCTAACTCCCAAAGCTAGAATTCTAAACTAA  118
trnT  -TTAACTCAAAGCGCCGGTCTTGTAATCCGGAGATCGGAGGTTAAAATCCCCCCTGAGGCCAGAGAAAAAAGATTTTAACTTCCACCTCTAACTCCCAAAGCTAGAATTCTAAACTAA  117
trnP  ---------------------------------------------------------------------------------------------------------------------A    1
                      trnT
                                                                                                                     trnP
```

1.96.2 cob-trnT -1

```
                     cob                                                        trnT
R     TATTTTTAATCCTCACCCCCCTAGCAGGCTGATTAGAAAACAAAGCCTTAGAATGAAACTGCCCTAGTAGCTTAACCCAAAGCGCCGGTCTTGTAATCCGGAGATCGGAGGTTAAAAT-C  119
cob   TATTTCTAATTCTCACCCCCCTAGCAGGATGACTAGAAAACAAAGCCCTAGAATGAAACT------------------------------------------------------------   60
trnT  -------------------------------------------------------------CCCCAGTAGCTTAACTCAAAGCGCCGGTCTTGTAATCCGGAGATCGGAGGTTAAAATCC   59
                     cob
                                                                                trnT
```

1.96.3 trnP-trnF -995

```
                     trnP
R     AAGATTTTAACTTTCACCTCTAACTCCCAAAGCTAGAATTCTAAACTAAACTATTCTCTGAGCGCCCCACTTAGTCCTTTAACAAGATTAGTAAACCTTCAGGACCTGTAATATGGTATCTGGCAGGG  128
trnP  AAGATTTTAACTTCCACCTCTAACTCCCAAAGCTAGAATTCTAAACTAAACTATTCTCTG--------------------------------------------------------------------   60
trnF  --------------------------------------------------------------------------------------------------------------------------------    0
                     trnP
```

```
R     AGGTTTATAAATGTTTCAATGCCTAAAACTTTATGTCCATTATAAATTAGAATATTTCCTTCAACATCAATGGGGATAGCACAAATTACAATGTATGACTCCATATTACATGGTGTAGTACATATATG  256
trnP  --------------------------------------------------------------------------------------------------------------------------------  60
trnF  --------------------------------------------------------------------------------------------------------------------------------  0

R     GTGTTTATACATGCTATGTATAATCACCATGGATTTATTTGAACCAAAGCAAGTAATAGATATTACTAAGACATTCATAAAGCATACTATTAGAATTCAGAATTTAGTCGATCACAACTGATAAATAT  384
trnP  --------------------------------------------------------------------------------------------------------------------------------  60
trnF  --------------------------------------------------------------------------------------------------------------------------------  0

R     AATAATCCCCATAAAATTTACTACAACATTTTCTATGCCCTTTTCAATATTAATAGTTTTATCTATATTTAATGTAGTAAGAAATCACCAACCAGTTTATATAAAGGCATATCATGAATGATAGGGTC  512
trnP  --------------------------------------------------------------------------------------------------------------------------------  60
trnF  --------------------------------------------------------------------------------------------------------------------------------  0

R     AGGGACAATAATCGTGGGGGTTTCACAAAATGAACTATTTCTGGCATCTGGTTCCTATTTCAGGGTCATACATCTCTTAATCCTCCCATAGTGAATTATATCTGACATAAGTTAATGGTGGTATTACT  640
trnP  --------------------------------------------------------------------------------------------------------------------------------  60
trnF  --------------------------------------------------------------------------------------------------------------------------------  0

R     AATGGTTCTTTACCCCACATGCCGAGCGTTCATTTATATGCATAGGGTATCTTTTTTTTCGGGTCACTTTCACTAACATTTGACGACTCCTTCCTAATGTTAACTGACAAGGTTGAACATTTCCTTGC  768
trnP  --------------------------------------------------------------------------------------------------------------------------------  60
trnF  --------------------------------------------------------------------------------------------------------------------------------  0

R     TTGCAAGTATTAACTGTTCAATACTTCACCAACATTGATAGAAGAACCACATAAGTGATATCAGGTGCATAAAGTACTTATCTTTACTCCATACTTTCCTGTATACTGTGCCCCCCCTCCTTTTTACG  896
trnP  --------------------------------------------------------------------------------------------------------------------------------  60
trnF  --------------------------------------------------------------------------------------------------------------------------------  0

R     TGAAGGTTTTTGCGCGACAAACCCCCTTTCCCCCTTGCGCCGAGAGAACCTTGTTTATTCCTGTCAAACCCCAAAACCAGGGAAGATTCGACTTGGCGCTCAACAAATTTCGATATGCGTTGACTATA  1024
trnP  --------------------------------------------------------------------------------------------------------------------------------  60
trnF  --------------------------------------------------------------------------------------------------------------------------------  0
```

```
                                         trnF
R      CAGTGTGCCACTAGTGTGTCACACTATTAAACTCATGTAGCTTAAACCAAAGCATGACACTGAAGATGTTAAGACGGGCCCTAGAAAG-C  1113
trnP   ------------------------------------------------------------------------------------------   60
trnF   ------------------------------CTCACGTAGCTTAAACTAAAGCATGACACTGAAGATGTTAAGACGGGCCCTAGAAAGCC   59
                                          trnF
```

1.97 NC_006355-NC_004373

Avg ovsize: -339

LCA: Holacanthopterygii-superorder

1.97.1 trnI-trnM -43

```
                                trnI                                                              trnM
R      CTGAATCAAAGGGTCACTTTGATAGAGTGAAGCATGGGGGTTAAAATCCTCCCAACTCCTTCCACGGAGCAAAGTAAGCTAATAGAAGCTATTGGGCCCATACCCCAAAAATGTTGACTAATACTC  126
trnI   CTGAATTAAAGGGCCGCTTTGATAGAGCGTAACATGGGGGTTAGATTCCCCCCAACTCCT------------------------------------------------------------------   60
trnM   ------------------------------------------------------------------------------------------------------CCCA-AAAGCTTGTCT--TAG-C   19
                                trnI
                                                                                                               trnM
```

1.97.2 trnM-trnQ -56

```
      trnM                                                                                                                        trnQ
R     GCTAATAGAAGCTATTGGGCCCATACCCCAAAAATGTTGACTAATACTCAGCCTTTGTTACTTTTCCCCATAGAAAAAAGGGGCTCGAACCCTTCCTGAAGAGATCAAAACTCTTGGTGCTTCCACTA   128
trnM  AGGAGTTAAACCTA------------------------------------------------------------------------------------------------------------------    14
trnQ  ----------------------------------------------------------------------TAGAAAGGGAGGGCTCGAACCTCCCCCGGGGAGATCAAAACTCCCAGTGCTCCCACTA    58
      trnM                                                                                                                        trnQ
```

```
R     -   128
trnM  -    14
trnQ  C    59
      trnQ
```

1.97.3 trnP-trnF -918

```
      trnP
R     -GATTTAAACCTC-CACCTCTAGCTCCCAAAGCTAGTATCCTGTCAGTTAAACGATTCTCTGGCAG-TGCATATATACTGTATTATTATCATACCATTATTTTAACCAAAATATGCATAGCTCCCAAG   125
trnP  AGATTTTAA-CTCTCACTACTAGCCCCCAAAACTAATATTCTAAC-GGTAAACTACCCTCTGCTATATGCA---------------------------------------------------------    69
trnF  --------------------------------------------------------------------------------------------------------------------------------     0
      trnP
```

```
R     GGCATACATGTATATTCACCATTAAAATAACTTAAACATTCATACATCACCACTAAACCAAGGTTTACGCAAAGCTAAACTTGAAGATTTACCATTCCCCCAAATCAACTGAGAGATTTAAGACTTAA   253
trnP  --------------------------------------------------------------------------------------------------------------------------------    69
trnF  --------------------------------------------------------------------------------------------------------------------------------     0
```

```
R     CCCCCTAGTCCAAATCACTACACAAACCAAGTACCCACATCCCGTTCAATTGTTGTTGACTTGCGCAGTAAGAACCGACCAACAAGCACATATCTTAACGCATTCGTTTCTTGATGGTCAGGGATAAC   381
trnP  --------------------------------------------------------------------------------------------------------------------------------    69
trnF  --------------------------------------------------------------------------------------------------------------------------------     0

R     CCGCCGTGGGGGTCACTAGCCTCACACTATTACTGGCATTTGGTTCCTATTTCAGGAACATTCCCTACTTTCACCCCATCCGTTTATCGAACCTTGCATAAGTTAATGCGGTGAATACTTATTCCCCG   509
trnP  --------------------------------------------------------------------------------------------------------------------------------    69
trnF  --------------------------------------------------------------------------------------------------------------------------------     0

R     TTACCCAACATGCCGAGCGCTCTCTCCACAGGTACAAGGGGTTTTCCTTTTTGGTTTCCTTTCATTTTGCATTTCACAGTGCACCCTAAAATGACCGAAAAGGTAGAACATTCTTCTTGCACGCCGGG   637
trnP  --------------------------------------------------------------------------------------------------------------------------------    69
trnF  --------------------------------------------------------------------------------------------------------------------------------     0

R     CGGTAATTGAATGATGTAAAGATATTACACAAAGAATTGCATTAATAGCTATCAAGAGCATAATGCTTACTTCCTACCTAGGAGATTTAGTGCCCCGGGGTGTTTCCACTAAAATAAATCTTTGCCCC   765
trnP  --------------------------------------------------------------------------------------------------------------------------------    69
trnF  --------------------------------------------------------------------------------------------------------------------------------     0

R     CCTTCCCCCCGCCCCCAACTCACTATTAAATTCATCACCCGTCTCTAAAACAACTATTTTCCTAAGTTAAGCCCCCGTTTGCCCTCTTCCCCCTAAATCATATTACATAAATTATTATATTCATTACA   893
trnP  --------------------------------------------------------------------------------------------------------------------------------    69
trnF  --------------------------------------------------------------------------------------------------------------------------------     0

                                                                                                                   trnF
R     AAATTGGAAAATTTTCTCCAAATTACAATGATGTAAATAATTTCATCAAGACAACTTCCACCAAATTTCAATCGAATTTTCCCAACAGCTGTCGTAGCTTAACAAAAGCATAACACTGAAGATGTTAA  1021
trnP  --------------------------------------------------------------------------------------------------------------------------------    69
trnF  ---------------------------------------------------------------------------------------------GTAGCTCAACTAAAGCATAACATTGAAGTTGTTAA    35
                                                                                                                     trnF

        trnF
R     GATGGACCCTTAAAAGT-C  1039
trnP  -------------------    69
trnF  GATGGGACCTAGCCCCCCC    54
        trnF
```

1.98 NC_011381-NC_004373

Avg ovsize: -343

LCA: Holacanthopterygii-superorder

1.98.1 trnM-trnI -68

trnM, trnI (R)

```
R     AGCTAAATAAGCTTTTGGGCCCATACCCCAAACATGTTGGTTAAAATCCTTCCTTTACTAATGACGGAGATGTGCCTGAAATAAAAGGATCATTTTGATAGAATGCCTCATGAGGGTTTAAGT--C   124
trnM  AG------------------------------------------------------------------------------------------------------------------------------   2
trnI  ----------------------------------------------------------------------TGTGCCTGAATTAAAGGGC-CGCTTTGATAGAGCGTAACATGGGGGTTAGATTCCC   55
```

trnM, trnI

1.98.2 nad1-trnM -177

nad1 (R)

```
R     CTTGTAATCTGACATCTCTCATTACCAATTGCCTTCTTTGGTCTTCCCCCCCAACTTTAATATTTACAGAGATATGCCTGAAATAAAGGATCCCCTTGATAGAGTGCCCCTTAGAATAAAAGAAGTTT   128
nad1  TTTATTATTTGACACCTGGCCCTCGGAGTTGCCCTTACGGGTCTGCCTCC------------------------------------------------------------------------------   50
trnM  --------------------------------------------------------------------------------------------------------------------------------   0
```

nad1

trnM (R)

```
R     ATAACCCCACCTGAGGGGATTAAAACTCTTAGTACTTCCTAGTAAAGTCAGCTAAATAAGCTTTTGGGCCCATACCCCAAACATGTTGGTTAAAATCCT   227
nad1  ---------------------------------------------------------------------------------------------------   50
trnM  ---------------------------------------------------------------------------------------------------   0
```

1.98.3 trnP-trnF -785

```
                                       trnP
R     GGGAGTTTAACCCCCACCCCTAGCTCCCAAAGCTAGAGCTCTAA-GTTAAACTATTCTTTGCCG-A-GCTCCGCCTCCACGCAAGTACATATGAACGTA-ATGTACATATATGTATTATCCCCAT-TA   123
trnP  AG-ATTTTAACTCTCACTACTAGCCCCCAAAACTAAATATTCTAACGGTAAACTACCCTCTGCTATATGCAACGTGACCGCTACAGTACCCACACACATACATGTACACATCTAT-TTATATAGATATA   126
trnF  --------------------------------------------------------------------------------------------------------------------------------     0
                                       trnP

R     -ATAGA-ATTTAACCATATATAAGTAATGTAATACTACATTAACTGAAGACACATAAATATTTGATATCAAACATATAAATAACCAAAATACTATAAATTTTACATAAACCATTATAAGTTCAAACAT   249
trnP  CATATATACTTATGCATTTATA----------------------------------------------------------------------------------------------------------   148
trnF  --------------------------------------------------------------------------------------------------------------------------------     0

R     ACAAGAATTGATATACCTTTAGCGAAATTGAATTGCCCTAGCTAATAAACCCATTAGTCTAGTTATACCAAGATTCAACATCCCTAAAAGACAAAATCCGATGCAGTAAGAGACCACCATCAGTTGAT   377
trnP  --------------------------------------------------------------------------------------------------------------------------------   148
trnF  --------------------------------------------------------------------------------------------------------------------------------     0

R     TTCTAAATGCATACGGTTCTTGATGGTCAGGGACAATAATTGTGGGGGTTTCACTTAATGAACTATTCCTTGCATTTGGTTCCTATTTCAGGAACATAAATTGAAAATACCCCATTCTTTCATTGAAA   505
trnP  --------------------------------------------------------------------------------------------------------------------------------   148
trnF  --------------------------------------------------------------------------------------------------------------------------------     0

R     ATTGCATAAGTTAATGGTGGAGTACATTCTACTCGTTACCCACCATGCCGAGCGCTCTTTCCATAGGGCAGCTGGTTCTCTTTTTTAGGTTTCCTTTCAATTGACATTTCAGAGTGCACCCTAAAATG   633
trnP  --------------------------------------------------------------------------------------------------------------------------------   148
trnF  --------------------------------------------------------------------------------------------------------------------------------     0

R     TTAAATTAAGGTTGAACATTTCCTTGTAATAATTAATATATGTGAATTCTTAAAAGACTTTAATTTATGAATTGCATAATTGATATCATGAGCATAATGAATGAAATATTCTCCTAACTTTTCTCTTA   761
trnP  --------------------------------------------------------------------------------------------------------------------------------   148
trnF  --------------------------------------------------------------------------------------------------------------------------------     0

R     AAACGCCCCCTCGGCTTTTACGCGTTAAACCCCCCCTACCCCCCCACTTCTCGAGACATCATTATAAATCCTGCAAACCCCCCGGAAAACAGGAAAGCCTCTACCAGTGGTTTTTAACCTCTAAAGTA   889
trnP  --------------------------------------------------------------------------------------------------------------------------------   148
trnF  --------------------------------------------------------------------------------------------------------------------------------     0
```

```
                                                                        trnF
R     TTTTTGATGATATGATATTGTAATATTACAAATGCTAATGTAGCTTAATTAAAGTATAACACTGAAGATGTTAAGATGGGCCCTAAAAAGCC-   981
trnP  ---------------------------------------------------------------------------------------------   148
trnF  ---------------------------------------GTAGCTCAACTAAAGCATAACATTGAAGTTGTTAAGATGGGACCTAGCCCCCCC    54
                                                                        trnF
```

1.99 NC_023228-NC_005961

Avg ovsize: -347

LCA: Teleostomi-superclass

1.99.1 trnQ-trnI -15

```
                              trnQ                                                               trnI
R     TTATGGAGT-GCACAAAGAGTTTTGATCTCTTAAGTTGAGG-TTCGAATCCTCCTTTCCTATATATTATCCCCA-GGA-GCTATGTCTGAATAAAGAGTC-ACTTTGATAGAGTGAAGCACGTAG-AT   122
trnQ  A-ATGAGGTAGTACTGGAAGTTTTGATCT-TTCAGGTGCGGGTTCGAGTCCTGCTT------------------------------------------------------------------------    54
trnI  -----------------------------------------------------------------------CCACGGAAGC-GTGCCTGAGAAAAG-GACTACCTTGATAAAGT-AAAAACAGAGCAT    54
                          trnQ
                                                                                                   trnI
```

```
        trnI
R     GAAAACCCT-   131
trnQ  ----------    54
trnI  TGAACCTCTC    64
        trnI
```

1.99.2 trnW-trnA -54

```
                                  trnW                                                      trnA
R     AGGTTATTTGACCAAGAGCCTTCAAAGCTCTAAGAAAGGGTGAAAATCCCTTAGTCTTTGGATAAAACTTGCGGGATACTACCCCACATCTTCTGCTTGCAAAGCAGACACTTTAATTAAG---   121
trnW  -GGTTAT---------------------------------------------------------------------------------------------------------------------     6
trnA  -------------------------------------------------------------ATAGAACTCGCGACA-ACT-CATCACATCTCCTGCATGCAACACAGACACTTTAATTAAGCCA    61
       trnW                                                                                 trnA
```

1.99.3 nad1-trnQ -973

```
                                  nad1
R     TTCGTGATAGTAACCCTAATAATTCTAGTTATAACAGCAGGTATTCCTCCAATAGACTAATATTTTACATATATGTAATATTTTACATATATGTAATATTTTACATATATGTAATATTTTGCATATAT   128
nad1  CT------------------------------------------------------------------------------------------------------------------------------     2
trnQ  --------------------------------------------------------------------------------------------------------------------------------     0
     nad1

R     GTAATATTTTACATATATGTAATATTTTACATATATGTAATATTTTACATATATGTAATATTTTACATATATGTAATATTTTACATGTATGTAATATTTTACATGTATGTAATAGAACATAATACATT   256
nad1  --------------------------------------------------------------------------------------------------------------------------------     2
trnQ  --------------------------------------------------------------------------------------------------------------------------------     0

R     CCACTCATAAACTTACTAATAGATGTATTAAACGCCCATAGGTATATCATATATTATATATCAGTATATCACCTACATAGTAAATTAATAACCATACATTGGATTTCAAGAATCAAGCTCCTTACATT   384
nad1  --------------------------------------------------------------------------------------------------------------------------------     2
trnQ  --------------------------------------------------------------------------------------------------------------------------------     0

R     CTCAAGATCTTATCTTGAGAGAATAGTATTCTCTCTCTACTTTATGCTAGTATGCGGGATCCCATCATGTACACCTCCTACTTATCTAGTCAGTTCTTATTTTATAACATACCTTGCATGCTGTTAAT   512
nad1  --------------------------------------------------------------------------------------------------------------------------------     2
trnQ  --------------------------------------------------------------------------------------------------------------------------------     0
```

```
R     AAAGTTCTACACATACTTGACATGAAGTGTTCTCCAGGGGATAAGGGGTTTTCCTCTCTCCCTTGTTGACTCGACTAGTGTCTGCTTAGAAGAATATATCTGGGTAACTCATAACCAATTTCATCTAT  640
nad1  --------------------------------------------------------------------------------------------------------------------------------    2
trnQ  --------------------------------------------------------------------------------------------------------------------------------    0

R     TACTACAAAAGACATACAGACATTTATTGGAACACTACATAAGTCCTATAAACATACATTTTTACTGTCTTTATATCCGCCCGCCTTAATACAAACTATAGTCCTATACACAGTAATAGAAATCCCGG  768
nad1  --------------------------------------------------------------------------------------------------------------------------------    2
trnQ  --------------------------------------------------------------------------------------------------------------------------------    0

R     ACTCTTTAATTTACCTCAGTGATTTCGACATAAAAAAATTTTCCATCTTACATCTCTCAAAGAAGCAACAAATATTAAACAAAATTTACTAAGCCTAAAAATTTTTTGAGATTATTCGATTATAAAAAA  896
nad1  --------------------------------------------------------------------------------------------------------------------------------    2
trnQ  --------------------------------------------------------------------------------------------------------------------------------    0

                                                                                      trnQ
R     ATCAAAAAACAAGCTACTAAAATATCACAAACCAAGTAACTTTGAAAATTTAGGAAATAGTGTTATGGAGTGCACAAAGAGTTTTGATCTCTTAAGTTGAGG-TTCGAAT  1005
nad1  --------------------------------------------------------------------------------------------------------------    2
trnQ  -------------------------------------------------------------------------------AGTTTTGATCT-TTCAGGTGCGGGTTCGAGT    30
                                                                                                     trnQ
```

1.100 NC_004377-NC_008225

Avg ovsize: -350

LCA: Gadiformes-order

1.100.1 nad6-trnE 61

```
R     AGAAGCAACAAAAATTACCCCTAAATTTATTACTGCT-AAAACAAC-TAATAGA-TATATCATCATTCCCACCTGAATTTTCATC-AGGACCACTGACTTGAAAAACCAACGTTGTAATTCAA---   119
nad6  TGC--CAACGC-AACTGCTCCTAAAATAAACACCCCTGACAATATCATTATTGAATAAGACATAATCCCTGCCCGGGATTAAACCGAGAATAAAT-ATTTAAATATCCGCCTTTG-ACCTCA----   117
trnE  -------------------------------------------------------------ATAATTCTTGCCCGGGCTCTAACC-AGGACCAATGACTTGAAAAACCACCGTTG-A-TTCAACTA    62
```

1.100.2 trnE-cob -81

```
R     -C-TGAATTTTCATCAGGACCACTGACTTGAAAAACCAACGTTGTAATTCAACTACGGGAATATAACTTTGTTTATTACCCCCAACACACAAATTAAATCTGTAAGTCACCACTTATGCTAGATTAAT   126
trnE  GCCCGGGCTCTAACCAGGACCAATGACTTGAAAAACCACCGTTG--ATTCAACTACAAGAA-------------------------------------------------------------------    59
cob   --------------------------------------------------------------------------------------------------------------------------------     0
```

```
R     TACACAGTGAAACCAATGACCAATTTACGAAAGACACACCCGCTCTTAAAAATTGTTAACGATGCTTTAATTGA-   200
trnE  ---------------------------------------------------------------------------    59
cob   --------------AATGACCAGCCTTCGAAAAACCCACCCCCTCCTAAAAATCGCAAATGATGCCGTAACAGAC    61
```

1.100.3 trnP-trnF -1032

```
                                         trnP
R     -AGATTTTAACTCCTGCCCCTAGCTCCCAAAGCT-AGAATTTTAAAACTTAACTATTCTCTGGCCCCAGAAATTTTGACTCGCCCCTACATTTAAAATATTCACCATAGACTATAGTAACTCTTCATG   126
trnP  GAGACTTAAACTCCCACCCCCAGCTCCCAAAGCTGAGATTCTTC--ATTAAACTATCCTCTGCAACCCCAAATAT-----------------------------------------------------    73
trnF  --------------------------------------------------------------------------------------------------------------------------------     0
                                         trnP

R     GTATAGTACATCTATGTATAACCACCATATACTTATTTTGACCATTCAAGAAATAAATACAATCAATTCATAAAAACTTTTCATTTAAATCTGATTAAAAACTAATTTAAACATACAAGAAATAACCA   254
trnP  --------------------------------------------------------------------------------------------------------------------------------    73
trnF  --------------------------------------------------------------------------------------------------------------------------------     0

R     CAATTAATTATTTAAGACTTAAATTTATTGAACAACTTATTAATTTTTAAGTACACGTGATTAATCGGCCTATCGTTTCTTTTAAGGTTTACTAAATCACCAGCTAGCGTGTATTTAAATGGTACACT   382
trnP  --------------------------------------------------------------------------------------------------------------------------------    73
trnF  --------------------------------------------------------------------------------------------------------------------------------     0

R     GTCCAATGAAGGTGAGGGGTACTTATTAGAAAATTCACCACTCCGTGAATTTTTCCTGGCATCTCTCCCTAGCTTCAGGGTCATAACTTCATACCGCTCACAGCTTGCTTTTTTGTCCATCTCTAAAT   510
trnP  --------------------------------------------------------------------------------------------------------------------------------    73
trnF  --------------------------------------------------------------------------------------------------------------------------------     0

R     GGAGTCGTTCATATTTCCAAATTTCCCACCATGCCGAGCATTCTCTCCATAGGGCTATTGGGTTCTTCTTTTTTTCTTTTCCCTTCATCAACCCCCCAGAGTGGAGTTATGAATAACTACCAGAGGT   638
trnP  --------------------------------------------------------------------------------------------------------------------------------    73
trnF  --------------------------------------------------------------------------------------------------------------------------------     0

R     AGGTCATTCCTTGAAATTTAAGACATTTTATTAAGGTTTTAAAGACTTTTTTTAGAGAATTACATTATATTATTTCAAGGGCATAACACAACTCCATTCGTTCGTCAAAAATTTCTTCAAAAACACA   766
trnP  --------------------------------------------------------------------------------------------------------------------------------    73
trnF  --------------------------------------------------------------------------------------------------------------------------------     0

R     AAATTTTTAAGGCCGTAAACCCCCCCTACCCCCCAGTTTTACGAGACTTGTAATTCCTGCAAACCCCCCGGAAACAGAAAAATCCCCAAACTGAGCTTACTTAATCGAAAGAACCTTTCAACTCCAAA   894
trnP  --------------------------------------------------------------------------------------------------------------------------------    73
trnF  --------------------------------------------------------------------------------------------------------------------------------     0
```

```
                                                                                                                                         
R     AATCTTTTACCCCTTGGATATTATTGACTATTAATAAAAACCCCTACGATCAAATTTTCCTACCCTTTAATAGGCTCGTTAATGGAAAAATATTACACTAAAACCACTTTTTAAAAAGCACAATGTTT  1022
trnP  --------------------------------------------------------------------------------------------------------------------------------    73
trnF  --------------------------------------------------------------------------------------------------------------------------------     0
```

```
                                                                                                          trnF
R     TTCCCTAAGGCCAAGTTTGACCCCAAGAATTGTGTTATTTTAATTTTAAAATTAAAACTAAAGTATTAAACTTTTTTTGCTGTCGTAGCTTAAATTTAAAGCTTAATGCTGAAGATATTAAGATGAAG  1150
trnP  --------------------------------------------------------------------------------------------------------------------------------    73
trnF  -----------------------------------------------------------------------------------CGTAACTTAA-TT-AAAGTTTAATATTGAAGCTATTAGGATGGGC    43
                                                                                                          trnF
```

```
       trnF
R     CCTAAAA--C-C  1159
trnP  ------------    73
trnF  CCTAAAAAGCCC    55
       trnF
```

1.101 NC_003159-NC_002647

Avg ovsize: -390

LCA: Stomiatiformes-order

1.101.1 trnN-trnY 0

```
                               trnN                                                                   trnY
R     CCTCGATCCCCCAAACTCTTAGTTAACAGCTAAGCGCCCCAACCAGCGAGCATCCATCTAACTGACC-AGCCTGGTAGGAAGAGGACTTAAACCTCTCTTTATGGGGCTACAACCCACCACTTAAATC   127
trnN  CCTCGATCCTACAAAGTCTTAGTTAACAGCTAAGCGCTCCAACCAGCGAGCATCAGTCT-ACTCTCCCCGCC--------------------------------------------------------    71
trnY  ------------------------------------------------------------------------TGGCAGGGAGAGGAGTTTAACCTCTGTTTATGGGGCTACAATCCACCGCTTAACAC    56
                               trnN                                                                   trnY
```

```
     trnY
R    TCA   130
trnN ---    71
trnY TCA    59
     trnY
```

1.101.2 trnY-trnC -79

```
                                       trnY
R    AGGACTTAAACCTCTCTTTATGGGGCTACAACCCACCACTTAAATCTCAGCCACCCTACCTTATACCCTGTTACGCCCCTGAACCACCTCAACACATGCTCCCCCATAGATTAACCACACTTATCGCC  128
trnY AGGAGTTTAACCTCTGTTTATGGGGCTACAATCCACCGCTTAACACTCAGCCACCCTACCT-------------------------------------------------------------------   61
trnC --------------------------------------------------------------------------------------------------------------------------------    0
                                       trnY
```

```
                                                                                      trnC
R    CACATAAGCAGGTCTCCTTTCGCCGCCGGG-CGTCGACG-GAAAGCCCCGGCAGGCTGGAA-ACCTGCTTCTTCAGATTTGCAATCTGACGTGTCACACCTC  227
trnY ------------------------------------------------------------------------------------------------------   61
trnC ------------TCTCCC--CGCCGCCGGGGCG-CGCGGGAAAGCCCCGGCGGGCGGTTAGGCCTGCTTCTTCAGGTTTGCAATCTGACGTGTCACACCTC   87
                                                                                     trnC
```

1.101.3 trnC-cox1 -1093

```
                                       trnC
R    CCGGCAGGCTGGAAA-CCTGCTTCTTCAGATTTGCAATCTGACGTGTCACACCTCAGGGCTTAAACTATGAACCCCGTAGCCCGCCCACAATGCTGCCCTGAAAGCCCTGACAGCACATGCTCACCCC  127
trnC -CGGCGGGCGGTTAGGCCTGCTTCTTCAGGTTTGCAATCTGACGTGTCACACCTCGGGGCT-------------------------------------------------------------------   60
cox1 --------------------------------------------------------------------------------------------------------------------------------    0
                                       trnC
```

```
R     CAGGGTTGGTGGCACTTTCTGGCCGCCCCCCCATAAGCAGGTCTCCTTTCGCCGCCGGGCGTCGACGGAAAGTCCCCGGCAGGCTGGAAACCTGCTTCTTCAGATTTGCAATCTGACGTGTCACACCT   255
trnC  --------------------------------------------------------------------------------------------------------------------------------    60
cox1  --------------------------------------------------------------------------------------------------------------------------------     0

R     CAGGGCTTAAACTATGAACCCCGTAGCCCGCCCACAATGCTGCCCTGAAAGCCCTGACAGCACATGCTCACCCCCAGGGTTGGTGGCACTTTCTGGCCGCCCCCCCATAAGCAGGTCTCCTTTCGCCG   383
trnC  --------------------------------------------------------------------------------------------------------------------------------    60
cox1  --------------------------------------------------------------------------------------------------------------------------------     0

R     CCGGGCGTCGACGGAAAGTCCCCGGCAGGCTGGAAACCTGCTTCTTCAGATTTGCAATCTGACGTGTCACACCTCAGGGCTTAAACTATGAACCCCGCAGCCCGCCCACAATGCTGCCCTGAAAGCCC   511
trnC  --------------------------------------------------------------------------------------------------------------------------------    60
cox1  --------------------------------------------------------------------------------------------------------------------------------     0

R     TGACAGCACATGCTCACCCCCAGGGTTGGTGGCACTTTCTGGCCACCCCCCATAAGCAGGTCTCCTTTCGCCGCCGGGCGTCGACGGAAAGCCCCGGCAGGCTGGAAACCTGCTTCTTCAGATTTGCA   639
trnC  --------------------------------------------------------------------------------------------------------------------------------    60
cox1  --------------------------------------------------------------------------------------------------------------------------------     0

R     ATCTGACGTGTCACACCTCAGGGCTTAAACTATGAACCCCGCAGCCCGCCCACAATGCTGCCCTGAAAGCCCTGACAGCACATGCTCACCCCCAGGGTTGGTGGCACTTTCTGGCCACCCCCCATAAG   767
trnC  --------------------------------------------------------------------------------------------------------------------------------    60
cox1  --------------------------------------------------------------------------------------------------------------------------------     0

R     CAGGTCTCCTTTCGCCGCCGGGCGTCGACGGAAAGCCCCGGCAGGCTGGAAACCTGCTTCTTCAGATTTGCAATCTGACGTGTCACACCTCAGGGCTTAAACTATGAACCCCGTAGCCCGCCCACAAT   895
trnC  --------------------------------------------------------------------------------------------------------------------------------    60
cox1  --------------------------------------------------------------------------------------------------------------------------------     0

R     GCTGCCCTGAAAGCCCTGACAGCACATGCTCACCCCCGGGGTTGGTGGCACTTTCTGGCCGCCCCCATAAGCAGGTCTCCTTTCGCCGCCGGGCGTCGACGGAAAGCCCCGGCAGGCTGGAAACCTGC  1023
trnC  --------------------------------------------------------------------------------------------------------------------------------    60
cox1  --------------------------------------------------------------------------------------------------------------------------------     0

R     TTCTTCAGATTTGCAATCGCGCGGTCGGAGCTGCCGCAAGCTACAGTCCCTGAATTCCAACCTCCCTCTGGGTCACCTTGACCTGCTTATAGGCTACACATCCCAACTCGGGTTTAGCCCTCACCCAC  1151
trnC  --------------------------------------------------------------------------------------------------------------------------------    60
cox1  --------------------------------------------------------------------------------------------------------------------------------     0
```

```
                         cox1
R     CTGTGGCAATTACACGCTGATTCTTCTCCACAAACCACAAAGACATTGGCACCCTTTACTT-  1212
trnC  --------------------------------------------------------------    60
cox1  --GTGGCAATCACACGCTGATTTTTCTCAACCAATCACAAAGACATTGGCACCCTGTATTTA    60
                         cox1
```

1.102 NC_007978-NC_014174

Avg ovsize: -405

LCA: Sauropsida-subclass

1.102.1 trnP-nad6 -21

```
                         trnP                                                      nad6
R     A-GGGCTTAAACCTCTATCTCCAGCTCCCAAAGCTGGTATTTTACACTAAACTATTCTCTGATCTCCTTCCCCCCTTAAACCGCT-CGAATAGCCCCACAAGA-CAACCCACGCACCAACTCCAACAC  125
trnP  AAGAGGTTAA-CAACCATCTCTAACTCCCAAAGCTAGAATTTTATCTTAAACTAT-CTCTTA------------------------------------------------------------------   60
nad6  -----------------------------------------------------------------------------------CTCCGAACCGACCCAAAACATCAACC-ACACACAAGATCTAACAC   44
                         trnP                                                        nad6
```

```
       nad6
R     AACAAAC-A----  133
trnP  -------------   60
nad6  TACAAACAATGTT   57
       nad6
```

1.102.2 nad5-cob -24

```
                                 nad5                                                                                cob
R     -ATC-AA-AACCTACTTAGGTTCATTCGCCCTATCCATCCTCAT-C-ACCCTATTAGTTCATAGAACCACAACTCAATGGCCCCAAACCTCCGAAAATCTCATCCACTCCTAAAA-CTCATTAACAAC   122
nad5  TACCTATCAACATTTGTAATTACATCAATACTAGCCATAATTATTCAACTATATTACT----------------------------------------------------------------------   58
cob   ----------------------------------------------------------------------------------CCAT-CCTACGAAAATTAAACCCAATCCTAAAAACT-ATTAACCAC   44
                              nad5                                                                                  cob
```

```
        cob
R     TCCTTAAT----   130
nad5  ------------   58
cob   TCCCTAATTGAC   56
         cob
```

1.102.3 trnE-trnF -1170

```
                                 trnE
R     G-GCTTTTCTCCAAGA-CTCGCGGCCTGAAAAGCCGCTGTTGTCAATTTCAACTACGAAAACACATTTCCCTTCTTTCCCCCCCCCCCCTGCCCCCCCGCATAGAGCGGGCGCGTAAATGTTCCTATG   126
trnE  TATCTAGACAACTAGAACCCCCAATACGAAAAATTGATGTTGT-TAT-TCAACTATAAAAAC------------------------------------------------------------------   60
trnF  --------------------------------------------------------------------------------------------------------------------------------   0
                                 trnE
```

```
R     TCCTTGTCTGCATTACCTTATATTCCCCATTAGACATACAATCCATGTTCAATAGCCATTAATATCCAAACAGGCATACCCCTCCAAAATCCCATTTCTCCTTCCAGAGTACCTCCCGCCCAATGGGA   254
trnE  --------------------------------------------------------------------------------------------------------------------------------   60
trnF  --------------------------------------------------------------------------------------------------------------------------------   0
```

```
R     GCAGAAATCCATTACAATATACGTACTAATACCATCTAGTCTCTAGTTTTTACATAGCTCCTTTAAAGATACGACCGTGCTTGACTGCCCCTTTGAATGAGTTTGGGACAAGGCCCTCCAAATTTTCT   382
trnE  --------------------------------------------------------------------------------------------------------------------------------   60
trnF  --------------------------------------------------------------------------------------------------------------------------------   0

R     CGAATACACAAAGCTTCGTGCCAGGTTATTTATTAATCGAGCTCCTCACGTGAAATCAGCAACCCGGTGTATGGAAGATCCTACGTTACTAGCTTCAGGACCATTCTTTCCCCCTACACCCCTAGCCC   510
trnE  --------------------------------------------------------------------------------------------------------------------------------   60
trnF  --------------------------------------------------------------------------------------------------------------------------------   0

R     ATCTTGCTCTTTTGCGCCTCTGGTTCCTATGTCAGGGCCATAACTTGGTTAGTCCTCTCAACTTGTACTTCACCGATACATCTGGTCGGCTATATATCACCATCTCACCCGTGATCGCGACATCCGAC   638
trnE  --------------------------------------------------------------------------------------------------------------------------------   60
trnF  --------------------------------------------------------------------------------------------------------------------------------   0

R     CGTCTTGGCACTTTTGGTTCCTTTTTTTTTTTGGGCGTCTTCAGGCAGCCCCTCCAGTGCACCGAGGTACACACAATCTAAGACCTGGGCCCTCCCTGGTATTCGAACGGAGTTTGGCCCTCAGGAAT   766
trnE  --------------------------------------------------------------------------------------------------------------------------------   60
trnF  --------------------------------------------------------------------------------------------------------------------------------   0

R     ACCTGAGTGTCATAGTTAACGGGTTGGGGGAATCATCTCCGCGCTGATGCACTTTGTTTTACACCTGGTTATGGCATCTCCGCAAGTTACTAACTATGCTGCTATTTAGTGAATGCTTGTTGGGCATA   894
trnE  --------------------------------------------------------------------------------------------------------------------------------   60
trnF  --------------------------------------------------------------------------------------------------------------------------------   0

R     ATTTATCATTTTTCATTTCCTCTAACTTTCTAAACAACACTAGAAGATTTCATTTGAAAAATGAACCGTATTTTCATCACGTATTTTATCATCACCTTCATCACTTACAAACGGCAGTGAAGTTTCAT   1022
trnE  --------------------------------------------------------------------------------------------------------------------------------   60
trnF  --------------------------------------------------------------------------------------------------------------------------------   0

R     TAAAAATAAAACCATTCACGATTTGCGTTCGTATATTCGAACAACATAACACAAACTATAAACGAAACCCCCCTAAAAACAACAAACAACAAACAACAAACAACAAACAACAAACAACAAACAACAAA   1150
trnE  --------------------------------------------------------------------------------------------------------------------------------   60
trnF  --------------------------------------------------------------------------------------------------------------------------------   0
```

```
                                                                                                                trnF
R     CAACAAACAACAAACAACAAACAACAAACAACAAACAACAAACAACAAACAACAAACAACAAACAACAAACGCTCTGTCTTTGTAGCTTACTACCAAAGCATGGCACTGAAGATGCCAAGACGGCTGC   1278
trnE  --------------------------------------------------------------------------------------------------------------------------------     60
trnF  --------------------------------------------------------------------------------TTGTAGCTTAACATTAA-GCACAGTATTGAAAATACTGAAATGAGTGT     47
                                                                                                                trnF
```

```
       trnF
R     CAT-TCTC-   1285
trnE  ---------     60
trnF  AAACACTCC     56
       trnF
```

1.103 NC_009585-NC_009584

Avg ovsize: -421

LCA: Ilisha-genus

1.103.1 trnP-trnT 0

1.103.2 cob-trnP -48

```
                              cob                                                  trnP
R     TATTTCTAATTCTCACCCCCCTAGCAGGATGACTAGAAAACAAAGCCCTAGAATGAAACTGCCCCAGTAGCTTAACTCAAAGCGCCGGTCTTGTAATCCGGAGATCGG   108
cob   TATTTTTAATCCTCACCCCCCTAGCAGGCTGATTAGAAAACAAAGCCTTAGAATGAAACT------------------------------------------------    60
trnP  ------------------------------------------------------------------------------------------------------------     0
                              cob
```

1.103.3 trnT-trnF -1216

```
                                    trnT
R     -TTAACTCAAAGCGCCGGTCTTGTAATCCGGAGATCGGAGGTTAAAATCCCCCCTGAGGCCAGAGAAAAAAGATTTTAACTTCCACCTCTAACTCCCAAAGCTAGAATTCTAAACTAAACTATTCTCT   127
trnT  CTTAACCCAAAGCGCCGGTCTTGTAATCCGGAGATCGGAGGTTAAAATCCCCCCTAAGGC--------------------------------------------------------------------    60
trnF  --------------------------------------------------------------------------------------------------------------------------------     0
                                    trnT

R     GATGTACTTTATATAATAATATATGGAATAATCCACGTTTACATTACAAGTTTGTTGCAAAGTAACAGCATGTTTCAATTACATAAATGGAAATGGACTAAACTTGTAAAATGTAATTATTTATCCTC   255
trnT  --------------------------------------------------------------------------------------------------------------------------------    60
trnF  --------------------------------------------------------------------------------------------------------------------------------     0

R     AATAAATGGGTATGTCGCATATATTAATGTACAAAAGCATTTACATTCACGTTAAACATTTATGTGCTCCTCCATAGTTCATGTGTTATTTTGCATTATTGGTACGGATACATATATGCATTATTATA   383
trnT  --------------------------------------------------------------------------------------------------------------------------------    60
trnF  --------------------------------------------------------------------------------------------------------------------------------     0

R     CATATATATATGGTATAGTACATATATATGCATTATTATACATATATATATGGTATAGTACATATATATGCATTATTATACATATATATATGGTATAGTACATACTATGTATTATCACCATTCATTTA   511
trnT  --------------------------------------------------------------------------------------------------------------------------------    60
trnF  --------------------------------------------------------------------------------------------------------------------------------     0

R     TTTGAACCAAACAAGTAATAGATATTACTAAAACATACATAACCCATTATTATAAGATTCAAAGTATAATGATATAAACATGATAAGTTGAATATTCCCCATTTTATTCACGTAAACATTTTCCATGC   639
trnT  --------------------------------------------------------------------------------------------------------------------------------    60
trnF  --------------------------------------------------------------------------------------------------------------------------------     0

R     GTTACTCAATATTACTTGATTGTTACAATATTTAATGTAGTAAGAAACCACCAACCAGTTTATATAAAGGCATATCATGAATGATAAGATCAGGGACAATAATCGTGGGGGTTTCACAAAATGAATTA   767
trnT  --------------------------------------------------------------------------------------------------------------------------------    60
trnF  --------------------------------------------------------------------------------------------------------------------------------     0

R     TTTCTGGCATTTGGTTCCTATTTCAGGGTCATATAACTCTTAATCCTCCTATTAATGAATTATATCTGGCATAAGTTAATGGTGGTGTACTAATGGTTCTTTACCCCACATGCCGAGCGTTCATTTAT   895
trnT  --------------------------------------------------------------------------------------------------------------------------------    60
trnF  --------------------------------------------------------------------------------------------------------------------------------     0
```

```
R     ATGCATTTGGTATTTTTTTTTCCGGGTCACTTTCACTTGGCATTTGGCGACTCCTTCCTAATGTTAACTGACAGGGTTGAACATTTCCTTGCTTACAATTGCTAACTGTTTAATACTTCATTAGCATT  1023
trnT  --------------------------------------------------------------------------------------------------------------------------------    60
trnF  --------------------------------------------------------------------------------------------------------------------------------     0

R     GATAGGAGAACCACATAAGTGATATCAGGTGCATAAAGTATACATATCTACTCCACACTTTCCTGTATATAGTGTCCCCCCTCTTCTTGTCTGAAAGTTTTTCGCGCGACAAACCCCCTTTCCCCCTT  1151
trnT  --------------------------------------------------------------------------------------------------------------------------------    60
trnF  --------------------------------------------------------------------------------------------------------------------------------     0

                                                                                                                                 trnF
R     ACGCCGAGAGAATCCTATTTATTCCTGTCAAACCCCAAAAACAGGGAAGATCCGACCTGGCGTCTCAACAAGTTTCAGTGTGTGTTGACTATACAGTGTGCCACTAGTGTGACACACTATTAAACTCA  1279
trnT  --------------------------------------------------------------------------------------------------------------------------------    60
trnF  ----------------------------------------------------------------------------------------------------------------------------CTCA     4
                                                                                                                                 trnF

                                  trnF
R     CGTAGCTTAAACTAAAGCATGACACTGAAGATGTTAAGACGGGCCCTAGAAAGC-  1333
trnT  -------------------------------------------------------    60
trnF  TGTAGCTTAAACCAAAGCATGACACTGAAGATGTTAAGACGGGCCCTAGAAAGCC    59
                                  trnF
```

1.104 NC_006335-NC_006343

Avg ovsize: -450

LCA: Plethodon-genus

1.104.1 nad5-trnT -77

```
                                     nad5                                                                      trnT
R     TAGTAACACTTACCTCTACCCTTTTAATGGCCTCCCCAGTCCTAATTAATTATATCAACTTACCCAAATCTATAATTGATAGAAATTTACACCAAATCAATACTACCGCAGTTTAAGAAAACATCAGC   128
nad5  -A-TATTTTTAATTTCTGCCCTCTT-------------------------------------------------------------------------------------------------------   23
trnT  ------------------------------------------------------------------------------------------------------CTATTGTAGTTTAATTAAACATTGGT   26
             nad5                                                                                                        trnT
```

```
             trnT
R     CTTGTAAGCTGAAGAATGGGGATTTAAAATC-T-   160
nad5  ----------------------------------   23
trnT  CTTGTAAACC-AAAATTGGGGGCTAATTAGCCCC   59
             trnT
```

1.104.2 cob-trnP -92

```
                             cob
R     TTATTTATATTC-TTC--CTCCCACTAATAGGAGTAGCCGAAAACAAACTTATAAAATGATAACAATTTAAGTCGGAAGTGCTCCTAATAATCCAGGCGCCGCCACAAGAAACCCTAACTGAGCCCGG   125
cob   TT-TTACTTTTCCTTCTACTCTCATTAAGTGGATTAATAGAAAATAAACTTATAAAATGAT-------------------------------------------------------------------   60
trnP  --------------------------------------------------------------------------------------------------------------------------------   0
                             cob
```

```
                                                                  trnP
R     GCTCCGCCAAATATAATCTATTAATATATAAAAATAATTTTTT-AAGCAGGAAGGATTTACACCTTCACCATTGACACCCAAGGCCAAAATTCTA-AATTTA   224
cob   ------------------------------------------------------------------------------------------------------   60
trnP  -------------------------ATTTTAAGTTAAAAATTCAAGCAAAAAAGATTTTCACTTTTGTCTCTGACACCCAAGGCCAAAATTCTAGAAACTA   76
                                                               trnP
```

1.104.3 trnT-nad6 -1182

trnT (R: positions 1–53)

```
R     GTTTAAGAAAACATCAGCCTTGTAAGCTGAAGAATGGGGATTTAAAATCTCCCGATAGTCCGGGCTCCGCCTAAGGACGTAGACTTAAACCCAGGCAGAGCCTGGGAAAGAATCTAATTTAAATATAT   128
trnT  GTTTAATTAAACATTGGTCTTGTAAACCAAA-ATTGGGGCTAATTAGCCCCC---------------------------------------------------------------------------   52
nad6  --------------------------------------------------------------------------------------------------------------------------------   0
```

trnT (trnT: positions 1–53)

```
R     ATTCCACATGCGAGTAAAAAGGGGGGGGGTCCTGGCTCTATCCAGGCCCTGCCTTGAGGCATTATTACCAATCCAGGCTCTACCACCATATAACCCAACCCCACATACCCATATAAACCAAAAGTCAC   256
trnT  --------------------------------------------------------------------------------------------------------------------------------   52
nad6  --------------------------------------------------------------------------------------------------------------------------------   0

R     CTAAAAACAACTACCGCTTATGTCCTTTCTTCACTATAGTAACGCGGTGACATATTATGTATAATAGTGCATTCATCTAATTTCCATACGAATCTGTTTTAGTACCCTCCTGGTTTTTAATTTCACAC   384
trnT  --------------------------------------------------------------------------------------------------------------------------------   52
nad6  --------------------------------------------------------------------------------------------------------------------------------   0

R     ACGGTAGAGAAATAAACAACCCGCCCCTCCAGACACGATGTCCAGATCTAAGGACCTATATTGTAGGTCCGCAGCTCGCTATCTTCACGAGACCACTGGTTATGAATCTATGTGCACATAGGCAAGAG   512
trnT  --------------------------------------------------------------------------------------------------------------------------------   52
nad6  --------------------------------------------------------------------------------------------------------------------------------   0

R     TAGGCGGTAACAGTACGACCCCCATATAGTTGAAGGTGATGCTCTCAAGAATCGGCGTACCCCGCACCCACATAACTGGTCTAGATGCATTCATTATTTTTTTTTCTCTTGTGAGGTCAGCCAACACC   640
trnT  --------------------------------------------------------------------------------------------------------------------------------   52
nad6  --------------------------------------------------------------------------------------------------------------------------------   0

R     CAATTTATAACTGGTAAATCTGGCTCTAAAACCTGAACATATAATGCCCGTGTAGTCTTACTATATACGCATGTTTTAATATAAGTCAATGTTGTATGGACATACACCTTCGTATTCCTCTGCCTGAT   768
trnT  --------------------------------------------------------------------------------------------------------------------------------   52
nad6  --------------------------------------------------------------------------------------------------------------------------------   0

R     TCCCAGAATATTACCTCCCAGTTTCCCCCCCCTCTTGCATTTCACAAAATAATTTTATTATACAATATATTTTTCTACTTTAAACCCCCCTCCCCCAAAATATAGGCTAATCGGCGCCTGAAATCTTT   896
trnT  --------------------------------------------------------------------------------------------------------------------------------   52
nad6  --------------------------------------------------------------------------------------------------------------------------------   0
```

```
R     AGTCAAACCCCGAAACCTAAAAACCCATCACACTCACGCACTGAAATAACAAATGATTAATAATAATTTTATTAACTTTTAATATATTTTAATATATTTTAATATATTTTAATATATTTTAATATATT  1024
trnT  --------------------------------------------------------------------------------------------------------------------------------    52
nad6  --------------------------------------------------------------------------------------------------------------------------------     0

R     ATAAGATATATTAGTGTAACTTACTAAAGCACGGCACTGAAAATGCCAATATAAATAATAATAAATTTTATAAACACTAAAAGGTCTGGTCCCAGCCTTAGTATAAATTTTTAATATGAGAACTTATA  1152
trnT  --------------------------------------------------------------------------------------------------------------------------------    52
nad6  --------------------------------------------------------------------------------------------------------------------------------     0

                                                                                                           nad6
R     TAATATATTTAAAATTTCTAACCAACAGCTGCCACCATAAACCAATACAACTTTTTAACCAAATTTACCAAATACATAAACCCCTCACAGCACGTAAAGATCCACGAGATACCCCACGAGTAATTTCT  1280
trnT  --------------------------------------------------------------------------------------------------------------------------------    52
nad6  -----------------------------------------------------------------------------------CTAACAGCACGCAAAGACCCACAAAACACCCCCCAAGTAATCCCT    45
                                                                                                            nad6

        nad6
R     AATACCGTAAAT-A  1293
trnT  --------------    52
nad6  AACACTACAAATAA    59
        nad6
```

1.105 NC_013257-NC_011128

Avg ovsize: -486

LCA: Endopterygota-infraclass

1.105.1 trnQ-trnM 26

```
trnQ                                                                    trnM
R     ATAGAATTAAACTATTTCCTAAAA-TATCAAAAATTTTTGTACATCATATACTAAAATGTA--A-AAAAGATAAGCTAAAAT-AAGCTATTGGGTTCATACCCCACTTATAAAGGTCCGACCCC   119
trnQ  ATAGAATTAAACTATATC-TAAAAGTATCAAAAACTATTGTGCTTATTACACTAAAATATATTATTAATAAAAATATAAATT-AA-TTATT---------------------------------    88
trnM  -----------------------------------------------------------------TAAAAATAAGCTAAATTTAAGCTTTTGGGCTCATACCTCAAATATAAAGTAGATAATTC    59
trnQ
                                                                     trnM
```

1.105.2 trnM-nad2 11

```
trnM                                                                    nad2
R     GCTAAAAT-AAGCTATTGGGTTCATACCCCACTTATAAAGGTCCGACCCCTTTTCTTTTTAATTTTTAA-A-AGCTTTAGATCTCT--AAT-TTTTTTTAATATACTAATTATAGGCACAATTAT   119
trnM  -CTAAATTTAAGCTTTTGGGCTCATACCTCAAATATAAAG-TA-GATAATTCTTTTTTTTAAT--------------------------------------------------------------    60
nad2  ----------------------------------------------------TTATTTTTAATTTACAACTTAAC-TAACCCCCCTAAAATATTTTTTTTTTTTATTT-TAATTTTTAGAA-CA-    69
trnM
                                                                     nad2
```

1.105.3 rrnS-trnI -1495

```
rrnS
R     CACAAAAATATTCATGTAAAATTAAAATTAAATAAATTTATAGCCAAAATCA-AAC-TTTAACTTATAAATATATTTAATATTATATTATTTAAATTTCTATTTAATTTAAATTAAATAAATTTTTAT   126
rrnS  TA-AAATTTA-T-ATGTAAAAT-AAA-TTTTATAA-TAAATTCCCTAAATCATAAAATTTTTCTT---------------------------------------------------------------    59
trnI  --------------------------------------------------------------------------------------------------------------------------------     0
rrnS
```

```
R      TTTATTTTTTTTAATTAAAAATTTATATTATTTAATTTTTTTAAAAATAAATTTAATTAAAATTATTTATATAATAAATATTTTTAAATACACAACTACTTTAATAAAAATAAATACATTATTTTTTC   254
rrnS   --------------------------------------------------------------------------------------------------------------------------------    59
trnI   --------------------------------------------------------------------------------------------------------------------------------     0

R      ATTAAATTTATTAATTAACCACCTTAAATAATTTAATTTATAATAATATCTTCCAAGATTTATATTATTTTAATAATTTAAAAAATATTTTTTTTAATAAAAATTAATCCTATTGAAATAAATATTTT   382
rrnS   --------------------------------------------------------------------------------------------------------------------------------    59
trnI   --------------------------------------------------------------------------------------------------------------------------------     0

R      ATTTTAATTTAATTATTAAAAAATAAATAATTTCAATTTAATAAATTTAATAATTTATATTAAATTATATTATAAATAATATTTTATAAACATAAAATTTAATATAAATATAAAATTTTATTTTTAAT   510
rrnS   --------------------------------------------------------------------------------------------------------------------------------    59
trnI   --------------------------------------------------------------------------------------------------------------------------------     0

R      TAAATTAAATAAATATAAAAAATAAATTTTTAACATAAATTAATTTTATTATTAATTTAATTATTAAAATTAAATTATTTTAATTTAATCAAATTAATAATTAATATTAAATGATAAATAAAATAAAC   638
rrnS   --------------------------------------------------------------------------------------------------------------------------------    59
trnI   --------------------------------------------------------------------------------------------------------------------------------     0

R      AATTTAATTAAAAATATAAAATTTATAATAATTATATAAAATTTTATAAATTTTATATTTTTAATTAAATAATTAATATAAAAATATTTAAATAAATATTTTATTTGTTAAAATAAAAATTATTATAA   766
rrnS   --------------------------------------------------------------------------------------------------------------------------------    59
trnI   --------------------------------------------------------------------------------------------------------------------------------     0

R      AAAAAATTATCTTCCAAGATTTTTTTTATAATTATTTTTAATTTTAAAAATTAAAAAATTTAAATAATTATTTTAATTTTTTAACTAATAATATTTTTTTTTTAATAAATATTTAAATTTTATATCTA   894
rrnS   --------------------------------------------------------------------------------------------------------------------------------    59
trnI   --------------------------------------------------------------------------------------------------------------------------------     0

R      TATATAATAAAATAATTATTAATTTAATTATTTTATATTATATAATAATATAAATATTTTATATTATATAAATAATATAATATATAATATTATATTTATAATTATAATTTAATTAAATATATAATTAA  1022
rrnS   --------------------------------------------------------------------------------------------------------------------------------    59
trnI   --------------------------------------------------------------------------------------------------------------------------------     0

R      TTTATATATCTCTCTTTTTATCTAAATATATAAATATAATTATTAAATCTAATATTATATATATATATATAATTATATTTATTTTATATACATATATGTAAATACTTATTATATATAAATATAATTAT  1150
rrnS   --------------------------------------------------------------------------------------------------------------------------------    59
trnI   --------------------------------------------------------------------------------------------------------------------------------     0
```

```
R     ATATATATATATAATTATATTTAAATAATTATATTAATATATATATATATAATATAATATAGATTTTTTATATATTAATAAATTAATTTATTTTCCATTAATTTAGTAAATATTTAATAAAACCTTTT  1278
rrnS  --------------------------------------------------------------------------------------------------------------------------------    59
trnI  --------------------------------------------------------------------------------------------------------------------------------     0

R     TTAGCTTTTTTTTTATATAATTAAATTTATTAATAAATAATAAATTAAAACCAATACATTTATTTTATATAAATATAATGACAATCACAAACTTTATTTAATATAAAATAAATTTTATAAATAAATTT  1406
rrnS  --------------------------------------------------------------------------------------------------------------------------------    59
trnI  --------------------------------------------------------------------------------------------------------------------------------     0

R     TTAATCATAAAAAATCTATTTATTTTTTATTTATTTTAACTAAATTTAACATTTAATAATTAATTATAACCATAATTTAAATATTTTTATACAAATTAATTAAATTTAAAATAAATAGATTTTTATAG  1534
rrnS  --------------------------------------------------------------------------------------------------------------------------------    59
trnI  --------------------------------------------------------------------------------------------------------------------------------     0

                                                  trnI
R     TTAAAAATTAATATTTTTAATATAATGAAGTGCCTGACTAAAGGATTATTTTGATAGAATAAATCATGTATTTTATATACC-T-  1616
rrnS  ------------------------------------------------------------------------------------    59
trnI  ------------------------ATAAAGTGCCTGATTAAAGGATTATTCTGATAGGATAAATTAAGTAAA-TATTTACCTTT    59
                                                  trnI
```

1.106 NC_006405-NC_005796

Avg ovsize: -491

LCA: Teleostomi-superclass

1.106.1 nad4-trnH 4

nad4 *trnH*

```
R      TTCTTCTTCATACTGCACCTGCACTTCTATTTATTCTTAATCCTGAGCTTATTTTTTGCTG-TGAATATAGTTTAACTAAAATCCTAGGTTGTGGTCCTAGAAATGAGG-GTTAATATC-CT   119
nad4   A-CTTAAT--TCCAGTA--TT-ACTTCTAGT-A--CTGAAGCCCGAACTAATATGAGGCTGATGCATAT-----------------------------------------------------   60
trnH   -----------------------------------------------------------------ATATAGTTTAAAAAAGACGTTAGATTGTGATTCTAAAAAT-AGGAGATAAAACCTCT   56
```

nad4

trnH

1.106.2 trnL1-trnT -2

trnL1 *trnT*

```
R      AATAGCCCTTCCCCTGGTCTTAGGCTCCAGTATCTCTTGGTGCAAATCCAAGTAAAAGCTAGCCCAGATAGCTTATATTAAAGCATCGGTCTTGTAAACCGAAGATTGTAGATTAAA--CCC   120
trnL1  AATAGCTCATCCGTTGGTCTTAGGAACCAAAAACTCTTGGTGCAACTCCAAGTAGAGGCT--------------------------------------------------------------   60
trnT   --------------------------------------------------------------CCTAG-TAGCTTATAA-AAAGCACCGGTCTTGTAATCCGAAGATTGAAGGTTAAAGTCCT   58
```

trnL1

trnT

1.106.3 cob-trnL1 -1475

cob

```
R      ATCTTTGCTCC-ATTCT-TCCCCATCATTGGACTCTTAGAAAACAAGTTACTTCAGCTTTAACATAAGCAGTTCCCGGATTTTTTCCGCTTCTACCGCCTCCCCGATAACCCCCATTCCAGGATTTTC   126
cob    -TATT-CCTAGTATTAAATCCAC-TAGTAGGATGACTAGAGAATAAAGCACTTAA-------------------------------------------------------------------------   52
trnL1  --------------------------------------------------------------------------------------------------------------------------------   0
```

cob

```
R       CCTAGCAAATTAGCTTTTTTTGTTGGCAGTTACCGACCCCTATGTACCTAAAACATACATCTATATACCACATATTAAATAGACCATTATATGTATAATCCCCAATAATAGATATACCCCATATATCA  254
cob     --------------------------------------------------------------------------------------------------------------------------------  52
trnL1   --------------------------------------------------------------------------------------------------------------------------------  0

R       GTAGCATATTATGTTTAATCAACATTAATCTATTAACCCCATGCTTACCTTTTCCCTAATACTGGTTAATTTTACACCCTATTTATTGGACATTCCCCCATAATTACAAAGAATCATGAATGGCACCG  382
cob     --------------------------------------------------------------------------------------------------------------------------------  52
trnL1   --------------------------------------------------------------------------------------------------------------------------------  0

R       CCCATCACGTGAAATGGCAGCCGCCCATACTTTCAATACCAGTTCGGATCCTTAAATTCTTTAGTAACGTCATATCCTCACCATTAATTATCCAATCGCTTCATCCACCCACCATCACACCCATAATT  510
cob     --------------------------------------------------------------------------------------------------------------------------------  52
trnL1   --------------------------------------------------------------------------------------------------------------------------------  0

R       AATGTAACTAAATTCCCTTATAATGAAAACGTACAACCTATACATCCACATTACTCCATTCCCCCAACAATGATTGTATAATCCCATAAAAATTTGAATTGTACCTTCACTTGAAGTAAGAACACGAC  638
cob     --------------------------------------------------------------------------------------------------------------------------------  52
trnL1   --------------------------------------------------------------------------------------------------------------------------------  0

R       TATTATTTATAATAAACTTTCATAATACCCAATAATGGTACATTAAGCATGAACATAAAATGTATAATCCACATAACAGTAACAACAACATAAATATTGTACTACCCTTCCCTGCATACACAACTAGC  766
cob     --------------------------------------------------------------------------------------------------------------------------------  52
trnL1   --------------------------------------------------------------------------------------------------------------------------------  0

R       AGCAATTGCTGCTGCTACATGGATACCTTAATGAGACCTAACTGAAATTCCAGCCCGGGGATCTTATTTTCCCCCGGTCATATATTGTAGAGTAACCTCAATTGACCTTCAAAAAACATCCGGTAGCC  894
cob     --------------------------------------------------------------------------------------------------------------------------------  52
trnL1   --------------------------------------------------------------------------------------------------------------------------------  0

R       CCGAATCTATGGACCTGTTGACAAGAGACGTCCTTTCTCGTAATTTAAAAAACATCCCTCCTTATGCGTGAAGTACCTGCAGAGTCCTTAACCTTACCTGGTCTGAATTGGATAGGCATAAATAATAT  1022
cob     --------------------------------------------------------------------------------------------------------------------------------  52
trnL1   --------------------------------------------------------------------------------------------------------------------------------  0

R       AGGGAACTTTCACCTGGCATCTCTCAGTGGGGTAATAACATTAAACTTGGGTTGGACATAACATGCTCGCGTGGGATTTCACCATCCCCTCTATTGTTCCCCCTTAAGTCAATGCTAGATGGACATAG  1150
cob     --------------------------------------------------------------------------------------------------------------------------------  52
trnL1   --------------------------------------------------------------------------------------------------------------------------------  0
```

```
R      CATTTATATAATTTCCCCCCCTCCTACCAAAATTCGGCTAAAATTTTAACGTAWWTTCTCGACACCCCCCCTACCCCCCCACACAAGGAATTTTAGTAGCTTTAACTAACCACCCCCCCGGAGTTAGT  1278
cob    --------------------------------------------------------------------------------------------------------------------------------  52
trnL1  --------------------------------------------------------------------------------------------------------------------------------  0

R      GTCTAAAAAAAATTTTACCGTTATGTGTTTTCATAATAAAATCAAATTTTTAGACCCATCACATGTCAAATTGGGTCCTAGATGTATATATATCATATTTTAATCTTCAACGATGTCTATAGCTTTAGT  1406
cob    --------------------------------------------------------------------------------------------------------------------------------  52
trnL1  --------------------------------------------------------------------------------------------------------------------------------  0

                                                                                             trnL1
R      CAACTTTTTTTAACTAAGGCCCTAGACTCCCCCCACAATTTTTTAACCTCTACCTACAAGCCATACCGCTTTTAAAGGAAAATAGCCCTTCCCCTGGTCTTAGGCTCCAGTATCTCTTGGTGCAAA  1532
cob    ------------------------------------------------------------------------------------------------------------------------------  52
trnL1  --------------------------------------------------------------------------------------------------------------------------CAAA  4
                                                                                                                             trnL1
```

1.107 NC_006405-NC_020000

Avg ovsize: -500

LCA: Anura-order

1.107.1 trnL1-trnT -5

```
                           trnL1                                                   trnT
R      AATAGCCCTTCCCCTGGTCTTAGGCTCCAGTATCTCTTGGTGCAAATCCAAGTAAAAGCTAGCCCAGATAGCTTATATTAAAGCATCGGTCTTGTAAACCGAAGATTGTAGATTAA-ACCC-  120
trnL1  AATAGTCTATCCATTGGTCTTAGGCGCCAAAATTTCTTGGTGCAACTCCAAGTGAAAGCT--------------------------------------------------------------  60
trnT   -----------------------------------------------------------------AG-TAGCTTA-ATTAAAGCATCGGCCTTGTAAGCCGAAGAATGGAGACTAACACTCT  55
                           trnL1
                                                                                       trnT
```

1.107.2 trnS1-nad5 -77

trnS1 *nad5*

```
R      TCTGGGAAAACGAGAACTGCTAATTCCTCGCGTCCATGGTTCAATTCCATGGCTTGCTCGAAACTTTTATCATGACACCATCAAGTTAAAAGTCATGGACCTCCTCCTGGTCACCTCATCATGCCTTA   128
trnS1  ACTGGGTAAAACGAGAACTGCTAATTACTCGCCCCCGCGGTTCAATTCCGCGGCTTTCCCG--------------------------------------------------------------------   60
nad5   --------------------------------------------------------------------------------------------------------------------------------   0
```

trnS1

nad5

```
R      TTATAACCATCATTACAATTTCACT   153
trnS1  -------------------------   60
nad5   ---------TCCTTACT-TTACCCA   15
```

nad5

1.107.3 cob-trnL1 -1418

cob

```
R      ATCT-TTGCTCCATTCTTCC-CCATCATTGGACTCT-TAGAAAACAAGTTACTTCAGCTTTAACATAAGCAGTTCCCGGATTTTTTCCGCTTCTACCGCCTCCCCGATAACCCCCATTCCAGGATTTT   125
cob    -TCTATTCCTT-ATCCTTATTCCGGCTGGGGTTGAATAGAAAACAAACTA-TTAA-CT---------------------------------------------------------------------   55
trnL1  --------------------------------------------------------------------------------------------------------------------------------   0
```

cob

```
R      CCCTAGCAAATTAGCTTTTTTTGTTGGCAGTTACCGACCCCTATGTACCTAAAACATACATCTATATACCACATATTAAATAGACCATTATATGTATAATCCCCAATAATAGATATACCCCATATATC   253
cob    --------------------------------------------------------------------------------------------------------------------------------   55
trnL1  --------------------------------------------------------------------------------------------------------------------------------   0
```

```
R      AGTAGCATATTATGTTTAATCAACATTAATCTATTAACCCCATGCTTACCTTTTCCCTAATACTGGTTAATTTTACACCCTATTTATTGGACATTCCCCCATAATTACAAAGAATCATGAATGGCACC   381
cob    --------------------------------------------------------------------------------------------------------------------------------   55
trnL1  --------------------------------------------------------------------------------------------------------------------------------   0
```

```
R      GCCCATCACGTGAAATGGCAGCCGCCCATACTTTCAATACCAGTTCGGATCCTTAAATTCTTTAGTAACGTCATATCCTCACCATTAATTATCCAATCGCTTCATCCACCCACCATCACACCCATAAT   509
cob    --------------------------------------------------------------------------------------------------------------------------------   55
trnL1  --------------------------------------------------------------------------------------------------------------------------------   0

R      TAATGTAACTAAATTCCCTTATAATGAAAACGTACAACCTATACATCCACATTACTCCATTCCCCCAACAATGATTGTATAATCCCATAAAAATTTGAATTGTACCTTCACTTGAAGTAAGAACACGA   637
cob    --------------------------------------------------------------------------------------------------------------------------------   55
trnL1  --------------------------------------------------------------------------------------------------------------------------------   0

R      CTATTATTTATAATAAACTTTCATAATACCCAATAATGGTACATTAAGCATGAACATAAAATGTATAATCCACATAACAGTAACAACAACATAAATATTGTACTACCCTTCCCTGCATACACAACTAG   765
cob    --------------------------------------------------------------------------------------------------------------------------------   55
trnL1  --------------------------------------------------------------------------------------------------------------------------------   0

R      CAGCAATTGCTGCTGCTACATGGATACCTTAATGAGACCTAACTGAAATTCCAGCCCGGGGATCTTATTTTCCCCCGGTCATATATTGTAGAGTAACCTCAATTGACCTTCAAAAAACATCCGGTAGC   893
cob    --------------------------------------------------------------------------------------------------------------------------------   55
trnL1  --------------------------------------------------------------------------------------------------------------------------------   0

R      CCCGAATCTATGGACCTGTTGACAAGAGACGTCCTTTCTCGTAATTTAAAAAACATCCCTCCTTATGCGTGAAGTACCTGCAGAGTCCTTAACCTTACCTGGTCTGAATTGGATAGGCATAAATAATA   1021
cob    --------------------------------------------------------------------------------------------------------------------------------   55
trnL1  --------------------------------------------------------------------------------------------------------------------------------   0

R      TAGGGAACTTTCACCTGGCATCTCTCAGTGGGGTAATAACATTAAACTTGGGTTGGACATAACATGCTCGCGTGGGATTTCACCATCCCCTCTATTGTTCCCCCTTAAGTCAATGCTAGATGGACATA   1149
cob    --------------------------------------------------------------------------------------------------------------------------------   55
trnL1  --------------------------------------------------------------------------------------------------------------------------------   0

R      GCATTTATATAATTTCCCCCCCTCCTACCAAAATTCGGCTAAAATTTTAACGTAWWTTCTCGACACCCCCCCTACCCCCCCACACAAGGAATTTTAGTAGCTTTAACTAACCACCCCCCCGGAGTTAG   1277
cob    --------------------------------------------------------------------------------------------------------------------------------   55
trnL1  --------------------------------------------------------------------------------------------------------------------------------   0

R      TGTCTAAAAAAATTTTACCGTTATGTGTTTTCATAATAAAATCAAATTTTTAGACCCATCACATGTCAAATTGGGTCCTAGATGTATATATATCATATTTTAATCTTCAACGATGTCTATAGCTTTAG   1405
cob    --------------------------------------------------------------------------------------------------------------------------------   55
trnL1  --------------------------------------------------------------------------------------------------------------------------------   0
```

```
                                                                               trnL1
R      TCAACTTTTTTTAACTAAGGCCCTAGACTCCCCCCACAATTTTTTAACCTCTACCTACAAGCCATACCGCTTTTAAAGGAAAATAGCCCTTCCCCTGGTCTTAGGCTCCAGTATCTCTTGGTGCAA-A 1532
cob    -------------------------------------------------------------------------------------------------------------------------------- 55
trnL1  ---------------------------------------------------------------------CTTTTAAAGGAAAATAGTCTATCCATTGGTCTTAGGCGCCAAAATTTCTTGGTGCAACT 59
                                                                               trnL1
```

1.108 NC_006405-NC_007440

Avg ovsize: -501

LCA: Neobatrachia-suborder

1.108.1 nad6-trnE 31

```
       nad6                                                        trnE
R      GGGAGAAGGATTTGAAGCAACAGCCACAAATCCAACCAATAGTCATAATTCAAATACCATATATTCCTGCCAGG-ACTCT-AACCTGGACCAACAGTATGAAAAACTGCCGCTGTAATTCAA- 120
nad6   CG-A--CGGATTTGAGGCCACAGCCAATATTCCCAATAATAAACACAACTCCTTTATCA-CTATTTCTGCTAGGTACT-T-AACCTAGTCT-------------------------------- 85
trnE   ------------------------------------------------------------ATATTTCTGCCAGG-ACT-TCAACCTAAACTTGTAATCTGAAAAACTACTGTTGTTGTTCAAC 61
       nad6
                                                                   trnE
```

1.108.2 trnE-cob -61

```
       trnE                                                        cob
R      CAGGACTCTAACCTGGACCAACAGTATGAAAAACTGCCGCTGTAATTCAACTACAAGAACTCATGGCCCCCGCAATTCGCAAAACCCATCCACTATTAAAAATTATTAACAGTGCCTTTAT 121
trnE   CAGGACTTCAACCTAAACTTGTAATCTGAAAAACTACTGTTGTTGTTCAACTACAGAAAC------------------------------------------------------------- 60
cob    ------------------------------------------------------------------------------------------------------------------------- 0
       trnE
```

1.108.3 cob-trnL1 -1473

```
                                       cob
R      ATCTTTGCTCCATTCTTCCCCATCATTGGACTCTTAGAAAACAAGTTACTTCAGCTTTAACATAAGCAGTTCCCGGATTTTTTCCGCTTCTACCGCCTCCCCGATAACCCCCATTCCAGGATTTTCCC   128
cob    --------------------------------------------------------------------------------------------------------------------------------     0
trnL1  --------------------------------------------------------------------------------------------------------------------------------     0

R      TAGCAAATTAGCTTTTTTTGTTGGCAGTTACCGACCCCTATGTACCTAAAACATACATCTATATACCACATATTAAATAGACCATTATATGTATAATCCCCAATAATAGATATACCCCATATATCAGT   256
cob    --------------------------------------------------------------------------------------------------------------------------------     0
trnL1  --------------------------------------------------------------------------------------------------------------------------------     0

R      AGCATATTATGTTTAATCAACATTAATCTATTAACCCCATGCTTACCTTTTCCCTAATACTGGTTAATTTTACACCCTATTTATTGGACATTCCCCCATAATTACAAAGAATCATGAATGGCACCGCC   384
cob    --------------------------------------------------------------------------------------------------------------------------------     0
trnL1  --------------------------------------------------------------------------------------------------------------------------------     0

R      CATCACGTGAAATGGCAGCCGCCCATACTTTCAATACCAGTTCGGATCCTTAAATTCTTTAGTAACGTCATATCCTCACCATTAATTATCCAATCGCTTCATCCACCCACCATCACACCCATAATTAA   512
cob    --------------------------------------------------------------------------------------------------------------------------------     0
trnL1  --------------------------------------------------------------------------------------------------------------------------------     0

R      TGTAACTAAATTCCCTTATAATGAAAACGTACAACCTATACATCCACATTACTCCATTCCCCCAACAATGATTGTATAATCCCATAAAAATTTGAATTGTACCTTCACTTGAAGTAAGAACACGACTA   640
cob    --------------------------------------------------------------------------------------------------------------------------------     0
trnL1  --------------------------------------------------------------------------------------------------------------------------------     0

R      TTATTTATAATAAACTTTCATAATACCCAATAATGGTACATTAAGCATGAACATAAAATGTATAATCCACATAACAGTAACAACAACATAAATATTGTACTACCCTTCCCTGCATACACAACTAGCAG   768
cob    --------------------------------------------------------------------------------------------------------------------------------     0
trnL1  --------------------------------------------------------------------------------------------------------------------------------     0

R      CAATTGCTGCTGCTACATGGATACCTTAATGAGACCTAACTGAAATTCCAGCCCGGGGATCTTATTTTCCCCCGGTCATATATTGTAGAGTAACCTCAATTGACCTTCAAAAAACATCCGGTAGCCCC   896
cob    --------------------------------------------------------------------------------------------------------------------------------     0
trnL1  --------------------------------------------------------------------------------------------------------------------------------     0
```

```
R      GAATCTATGGACCTGTTGACAAGAGACGTCCTTTCTCGTAATTTAAAAAACATCCCTCCTTATGCGTGAAGTACCTGCAGAGTCCTTAACCTTACCTGGTCTGAATTGGATAGGCATAAATAATATAG  1024
cob    --------------------------------------------------------------------------------------------------------------------------------     0
trnL1  --------------------------------------------------------------------------------------------------------------------------------     0

R      GGAACTTTCACCTGGCATCTCTCAGTGGGGTAATAACATTAAACTTGGGTTGGACATAACATGCTCGCGTGGGATTTCACCATCCCCTCTATTGTTCCCCCTTAAGTCAATGCTAGATGGACATAGCA  1152
cob    --------------------------------------------------------------------------------------------------------------------------------     0
trnL1  --------------------------------------------------------------------------------------------------------------------------------     0

R      TTTATATAATTTCCCCCCCTCCTACCAAAATTCGGCTAAAATTTTAACGTAWWTTCTCGACACCCCCCCTACCCCCCCACACAAGGAATTTTAGTAGCTTTAACTAACCACCCCCCCGGAGTTAGTGT  1280
cob    --------------------------------------------------------------------------------------------------------------------------------     0
trnL1  --------------------------------------------------------------------------------------------------------------------------------     0

R      CTAAAAAAATTTTACCGTTATGTGTTTTCATAATAAAATCAAATTTTTAGACCCATCACATGTCAAATTGGGTCCTAGATGTATATATATCATATTTTAATCTTCAACGATGTCTATAGCTTTAGTCA  1408
cob    --------------------------------------------------------------------------------------------------------------------------------     0
trnL1  --------------------------------------------------------------------------------------------------------------------------------     0

                                                                                            trnL1
R      ACTTTTTTTAACTAAGGCCCTAGACTCCCCCCACAATTTTTTAACCTCTACCTACAAGCCATACCGCTTTTAAAGGAAAATAGCCCTTCCCCTGGTCTTAGGCTCCAG-TATCTCTTGGTGCAA--A  1532
cob    -------------------------------------------------------------------------------------------------------------------------------     0
trnL1  -----------------------------------------------------------------GCTTTTAATGGAAAAGAGCT-TTCCTCTGGTTTTAGGGGCCAGCTA-ATCTTGGTGCAAGTC    60
                                                                                            trnL1
```

1.109 NC_004592-NC_006335

Avg ovsize: -503

LCA: Teleostomi-superclass

1.109.1 nad5-nad6 -30

```
                                        nad5                                                  nad6
R     ACATACTTAACCCTTTTTTTCCTAACCTTTACTCTTGCCCTTTTCTTTTCTGCTTCTTAAACAGCCCGAACCGTCCCCCGACTCAACCCCCGTGTTAACTCTAATACTACAAA-AA  115
nad5  ---TA-GTAACACTTACCT--CTACCTTT--------------------------------------------------------------------------------------   24
nad6  ------------------------------------------------------------ACAGCACGTAAAGATCCACGAGATACCCCACGAGTAATTTCTAATACCGTAAATAA   56
                     nad5                                                                   nad6
```

1.109.2 trnT-trnF -303

```
                                         trnT
R     -TCAGAGAAGAGCATCGGTTTTGTAATCCGAA-AGTCGAAGGTTAAAATCCATCCTGATGCTAAGAGC-CC-CCTTTTATTTAAATTTTCTGTATTTTACATATTTTCTAATCACTTAGTGACTAAAA  124
trnT  GTTTAAGAAAA-CATCAGCCTTGTAAGCTGAAGAATGGGGATTTAAAAT-C-TCCCGATAGTCCGGGCTCCGCCT-----------------------------------------------------   72
trnF  --------------------------------------------------------------------------------------------------------------------------------    0
                                         trnT

R     TTAACATAAAAAGTATAATAAATCATCAAAACTCCAAACACCTAATAATATAACAAATAAGAATAAATAAAACAAAACCGGCAAGTCTCCAACAATAGCTGTAAATGCTCACCCTATAAAGATTATAT  252
trnT  --------------------------------------------------------------------------------------------------------------------------------   72
trnF  --------------------------------------------------------------------------------------------------------------------------------    0

                                                                                                                                trnF
R     TTACTGCCTCTTAAAGCCTATAGCCTTATTTACCCTTCCCCTTAATATGCCTTAAATCTTCCTATCCAAACAACTATTAAACATTAAATATCCCTCCACTAAACCCCTTCGCTCACGTATTCTACTTA  380
trnT  --------------------------------------------------------------------------------------------------------------------------------   72
trnF  ----------------------------------------------------------------------------------------------------------------------------TACTAA    6
                                                                                                                              trnF
```

```
                                     trnF
R     AGATATAACACTGAAGATGTTAAGACGGCCCCTAA-AAAG-GC   421
trnT  -------------------------------------------    72
trnF  AGC-ACGGCACTGAAAATGCCAAGACAGATAATAATAAATCTC    48
                                     trnF
```

1.109.3 trnP-trnT -1178

```
                                  trnP
R     GAGATTTTAACTCCT-ATCTTTAGCTCCCAAAGCTAAAATTTTAAATTAAACTACC-CTCTGGAGATGTACCATTTAGTATTATGGTCTTTAAGATTTGTATAGTATAACTATGCATTATCAACATAA   126
trnP  AGGATTTACAC-CTTCACCATTGACACCCAAGGCCAAAATTCTAAATTTAACTACCGCT---------------------------------------------------------------------    58
trnT  --------------------------------------------------------------------------------------------------------------------------------     0
                                  trnP

R     ATTTATTTAGACCTATCATACATCAGTATTCCTCAAAGAACCACATAAAGCCAAACACGTGATAATAACCAATTAGGAAGCTCTACCACCGGGAAATTGAATCTTCAATAGATAATCCATCACCCTAC   254
trnP  --------------------------------------------------------------------------------------------------------------------------------    58
trnT  --------------------------------------------------------------------------------------------------------------------------------     0

R     TGCTCTCTCACAAACCCACCATCTATACAGTTTAGTGCCGCTTAATGTAGTAAGAACCGACCAACATTGCCACTCGCGCATACTCTTCATGATGATCACGTGCAATTATTGTGGGGGTCGCTATTAGT   382
trnP  --------------------------------------------------------------------------------------------------------------------------------    58
trnT  --------------------------------------------------------------------------------------------------------------------------------     0

R     GAACTATTACTTGCATCTGGTTCCTATTTCAGGGTCAATCCTTAAGAAACCACCAACTGAAAGCCGAATGCAATGCATCTGGTTAATGGTGTCGACCTTACGACTCGTTACCCACCAAGCCGGGCGTT   510
trnP  --------------------------------------------------------------------------------------------------------------------------------    58
trnT  --------------------------------------------------------------------------------------------------------------------------------     0

R     CTTTTAAATGCATGGGGTTCTCTTTTTTTTCTTTCCTTTCACTTTGCATCCCCAGTGCACACTAAAAAGACTAACAAGGGAGACCTAGATCTTGTTGTCCAGAGGACCAATGTTCCATGTAAAAAGAC   638
trnP  --------------------------------------------------------------------------------------------------------------------------------    58
trnT  --------------------------------------------------------------------------------------------------------------------------------     0
```

```
R     TTTGACAGAAGAAACCCATAATTTGTTTTCTAGTGCATAAGCCTACTCCTGTCAGCCCCCGGTGGTAACCTTCAAAATTTTTCCTAAAGCCCCCCTACCCCCCAAAAATCTACGTAAGGGGCACCACT   766
trnP  --------------------------------------------------------------------------------------------------------------------------------    58
trnT  --------------------------------------------------------------------------------------------------------------------------------     0

R     TTTAGAACTAATTTACCTTAAAAAATATAAAACCCCAACATAAACTTGTTGCCCTCCAAATAAAGGTGCGAATTTGTGCCCTCTTTATAGTAGGCCATGTCTTTTCATTTATTTATGTAAAATTGAAT   894
trnP  --------------------------------------------------------------------------------------------------------------------------------    58
trnT  --------------------------------------------------------------------------------------------------------------------------------     0

R     AGATAAGAAAGGACAAAAAAACTAGAGCCCTATAAAACTCTTTCAACAAATACTCCTCTCCTTTCCTAATTATTAGCTAAACCAGAAAATATCTCTATCTTAATTCATCTTGGCCCTTCACAACAAAC  1022
trnP  --------------------------------------------------------------------------------------------------------------------------------    58
trnT  --------------------------------------------------------------------------------------------------------------------------------     0

R     GGCCTTAATTTAACAATTAAATTAGCCTAAGTCCTTAAAATAAATAATATAACTTTATCATGCGTTCTGTAAAGAGCAAATTTTGTCCTTCTTTATACGCGTCCTAGCGTTTCTAGTTATTTTTATGA  1150
trnP  --------------------------------------------------------------------------------------------------------------------------------    58
trnT  --------------------------------------------------------------------------------------------------------------------------------     0

                                                                                                                      trnT
R     AAGCCCCCGTAAAGAACTCATTATTTACTTTATAGGTCCCTTACAGAAACCTGCATAGAATGTGCCTAAAATATAAGTCTTCCCCCACCAAAATTATGCACGCACCAGAAGCTCAGAGAAGAGCATCG  1278
trnP  --------------------------------------------------------------------------------------------------------------------------------    58
trnT  -------------------------------------------------------------------------------------CACCA-AATCA-ATAC-TACC-GCAGTTTA-AGAA-AACATCA    37
                                                                                                                   trnT

                    trnT
R     GTTTTGTAATCCGAA-AGTCGAAGGTTAAAAT--C  1310
trnP  -----------------------------------    58
trnT  GCCTTGTAAGCTGAAGAATGGGGATTTAAAATCTC    72
                    trnT
```

1.110 NC_008225-NC_006286

Avg ovsize: -520

LCA: Teleostomi-superclass

1.110.1 trnP-trnE -28

```
R     GAGACTTAAAC--TCC-CACCCCCAGCTCCCAAAGCTGAGATTCTTCATTAAACTATCCTCTGCAACCCCAAATATAATTCTTGCCCGGGCTCTAACCAGGACCAATGACTTGAAAAACCACCGTTG-   124
trnP  ---CTTTAAGCGCGCCTCATCTCCCGCCCCCAAAACGGGCATTTTAAACTAAACTATCCTCTG-----------------------------------------------------------------   60
trnE  -------------------------------------------------------------------------------------------TCTA-CC-GGACCTGCGGTCTGAAAAACCGCCGTTGT   35
```

```
R     ATTCAACT-   132
trnP  ---------    60
trnE  GTTCAACTA    44
```

1.110.2 nad5-nad6 -62

```
R     CCATACCTTCTTACCCACCTGACAATACTCCTAATATCCTTAATTCTATTTTCCCTTAGACACCCACTCTAATCGCACGCA-AG-GCCCCGTAAGATCCCCCACGAGTAACCTCCAATACTA-AAGCC   125
nad5  --------------------------------------------------------------------------------------------------------------------------------     0
nad6  --------------------------------------------------------------CCC-CCCTAAATACCCGAATAGCGCCCCGTTCCCCACCTAAAGA-TAACCAT-AATACCACAAAAA    63
```

```
        nad6
R        A   126
nad5     -     0
nad6     G    64
        nad6
```

1.110.3 trnE-trnF -1470

```
                                              trnE
R     GCCCGGG-CTCTAACCAGGACCAATGACTTGAAAAACCACCGTTGA-TTCAACTACAAGAACAAACAACGCATCTGCCAACTATAGTCCTTGAAATATAAAATGTCTAGTCATTATTCTATGTATTAA   126
trnE  GCCGGCAATTCTA-CC-GGACCTGCGGTCTGAAAAACCGCCGTTGTGTTCAACTACAAAAAC------------------------------------------------------------------    60
trnF  --------------------------------------------------------------------------------------------------------------------------------     0
                                              trnE

R     CACCATAAAAATATTTTAACCATACAGGAATTACTATCGTTTTCATTTTTCAAAACGAAGTAGGTATAATCACATTAACATAATTAAACCACCCTTTTTATCTACCACTCCCTATCCTCTTAAATACT   254
trnE  --------------------------------------------------------------------------------------------------------------------------------    60
trnF  --------------------------------------------------------------------------------------------------------------------------------     0

R     CATTAAATCACCCGCTAGTCTCGCTTCTGTGGCCCACGTTCTCTTGTAAGGTCAAGGGCAAGACATTTGACTTCCCCTTTCAGTGAATTATTCCTGGCATTCAGCCTAGCTTCACGGCCATTAAAAGT   382
trnE  --------------------------------------------------------------------------------------------------------------------------------    60
trnF  --------------------------------------------------------------------------------------------------------------------------------     0

R     TTATTGTACATAACTTGCATTTTTGCGCATTTATGAATGGTGGAACACATCAATTGATAATCGCTTCGCTAGCCGAGCGCTCTTTCTAAAGGGTATTTGGTATTTTTTCTTCTTTCCTTTCATTTTAC   510
trnE  --------------------------------------------------------------------------------------------------------------------------------    60
trnF  --------------------------------------------------------------------------------------------------------------------------------     0

R     ATTTCAGAGTGTAAACAAATGATTCTCTTAAGGTTGAACAAATTTCTTGAACTCAGGAAATAATGTATTATGTTTAATAATTATTCTTGCTAAATTGCATACCTGTAGCAAGAGCATAATACTATATT   638
trnE  --------------------------------------------------------------------------------------------------------------------------------    60
trnF  --------------------------------------------------------------------------------------------------------------------------------     0
```

```
R     ACTTATTCAGACTAACCTATCTTATGCCCCCTCCCGTTCGTCTAAACCCCCCTACCCCCCAGTTCCTAAAGTCTTATTCTGCAAACCCCCCGGAAACAGAAACTCCATGAACTGGACAACAAACAATT   766
trnE  --------------------------------------------------------------------------------------------------------------------------------    60
trnF  --------------------------------------------------------------------------------------------------------------------------------     0

R     TAACCCATAACACCCCGCACCCCCAACCTTCCACACATACACACATACATATATACATATATACATGTATACGTGTGTACATATATACATATATACATGTATACGTGTGTACATATATACATATATAC   894
trnE  --------------------------------------------------------------------------------------------------------------------------------    60
trnF  --------------------------------------------------------------------------------------------------------------------------------     0

R     ATATATACATGTATACGTGTGTACATATATACATATATACATGTATACGTGTGTACATATATACATATATACATGTATACGTGTGTACATATATACATATATACATGTATACGTGTGTACATATATAC  1022
trnE  --------------------------------------------------------------------------------------------------------------------------------    60
trnF  --------------------------------------------------------------------------------------------------------------------------------     0

R     ATATATACATATATACATGTATACGTGTGTACATATATACATATATACATGTATACGTGTGTACATATATACATATATACATGTATACGTGTGTACATATATACATATATACATGTATACGTGTGTAC  1150
trnE  --------------------------------------------------------------------------------------------------------------------------------    60
trnF  --------------------------------------------------------------------------------------------------------------------------------     0

R     ATATATACATATATACATGTATACGTGTGTACATATATACATATATACATATATACATATATACATGTATACGTGTGTACATATATACATATATACATGTATACGTGTGTACATATATACATATATAC  1278
trnE  --------------------------------------------------------------------------------------------------------------------------------    60
trnF  --------------------------------------------------------------------------------------------------------------------------------     0

R     ATGTATACGTGTGTACATATATACATATATACATGTATACGTGTGTACATATATACATATATACATATATACATATATACATGTATACATGTATACGTGTGTACATATATACATATATACATATATAC  1406
trnE  --------------------------------------------------------------------------------------------------------------------------------    60
trnF  --------------------------------------------------------------------------------------------------------------------------------     0

                                                                                                                                  trnF
R     ATATTTACATATATTTACATACATTTACATATTTACATATATTTATGCTTATGTGTTTACTTATATCCTCAACCCGGTACTTCCTCCACCAACAAACCAACCCCTGTAAGATGACAATAATATGTTAC  1534
trnE  --------------------------------------------------------------------------------------------------------------------------------    60
trnF  ----------------------------------------------------------------------------------------------------------------------------TTTC     4
                                                                                                                                  trnF
```

```
                                  trnF
R     CGTAACTTAATTAAAGTTTAATATTGAAGCTATTAGGATGGG-CCCTAAAAAGCC  1588
trnE  -------------------------------------------------------    60
trnF  TGTAGCTTAACCAAAGCAGGGCACTGAAGATGCCAAGATGGCACCCCATCGAACG    59
                                  trnF
```

1.111 NC_008225-NC_004377

Avg ovsize: -524

LCA: Gadiformes-order

1.111.1 trnP-trnE -34

```
                                  trnP                                                                        trnE
R     GAGACTTAAACTCCCACCCCCAGCTCCCAAAGCTGAGATTCTTCAT--TAAACTATCCTCTGCAACCCCAAATATAATTCTTGCCCGGGCTCTAACCAGGACCAATGACTTGAAAAACCACCGTTG--  124
trnP  -AGATTTTAACTCCTGCCCCTAGCTCCCAAAGCT-AGAATTTTAAAACTTAACTATTCTCTG------------------------------------------------------------------   60
trnE  ------------------------------------------------------------------------------------------------CAGGACCACTGACTTGAAAAACCAACGTTGTA   32
                                  trnP
                                                                                                                     trnE
```

```
       trnE
R     ATTCAACT  132
trnP  --------   60
trnE  ATTCAAC-   39
       trnE
```

1.111.2 nad6-cob -70

```
                                  nad6
R     TGC--CAACGCAACT-GCTCCTAAAATAAACACCCCTGACAATATCATTATTGA-ATAAGACATAATCCCTGCCCGGGATTAAACCGAGAATAAATATTTAAATATCCGCCTTTGACCTCAGGGACTT   124
nad6  AGAAGCAACAAAAATTACCCCTAAATTTATTACTGCT-AAAACAAC-TAATAGATATA----------------------------------------------------------------------   56
cob   --------------------------------------------------------------------------------------------------------------------------------   0
                                nad6
```

```
                                cob
R     AATGACCAGCCTTCGAAAAACCCACCCCCTCCTAAAAATCGCAAATGATGCCGTAACAGA-   184
nad6  -------------------------------------------------------------   56
cob   AATGACCAATTTACGAAAGACACACCCGCTCTTAAAAATTGTTAACGATGCTTTAATTGAC   61
                              cob
```

1.111.3 trnE-trnF -1470

```
                                  trnE
R     GCCCGGGCTCTAACCAGGACCAATGACTTGAAAAACCACCGTTG-A-TTCAACTACAAGAACA-AACAACGCATCTGCCAACTATAGTCCTTGAAATATAAAATGTCTAGTCATTATTCTATGTATTA   125
trnE  -C-TGAATTTTCATCAGGACCACTGACTTGAAAAACCAACGTTGTAATTCAACTACGGGAATATAAC-------------------------------------------------------------   65
trnF  --------------------------------------------------------------------------------------------------------------------------------   0
                                  trnE
```

```
R     ACACCATAAAAATATTTTAACCATACAGGAATTACTATCGTTTTCATTTTTCAAAACGAAGTAGGTATAATCACATTAACATAATTAAACCACCCTTTTTATCTACCACTCCCTATCCTCTTAAATAC   253
trnE  --------------------------------------------------------------------------------------------------------------------------------   65
trnF  --------------------------------------------------------------------------------------------------------------------------------   0
```

```
R     TCATTAAATCACCCGCTAGTCTCGCTTCTGTGGCCCACGTTCTCTTGTAAGGTCAAGGGCAAGACATTTGACTTCCCCTTTCAGTGAATTATTCCTGGCATTCAGCCTAGCTTCACGGCCATTAAAAG   381
trnE  --------------------------------------------------------------------------------------------------------------------------------   65
trnF  --------------------------------------------------------------------------------------------------------------------------------   0
```

```
R     TTTATTGTACATAACTTGCATTTTTGCGCATTTATGAATGGTGGAACACATCAATTGATAATCGCTTCGCTAGCCGAGCGCTCTTTCTAAAGGGTATTTGGTATTTTTTCTTCTTTCCTTTCATTTTA  509
trnE  --------------------------------------------------------------------------------------------------------------------------------  65
trnF  --------------------------------------------------------------------------------------------------------------------------------  0

R     CATTTCAGAGTGTAAACAAATGATTCTCTTAAGGTTGAACAAATTTCTTGAACTCAGGAAATAATGTATTATGTTTAATAATTATTCTTGCTAAATTGCATACCTGTAGCAAGAGCATAATACTATAT  637
trnE  --------------------------------------------------------------------------------------------------------------------------------  65
trnF  --------------------------------------------------------------------------------------------------------------------------------  0

R     TACTTATTCAGACTAACCTATCTTATGCCCCCTCCCGTTCGTCTAAACCCCCCTACCCCCCAGTTCCTAAAGTCTTATTCTGCAAACCCCCCGGAAACAGAAACTCCATGAACTGGACAACAAACAAT  765
trnE  --------------------------------------------------------------------------------------------------------------------------------  65
trnF  --------------------------------------------------------------------------------------------------------------------------------  0

R     TTAACCCATAACACCCCGCACCCCCAACCTTCCACACATACACACATACATATATACATATATACATGTATACGTGTGTACATATATACATATATACATGTATACGTGTGTACATATATACATATATA  893
trnE  --------------------------------------------------------------------------------------------------------------------------------  65
trnF  --------------------------------------------------------------------------------------------------------------------------------  0

R     CATATATACATGTATACGTGTGTACATATATACATATATACATGTATACGTGTGTACATATATACATATATACATGTATACGTGTGTACATATATACATATATACATGTATACGTGTGTACATATATA  1021
trnE  --------------------------------------------------------------------------------------------------------------------------------  65
trnF  --------------------------------------------------------------------------------------------------------------------------------  0

R     CATATATACATATATACATGTATACGTGTGTACATATATACATATATACATGTATACGTGTGTACATATATACATATATACATGTATACGTGTGTACATATATACATATATACATGTATACGTGTGTA  1149
trnE  --------------------------------------------------------------------------------------------------------------------------------  65
trnF  --------------------------------------------------------------------------------------------------------------------------------  0

R     CATATATACATATATACATGTATACGTGTGTACATATATACATATATACATATATACATATATACATGTATACGTGTGTACATATATACATATATACATGTATACGTGTGTACATATATACATATATA  1277
trnE  --------------------------------------------------------------------------------------------------------------------------------  65
trnF  --------------------------------------------------------------------------------------------------------------------------------  0

R     CATGTATACGTGTGTACATATATACATATATACATGTATACGTGTGTACATATATACATATATACATATATACATATATACATGTATACATGTATACGTGTGTACATATATACATATATACATATATA  1405
trnE  --------------------------------------------------------------------------------------------------------------------------------  65
trnF  --------------------------------------------------------------------------------------------------------------------------------  0
```

```
                                                                                                                                        trnF
R     CATATTTACATATATTTACATACATTTACATATTTACATATATTTATGCTTATGTGTTTACTTATATCCTCAACCCGGTACTTCCTCCACCAACAAACCAACCCCTGTAAGATGACAATAATATGTTA  1533
trnE  ------------------------------------------------------------------------------------------------------------------------------------    65
trnF  ------------------------------------------------------------------------------------------------------------------------------------     0
```

```
                          trnF
R     CCGTAACTTAA-T-TAAAGTTTAATATTGAAGCTATTAGGATGGGCCCTAAAAAGCC  1588
trnE  -----------------------------------------------------------    65
trnF  -CGTAGCTTAAATTTAAAGCTTAATGCTGAAGATATTAAGATGAAGCCTAAAAC-CT    55
                          trnF
```

1.112 NC_008831-NC_003979

Avg ovsize: -617
LCA: Harpacticoida-order

1.112.1 cox3-trnW -62

```
                             cox3                                                      trnW
R     GATGTGGTTTGGCTATTTCTGTATTTAGTTATTTATTGGTGAGG-AAA-CT-CGGTCTACTAAAAAACTTAGGTTAAATAAACCAATTAAATTTCAAATTTAATAATGAGAGAATCAGTTT  118
cox3  GATGTGGTGTGACTCTTTCTTTACTTAACTATTTATTGGTGGGGGAATTCTAGGGTCTA------------------------------------------------------------   59
trnW  -------------------------------------------------------------------------------------------------------------------------    0
                             cox3
```

1.112.2 trnS1-cox1 -771

```
                                    trnS1
R      AGTATTAAAATAACAAAATCTTTAAAATTTTTAATTTCTAATTAAAAACAAGCATATTAATGTTGGATTTTTGGCTTGGCTCTAAATTGGGGAATATTGGAAAAAGAGGCTCAAAACAGAGGGGAGCC 128
trnS1  A------------------------------------------------------------------------------------------------------------------------------- 1
cox1   -------------------------------------------------------------------------------------------------------------------------------- 0
      trnS1

R      GGCGAAAGCCCTAAATTAGAAAATTAGGGGTAGGGAGGCTGTAAACCGGCCATTTGGACGTTTGCGTGTAATTAATTTTTGAAGATTAAATGGGCGCCGATCCGCGGTCGGGGGAGGTCATCGCTGGT 256
trnS1  -------------------------------------------------------------------------------------------------------------------------------- 1
cox1   -------------------------------------------------------------------------------------------------------------------------------- 0

R      ATGCCTGGGTGTAGGGCGGATGTCCTAGCTCTGCGCTTGACGGCTGATTATCGCTGAAAAAAAGAAAGAAAAAAAGAAAAGTTGAAAATTTTTGTAGATACACAGTAACCCCCCGGGGGGTTGGTAAG 384
trnS1  -------------------------------------------------------------------------------------------------------------------------------- 1
cox1   -------------------------------------------------------------------------------------------------------------------------------- 0

R      AGTGGGTACCAAAGGGGAGAGGAAGAATTATATCAGCTAGAAATTTCTTATTAGGGGTTGAAGGGCAATTAAAAAAAAGTTGTGTGTACCAAGTTACAAAAATTTCCAAGTTTAGATTAAAAGACAGA 512
trnS1  -------------------------------------------------------------------------------------------------------------------------------- 1
cox1   -------------------------------------------------------------------------------------------------------------------------------- 0

R      AAACAAAATTTTTTAAACTGAGGGAGTGGGCAGTAATTACCTGGCGAGCCTAGGAGCCTGGGGGGGTCAAGTAGATTCTTGGCTTGGCTCTAAATTGGGGAATATTGGAAAAAGAGGCTCAAAACAGA 640
trnS1  -------------------------------------------------------------------------------------------------------------------------------- 1
cox1   -------------------------------------------------------------------------------------------------------------------------------- 0

R      GGGGAGCCGGCGAAAGCCCTAAATTAGAAAATTAGGGGTAGGGAGGCTGTAAACCGGCCATTTGGACGTTTGCGTGTAATTAATTTTTGAAGATTAAATGGGCGCCGATCCGCGGTCGGGGGATGTTT 768
trnS1  -------------------------------------------------------------------------------------------------------------------------------- 1
cox1   -------------------------------------------------------------------------------------------------------------------------------- 0
```

cox1

```
R       AGAATAAAGTGGAGAGGGGCTACGAACCACAAAGATATTGGTACTTTATAC-TTGTTGGCGGG   830
trnS1   ---------------------------------------------------------------     1
cox1    ----TAAAATGGAGAGGGGCGACTAATCATAAAGATATTGGTACATTATACCTTATTGCTGGG    59
```

cox1

1.112.3 trnW-rrnL -1020

trnW

```
R      AAACTTAGGTTAAATAAACCAATTAAATTTCAAATTTAATAATGAGAGAATCAGTTTTTAGTTTTACCGAGAACAAAGTAATTTTCTTTTTGCATTATGAGGTTTTAAAAATCTTAAATATTTTAGAG   128
trnW   --------------------------------------------------------------------------------------------------------------------------------     0
rrnL   --------------------------------------------------------------------------------------------------------------------------------     0

R      CTCTAAGATTTCATTTTTTAGTTATTGTCTATACCCGTTTTAAAGGGTTGAATAAAATAACTTAAAGCAGGATATCTGATATTGAAATTTTGTAGTTGTGGTTTATCAAATTCATTTTGAAGAAAAGG   256
trnW   --------------------------------------------------------------------------------------------------------------------------------     0
rrnL   --------------------------------------------------------------------------------------------------------------------------------     0

R      TTTTCTAAGTATCAAGGAGGGATTAGCTTTCTTGAATTAAAATCTTAAAATAATATCCCTAGTAGAATGTAATAATTCAAGGCTAAATAAAATTTATGTCTATCTACCACATCAAAACTAACTTAAAA   384
trnW   --------------------------------------------------------------------------------------------------------------------------------     0
rrnL   --------------------------------------------------------------------------------------------------------------------------------     0

R      AATTAAATCTTTAGTAAAAATTCAGTGAAACCCAATATTAGAACAGTTTTAAGTGTTAGAAAATTTTTAGCAGTCGTGGAACTTGGTTTAGGTTAATCTGTTTGCCAAATACTTAGGAGAATAATCTC   512
trnW   --------------------------------------------------------------------------------------------------------------------------------     0
rrnL   --------------------------------------------------------------------------------------------------------------------------------     0

R      TAAAGCTCTGCCCGGTGATAAAAATTAAACGGCTTGACAAGGTAGCAAAATCAATTGTTCTTTAATTGAGAACCAGAATGAACGGCTTAATTAACTTAAAATATCAGACCTGTTTCGTTTAAATTAAT   640
trnW   --------------------------------------------------------------------------------------------------------------------------------     0
rrnL   --------------------------------------------------------------------------------------------------------------------------------     0
```

```
R     ATTCTGAGTGCAAATACTTAGACTAAATTCAGGGACGAGAAGACCCTAAAATCTTATTTACTTATTTAGATTATTATAAAAAATTAATTGGTTGGGGCAACCTAATAAATGTAAATACTTTTTATAGT   768
trnW  --------------------------------------------------------------------------------------------------------------------------------     0
rrnL  --------------------------------------------------------------------------------------------------------------------------------     0

R     CTAAACTTGAACTCTGCAACTTATTACCAAGATACTTTAGGGATAACAGCATTAAGGGCCTTGGAGTTCATATCTACTGTGCCTGAATGACCTCGATGTTGAATTAAGAAACCTTCTAAGAGAAAAAG   896
trnW  --------------------------------------------------------------------------------------------------------------------------------     0
rrnL  --------------------------------------------------------------------------------------------------------------------------------     0

                                                                                                  rrnL
R     CCTAGAAGTTCAGTCTGTTCGACTGGTATTTTCTTACATGATTTGAGTTAAGATCGACGTAAGTCAGATTGGTTTCTATCTTGAATTTTCTTTGATTTTGATTAGTACGAAAGGAATTTAAAAA  1020
trnW  ----------------------------------------------------------------------------------------------------------------------------     0
rrnL  ----------------------------------------------------------------------------------------------------------------------------     0
```

1.113 NC_007440-NC_006405

Avg ovsize: -667

LCA: Neobatrachia-suborder

1.113.1 nad6-cob -86

```
                                     nad6                                                                               cob
R     CG-A--CGGATTTGAGGCCACAGCCAATATTCCCAATAATAAACACAACTCCTTTATCACTATTTCTGCTAGGTACTTAACCTAGTCTGTATAATCTACCTTCTAGTTACAGAAATTATGGCACCTCT   125
nad6  GGGAGAAGGATTTGAAGCAACAGCCACAAATCCAACCAATAGTCATAATTCAAATACCA-TA------------------------------------------------------------------    61
cob   --------------------------------------------------------------------------------------------------------------------------------     0
                                    nad6
```

```
        cob
R       ACGTAAATCTCACCCAATTCT   146
nad6    ---------------------    61
cob     --------------------T     1
                            cob
```

1.113.2 cob-trnE -340

```
                                               cob
R       GTAAGCATCATTCCCCACAACAAGCATCGCGCAGTTATGAGCCACCATCTGCCGTTTTAAGCGTCAAATAAGCCATCCTACGAAGACTCCACCTAAATTGATGGCACATGACTATCTTCCTCAATGGG   128
cob     --------------------------------------------------------------------------------------------------------------------------------     0
trnE    --------------------------------------------------------------------------------------------------------------------------------     0

R       CCTTGATTAACGACCTATACTGGTTCACTCATTCAAACACATTTAAAAATCCCCATAATTATTTTCCCCATGAATTTGATCCTCTACTACCCTACGAAATGGTCTACCCTTACGCTAGGGGTTGGGCC   256
cob     --------------------------------------------------------------------------------------------------------------------------------     0
trnE    --------------------------------------------------------------------------------------------------------------------------------     0

                                                                                                                      trnE
R       GTCCACCCTCTACCAACAAGCCGCCAAACACATCCTAGATCAACCCTGAATCACACCAACACACAACAATTTGAACATGCCTCCATATTTCTGCCAGGACTTCAACCTAAACTTGTAATCTGAAAAAC   384
cob     --------------------------------------------------------------------------------------------------------------------------------     0
trnE    ------------------------------------------------------------------------------------ATATTCCTGCCAGGACTCTAACCTGGACCAACAGTATGAAAAAC    44
                                                                                                                      trnE

           trnE
R       TACTGTTGTTGTTCAA-   400
cob     -----------------     0
trnE    TGCCGCTGTAATTCAAC    61
           trnE
```

1.113.3 trnE-trnL1 -1577

```
                                            trnE
R      CAGGACTTCAACCTAAACTTGTAATCTGAAAAACTACTGTTGTTGTTCAACTACAGAAACTTCCCCATAAAATGATTCTTCCCCCTATTATGCCCGTTTTTAGTTAGTAGGGTAAAACATCTTATGAT   128
trnE   CAGGACTCTAACCTGGACCAACAGTATGAAAAACTGCCGCTGTAATTCAACTACAAGAACT-------------------------------------------------------------------    61
trnL1  --------------------------------------------------------------------------------------------------------------------------------     0
                                            trnE

R      TTGCCCCCCCCCATCTCTAGTCACTATTTATCTCTCAGTATCCCCATAAGATGATTCTTCCCCATATGTCTATTTACACTTTATTGGGTAAAACATTTTATGATCAACCTCATTCATTTCTAACCATC   256
trnE   --------------------------------------------------------------------------------------------------------------------------------    61
trnL1  --------------------------------------------------------------------------------------------------------------------------------     0

R      ATTTATCTCTCAGTATCCCCATAAGATGATTCTTCCTTTATATGTCCGTCTATACTTTATTGGGTATAACATTTTATGATCACCACATTCATTTCTAACCATCATTTATCTCTCAGTATCCCCATAAA   384
trnE   --------------------------------------------------------------------------------------------------------------------------------    61
trnL1  --------------------------------------------------------------------------------------------------------------------------------     0

R      ATGATTTACCCATATATATGTTTAAGACATATATGTTTAATCCCCATTCATGTCTAGTCACCACTCATTCACCCATATTTATGGTCATTGAACACATGAAGACCCAACAAATAAAAAATATCCCTTTT   512
trnE   --------------------------------------------------------------------------------------------------------------------------------    61
trnL1  --------------------------------------------------------------------------------------------------------------------------------     0

R      TAATGTTTGCCTGCGCCCATATTATGACTACTTGATTGGACCTTCCCTTGCCCGGGTAAATTCGTATCTTAATCTAAGTGAGTCCAACTGGTTGATCCCTCCGTCTTAATCTTCATAAAATTTATGTT   640
trnE   --------------------------------------------------------------------------------------------------------------------------------    61
trnL1  --------------------------------------------------------------------------------------------------------------------------------     0

R      ACGAATAAACTCTAAAATGGTTTTCTTTACACCTTAAGTCTCCCATACCTTCTTTACCTATAGTTAATAATGCATCATTCATCCTACGAAGACTCCACCCAAATTGATAGCACCATGAATATTTTTTT   768
trnE   --------------------------------------------------------------------------------------------------------------------------------    61
trnL1  --------------------------------------------------------------------------------------------------------------------------------     0

R      CAATGGACCTTCATTAACGACCTATACTGGTTCACTCATTCAAACACACTTAAAAATCTCCCATAATTATTTTCCCCTCATGAATTGATCTTTTACTACCCTTCGTTACTACCACGAATCCTATTCTT   896
trnE   --------------------------------------------------------------------------------------------------------------------------------    61
trnL1  --------------------------------------------------------------------------------------------------------------------------------     0
```

```
R      TCACCCAACCTTACGAGACGGTCCACGGTTGGTTTAAGGACTGGATCGTCCTCAGTTTCCAATGGTCCAAATTTGTAGAGTTACAGCCGTCGATCTTACGTTCATCTGACGGATGTGAATCTGTGGGA  1024
trnE   --------------------------------------------------------------------------------------------------------------------------------    61
trnL1  --------------------------------------------------------------------------------------------------------------------------------     0

R      CCCATATTGAAGAGACGCTGCGATCGATCTTCAATAAATATCCCTCCTATGAGCGAGGGACCTACCTACAAGATAAGACCACCCTTGAACTACTGAGGCATTGGTAGGGGTTTTTATAGGGGAGGTTT  1152
trnE   --------------------------------------------------------------------------------------------------------------------------------    61
trnL1  --------------------------------------------------------------------------------------------------------------------------------     0

R      CATCCACATCTCAGAGTGTCCGCTATGGCATGGTGAGAGGTCCGGACTATACCTTAGTCCCCATTGCAGGTTATTGTTCCCATATCTATGAATGCTCGACGGACATAGAGACTACCCTGATATTTCCT  1280
trnE   --------------------------------------------------------------------------------------------------------------------------------    61
trnL1  --------------------------------------------------------------------------------------------------------------------------------     0

R      CCCACCTTTAGGATTTACGCTCAAATTTGATTTTTTCGCTTTTTTTCATCTTTTTTTCATCTTTTTTGAAAAATTCAAAAAATCCATTTTTACCCATTTTTACCCATTTTTACCCATTTTTCTCTTTT  1408
trnE   --------------------------------------------------------------------------------------------------------------------------------    61
trnL1  --------------------------------------------------------------------------------------------------------------------------------     0

R      TTTGCTCCTACCCCCCCCCTTCCCCCCCCCCAACTCTTTTTTCCTATTAAATTCCCCTAATCCCCCCCGAGTTAGGTTTAAAAAAAAAAAGCCAATTGGGCCCCCCCGAAAAAAATTTCTTATACTTAG  1536
trnE   --------------------------------------------------------------------------------------------------------------------------------    61
trnL1  --------------------------------------------------------------------------------------------------------------------------------     0

                                                                                                                  trnL1
R      ATTTACACTTTTTTTTTTTCTGCAGAACTTGTATAAGTTTTTAACAATAACAAAAACCTTTATACCCTACGCAGAAAATACTACATCAAAAAGCTAAGACTGCTTTTAATGGAAAAGAGCT-TTCCTC  1663
trnE   --------------------------------------------------------------------------------------------------------------------------------    61
trnL1  ------------------------------------------------------------------------------------------------------CTTTTAAAGGAAAATAGCCCTTCCCC    26
                                                                                                                  trnL1

                 trnL1
R      TGGTTTTAGGGGCCAGCTAA-TCTTGGTGCAAGT  1696
trnE   ---------------------------------    61
trnL1  TGGTCTTAGGCTCCAG-TATCTCTTGGTGCAAAT    59
                 trnL1
```

1.114 NC_008448-NC_004594

Avg ovsize: -793
LCA: Galaxiidae-family

1.114.1 nad4-trnG -464

```
                                              nad4
R     AAAATATTAGATTATGATCTAAAGATGGGGGTTAAATTCCTCTTATTTAGCCAGGAGAGGCTCGAAGCACTGGTGACTGCTGCAGTATTATTAGCTTCTGGCGTCACCGTCACGTGAGCCCACCATAG   128
nad4  A-------------------------------------------------------------------------------------------------------------------------------     1
trnG  --------------------------------------------------------------------------------------------------------------------------------     0
      nad4

R     CATTATAGAAGGCGAACGAAAACAGGCCATTCAGTCTTTAGTCCTAACTATTTTGCTAGGCTTCTATTTTACCTTCCTTCAAGCTATGGAATATTATGAAGCCCCCTTTACCATTGCTGATGGAGTAT   256
nad4  --------------------------------------------------------------------------------------------------------------------------------     1
trnG  --------------------------------------------------------------------------------------------------------------------------------     0

R     ACGGTTCAACCTTTTTTGTAGCTACAGGTTTCCACGGCCTCCATGTAGTTATCGGCTCAACTTTTTTAGCTGTCTGCCTACTCCGACAAATAATGTATCATTTTACATCTAAACATCACTTCGGCTTC   384
nad4  --------------------------------------------------------------------------------------------------------------------------------     1
trnG  --------------------------------------------------------------------------------------------------------------------------------     0

                                                                                                                   trnG
R     GAAGCAGCCGCCTGATATTGACATTTCGTTGATGTCGTGTGACTTTTCCTTTATGCCTCTATTTATTGATGAGGCTCATAGTCTTTCTAGTATTAATATTAGTATAAGTGACTTCCAATCACGCAGTC   512
nad4  --------------------------------------------------------------------------------------------------------------------------------     1
trnG  ---------------------------------------------------------------------------------TCTTTCTAGTATTAA-GTTAGTATAAGTGACTTCCAATCACACGGTC    46
                                                                                                                    trnG
```

```
               trnG
R      TTGGTTA-AA-CC   523
nad4   -------------     1
trnG   TTGGTTAGAGTCC    59
               trnG
```

1.114.2 cox3-nad4l -487

```
                                  cox3
R      -CATTTCGTTGATGTCGTGTGACTTTTCCTTTATGCCTCTATTTATTGATGAGGCTCATAGTCTTTCTAGTACTAATATTAGTATAAGTGACTTCCAATCACGAAGTCTTGATTAAGCCCCAAAGAAG   127
cox3   GCACTTCGTTGACGTTGTCTGATTATTCTTGTATGTCTCAATTTATTGATGAGGCTCATA--------------------------------------------------------------------    60
nad4l  --------------------------------------------------------------------------------------------------------------------------------     0
                                  cox3

R      ACAATAATTTAATTATGCTATCTTTACTATCGCAGGCCTCCGCTCAAGAACTTTAACCCTTACGTCTTTTTGGGTATCTTAAATGTTCATATGCCGAAAAACTCTTGCCCTGTGAGTATGTGTATAAC   255
cox3   --------------------------------------------------------------------------------------------------------------------------------    60
nad4l  --------------------------------------------------------------------------------------------------------------------------------     0

R      CCATTAGGATCCGCCTACCTCCCCCTTTCTATCCAATTTTTTCTAGTAATAATCTTATTATTACTCTTCCGCTTAGAAATTGCTCTCTACATCCCCTTCCTTAAGGAGACCCACTAATGCTACCTCTA   383
cox3   --------------------------------------------------------------------------------------------------------------------------------    60
nad4l  --------------------------------------------------------------------------------------------------------------------------------     0

R      ACCACTTTTATTTGAACTGTAGTTGCTCTTATCTTGCTAACCTCGGCTTAACTTATAATTATTGCAGTGGAAGGACCTAAAATAAGATGAAAAGGTGGTTAATCCAAAACAAGACCTCTGATTTCGAC   511
cox3   --------------------------------------------------------------------------------------------------------------------------------    60
nad4l  --------------------------------------------------------------------------------------------------------------------------------     0
```

```
                                                      nad4l
R      TCAGAAAATTATGGTTTAAATCCATAACCCCCTTATGACCCCCACCCTTTTTACCTTCTCTTCAGCTTTTATTCTAGGATTAATAGGTCTGG-C  604
cox3   ----------------------------------------------------------------------------------------------   60
nad4l  -----------------------------------TGACTCCAGTACACTTTACCTTCACTTCAGCCTTTGTCCTAGGGCTAATAGGATTAGCC   59
                                                      nad4l
```

1.114.3 trnR-trnH -1428

```
                   trnR
R      TCCAAAACAAGACCTCTGATTTCGACTCAGAAAATTATGGTTTAAATCCATAACCCCCTTATGACCCCCATACATTTTACCTTCTCCTCAGCTCTTGTTCAAGGACTAACAGGCCGGGCTTTTCACCA  128
trnR   TCTAAGAGAAGACCTCTGATTTCGGCTCAGACAATTATGGTTTAAGTCCATAACCGCCTT--------------------------------------------------------------------   60
trnH   --------------------------------------------------------------------------------------------------------------------------------    0
                   trnR

R      TAAGCATCTTCTACCCACGCTATGTGTCTAGAAGGTATTATACTTTCTTTATTTATTGCACCCTCTCAATGAAACCTTCAATTAGATTATACCGGACTTTCCTCCGCCCTATGCTACTACTTGCTTTT  256
trnR   --------------------------------------------------------------------------------------------------------------------------------   60
trnH   --------------------------------------------------------------------------------------------------------------------------------    0

R      TCTGCTTGCAGAGCAACTACAGGCTTAACATCATTAGTTACCACTGCTCGCACTGACAGCCCCCAACGCTTAAAAGTATAAACCTTTTATAGTACTAAATATTTTAATCCCTGCCTTAATAATACTCC  384
trnR   --------------------------------------------------------------------------------------------------------------------------------   60
trnH   --------------------------------------------------------------------------------------------------------------------------------    0

R      CAACTATTTGATTGTCCTCTACTAAATAACTCTGATCTTCTTTTGTAATTCAAAGCCTTGTAATTGCTTAATTAAGCCTCTTTTGATTTAAAAATACCAATGAAGTCGGTTGGTCGCCCACAAATCTT  512
trnR   --------------------------------------------------------------------------------------------------------------------------------   60
trnH   --------------------------------------------------------------------------------------------------------------------------------    0

R      TTTTGCAGTTGACCCCCTATTTTCCCCTTCTTACCCTCAACTGCTAACTCCTCCCTCTTAATATTTAGGCAAGCCAGAACCATACTTCTTTAGGACCAATCACTCGACAACATACTCTAATGCTATTA  640
trnR   --------------------------------------------------------------------------------------------------------------------------------   60
trnH   --------------------------------------------------------------------------------------------------------------------------------    0
```

```
R     GTCTCTTTACAAGTATTTTTAATTTTAGCATTTAATGCAACCGGATAATTATATCTTATATAATGTTTGAAACCACACTTCTCTCAACTTTAGTTATTATTGCCCGCTGGGGCAAGCAAACAGAACAT   768
trnR  --------------------------------------------------------------------------------------------------------------------------------   60
trnH  --------------------------------------------------------------------------------------------------------------------------------   0

R     TTAAACGCCAATACCTATTTTTATTTAATACACTTGCAGGGTCTTTGCCCTTACTGATTGCTTTACTAGCCCTTCAAAAGCATACAAGTACCTTATCAATACTTGTGACACAATTCATTCAGCCATT   896
trnR  --------------------------------------------------------------------------------------------------------------------------------   60
trnH  --------------------------------------------------------------------------------------------------------------------------------   0

R     AAATTCAATCTCCTCAACTTATAAATCATGTTAAGCACATGCCTTCTAGCTTTTCAGTTAGAATACCTCTTTATGGCACACACCTTTGACTTCCCAAAGCCCACTTAGAAACCCCAATTGATGGCTCT   1024
trnR  --------------------------------------------------------------------------------------------------------------------------------   60
trnH  --------------------------------------------------------------------------------------------------------------------------------   0

R     ATAGTCCCTGTTGTAATGCTCCTAAAATTAGGAGCCTGCCGTATAATACAAACTATACCATACTAGAGCCCCTAGACAAAGAACTTGCACCCCCGTTTATTATTTTAGCTCCGTAAGAGATTGCCACA   1152
trnR  --------------------------------------------------------------------------------------------------------------------------------   60
trnH  --------------------------------------------------------------------------------------------------------------------------------   0

R     ACTGTTCTATCTGCCTACATCAGACAGGCCTAAAAGCTCTTATTGCATATTCTTCTGTTAGGATATGGGCCTTGTGACAGGAGGTATTTTAATTCAGACCCCTTAAGGATTCACAGAAGATGTTATTC   1280
trnR  --------------------------------------------------------------------------------------------------------------------------------   60
trnH  --------------------------------------------------------------------------------------------------------------------------------   0

R     TTATAATTGCACGTCATCTCACCTCCCCAGCTCTTTTCTGCTTAGCTAACACCAGCTATGGATGAACCTGGAGTCAAACTTTACTTTTAACCCGCGGGATACAAATTACTTTAGCCTTAATAATAATA   1408
trnR  --------------------------------------------------------------------------------------------------------------------------------   60
trnH  --------------------------------------------------------------------------------------------------------------------------------   0

                                                                                                          trnH
R     TGGCAACTCATTGCTAATATGGTTAACCTGGCTTTAACCCCCCCCGCTGGGGGGGGGGGGGGGGGGGCCTGACTATTCTGTATATATAGTTTAATCAAAATATTAGATTGTGATTCTAAAGATAGGGG   1536
trnR  --------------------------------------------------------------------------------------------------------------------------------   60
trnH  --------------------------------------------------------------------------------TAGATATAGTTTAGCCAAAACATCAGATTGTGATTCTGAAAACAGGGG   48
                                                                                                          trnH
```

```
         trnH
R       TTAAACTC-CC  1546
trnR    -----------    60
trnH    TTAAACCCCCC    59
         trnH
```

1.115 NC_008683-NC_015076

Avg ovsize: -940
LCA: Otocephala-clade

1.115.1 trnQ-trnM 2

```
                          trnQ                                                trnM
R       GGGATCGAACCCATGCCCA-AGAGATCAAAACTCTTAGTGCTTCCTCTACACCACTTTCTAAGATGGGGTCAGCTAATCAAGCTTTCGGGCCCATACCCCGAACATGACGGTTAAAGTCCC-   120
trnQ    GGATTTGAACCCA-GCCCGCAGAGATCAAAACTCTGAGTGCTCCCGCTACACCACTCTCTGA-AT-----------------------------------------------------------    63
trnM    ----------------------------------------------------------------ATATGGTAAGCTAAAAAAGCTTTCGGGCCCATACCCCGAATATGTTGGTTAAACCCCCT    59
                          trnQ
                                                                                      trnM
```

1.115.2 trnI-trnV 0

1.115.3 rrnS-trnI -2822

```
R     GA--CAAGGGGAGGCAAGTCGTAACATGGTAAGTGTACCGGAAGGTGCACTTGGATTAAACCCAGGGCGTGGCTGAGTTAGTCAAGCATCTCACTTACACCGAGAAGACATCCATGCAAGTTGGATCG   126
rrnS  GCCTCAAGGGGAGGCAAGTCGTAACATGGTAAGTGTACCGGAAGGTGCACTTGGAAGAA---------------------------------------------------------------------    59
trnI  --------------------------------------------------------------------------------------------------------------------------------     0

R     CCCTGAGCCAAACAGCTAGCTTAACCACCAATATAACCTAACAATGTTAATAACAAAACATGACTTAACACCACAAACTAAACCATTTTTTTACCTGAGTATGGGAGACAGAAAAGGTTCAACCCAAA   254
rrnS  --------------------------------------------------------------------------------------------------------------------------------    59
trnI  --------------------------------------------------------------------------------------------------------------------------------     0

R     GCAATAGAAAAAGTACCGCAAGGGAAAGCTGAAAGAGAAATGAAACAACCCATATAAGCACTAAAAAACAAAGACTAAACCTTGTACCTTTTGCATCATGATTTAGCCAGCACCCTCAAGCAAAGAGA   382
rrnS  --------------------------------------------------------------------------------------------------------------------------------    59
trnI  --------------------------------------------------------------------------------------------------------------------------------     0

R     CCTTTAGTTTGAAACCCCGAAACCAGGTGAGCTACCCCGAGACAGCCTATTTAAATTTAGGGCTAACCCGTCTCTGTGGCAAAAGAGTGGGAAGAGCTCCGGGTAGAAGTGATAGACCTACCGAACCT   510
rrnS  --------------------------------------------------------------------------------------------------------------------------------    59
trnI  --------------------------------------------------------------------------------------------------------------------------------     0

R     GGTGATAGCTGGTTGCCTGAGAAGTGGATAGAAGTTCAGCCTCGTACGCCCCAAATCAAAAAATATAACATTAAGACATAAGGGATACATACGAGAGTTAGTTAAAGGGGGTACAGCCCCTCTAACAA   638
rrnS  --------------------------------------------------------------------------------------------------------------------------------    59
trnI  --------------------------------------------------------------------------------------------------------------------------------     0

R     AGGATACAACCTTCACAGGAGGATAAAGATCATAATATATAAAACATGCTGTTTTAGTGGGCCTAAAAGCAGCCATCTAAATAGAAAGCGTTAAAGCTCAGACAGAAAGAAGTTTATTATACTGATAA   766
rrnS  --------------------------------------------------------------------------------------------------------------------------------    59
trnI  --------------------------------------------------------------------------------------------------------------------------------     0
```

```
R     AAAATCTTATTCCCCTAACAATATCAGGCTAACCCATGCCCACATGGAAGAAATTATGCTAAAATGAGTAACAAGAAGACCTGCTCTTCTCCAAGCACAAGTGTAAGCCAGATCGGACAGACCACTGG   894
rrnS  --------------------------------------------------------------------------------------------------------------------------------    59
trnI  --------------------------------------------------------------------------------------------------------------------------------     0

R     AAATTAACGAACCCAACCCAAGAGAGTAATGTGAATAATAGAAAAACCAAGAAAAACCCACAACCAAACAATCGTTAACCCCACACTGGAGTGCTATTTTTAAAGGAAAGACTAAAAGAAAGGGAAGG  1022
rrnS  --------------------------------------------------------------------------------------------------------------------------------    59
trnI  --------------------------------------------------------------------------------------------------------------------------------     0

R     AACTCGGCAAACACAAGCCTCGCCTGTTTACCAAAAACATCGCCTCCTGCAACTAAACTGAGTATAGGAGGTCCAGCCTGCCCAGTGACTACGGGTTCAACGGCCGCGGTATTTTGACCGTGCAAAGG  1150
rrnS  --------------------------------------------------------------------------------------------------------------------------------    59
trnI  --------------------------------------------------------------------------------------------------------------------------------     0

R     TAGCGCAATCACTTGTCTTTTAAATAGAGACCTGTATGAATGGCTAAACGAGGGCTTAACTGTCTCCCCCTTCAAGTCAGTGAAATTGATCTATCCGTGCAGAAGCGGGTATAACCATACAAGACGAG  1278
rrnS  --------------------------------------------------------------------------------------------------------------------------------    59
trnI  --------------------------------------------------------------------------------------------------------------------------------     0

R     AAGACCCTTTGGAGCTTAAGGTACAAAATTCAACCACGTTAAGCAACTTAATAAAAAGCAAAAACTTAGTGGAAAATGAAATTTTACCTTCGGTTGGGGCGACCACGGAGGAAAAACAAGCCTCCGAG  1406
rrnS  --------------------------------------------------------------------------------------------------------------------------------    59
trnI  --------------------------------------------------------------------------------------------------------------------------------     0

R     TGGAATGGGCCAAACCCCTAAAACCAAGAGAAACATCTCTAAGCCACAGAACATCTGACCAAAAATGATCCGGCTAATAAAGCCGATCAACGAACCAAGTTACCCTAGGGATAACAGCGCAATCCTCT  1534
rrnS  --------------------------------------------------------------------------------------------------------------------------------    59
trnI  --------------------------------------------------------------------------------------------------------------------------------     0

R     CCCAGAGTCCATATCGACGAGGGGGTTTACGACCTCGATGTTGGATCAGGACATCCTAATGGTGCAGCCGCTATTAAGGGTTCGTTTGTTCAACGATTAAAGTCCTACGTGATCTGAGTTCAGACCGG  1662
rrnS  --------------------------------------------------------------------------------------------------------------------------------    59
trnI  --------------------------------------------------------------------------------------------------------------------------------     0

R     AGCAATCCAGGTCAGTTTCTATCTGTAACGCTACTTTTCCTAGTACGAAAGGATCGGAAAAGAGGGGCCCATACTTAAAGCACGCCCCACCCCTAATTAATGAAAACAAATAAATTAAACAAAGGGAG  1790
rrnS  --------------------------------------------------------------------------------------------------------------------------------    59
trnI  --------------------------------------------------------------------------------------------------------------------------------     0
```

```
R     GGCCAAAACCCCAACCGCCCGAAATAAGGACATACTGGGGTGGCAGAGCATGGTAAATTGCGAAAGGCCTAAGCCCTTTAAACCAGAGGTTCAAATCCTCTTCCCAGTTTATGCTAAACACTCTAATA  1918
rrnS  --------------------------------------------------------------------------------------------------------------------------------    59
trnI  --------------------------------------------------------------------------------------------------------------------------------     0

R     AACCACCTGATCAACCCCCTAGCCTACATCGTGCCTGTCCTGCTAGCAGTAGCCTTCCTAACTCTAATCGAACGCAAAGTATTAGGATACATACAACTACGAAAAGGGCCAAATGTGGTCGGACCATA  2046
rrnS  --------------------------------------------------------------------------------------------------------------------------------    59
trnI  --------------------------------------------------------------------------------------------------------------------------------     0

R     CGGTCTCCTACAACCTATCGCCGACGGAGTAAAATTATTTATTAAAGAGCCAGTCCGCCCTTCTACATCCTCCCCATTCCTATTCTTAGCCACCCCCATTCTTGCATTAACTCTAGCCCTAACTCTAT  2174
rrnS  --------------------------------------------------------------------------------------------------------------------------------    59
trnI  --------------------------------------------------------------------------------------------------------------------------------     0

R     GAGCACCCATACCCATACCATACCCAGTAATTGATCTCAACCTAGGAGTTCTCTTTATCCTAGCCCTATCAAGCCTCGCAGTATATTCTATTCTAGGATCAGGATGAGCATCAAATTCAAAATACGCA  2302
rrnS  --------------------------------------------------------------------------------------------------------------------------------    59
trnI  --------------------------------------------------------------------------------------------------------------------------------     0

R     CTAATCGGAGCCCTACGGGCCGTAGCCCAAACAATTTCCTATGAAGTAAGCCTCGGACTAATCCTTCTATCAGTAATTATCTTCTCAGGAGGCTACACCCTACAAACATTTAGTACGGCCCAAGAAAG  2430
rrnS  --------------------------------------------------------------------------------------------------------------------------------    59
trnI  --------------------------------------------------------------------------------------------------------------------------------     0

R     TATCTGACTATTAGCCCCTGCATGACCATTAGCCGCAATATGATACATCTCAACCCTGGCAGAAACTAACCGAGCACCATTCGACCTAACAGAAGGAGAATCAGAGCTAGTCTCAGGCTTCAACGTAG  2558
rrnS  --------------------------------------------------------------------------------------------------------------------------------    59
trnI  --------------------------------------------------------------------------------------------------------------------------------     0

R     AATATGCAGGAGGGCCCTTCGCCCTCTTCTTCCTAGCCGAGTATGCAAACATCCTATTAATAAATACCTTATCAGCTGTACTATTTCTGGGAACATCACACATCCACCACATCCCAGAATTAACAACA  2686
rrnS  --------------------------------------------------------------------------------------------------------------------------------    59
trnI  --------------------------------------------------------------------------------------------------------------------------------     0

R     ATTAGCCTTATGACTAAGGCCGCGCTACTCTCTATTGTATTCCTCTGAGTACGAGCCTCGTATCCACGATTCCGATACGACCAACTAATACACCTTGTATGAAAAAACTTCCTCCCCCTAACATTGGC  2814
rrnS  --------------------------------------------------------------------------------------------------------------------------------    59
trnI  --------------------------------------------------------------------------------------------------------------------------------     0
```

```
                                                                                                                           trnI
R      TCTAGTACTATGACACATTGCCCTGCCAATCGCACTAGCGGGCCTTCCCCCACAACTATAGTTCAGGAACTGTGCCCGAATGCCTAGGGACCACTTTGATAGAGTGGCCTATAGGGGCTAAAATCCCC  2942
rrnS   ------------------------------------------------------------------------------------------------------------------------------------  59
trnI   -----------------------------------------------------------------GGAACTGTGCCTGACCGCTTAAGGGGCACTTTGATAGAGTG-ACTAAAGGGGGTTGAA----C  58
                                                                                                     trnI
```

```
        trnI
R      TCAGTTCTT  2951
rrnS   ---------    59
trnI   TC-------    60
       trnI
```

1.116 NC_008683-NC_008667

Avg ovsize: -962
LCA: Cyprinoidea-superfamily

1.116.1 trnI-trnV 0

1.116.2 nad1-trnQ -71

```
                                 nad1
R      CTAGTACTATGACACATTGCCCTGCCAATCGCACTAGCGGGCCTTCCCCCACAACTATAGTTCAGGAACTGTGCCCGAATGCCTAGGGACCACTTTGATAGAGTGGCCTATAGGGGCTAAAATCCCCT  128
nad1   CTAGTACTATGACACATTGCCCTGCCAATCGCACTAGCAGGCCTCCCCCCACAACTATAATTCA----------------------------------------------------------------  64
trnQ   --------------------------------------------------------------------------------------------------------------------------------  0
                                 nad1
```

```
                       trnQ
R     CAGTTCTTAGAAAGAAGGGGATCGAACCCATGCCCAAGAGATCAAAACTCTTAGTGCTTCCTCTA-   193
nad1  ------------------------------------------------------------------   64
trnQ  -------TAGAAAGAAGGGGATCGAACCCATGCCCAAGAGATCAAAACTCTTAGTGCTTCCTCTAC   59
                        trnQ
```

1.116.3 rrnS-trnI -2816

```
                             rrnS
R     GACAAGGGGAGGCAAGTCGTAACATGGTAAGTGTACCGGAAGGTGCACTTGGATTAAACCCAGGGCGTGGCTGAGTTAGTCAAGCATCTCACTTACACCGAGAAGACATCCATGCAAGTTGGATCGCC   128
rrnS  GACAAGGGGAGGCAAGTCGTAACATGGTAAGTGTACCGGAAGGTGCACTTGGATTAAACC--------------------------------------------------------------------   60
trnI  --------------------------------------------------------------------------------------------------------------------------------   0
                             rrnS

R     CTGAGCCAAACAGCTAGCTTAACCACCAATATAACCTAACAATGTTAATAACAAAACATGACTTAACACCACAAACTAAACCATTTTTTTACCTGAGTATGGGAGACAGAAAAGGTTCAACCCAAAGC   256
rrnS  --------------------------------------------------------------------------------------------------------------------------------   60
trnI  --------------------------------------------------------------------------------------------------------------------------------   0

R     AATAGAAAAAGTACCGCAAGGGAAAGCTGAAAGAGAAATGAAACAACCCATATAAGCACTAAAAAACAAAGACTAAACCTTGTACCTTTTGCATCATGATTTAGCCAGCACCCTCAAGCAAAGAGACC   384
rrnS  --------------------------------------------------------------------------------------------------------------------------------   60
trnI  --------------------------------------------------------------------------------------------------------------------------------   0

R     TTTAGTTTGAAACCCCGAAACCAGGTGAGCTACCCCGAGACAGCCTATTTAAATTTAGGGCTAACCCGTCTCTGTGGCAAAAGAGTGGGAAGAGCTCCGGGTAGAAGTGATAGACCTACCGAACCTGG   512
rrnS  --------------------------------------------------------------------------------------------------------------------------------   60
trnI  --------------------------------------------------------------------------------------------------------------------------------   0

R     TGATAGCTGGTTGCCTGAGAAGTGGATAGAAGTTCAGCCTCGTACGCCCCAAATCAAAAAATATAACATTAAGACATAAGGGATACATACGAGAGTTAGTTAAAGGGGGTACAGCCCCTCTAACAAAG   640
rrnS  --------------------------------------------------------------------------------------------------------------------------------   60
trnI  --------------------------------------------------------------------------------------------------------------------------------   0
```

```
R     GATACAACCTTCACAGGAGGATAAAGATCATAATATATAAAACATGCTGTTTTAGTGGGCCTAAAAGCAGCCATCTAAATAGAAAGCGTTAAAGCTCAGACAGAAAGAAGTTTATTATACTGATAAAA   768
rrnS  --------------------------------------------------------------------------------------------------------------------------------    60
trnI  --------------------------------------------------------------------------------------------------------------------------------     0

R     AATCTTATTCCCCTAACAATATCAGGCTAACCCATGCCCACATGGAAGAAATTATGCTAAAATGAGTAACAAGAAGACCTGCTCTTCTCCAAGCACAAGTGTAAGCCAGATCGGACAGACCACTGGAA   896
rrnS  --------------------------------------------------------------------------------------------------------------------------------    60
trnI  --------------------------------------------------------------------------------------------------------------------------------     0

R     ATTAACGAACCCAACCCAAGAGAGTAATGTGAATAATAGAAAAACCAAGAAAAACCCACAACCAAACAATCGTTAACCCCACACTGGAGTGCTATTTTTAAAGGAAAGACTAAAAGAAAGGGAAGGAA  1024
rrnS  --------------------------------------------------------------------------------------------------------------------------------    60
trnI  --------------------------------------------------------------------------------------------------------------------------------     0

R     CTCGGCAAACACAAGCCTCGCCTGTTTACCAAAAACATCGCCTCCTGCAACTAAACTGAGTATAGGAGGTCCAGCCTGCCCAGTGACTACGGGTTCAACGGCCGCGGTATTTTGACCGTGCAAAGGTA  1152
rrnS  --------------------------------------------------------------------------------------------------------------------------------    60
trnI  --------------------------------------------------------------------------------------------------------------------------------     0

R     GCGCAATCACTTGTCTTTTAAATAGAGACCTGTATGAATGGCTAAACGAGGGCTTAACTGTCTCCCCCTTCAAGTCAGTGAAATTGATCTATCCGTGCAGAAGCGGGTATAACCATACAAGACGAGAA  1280
rrnS  --------------------------------------------------------------------------------------------------------------------------------    60
trnI  --------------------------------------------------------------------------------------------------------------------------------     0

R     GACCCTTTGGAGCTTAAGGTACAAAATTCAACCACGTTAAGCAACTTAATAAAAAGCAAAAACTTAGTGGAAAATGAAATTTTACCTTCGGTTGGGGCGACCACGGAGGAAAAACAAGCCTCCGAGTG  1408
rrnS  --------------------------------------------------------------------------------------------------------------------------------    60
trnI  --------------------------------------------------------------------------------------------------------------------------------     0

R     GAATGGGCCAAACCCCTAAAACCAAGAGAAACATCTCTAAGCCACAGAACATCTGACCAAAAATGATCCGGCTAATAAAGCCGATCAACGAACCAAGTTACCCTAGGGATAACAGCGCAATCCTCTCC  1536
rrnS  --------------------------------------------------------------------------------------------------------------------------------    60
trnI  --------------------------------------------------------------------------------------------------------------------------------     0

R     CAGAGTCCATATCGACGAGGGGGTTTACGACCTCGATGTTGGATCAGGACATCCTAATGGTGCAGCCGCTATTAAGGGTTCGTTTGTTCAACGATTAAAGTCCTACGTGATCTGAGTTCAGACCGGAG  1664
rrnS  --------------------------------------------------------------------------------------------------------------------------------    60
trnI  --------------------------------------------------------------------------------------------------------------------------------     0
```

```
R     CAATCCAGGTCAGTTTCTATCTGTAACGCTACTTTTCCTAGTACGAAAGGATCGGAAAAGAGGGGCCCATACTTAAAGCACGCCCCACCCCTAATTAATGAAAACAAATAAATTAAACAAAGGGAGGG  1792
rrnS  --------------------------------------------------------------------------------------------------------------------------------    60
trnI  --------------------------------------------------------------------------------------------------------------------------------     0

R     CCAAAACCCCAACCGCCCGAAATAAGGACATACTGGGGTGGCAGAGCATGGTAAATTGCGAAAGGCCTAAGCCCTTTAAACCAGAGGTTCAAATCCTCTTCCCAGTTTATGCTAAACACTCTAATAAA  1920
rrnS  --------------------------------------------------------------------------------------------------------------------------------    60
trnI  --------------------------------------------------------------------------------------------------------------------------------     0

R     CCACCTGATCAACCCCCTAGCCTACATCGTGCCTGTCCTGCTAGCAGTAGCCTTCCTAACTCTAATCGAACGCAAAGTATTAGGATACATACAACTACGAAAAGGGCCAAATGTGGTCGGACCATACG  2048
rrnS  --------------------------------------------------------------------------------------------------------------------------------    60
trnI  --------------------------------------------------------------------------------------------------------------------------------     0

R     GTCTCCTACAACCTATCGCCGACGGAGTAAAATTATTTATTAAAGAGCCAGTCCGCCCTTCTACATCCTCCCCATTCCTATTCTTAGCCACCCCCATTCTTGCATTAACTCTAGCCCTAACTCTATGA  2176
rrnS  --------------------------------------------------------------------------------------------------------------------------------    60
trnI  --------------------------------------------------------------------------------------------------------------------------------     0

R     GCACCCATACCCATACCATACCCAGTAATTGATCTCAACCTAGGAGTTCTCTTTATCCTAGCCCTATCAAGCCTCGCAGTATATTCTATTCTAGGATCAGGATGAGCATCAAATTCAAAATACGCACT  2304
rrnS  --------------------------------------------------------------------------------------------------------------------------------    60
trnI  --------------------------------------------------------------------------------------------------------------------------------     0

R     AATCGGAGCCCTACGGGCCGTAGCCCAAACAATTTCCTATGAAGTAAGCCTCGGACTAATCCTTCTATCAGTAATTATCTTCTCAGGAGGCTACACCCTACAAACATTTAGTACGGCCCAAGAAAGTA  2432
rrnS  --------------------------------------------------------------------------------------------------------------------------------    60
trnI  --------------------------------------------------------------------------------------------------------------------------------     0

R     TCTGACTATTAGCCCCTGCATGACCATTAGCCGCAATATGATACATCTCAACCCTGGCAGAAACTAACCGAGCACCATTCGACCTAACAGAAGGAGAATCAGAGCTAGTCTCAGGCTTCAACGTAGAA  2560
rrnS  --------------------------------------------------------------------------------------------------------------------------------    60
trnI  --------------------------------------------------------------------------------------------------------------------------------     0

R     TATGCAGGAGGGCCCTTCGCCCTCTTCTTCCTAGCCGAGTATGCAAACATCCTATTAATAAATACCTTATCAGCTGTACTATTTCTGGGAACATCACACATCCACCACATCCCAGAATTAACAACAAT  2688
rrnS  --------------------------------------------------------------------------------------------------------------------------------    60
trnI  --------------------------------------------------------------------------------------------------------------------------------     0
```

```
R     TAGCCTTATGACTAAGGCCGCGCTACTCTCTATTGTATTCCTCTGAGTACGAGCCTCGTATCCACGATTCCGATACGACCAACTAATACACCTTGTATGAAAAAACTTCCTCCCCCTAACATTGGCTC  2816
rrnS  --------------------------------------------------------------------------------------------------------------------------------    60
trnI  --------------------------------------------------------------------------------------------------------------------------------     0
```

```
                                                                                          trnI
R     TAGTACTATGACACATTGCCCTGCCAATCGCACTAGCGGGCCTTCCCCCACAACTATAGTTCAGGAACTGTGCCCGAATGCCTAGGGACCACTTTGATAGAGTGGCCTATAGGGGCTAAAATCCCCTC  2944
rrnS  --------------------------------------------------------------------------------------------------------------------------------    60
trnI  ------------------------------------------------------------TCAGGAACTGTGCCCGAATGCCTAGGGACCACTTTGATAGAGTGGCCTATAGGGGCTAAAAT-----C    63
                                                                                   trnI
```

```
       trnI
R     AGTTCTT  2951
rrnS  -------    60
trnI  -------    63
```

1.117 NC_016059-NC_015305

Avg ovsize: -987

LCA: Ranidae-family

1.117.1 nad4-trnH 11

```
                                  nad4                                                     trnH
R     TTCTCCTCCTCCATATCTTACCAGGCCTCCTCCTTGTACTCAAACCAGAATTAATTTTCTGTGAATATAGTTTAACAAAAACCCTAGATTGTGATTCTAGAAACAAAGGTTGAACCCCT-  119
nad4  --CT--TCAT--AT-TC-TACCCGGACTTCTATTTATCCTCAAGCCAGAGCTACTTTTCTGTGTATA-----------------------------------------------------   59
trnH  --------------------------------------------------------TTATGAGTGTA-A-TTAAACAATAATGCTAGATCGTGACTCTAAAATTGAAAGTTAAAATCCTT   62
                                      nad4
                                                                                         trnH
```

1.117.2 trnH-trnS1 2

```
                            trnH                                                         trnS1
R      GTTTAACAAAAACCCTAGATTGTGATTCTAGAAACAAAGGTTGAACCCCTTTTATTCACCGAGCCCGACTGGAGTAATGAGAACTGCTAATTCCTCACCCCCATGGTTCAATTCCATG---   118
trnH   ATTAAACAATAATGCTAGATCGTGACTCTAAAATTGAAAGTTAAAATCCTTCTGCTC----------------------------------------------------------------    57
trnS1  -------------------------------------------------------TCCCCGAGCCTGCCCGGCATAGTGAGAACTGCTAATTCCTCACCAC-ATGGTTCGACTCCATGCTC    65
                           trnH                                                           trnS1
```

1.117.3 cob-trnL1 -2974

```
                              cob
R       --A-TCTTTGTCCTTCTTATCCCATCACTAGGACTCCTGGAAAACAAGCTCCTAAAAATTTAAAACAACTGCCTCCGCCTTATGTATATAGAGCATAAATTTATTACCCCATATTAAGGCTAACATAC   125
cob     CTAGTATTTATCCT-CGTA-CCCATG-CTAGGCCTCCTAGAAAATAAGCTTCTTAAAATCTA------------------------------------------------------------------    59
trnL1   --------------------------------------------------------------------------------------------------------------------------------     0
                             cob

R       CTATTTCATCCACATTAATGTATGATTTATAACATTCATGTATAATAACCATACATTAAACTTATATACATATTAAGATGTACATATATGCACCACAAATATTATATTAATGTATTAAGACATTTCAT   253
cob     --------------------------------------------------------------------------------------------------------------------------------    59
trnL1   --------------------------------------------------------------------------------------------------------------------------------     0

R       GTATGTTCTAAAACATTATATGTATATAGTACATTAATCTATTTACCCCAAGCATATCATAAACAATGTAAAAGAATGGTCTGATACCTTAATAACCTGACCAAAAATGTTTAACCCCATCTTATGCT   381
cob     --------------------------------------------------------------------------------------------------------------------------------    59
trnL1   --------------------------------------------------------------------------------------------------------------------------------     0

R       TCGTAACCGTCCATATCATGACTACTTGATCGCACCTTCCCTTGTTAACGTCTACGCAGATCATAAACCAAAGTGAGTCCAACCGGTTGATCCCCAAAAACAGAAACCCAATGTTCCTGTATACCTTG   509
cob     --------------------------------------------------------------------------------------------------------------------------------    59
trnL1   --------------------------------------------------------------------------------------------------------------------------------     0
```

```
R       AACTTAACAAATGAATACTTTTACAACTTTAGAGTTACATCCCACATATACCACCCAATTGAATGCTTCATTCATTTAATCAAGGTAAGACCTTAACTTGCACAAAATCACGCAGATTATTATAACAA   637
cob     --------------------------------------------------------------------------------------------------------------------------------    59
trnL1   --------------------------------------------------------------------------------------------------------------------------------     0

R       TCTTTCAATAATACCCTACCATGGACCTGCATACAGTATACTAAACGATAAATTGCAAATAAAGTCCTTACAACATTAACCTGCCAGACCTCTACCTTGAACAATCAACACGACGCGTACGCTTTCAC   765
cob     --------------------------------------------------------------------------------------------------------------------------------    59
trnL1   --------------------------------------------------------------------------------------------------------------------------------     0

R       CCTATCGTACCCCCGTCAACCAGCCATGGATCGGGCCTAGGGTGATCTTAATTTTCCAATGGACCTTAATTGTAGAGTTACAGCGATTGACCTTTAGGAGGTATCTGACGGAACCGAATCTATGGACC   893
cob     --------------------------------------------------------------------------------------------------------------------------------    59
trnL1   --------------------------------------------------------------------------------------------------------------------------------     0

R       CAAATAGAAGAGACGCTCAAAATGCTTTTTAAAAGGTATCCCTCCTATGAACGAAAGACCTCTCTACAAGCTCAGACCACTTAATGACAGCTAAGTCTACTGGTATTTTTTTTTGGGGGTCCTTTCAC  1021
cob     --------------------------------------------------------------------------------------------------------------------------------    59
trnL1   --------------------------------------------------------------------------------------------------------------------------------     0

R       CAGCAACTCCAGTGGGGTCACGGCTTACGGATAAGGTTGGGACATATAGTCCAGGTACCATAATTTTACATTGTCTCTTGTATGATGCATTTTAATGAATGCTATAATGACATAATGTCAGCCCATAA  1149
cob     --------------------------------------------------------------------------------------------------------------------------------    59
trnL1   --------------------------------------------------------------------------------------------------------------------------------     0

R       TTACTGATTCTTCCCCCTTTCCGCAATAAAAATTTTTACGGCTTTTTGTCGCAGAACCCCCCCCTTTCCCCCCCCACAGCTTATTTTCCTGTAGATTGCCTTAAAACCCCCCCCGAGATTAAGGTTTA  1277
cob     --------------------------------------------------------------------------------------------------------------------------------    59
trnL1   --------------------------------------------------------------------------------------------------------------------------------     0

R       TAGGAGTTCTGCCATGGGATTGTGACCGTCAACTTTTTTTTTTCCTATAGATTGCCTTAAAACCCCCCCGAGATTAAGGTTTATAGGAGTTCTGCCATGGGATTGTGACCGTCAACTTTTTTTTTTCC  1405
cob     --------------------------------------------------------------------------------------------------------------------------------    59
trnL1   --------------------------------------------------------------------------------------------------------------------------------     0

R       TATAGATTGCCTTAAAACCCCCCCCGAGATTAAGGTTTATAGGAGTTCTGCCATGGGATTGTGACCGTCAACTTTTTTTTTTCCTATAGATTGCCTTAAAACCCCCCCCGAGATTAAGGTTTATAGGA  1533
cob     --------------------------------------------------------------------------------------------------------------------------------    59
trnL1   --------------------------------------------------------------------------------------------------------------------------------     0
```

```
R       GTTCTGCCATGGGATTGTGACCGTCAACTTTTTTTTTCCTATAGATTGCCTTAAAACCCCCCCCGAGATTAAGGTTTATAGGAGTTCTGCTGTGGGCCACACCACTGTCCTGCCCTGCCCCACCTAGG  1661
cob     --------------------------------------------------------------------------------------------------------------------------------    59
trnL1   --------------------------------------------------------------------------------------------------------------------------------     0

R       GCTTACTTAACTATATAAAAACTATCGAGTCCTTATAGGTACTTATCCTTATAGGTACTTATCCTTATAGGTACTTATCCTTATAGGTACTTATCCTTATAGGTACTTATCCTTATAGGTACTTATCC  1789
cob     --------------------------------------------------------------------------------------------------------------------------------    59
trnL1   --------------------------------------------------------------------------------------------------------------------------------     0

R       TTATAGGTACTTATCCTTGTAGGTACTTATCCTTGTAGGTACTTATCCTTATAGGTACTTATCCTTATAGGTACTTATCCTTGTAGGTACTTATCCTTGTAGGTACTTATCCTTGTAGGTACTTATCC  1917
cob     --------------------------------------------------------------------------------------------------------------------------------    59
trnL1   --------------------------------------------------------------------------------------------------------------------------------     0

R       TTGTAGGTACTTATCCTTGTAGGTACTTATCCTTGTAGGTTACTTATCCTTGTAGGTTACTTATCCTTGTAGGTTACTTATCCTTGTAGGTTACTTATCCTTGTAGGTTACTTATCCTTGTAGGTTAC  2045
cob     --------------------------------------------------------------------------------------------------------------------------------    59
trnL1   --------------------------------------------------------------------------------------------------------------------------------     0

R       TTATCCTTGTAGGTTACTTATCCTTGTAGGTTACTTATCCTTGTAGGTTACTTATCCTTGTAGGTTACTTATCCTTGTAGGTTACTTATCCTTGTAGGTTACTTATCCTTGTAGGTTACTTATCCTTG  2173
cob     --------------------------------------------------------------------------------------------------------------------------------    59
trnL1   --------------------------------------------------------------------------------------------------------------------------------     0

R       TAGGTTACTTATCCTTGTGGATTACTTATCCTTGTGGATTACTTATCCTTGTGGATTACTTATCCTTGTGGATTACTTATCCTTGTGGATTACTTATCCTTGTGGATTACTTATCCTTGTGGATTACT  2301
cob     --------------------------------------------------------------------------------------------------------------------------------    59
trnL1   --------------------------------------------------------------------------------------------------------------------------------     0

R       TATCCTTGTGGATTACTTATCCTTGTGGATTACTTATCCTTGTGGATTACTTATCCTTGTGGATTACTTATCCTTGTGGATTACTTATCCTTGTGGATTACTTATCCTTGTGGATTACTTATCCTTGT  2429
cob     --------------------------------------------------------------------------------------------------------------------------------    59
trnL1   --------------------------------------------------------------------------------------------------------------------------------     0

R       GGATTACTTATCCTTGTGGATTACTTATCCTTGTGGATTACTTATCCTTGTGGATTACTTATCCTTGTGGATTACTTATCCTTGTGGATTACTTATCCTTGTGGATTACTTATCCTTGTGGATTACTT  2557
cob     --------------------------------------------------------------------------------------------------------------------------------    59
trnL1   --------------------------------------------------------------------------------------------------------------------------------     0
```

```
R      ATCCTTGTGGATTACTTATCCTTGTGGATTACTTATCCTTGTGGATTACTTATCCTTGTGGGTTACTTATCCTTGTGGGTTACTTATCCTTGTGGGTTACTTATCCTTGTGGGTTACTTATCCTTGTG  2685
cob    --------------------------------------------------------------------------------------------------------------------------------  59
trnL1  --------------------------------------------------------------------------------------------------------------------------------  0

R      GGTTACTTATCCTTGTGGGTTACTTATCCTTGTGGGTTACTTATCCTTGTGGATTACTTATCCTTGTGGGTTACCACGTATTTATTAAACCTATAACATCCCTGTATTCCCACATGAACTGCCACGAG  2813
cob    --------------------------------------------------------------------------------------------------------------------------------  59
trnL1  --------------------------------------------------------------------------------------------------------------------------------  0

R      TTTCGCGAACCATTCTCTGATACTGTCATCTGCCTTTGTTATTTATCTACTAATATCCTTCCTATTCCCCAAACTACCCGCCCCAACCTCCAAACAACCTTAACTCACCTAAATTTTCTGTTTGTATG  2941
cob    --------------------------------------------------------------------------------------------------------------------------------  59
trnL1  --------------------------------------------------------------------------------------------------------------------------------  0

                                                                                                                   trnL1
R      AGATTGCCTGAATAATATAAATTTAGCCCACCCTTACAACTTTATACCTACCATAAGATTAGACCACTTAATTTTAATAACCCTCCCCACCCATCCGCTTTTAAAGGAAAAGAGCCCTCCACTGGCCT  3069
cob    --------------------------------------------------------------------------------------------------------------------------------  59
trnL1  --------------------------------------------------------------------------------------------ACCCGCTTTTAAAGGAAAACAGCCCTCCACTGGCCT  36
                                                                                                                trnL1

                  trnL1
R      TAGGAGCCAGCATCTCTTGGTGCAAGT-  3096
cob    ----------------------------  59
trnL1  TAGGAGCCAGCACCTCTTGGTGCAAGTC  64
                  trnL1
```

1.118 NC_016059-NC_008975

Avg ovsize: -1045

LCA: Ranoidea-superfamily

1.118.1 nad5-nad6 -44

```
                                       nad5                                                              nad6
R     G-CCTGATTAAGACCTACCTCTCCGTTTTTTTTGTCACACTTGTCTTTACCCTCACAATCTTACAGCTCGTAAGGTCCCCCTCACTTGTATCCACGCACCACCTCTAAGGCAAC--A-A-   116
nad5  TACCTG-TCAACA-----------------------------------------------------------------------------------------------------------   12
nad6  ---------------------------------------------------------ACCTCACAGCCCGCAAAGTTCCAG--C-CTTGTGCCCACGAACCACCTCTAATACAACAAATAA   61
          nad5
                                                                                             nad6
```

1.118.2 trnS1-nad5 -65

```
                          trnS1                                                                        nad5
R       CTGGAG-TAATGAGAACTGCTAATTCCTCACCCCCATGGTTCAATTCCATGGCCCGCTCGTACTTATACTCCATTAAACCCTAGTATAAGTTATGTATTCACTAAATGCAATAGCCTTCGCCGCCATA   127
trnS1   ATTGGGATAATGAGTACTGCTAATTCCTCACCACCATGGTTCGATTCCACGGCTAGCTCGAA------------------------------------------------------------------   62
nad5    -------------------------------------------------------------------------------------------------------------------------------A   1
                          trnS1
                                                                                                                                      nad5
```

```
              nad5
R       ATTACCTCCATTCTTCTTATTGT   150
trnS1   -----------------------   62
nad5    ACTACCTTGATCCTTCCCCTA--   22
             nad5
```

1.118.3 cob-trnL1 -3028

cob

```
R       ATCTTTGTCCTTCTTATCCCATCACTAGGACTCCTGGAAAACAAGCTCCTAAAAATTTAAAACAACTGCCTCCGCCTTATGTATATAGAGCATAAATTTATTACCCCATATTAAGGCTAACATACCTA   128
cob     --------------------------------------------------------------------------------------------------------------------------------     0
trnL1   --------------------------------------------------------------------------------------------------------------------------------     0

R       TTTCATCCACATTAATGTATGATTTATAACATTCATGTATAATAACCATACATTAAACTTATATACATATTAAGATGTACATATATGCACCACAAATATTATATTAATGTATTAAGACATTTCATGTA   256
cob     --------------------------------------------------------------------------------------------------------------------------------     0
trnL1   --------------------------------------------------------------------------------------------------------------------------------     0

R       TGTTCTAAAACATTATATGTATATAGTACATTAATCTATTTACCCCAAGCATATCATAAACAATGTAAAAGAATGGTCTGATACCTTAATAACCTGACCAAAAATGTTTAACCCCATCTTATGCTTCG   384
cob     --------------------------------------------------------------------------------------------------------------------------------     0
trnL1   --------------------------------------------------------------------------------------------------------------------------------     0

R       TAACCGTCCATATCATGACTACTTGATCGCACCTTCCCTTGTTAACGTCTACGCAGATCATAAACCAAAGTGAGTCCAACCGGTTGATCCCCAAAAACAGAAACCCAATGTTCCTGTATACCTTGAAC   512
cob     --------------------------------------------------------------------------------------------------------------------------------     0
trnL1   --------------------------------------------------------------------------------------------------------------------------------     0

R       TTAACAAATGAATACTTTTACAACTTTAGAGTTACATCCCACATATACCACCCAATTGAATGCTTCATTCATTTAATCAAGGTAAGACCTTAACTTGCACAAAATCACGCAGATTATTATAACAATCT   640
cob     --------------------------------------------------------------------------------------------------------------------------------     0
trnL1   --------------------------------------------------------------------------------------------------------------------------------     0

R       TTCAATAATACCCTACCATGGACCTGCATACAGTATACTAAACGATAAATTGCAAATAAAGTCCTTACAACATTAACCTGCCAGACCTCTACCTTGAACAATCAACACGACGCGTACGCTTTCACCCT   768
cob     --------------------------------------------------------------------------------------------------------------------------------     0
trnL1   --------------------------------------------------------------------------------------------------------------------------------     0

R       ATCGTACCCCCGTCAACCAGCCATGGATCGGGCCTAGGGTGATCTTAATTTTCCAATGGACCTTAATTGTAGAGTTACAGCGATTGACCTTTAGGAGGTATCTGACGGAACCGAATCTATGGACCCAA   896
cob     --------------------------------------------------------------------------------------------------------------------------------     0
trnL1   --------------------------------------------------------------------------------------------------------------------------------     0
```

```
R      ATAGAAGAGACGCTCAAAATGCTTTTTAAAAGGTATCCCTCCTATGAACGAAAGACCTCTCTACAAGCTCAGACCACTTAATGACAGCTAAGTCTACTGGTATTTTTTTTTGGGGGTCCTTTCACCAG  1024
cob    --------------------------------------------------------------------------------------------------------------------------------     0
trnL1  --------------------------------------------------------------------------------------------------------------------------------     0

R      CAACTCCAGTGGGGTCACGGCTTACGGATAAGGTTGGGACATATAGTCCAGGTACCATAATTTTACATTGTCTCTTGTATGATGCATTTTAATGAATGCTATAATGACATAATGTCAGCCCATAATTA  1152
cob    --------------------------------------------------------------------------------------------------------------------------------     0
trnL1  --------------------------------------------------------------------------------------------------------------------------------     0

R      CTGATTCTTCCCCCTTTCCGCAATAAAAATTTTTACGGCTTTTTGTCGCAGAACCCCCCCCTTTCCCCCCCCACAGCTTATTTTCCTGTAGATTGCCTTAAAACCCCCCCCGAGATTAAGGTTTATAG  1280
cob    --------------------------------------------------------------------------------------------------------------------------------     0
trnL1  --------------------------------------------------------------------------------------------------------------------------------     0

R      GAGTTCTGCCATGGGATTGTGACCGTCAACTTTTTTTTTTCCTATAGATTGCCTTAAAACCCCCCCGAGATTAAGGTTTATAGGAGTTCTGCCATGGGATTGTGACCGTCAACTTTTTTTTTTCCTAT  1408
cob    --------------------------------------------------------------------------------------------------------------------------------     0
trnL1  --------------------------------------------------------------------------------------------------------------------------------     0

R      AGATTGCCTTAAAACCCCCCCCGAGATTAAGGTTTATAGGAGTTCTGCCATGGGATTGTGACCGTCAACTTTTTTTTTTCCTATAGATTGCCTTAAAACCCCCCCCGAGATTAAGGTTTATAGGAGTT  1536
cob    --------------------------------------------------------------------------------------------------------------------------------     0
trnL1  --------------------------------------------------------------------------------------------------------------------------------     0

R      CTGCCATGGGATTGTGACCGTCAACTTTTTTTTTTCCTATAGATTGCCTTAAAACCCCCCCGAGATTAAGGTTTATAGGAGTTCTGCTGTGGGCCACACCACTGTCCTGCCCTGCCCCACCTAGGGCT  1664
cob    --------------------------------------------------------------------------------------------------------------------------------     0
trnL1  --------------------------------------------------------------------------------------------------------------------------------     0

R      TACTTAACTATATAAAAACTATCGAGTCCTTATAGGTACTTATCCTTATAGGTACTTATCCTTATAGGTACTTATCCTTATAGGTACTTATCCTTATAGGTACTTATCCTTATAGGTACTTATCCTTA  1792
cob    --------------------------------------------------------------------------------------------------------------------------------     0
trnL1  --------------------------------------------------------------------------------------------------------------------------------     0

R      TAGGTACTTATCCTTGTAGGTACTTATCCTTGTAGGTACTTATCCTTATAGGTACTTATCCTTATAGGTACTTATCCTTGTAGGTACTTATCCTTGTAGGTACTTATCCTTGTAGGTACTTATCCTTG  1920
cob    --------------------------------------------------------------------------------------------------------------------------------     0
trnL1  --------------------------------------------------------------------------------------------------------------------------------     0
```

```
R       TAGGTACTTATCCTTGTAGGTACTTATCCTTGTAGGTTACTTATCCTTGTAGGTTACTTATCCTTGTAGGTTACTTATCCTTGTAGGTTACTTATCCTTGTAGGTTACTTATCCTTGTAGGTTACTTA  2048
cob     --------------------------------------------------------------------------------------------------------------------------------     0
trnL1   --------------------------------------------------------------------------------------------------------------------------------     0

R       TCCTTGTAGGTTACTTATCCTTGTAGGTTACTTATCCTTGTAGGTTACTTATCCTTGTAGGTTACTTATCCTTGTAGGTTACTTATCCTTGTAGGTTACTTATCCTTGTAGGTTACTTATCCTTGTAG  2176
cob     --------------------------------------------------------------------------------------------------------------------------------     0
trnL1   --------------------------------------------------------------------------------------------------------------------------------     0

R       GTTACTTATCCTTGTGGATTACTTATCCTTGTGGATTACTTATCCTTGTGGATTACTTATCCTTGTGGATTACTTATCCTTGTGGATTACTTATCCTTGTGGATTACTTATCCTTGTGGATTACTTAT  2304
cob     --------------------------------------------------------------------------------------------------------------------------------     0
trnL1   --------------------------------------------------------------------------------------------------------------------------------     0

R       CCTTGTGGATTACTTATCCTTGTGGATTACTTATCCTTGTGGATTACTTATCCTTGTGGATTACTTATCCTTGTGGATTACTTATCCTTGTGGATTACTTATCCTTGTGGATTACTTATCCTTGTGGA  2432
cob     --------------------------------------------------------------------------------------------------------------------------------     0
trnL1   --------------------------------------------------------------------------------------------------------------------------------     0

R       TTACTTATCCTTGTGGATTACTTATCCTTGTGGATTACTTATCCTTGTGGATTACTTATCCTTGTGGATTACTTATCCTTGTGGATTACTTATCCTTGTGGATTACTTATCCTTGTGGATTACTTATC  2560
cob     --------------------------------------------------------------------------------------------------------------------------------     0
trnL1   --------------------------------------------------------------------------------------------------------------------------------     0

R       CTTGTGGATTACTTATCCTTGTGGATTACTTATCCTTGTGGATTACTTATCCTTGTGGGTTACTTATCCTTGTGGGTTACTTATCCTTGTGGGTTACTTATCCTTGTGGGTTACTTATCCTTGTGGGT  2688
cob     --------------------------------------------------------------------------------------------------------------------------------     0
trnL1   --------------------------------------------------------------------------------------------------------------------------------     0

R       TACTTATCCTTGTGGGTTACTTATCCTTGTGGGTTACTTATCCTTGTGGATTACTTATCCTTGTGGGTTACCACGTATTTATTAAACCTATAACATCCCTGTATTCCCACATGAACTGCCACGAGTTT  2816
cob     --------------------------------------------------------------------------------------------------------------------------------     0
trnL1   --------------------------------------------------------------------------------------------------------------------------------     0

R       CGCGAACCATTCTCTGATACTGTCATCTGCCTTTGTTATTTATCTACTAATATCCTTCCTATTCCCCAAACTACCCGCCCCAACCTCCAAACAACCTTAACTCACCTAAATTTTCTGTTTGTATGAGA  2944
cob     --------------------------------------------------------------------------------------------------------------------------------     0
trnL1   --------------------------------------------------------------------------------------------------------------------------------     0
```

```
                                                                                            trnL1
R      TTGCCTGAATAATATAAATTTAGCCCACCCTTACAACTTTATACCTACCATAAGATTAGACCACTTAATTTTAATAACCCTCCCCACCC-AT-CCGCTTTTAAAGGAAAAGAGCCCTCCACTGGCCTT 3070
cob    --------------------------------------------------------------------------------------------------------------------------------    0
trnL1  ------------------------------------------------------------------------------------CACCTAATGCAGCTTTTAAAGGAAAAAAGTCTTCCTCTGGCCTT   44
                                                                                            trnL1
```

```
               trnL1
R      AGGAGCCAGCATCTCTTGGTGCAAGT- 3096
cob    ---------------------------    0
trnL1  AGGAGCCAGCATCTCTTGGTGCAAATC   71
               trnL1
```

1.119 NC_006335-NC_004592

Avg ovsize: -1121

LCA: Teleostomi-superclass

1.119.1 nad5-trnT -80

```
                             nad5                                                                                   trnT
R      ---TAG-TAACACTTACCT--CTACCCTTTTAATGGCCTCCCCAGTCCTAATTAATTATATCAACTTACCCAAATCTATAATTGATAGAAATTTACACCAAATCAATACTACC-GCAGTTTA-AGAAA 120
nad5   ACATACTTAACCCTTTTTTTCCTAACCTTT--------------------------------------------------------------------------------------------------  30
trnT   --------------------------------------------------------------------------------------------------------------ACCAGAAGCTCAGAGAAG  18
                 nad5                                                                                                  trnT
```

```
                                 trnT
R       A-CATCAGCCTTGTAAGCTGAAGAATGGGGATTTAAAATCT   160
nad5    -----------------------------------------    30
trnT    AGCATCGGTTTTGTAATCCGAA-AGTCGAAGGTTAAAATCC    58
                                 trnT
```

1.119.2 trnT-nad6 -1166

```
                                          trnT
R       GTTTAAGAAAA-CATCAGCCTTGTAAGCTGAAGAATGGGGATTTAAAATC--TCCCGATAGTCCGGGCTCCGCCTAAGGACGTAGACTTAAACCCAGGCAGAGCCTGGGAAAGAATCTAATTTAAATA   125
trnT    -TCAGAGAAGAGCATCGGTTTTGTAATCCGAA-AGTCGAAGGTTAAAATCCATCCTGATGCTAAGAGC-CC-CCT------------------------------------------------------    71
nad6    --------------------------------------------------------------------------------------------------------------------------------     0
                                          trnT

R       TATATTCCACATGCGAGTAAAAAGGGGGGGGGTCCTGGCTCTATCCAGGCCCTGCCTTGAGGCATTATTACCAATCCAGGCTCTACCACCATATAACCCAACCCCACATACCCATATAAACCAAAAGT   253
trnT    --------------------------------------------------------------------------------------------------------------------------------    71
nad6    --------------------------------------------------------------------------------------------------------------------------------     0

R       CACCTAAAAACAACTACCGCTTATGTCCTTTCTTCACTATAGTAACGCGGTGACATATTATGTATAATAGTGCATTCATCTAATTTCCATACGAATCTGTTTTAGTACCCTCCTGGTTTTTAATTTCA   381
trnT    --------------------------------------------------------------------------------------------------------------------------------    71
nad6    --------------------------------------------------------------------------------------------------------------------------------     0

R       CACACGGTAGAGAAATAAACAACCCGCCCCTCCAGACACGATGTCCAGATCTAAGGACCTATATTGTAGGTCCGCAGCTCGCTATCTTCACGAGACCACTGGTTATGAATCTATGTGCACATAGGCAA   509
trnT    --------------------------------------------------------------------------------------------------------------------------------    71
nad6    --------------------------------------------------------------------------------------------------------------------------------     0

R       GAGTAGGCGGTAACAGTACGACCCCCATATAGTTGAAGGTGATGCTCTCAAGAATCGGCGTACCCCGCACCCACATAACTGGTCTAGATGCATTCATTATTTTTTTTTCTCTTGTGAGGTCAGCCAAC   637
trnT    --------------------------------------------------------------------------------------------------------------------------------    71
nad6    --------------------------------------------------------------------------------------------------------------------------------     0
```

```
                                                                                                                                                                                 nad6
R     ACCCAATTTATAACTGGTAAATCTGGCTCTAAAACCTGAACATATAATGCCCGTGTAGTCTTACTATATACGCATGTTTTAATATAAGTCAATGTTGTATGGACATACACCTTCGTATTCCTCTGCCT  765
trnT  --------------------------------------------------------------------------------------------------------------------------------  71
nad6  --------------------------------------------------------------------------------------------------------------------------------  0

R     GATTCCCAGAATATTACCTCCCAGTTTCCCCCCCCTCTTGCATTTCACAAAATAATTTTATTATACAATATATTTTTCTACTTTAAACCCCCCTCCCCCAAAATATAGGCTAATCGGCGCCTGAAATC  893
trnT  --------------------------------------------------------------------------------------------------------------------------------  71
nad6  --------------------------------------------------------------------------------------------------------------------------------  0

R     TTTAGTCAAACCCCGAAACCTAAAAACCCATCACACTCACGCACTGAAATAACAAATGATTAATAATAATTTTATTAACTTTTAATATATTTTAATATATTTTAATATATTTTAATATATTTTAATAT  1021
trnT  --------------------------------------------------------------------------------------------------------------------------------  71
nad6  --------------------------------------------------------------------------------------------------------------------------------  0

R     ATTATAAGATATATTAGTGTAACTTACTAAAGCACGGCACTGAAAATGCCAATATAAATAATAATAAATTTTATAAACACTAAAAGGTCTGGTCCCAGCCTTAGTATAAATTTTTAATATGAGAACTT  1149
trnT  --------------------------------------------------------------------------------------------------------------------------------  71
nad6  --------------------------------------------------------------------------------------------------------------------------------  0

                                                                                                                      nad6
R     ATATAATATATTTAAAATTTCTAACCAACAGCTGCCACCATAAACCAATACAACTTTTTAACCAAATTTACCAAATACATAAACCCCTCACAGCACGTAAAGATCCACGAGATACCCCACGAGTAATT  1277
trnT  --------------------------------------------------------------------------------------------------------------------------------  71
nad6  -----------------------------------------------------------------------------------------ACAGCCCGAACCGTCCCCCGACTCAACCCCCGTGTTAAC  39
                                                                                                                      nad6

        nad6
R     TCTAATACCGTAAATA-  1293
trnT  -----------------  71
nad6  TCTAATACTACAAAAAG  56
        nad6
```

1.119.3 trnP-trnF -2117

```
                                            trnP
R       AGGATTTACACCTTC-ACCATTGACACCCAAGGCCAAAATTCTAAATTTAACTACCGCTTATGTCCTTTCTTCACTATAGTAACGCGGTGACATATTATGTATAATAGTGCATTCATCTAATTTCCAT   127
trnP    GAGATTTTAA-CTCCTATCTTTAGCTCCCAAAGCTAAAATTTTAAATTAAACTACC-CT---------------------------------------------------------------------    57
trnF    --------------------------------------------------------------------------------------------------------------------------------     0
                                           trnP

R       ACGAATCTGTTTTAGTACCCTCCTGATTTTTAATTTCACACACGGTAGAGAAATAAACAACCCGCCCCTCCAGACACGATGTCCAGATCTAAGGACCTATATTGTAGGTCCGCAGCTCGCTATCTTCA   255
trnP    --------------------------------------------------------------------------------------------------------------------------------    57
trnF    --------------------------------------------------------------------------------------------------------------------------------     0

R       CGAGACCACTGGTTATGAATCTATGTGCACATAGGCAAGAGTAGGCGGTAACAGTACGACCCCCATATAGTTGAAGGTGATGCTCTCAAGAATCGGCGTACCCCGCACCCACATAACTGGTCTAGATG   383
trnP    --------------------------------------------------------------------------------------------------------------------------------    57
trnF    --------------------------------------------------------------------------------------------------------------------------------     0

R       CATTCATTATTTTTTTTTCTCTTGTGAGGTCAGCCAACACCCAATTTATAACTGGTAAATCTGGCTCTAAAACCTGAACATATAATGCCCGTGTAGTCTTACTATATACGCATGTTTTAATATAAGTC   511
trnP    --------------------------------------------------------------------------------------------------------------------------------    57
trnF    --------------------------------------------------------------------------------------------------------------------------------     0

R       AATGTTGTATGGACATACACCTTCGTATTCCTCTGCCTGATTCCCAGAATATTACCTCCCAGTTTCCCCCCCCTCTTGCATTTCACAAAATAATTTTATTATACAATATATTTTTCTACTTTAAACCC   639
trnP    --------------------------------------------------------------------------------------------------------------------------------    57
trnF    --------------------------------------------------------------------------------------------------------------------------------     0

R       CCCTCCCCCAAAATATAGGCTAATCGGCGCCTGAAATCTTTAGTCAAACCCCGAAACCTAAAAACCCATCACACTCACGCACTGAAATAACAAATGATTAATAATAATTTTATTAACTTTTAATATAT   767
trnP    --------------------------------------------------------------------------------------------------------------------------------    57
trnF    --------------------------------------------------------------------------------------------------------------------------------     0

R       TTTAATATATTTTAATATATTTTAATATATATTTTAATATATTGTAAGATATGCTAGTGTAGCTTACTAAAGCACGGCACTGAAAATGCCAATATAAATAATAATAAATTTTATAAACACTAAAAGGT   895
trnP    --------------------------------------------------------------------------------------------------------------------------------    57
trnF    --------------------------------------------------------------------------------------------------------------------------------     0
```

```
R     CTGGTCCCAGCCTTAGTATAAATTTTTAATATGAGAACTTATATAATATATTTAAAATTTCTAACCAACAGCTGCCACCAATTAAATCTAATATAGAACTTCAAACAGGATTAATTAAAACCTATATA  1023
trnP  --------------------------------------------------------------------------------------------------------------------------------    57
trnF  --------------------------------------------------------------------------------------------------------------------------------     0

R     CTAGTAACACTTACCTCTACCCTTTTAATGGCCTCCCCAGTCCTAATTAATTATATCAACTTACCCAAATCTATAATTGATAGAAATTTACACCAAATCAATACTACCGCAGTTTAAGAAAACATCAG  1151
trnP  --------------------------------------------------------------------------------------------------------------------------------    57
trnF  --------------------------------------------------------------------------------------------------------------------------------     0

R     CCTTGTAAGCTGAAGAATGGGGATTTAAAATCTCCCGATAGTCCGGGCTCCGCCTAAGGACGTAGACTTAAACCCAGGCAGAGCCTGGGAAAGAATCTAATTTAAATATATATTCCACATGCGAGTAA  1279
trnP  --------------------------------------------------------------------------------------------------------------------------------    57
trnF  --------------------------------------------------------------------------------------------------------------------------------     0

R     AGAGGGGGGGGTCCTGGCTCTATCCAGGCCCTGCCTTGAGGCATTATTACCAATCCAGGCTCTACCACCATATAACCCAACCCCACATACCCATATAAACCGAAAGTCACCTAAAAACAACTACCGC  1407
trnP  --------------------------------------------------------------------------------------------------------------------------------    57
trnF  --------------------------------------------------------------------------------------------------------------------------------     0

R     TTATGTCCTTTCTTCACTATAGTAACGCGGTGACATATTATGTATAATAGTGCATTCATCTAATTTCCATACGAATCTGTTTTAGTACCCTCCTGATTTTTAATTTCACACACGGTAGAGAAATAAAC  1535
trnP  --------------------------------------------------------------------------------------------------------------------------------    57
trnF  --------------------------------------------------------------------------------------------------------------------------------     0

R     AACCCGCCCCTCCAGACACGATGTCCAGATCTAAGGACCTATATTGTAGGTCCGCAGCTCGCTATCTTCACGAGACCACTGGTTATGAATCTATGTGCACATAGGCAAGAGTAGGCGGTAACAGTACG  1663
trnP  --------------------------------------------------------------------------------------------------------------------------------    57
trnF  --------------------------------------------------------------------------------------------------------------------------------     0

R     ACCCCCATATAGTTGAAGGTGATGCTCTCAAGAATCGGCGTACCCCGCACCCACATAACTGGTCTAGATGCATTCATTATTTTTTTTCTCTTGTGAGGTCAGCCAACACCCAATTTATAACTGGTAA  1791
trnP  --------------------------------------------------------------------------------------------------------------------------------    57
trnF  --------------------------------------------------------------------------------------------------------------------------------     0

R     ATCTGGCTCTAAAACCTGAACATATAATGCCCGTGTAGTCTTACTATATACGCATGTTTTAATATAAGTCAATGTTGTATGGACATACACCTTCGTATTCCTCTGCCTGATTCCCAGAATATTACCTC  1919
trnP  --------------------------------------------------------------------------------------------------------------------------------    57
trnF  --------------------------------------------------------------------------------------------------------------------------------     0
```

```
R     CCAGTTTCCCCCCCCTCTTGCATTTCACAAAATAATTTTATTATACAATATATTTTTCTACTTTAAACCCCCCTCCCCCAAAATATAGGCTAATCGGCGCCTGAAATCTTTAGTCAAACCCCGAAACC  2047
trnP  --------------------------------------------------------------------------------------------------------------------------------    57
trnF  --------------------------------------------------------------------------------------------------------------------------------     0
```

```
                                                                                                                                trnF
R     TAAAAACCCATCACACTCACGCACTGAAATAACAAATGATTAATAATAATTTTATTAACTTTTAATATATTTTAATATATTTTAATATATTTTAATATATATTTTAATATATTGTAAGATATGCTAGT  2175
trnP  --------------------------------------------------------------------------------------------------------------------------------    57
trnF  --------------------------------------------------------------------------------------------------------------------------------     0
```

```
                             trnF
R     GTAGCTTACTAAAGC-ACGGCACTGAAAATGCCAAGACAGATAATAATTAAATCT  2228
trnP  ------------------------------------------------------    57
trnF  GTATTCTACTTAAGATATAACACTGAAGATGTTAAGACGGCCCCTAAAAAGGCC    54
                             trnF
```

1.120 NC_007178-NC_008975

Avg ovsize: -1122

LCA: Old World tree frogs-family

1.120.1 trnT-trnL1 -15

```
                            trnT                                                                     trnL1
R       -TTAACACCAAAGCATTGGTCTTGTAAACCAAAGATTGTAGACTAATATC-TACTCAGGACTAAAATTTATTTAAATCCAATTCTGGCTTTTAAAGGAAAAAAGAATTCCCCTGGCCTTAGGCGCCAG  126
trnT    CTTAAC-CTAAAGCATTGGTCTTGTAAGCCAAAGATTGTAAACTAA-ACCTTACCCAGAGCT------------------------------------------------------------------   60
trnL1   -----------------------------------------------------------------------------CTAATGCAG-CTTTTAAAGGAAAAAAGTCTTCCTCTGGCCTTAGGAGCCAG   50
                            trnT                                                                     trnL1
```

```
           trnL1
R      CACTTCTTGGTGCAAGT-   143
trnT   ------------------    60
trnL1  CATCTCTTGGTGCAAATC    68
           trnL1
```

1.120.2 trnL1-trnP -62

```
                                    trnL1                                                                   trnP
R      CTTAGGCGCCAGCACTTCTTGGTGCAAGTCCAAGTAAAAGCTTAGTTTAATACACAAACATATCAGAACAAGAAGAGTTTAAC-TTCTATCTTCGACCCCCAAAGCCGACATTCTAATTAAA-   121
trnL1  -----------------------------------------------------------------------------------------------------------------------------     0
trnP   --------------------------------------------------------------TCAAGACAAAAGGAATTTAACCTTC-ATCTCCGACCCCCAAAGCCGGCATTCTATTTAAAC    60
                                                                                                    trnP
```

1.120.3 nad5-trnT -3290

```
                                    nad5
R      GCTTACCTTGCGACTCTCTTCATCACGCTTGCCCTAGCACTCCTCCTAACACCCGAGTAGCAATCCTACACATACTATACATACTATGTATAATCAACATTCATACTATGTTTTTTATACATACTATG   128
nad5   ---TACCTGTCAACATTATTAATTACTCTTCTACTGACACT---------------------------------------------------------------------------------------    38
trnT   --------------------------------------------------------------------------------------------------------------------------------     0
                       nad5

R      TATAATCAACATTCATACTATGTTTTTTATACATATTATGTATAATCAACATTCATATATGTTTATACATACTATGTATAATCAACATTCATACTATGTTTTTTATACATACTATGTATAATCAACAT   256
nad5   --------------------------------------------------------------------------------------------------------------------------------    38
trnT   --------------------------------------------------------------------------------------------------------------------------------     0
```

```
R     TATAATAAATGAGGACTAAGGTTAACATCACACTTTTTGGACAATTCAATGAATGTACCGAGGAAATTAAATGAATGTAAAACAACACACAAATAATGTATATGATACCAAAAATGTAATTAAAATAA  384
nad5  --------------------------------------------------------------------------------------------------------------------------------  38
trnT  --------------------------------------------------------------------------------------------------------------------------------  0

R     CACTAATTGAAGAATACATTATTTCATAAACACTAGCGTATACATTCCATTAAGGCGGATGATCTCTGCAGCATATGATTAATACCACATATTATGAAAATTAGCACGGAATGATTGGCCGCCGCCCA  512
nad5  --------------------------------------------------------------------------------------------------------------------------------  38
trnT  --------------------------------------------------------------------------------------------------------------------------------  0

R     TATCATGACTACTTGATAGGACCTTCCCTTGTCTTAGGATATTCATATCTTGCCCAATGTTAGTCCAATTGCTAACCCACATACCATCATAAACAATGTTTATGTAATTAATATAACTTAACAGACCA  640
nad5  --------------------------------------------------------------------------------------------------------------------------------  38
trnT  --------------------------------------------------------------------------------------------------------------------------------  0

R     AACCTAACAACTTAAGACTCATATAAATGATTTAACACTAAACTGAATGCATCATTCATATAGACAAGGTAAAGCCTTCACTTGCTTAAGATCATGAATATGTATTACAACTAACCTTGTAAATCAAC  768
nad5  --------------------------------------------------------------------------------------------------------------------------------  38
trnT  --------------------------------------------------------------------------------------------------------------------------------  0

R     TATAATGGATCATTATATAGGACTTTCACAAAATGCAGGAAAAAATTCTTTTTTCCCCAGTATGAAAATTCTCTTCAAGTCATCTTCCACATGAATATTTTCTTCAAATGACCTGATATACTCAACTT  896
nad5  --------------------------------------------------------------------------------------------------------------------------------  38
trnT  --------------------------------------------------------------------------------------------------------------------------------  0

R     GACGCCTATTCATTAAAGCTAACGTCCAACAACCCAACGAATGCGCGGAAGCATCTCGCTGGTTAAGCTTCCAAGGGACCAAGGTCGTAGCCGACCAACTAGAACTTTTCAGGAGGTATCTGACGGAA  1024
nad5  --------------------------------------------------------------------------------------------------------------------------------  38
trnT  --------------------------------------------------------------------------------------------------------------------------------  0

R     CCGAATCTATGGACATACAAAACTTAATAAACATCTAGTGCTTTTTAAGAGGCATCCCTCTCTATGGAGTCGGGACCTTTATATAAGCTCAGACTACTTGTGAAGTCTGGGTCTTTCTGGACTGGGGG  1152
nad5  --------------------------------------------------------------------------------------------------------------------------------  38
trnT  --------------------------------------------------------------------------------------------------------------------------------  0

R     GGGGTAAACAAGAACTCATCAACATTGGACAGGGGGGGTTTCACTGTAAGATTCATATCCGAAACCGTGGAACAAGTCACACGCTTAGCAGTGCGCACCGCCCCTGTCTCAGTAAACGTCTCCGCCGT  1280
nad5  --------------------------------------------------------------------------------------------------------------------------------  38
trnT  --------------------------------------------------------------------------------------------------------------------------------  0
```

```
R     TTCAATGGTTATTCGGGTGGGACTAACAGCTATACTGGATGTCCTCCACTAGCCTGAACTCCAACGAGCATAACTTAATGCTAGACGGACATATTTTTCAACAGGTCCTTAATTCCACACACTATATT  1408
nad5  --------------------------------------------------------------------------------------------------------------------------------  38
trnT  --------------------------------------------------------------------------------------------------------------------------------  0

R     TCTCCCCCTTTTGCAATTTTTACAAATTTTTAAAAATAGAAAATTTTTCTGCCTACCCCCCCCTTTCCCCCCCCAAAAACAGACCAGCTAGCAACCCCTAGCGCCCCCCCGGGGCTAAGGACTAAAAA  1536
nad5  --------------------------------------------------------------------------------------------------------------------------------  38
trnT  --------------------------------------------------------------------------------------------------------------------------------  0

R     GATCTATTTGTGGGGGTTAACAAAGCACAACCCACAACCCTGCTGCTTTGAGATATGTTCAATATATTCGTGTCTTAATCATTTTATATGTGATATAACACGTAATATGTCTTATAAATGATGTATAC  1664
nad5  --------------------------------------------------------------------------------------------------------------------------------  38
trnT  --------------------------------------------------------------------------------------------------------------------------------  0

R     ACTATAAATGATGTATACACTATAAATGATGTATACACTATAAATGATGTATACACTATAAATGATGTATACACTATAAATGATGTATACACTATAAATGATGTATACACTATAAATGACGTATACAC  1792
nad5  --------------------------------------------------------------------------------------------------------------------------------  38
trnT  --------------------------------------------------------------------------------------------------------------------------------  0

R     TATAAATGATGTATACACTATAAATGATGTATACACTATAAATGATGTATACACTATAAATGATGTGTACACTATAAATGATGTATACACTATAAATGATGTATACACTATAAATGATGTATACACTA  1920
nad5  --------------------------------------------------------------------------------------------------------------------------------  38
trnT  --------------------------------------------------------------------------------------------------------------------------------  0

R     TAAATGATGTATACACTATAAATGATGTATACACTATAAATGATGTATACACTATAAATGATGTATACACTATAAATGATGTATACACTATAAATGATGTATACACTATAAATGATGTATACACTATA  2048
nad5  --------------------------------------------------------------------------------------------------------------------------------  38
trnT  --------------------------------------------------------------------------------------------------------------------------------  0

R     AATGATGTATACACTATAAATGATGTATACACTATAAATGATGTATACACTATAAATGATGTATACACTATAAATGATGTATACACTATATAAATGATGTATACACTATATAAATGATGTATACACTA  2176
nad5  --------------------------------------------------------------------------------------------------------------------------------  38
trnT  --------------------------------------------------------------------------------------------------------------------------------  0

R     TATAAATGATGTATACACTATATAAATGATGTATACACTATATAAATGATGTATACACTATATAAATGATGTACACACTATATAAATGATGTATACACTATATAAATGATGTATACACTATATAAATG  2304
nad5  --------------------------------------------------------------------------------------------------------------------------------  38
trnT  --------------------------------------------------------------------------------------------------------------------------------  0
```

```
R     ATGTATACACTATATAAATGATGTATACACTATATAAATGATGTATACACTATATAAATGATGTATACACTATATAAATGATGTATACACTATATAAATGATGTATACACTATATAAATGATGTATAC  2432
nad5  --------------------------------------------------------------------------------------------------------------------------------  38
trnT  --------------------------------------------------------------------------------------------------------------------------------  0

R     ACTATATAAATGATGTATACACTATATAAATGATGTATACACTATATAAATGATGTATACACTATATAAATGATGTATACACTATATAAATGATGTATACACTATATAAATGATGTATACACTATATA  2560
nad5  --------------------------------------------------------------------------------------------------------------------------------  38
trnT  --------------------------------------------------------------------------------------------------------------------------------  0

R     AATGATGTATACACTATATAAATGATGTATACACTATATAAATGATGTATACACTATATAAATGATGTATACACTATATAAATGATGTATACACTATATAAATGATGTATACACTATATAAATGATGT  2688
nad5  --------------------------------------------------------------------------------------------------------------------------------  38
trnT  --------------------------------------------------------------------------------------------------------------------------------  0

R     ATACACTATATAAATGATGTATACACTATATAAATGATGTATACACTATATAAATGATGTATACACTATATAAATGATGTATACACTATATAAATGATGTATACACTATATAAATGATGTATACACTA  2816
nad5  --------------------------------------------------------------------------------------------------------------------------------  38
trnT  --------------------------------------------------------------------------------------------------------------------------------  0

R     TATAAATGATGTATACACTATACGCTACACAAACACGTGTCTGCAGTAATTAAACATACAGGTGCCTGAACGCACACTGACGTGGTTTGTTCCCACGCATACACGGTGATCCTCACTAACCTCTACTT  2944
nad5  --------------------------------------------------------------------------------------------------------------------------------  38
trnT  --------------------------------------------------------------------------------------------------------------------------------  0

R     ATTTTTAACCCACCCTAAATTATTCTTCCTATTAAGCTTACTGCTAGAATATAAGGGTTATTTATACTTCCCCCTTTGTGCATATGTAATGTTCCGCACTTTTACATGAATAAATTCTTCATCATTTA  3072
nad5  --------------------------------------------------------------------------------------------------------------------------------  38
trnT  --------------------------------------------------------------------------------------------------------------------------------  0

R     ACCCCGACTATGATACCCTTTACCAATTTATTTTTATTGCATTAATACACATTTCCTGACTAGTTAGCATTAACTCTTTTCCTTCCTTGATAAACTATATTGTAGCATGCCTTGCATACGTAGTCGGA  3200
nad5  --------------------------------------------------------------------------------------------------------------------------------  38
trnT  --------------------------------------------------------------------------------------------------------------------------------  0

R     TCAAACTACGCTCTAACAGATTATTATAACCTACACAATATTTTAGTTATACTAGTAATGTATAACCAATGTTTATTGGCGTTCGCTCTATTTCTTAATGCAACTAACACACCCTTCTACCCGTATTT  3328
nad5  --------------------------------------------------------------------------------------------------------------------------------  38
trnT  --------------------------------------------------------------------------------------------------------------------------------  0
```

```
                                trnT
R       CTAAGTCCTGATAGCTTAACACCAAAGCATTGGTCTTGTAAACCAAAGATTGTAGACTA-AT-AT  3391
nad5    -----------------------------------------------------------------    38
trnT    ---AGCCCTGGTAGCTTAAC-CTAAAGCATTGGTCTTGTAAGCCAAAGATTGTAAACTAAACCTT    61
                                trnT
```

1.121 NC_008683-NC_012762

Avg ovsize: -1446

LCA: Teleostomi-superclass

1.121.1 trnL2-nad1 -3

```
                               trnL2                                                     nad1
R       AT-GGTAAATTGCGAAAGGCCTAAGCCCTTTA-AACCAGAGGTTCAAATCCTCTTCCCAGTTTATGCTAAACACTCTAATAAACCACCTGATCAACCCCCT-AGCCTACATCGT-GCCTGTCCT-  120
trnL2   CCCGGTA-ATTGCATAAAACTTAAAAC-TTTACCACCAGAGGTTCAACTCCTCTTC---------------------------------------------------------------------   54
nad1    -----------------------------------------------------------GTTTGTTCTAAAC-CTC--CTA--CTA-CTCATCATTCCCATTATCCTGG-CCATAGCCT-TCCTC   58
                            trnL2
                                                                                     nad1
```

1.121.2 rrnS-trnI -2890

```
                              rrnS
R       --GACAAGGGAGGCAAGTCGTAACATGGTAAGTGTACCGGAAGGTGCACTTGGATTAAACCCAGGGCGTGGCTGAGTTAGTCAAGCATCTCACTTACACCGAGAAGACATCCATGCAAGTTGGATCG  126
rrnS    ATTACTAGAGGAGATAAGTCGTAACAAGGTAAGCATACTGGAAAGTGTGCTTGGATCA---------------------------------------------------------------------   58
trnI    -------------------------------------------------------------------------------------------------------------------------------    0
                          rrnS
```

```
R     CCCTGAGCCAAACAGCTAGCTTAACCACCAATATAACCTAACAATGTTAATAACAAAACATGACTTAACACCACAAACTAAACCATTTTTTTACCTGAGTATGGGAGACAGAAAAGGTTCAACCCAAA   254
rrnS  --------------------------------------------------------------------------------------------------------------------------------   58
trnI  --------------------------------------------------------------------------------------------------------------------------------   0

R     GCAATAGAAAAAGTACCGCAAGGGAAAGCTGAAAGAGAAATGAAACAACCCATATAAGCACTAAAAAACAAAGACTAAACCTTGTACCTTTTGCATCATGATTTAGCCAGCACCCTCAAGCAAAGAGA   382
rrnS  --------------------------------------------------------------------------------------------------------------------------------   58
trnI  --------------------------------------------------------------------------------------------------------------------------------   0

R     CCTTTAGTTTGAAACCCCGAAACCAGGTGAGCTACCCCGAGACAGCCTATTTAAATTTAGGGCTAACCCGTCTCTGTGGCAAAAGAGTGGGAAGAGCTCCGGGTAGAAGTGATAGACCTACCGAACCT   510
rrnS  --------------------------------------------------------------------------------------------------------------------------------   58
trnI  --------------------------------------------------------------------------------------------------------------------------------   0

R     GGTGATAGCTGGTTGCCTGAGAAGTGGATAGAAGTTCAGCCTCGTACGCCCCAAATCAAAAAATATAACATTAAGACATAAGGGATACATACGAGAGTTAGTTAAAGGGGGTACAGCCCCTCTAACAA   638
rrnS  --------------------------------------------------------------------------------------------------------------------------------   58
trnI  --------------------------------------------------------------------------------------------------------------------------------   0

R     AGGATACAACCTTCACAGGAGGATAAAGATCATAATATATAAAACATGCTGTTTTAGTGGGCCTAAAAGCAGCCATCTAAATAGAAAGCGTTAAAGCTCAGACAGAAAGAAGTTTATTATACTGATAA   766
rrnS  --------------------------------------------------------------------------------------------------------------------------------   58
trnI  --------------------------------------------------------------------------------------------------------------------------------   0

R     AAAATCTTATTCCCCTAACAATATCAGGCTAACCCATGCCCACATGGAAGAAATTATGCTAAAATGAGTAACAAGAAGACCTGCTCTTCTCCAAGCACAAGTGTAAGCCAGATCGGACAGACCACTGG   894
rrnS  --------------------------------------------------------------------------------------------------------------------------------   58
trnI  --------------------------------------------------------------------------------------------------------------------------------   0

R     AAATTAACGAACCCAACCCAAGAGAGTAATGTGAATAATAGAAAAACCAAGAAAAACCCACAACCAAACAATCGTTAACCCCACACTGGAGTGCTATTTTTAAAGGAAAGACTAAAAGAAAGGGAAGG   1022
rrnS  --------------------------------------------------------------------------------------------------------------------------------   58
trnI  --------------------------------------------------------------------------------------------------------------------------------   0

R     AACTCGGCAAACACAAGCCTCGCCTGTTTACCAAAAACATCGCCTCCTGCAACTAAACTGAGTATAGGAGGTCCAGCCTGCCCAGTGACTACGGGTTCAACGGCCGCGGTATTTTGACCGTGCAAAGG   1150
rrnS  --------------------------------------------------------------------------------------------------------------------------------   58
trnI  --------------------------------------------------------------------------------------------------------------------------------   0
```

```
R     TAGCGCAATCACTTGTCTTTTAAATAGAGACCTGTATGAATGGCTAAACGAGGGCTTAACTGTCTCCCCCTTCAAGTCAGTGAAATTGATCTATCCGTGCAGAAGCGGGTATAACCATACAAGACGAG  1278
rrnS  --------------------------------------------------------------------------------------------------------------------------------    58
trnI  --------------------------------------------------------------------------------------------------------------------------------     0

R     AAGACCCTTTGGAGCTTAAGGTACAAAATTCAACCACGTTAAGCAACTTAATAAAAAGCAAAAACTTAGTGGAAAATGAAATTTTACCTTCGGTTGGGGCGACCACGGAGGAAAAACAAGCCTCCGAG  1406
rrnS  --------------------------------------------------------------------------------------------------------------------------------    58
trnI  --------------------------------------------------------------------------------------------------------------------------------     0

R     TGGAATGGGCCAAACCCCTAAAACCAAGAGAAACATCTCTAAGCCACAGAACATCTGACCAAAAATGATCCGGCTAATAAAGCCGATCAACGAACCAAGTTACCCTAGGGATAACAGCGCAATCCTCT  1534
rrnS  --------------------------------------------------------------------------------------------------------------------------------    58
trnI  --------------------------------------------------------------------------------------------------------------------------------     0

R     CCCAGAGTCCATATCGACGAGGGGGTTTACGACCTCGATGTTGGATCAGGACATCCTAATGGTGCAGCCGCTATTAAGGGTTCGTTTGTTCAACGATTAAAGTCCTACGTGATCTGAGTTCAGACCGG  1662
rrnS  --------------------------------------------------------------------------------------------------------------------------------    58
trnI  --------------------------------------------------------------------------------------------------------------------------------     0

R     AGCAATCCAGGTCAGTTTCTATCTGTAACGCTACTTTTCCTAGTACGAAAGGATCGGAAAAGAGGGGCCCATACTTAAAGCACGCCCCACCCCTAATTAATGAAAACAAATAAATTAAACAAAGGGAG  1790
rrnS  --------------------------------------------------------------------------------------------------------------------------------    58
trnI  --------------------------------------------------------------------------------------------------------------------------------     0

R     GGCCAAAACCCCAACCGCCCGAAATAAGGACATACTGGGGTGGCAGAGCATGGTAAATTGCGAAAGGCCTAAGCCCTTTAAACCAGAGGTTCAAATCCTCTTCCCAGTTTATGCTAAACACTCTAATA  1918
rrnS  --------------------------------------------------------------------------------------------------------------------------------    58
trnI  --------------------------------------------------------------------------------------------------------------------------------     0

R     AACCACCTGATCAACCCCCTAGCCTACATCGTGCCTGTCCTGCTAGCAGTAGCCTTCCTAACTCTAATCGAACGCAAAGTATTAGGATACATACAACTACGAAAAGGGCCAAATGTGGTCGGACCATA  2046
rrnS  --------------------------------------------------------------------------------------------------------------------------------    58
trnI  --------------------------------------------------------------------------------------------------------------------------------     0

R     CGGTCTCCTACAACCTATCGCCGACGGAGTAAAATTATTTATTAAAGAGCCAGTCCGCCCTTCTACATCCTCCCCATTCCTATTCTTAGCCACCCCCATTCTTGCATTAACTCTAGCCCTAACTCTAT  2174
rrnS  --------------------------------------------------------------------------------------------------------------------------------    58
trnI  --------------------------------------------------------------------------------------------------------------------------------     0
```

```
R     GAGCACCCATACCCATACCATACCCAGTAATTGATCTCAACCTAGGAGTTCTCTTTATCCTAGCCCTATCAAGCCTCGCAGTATATTCTATTCTAGGATCAGGATGAGCATCAAATTCAAAATACGCA  2302
rrnS  --------------------------------------------------------------------------------------------------------------------------------    58
trnI  --------------------------------------------------------------------------------------------------------------------------------     0

R     CTAATCGGAGCCCTACGGGCCGTAGCCCAAACAATTTCCTATGAAGTAAGCCTCGGACTAATCCTTCTATCAGTAATTATCTTCTCAGGAGGCTACACCCTACAAACATTTAGTACGGCCCAAGAAAG  2430
rrnS  --------------------------------------------------------------------------------------------------------------------------------    58
trnI  --------------------------------------------------------------------------------------------------------------------------------     0

R     TATCTGACTATTAGCCCCTGCATGACCATTAGCCGCAATATGATACATCTCAACCCTGGCAGAAACTAACCGAGCACCATTCGACCTAACAGAAGGAGAATCAGAGCTAGTCTCAGGCTTCAACGTAG  2558
rrnS  --------------------------------------------------------------------------------------------------------------------------------    58
trnI  --------------------------------------------------------------------------------------------------------------------------------     0

R     AATATGCAGGAGGGCCCTTCGCCCTCTTCTTCCTAGCCGAGTATGCAAACATCCTATTAATAAATACCTTATCAGCTGTACTATTTCTGGGAACATCACACATCCACCACATCCCAGAATTAACAACA  2686
rrnS  --------------------------------------------------------------------------------------------------------------------------------    58
trnI  --------------------------------------------------------------------------------------------------------------------------------     0

R     ATTAGCCTTATGACTAAGGCCGCGCTACTCTCTATTGTATTCCTCTGAGTACGAGCCTCGTATCCACGATTCCGATACGACCAACTAATACACCTTGTATGAAAAAACTTCCTCCCCCTAACATTGGC  2814
rrnS  --------------------------------------------------------------------------------------------------------------------------------    58
trnI  --------------------------------------------------------------------------------------------------------------------------------     0

                                                                                                                 trnI
R     TCTAGTACTATGACACATTGCCCTGCCAATCGCACTAGCGGGCCTTCCCCCACAACTATAGTTCAGGAACTGTGCCCGAATGCCTAGGGACCACTTTGATAGAGTGGCCTATAGGGGCTAAAATCCCC  2942
rrnS  --------------------------------------------------------------------------------------------------------------------------------    58
trnI  --------------------------------------------------------------------------------------------------------------------------------     0

        trnI
R     TCAGTTCTT  2951
rrnS  ---------    58
trnI  ----TTCC-     4
          trnI
```

1.122 NC_008975-NC_016059

Avg ovsize: -1588
LCA: Ranoidea-superfamily

1.122.1 trnS1-nad6 10

```
R      ATTGGGATAATGAGTACTGCTAATTCCTCACCACCATGGTTCGATTCCACGGCTAGCTCGAATACCTCACAGCCCGCAAAGTTCCAGC-C--TTGTGCCCACGAACCACCTCTAATACAACAAATA   123
trnS1  CTGGAG-TAATGAGAACTGCTAATTCCTCACCCCCATGGTTCAATTCCATGGCCCGCTCG--TA-CTTATA-CTC---------------------------------------------------    70
nad6   -----------------------------------------------------------------CTTACAGCTCGTAAGGTCCCCCCTCACTTGTATCCACGCACCACCTCTAAGGCAACAAA--    59
```

1.122.2 nad5-trnL1 -82

```
R      TACCTGTCAACATTATTAATTACTCTTCTACTGACACTTATTTTTGTACAGTTTTTTAGAACTCATGAGAACACCTAATGCAGCTTTTAAAGGAAAAAAGTCTTCCTCTGGCCTTAGGAGCCAGCATC   128
nad5   --------------------------------------------------------------------------------------------------------------------------------     0
trnL1  ----------------------------------------------------------------------------------GCTTTTAAAGGAAAAGAGCCCTCCACTGGCCTTAGGAGCCAGCATC    46
```

```
R      TCTTGGTGCAAAT-   141
nad5   --------------     0
trnL1  TCTTGGTGCAAGTC    60
```

1.122.3 cob-nad5 -4694

1.123 NC_015305-NC_001922

Avg ovsize: -1671
LCA: Tetrapoda-superclass

1.123.1 trnL1-trnT -7

```
                                  trnL1                                                    trnT
R      AAACAG-CCCTCCACTGGCCTTAGGAGCCAGCACCTCTTGGTGCAAGTCCAAGT-AAAAGCTGCCGTCCTGATAGCTTAACTAAA-GCACTGGTCTTGTAAGCCAGAGATTGCAGCCTGACCT-CT   122
trnL1  AACTAGGACATCCCCTGGCCTTAGGAGCCAATA-ATCTTGGTGCAACCCCAAGTGAAAA-------------------------------------------------------------------    58
trnT   ------------------------------------------------------------------TCCCG-TGGCTAAAGGAAAAGCGCTGGCCTTGTAAGACAGA-AGTGGA-CGGAACCAACC    57
                                trnL1
                                                                                            trnT
```

1.123.2 trnS1-nad5 -34

```
                                 trnS1                                                                     nad5
R      TGCCCGGCATAGTGAGAACTGCTAATTCCTCACCACATGGTTCGACTCCATG-CTCGCTCGCACTTGCACTTCACTAAATCCCAGTACGAGTCATGCCATCCCTATATACAATTACTTTTGTGACAAC   127
trnS1  -CCGCG-CA--G-GA--ACTGCTAATTCCTAGCCCCCAGGACTAACAGCCTGGCCCTCTCG-------------------------------------------------------------------    54
nad5   -----------------------------------------------------------------------------------------------GCCGACCCTATCT-C--TTACCTTCTTCACC-C    29
                                 trnS1
                                                                                                            nad5
```

```
                    nad5
R      AATAT-CTGCAATCGCCTTAATTAT   151
trnS1  -------------------------    54
nad5   TACCTGCTTTTATC-CTTTTACTA-    52
                    nad5
```

1.123.3 cob-trnH -4972

1.124 NC_015305-NC_016059

Avg ovsize: -1685

LCA: Ranidae-family

1.124.1 nad4-trnS1 -32

```
                                   nad4                                                                     trnS1
R      --CT--TCAT--AT-TCT-ACCCGGACTTCTATTTATCCTCAAGCCAGAGCTACTTTTCTGTGTATAGCCCACAACAGCATAGTGACTCCCCGAGCCTGCCCGGCATAGTGAGAACTGCTAATTCCTC   120
nad4   TTCTCCTCCTCCATATCTTACCAGGCCTCCTCCTTGTACTCAAACCAGAATTAATTTTCT--------------------------------------------------------------------    60
trnS1  --------------------------------------------------------------------------------------------GAGCCCGACTGGAGTAATGAGAACTGCTAATTCCTC    36
                              nad4
                                                                                                               trnS1
```

```
                  trnS1
R      ACCAC-ATGGTTCGACTCCATGCT   143
nad4   ------------------------    60
trnS1  ACCCCCATGGTTCAATTCCATGG-    59
                  trnS1
```

1.124.2 trnH-trnL1 -110

```
                                  trnH
R      ATTAAACAATAATGCTAGATCGTGACTCTAAAATTGAAAGTTAAAATCCTTCTGCTCATCTACACTAATCCTCATTCAACTCGTGCAAACAATGGTCCCCCCCTACCTCTCAGTATACCGCCCTGATT   128
trnH   GTTTAACAAAAACCCTAGATTGTGATTCTAGAAACAAAGGTTGAACCCCTTTTATTCACC--------------------------------------------------------------------   60
trnL1  --------------------------------------------------------------------------------------------------------------------------------   0
                                  trnH

                                                                                       trnL1
R      ACCCCCAACGTATGCTGACACAAAGTCACAAAAATTAACCCGCTTTTAAAGGAAAACAGCCCTCCACTGGCCTTAGGAGCCAGCACCTCTTGGTGCAAGT-   228
trnH   -----------------------------------------------------------------------------------------------------   60
trnL1  ------------------------------------------CTTTTAAAGGAAAAGAGCCCTCCACTGGCCTTAGGAGCCAGCATCTCTTGGTGCAAGTC   59
                                                                                         trnL1
```

1.124.3 cob-trnH -4914

2 TDRL

2.1 NC_009689-NC_007895

Avg ovsize: 15

LCA: Coleoidea-subclass

2.1.1 trnD-atp8 69

```
                         trnD                                                   atp8
R     AAAGTTAGTTAATT-TATA-ACCTA-AAGTTGTCAACTTTAAATTACT-T-TTCAGTACTTTTCTATGCCACAATTATCACCTATTAATTGAATTTTTTTTATTCTCTTTTTTTTGATTTATAAT-   119
trnD  ----TTAGTTAATA-TATA-ACATA-AAATTGTCAATCTTAAATAACTATATTCA-TAGTACTTTTACCACTATAA--------------------------------------------------    69
atp8  --------TTAAAAGTAAACATATATAAATTTTCTATTTTTAATAAATAAATT-A-TACTTTTATATGCCTCAATTATCCCCTATTAATTGAATTTTTTCTATTTTTCACATTCTGATCTATTTTA   115
                           trnD
                                                                                atp8
```

2.1.2 cox2-trnD 59

```
                        cox2                                                  trnD
R     ATTACATTAGAAATTATTAATATAAAATCATTTATTAAATGATT-AACAATAATAAATTAATAAGAAAAGTTAGTTAATTTATAACCTAAAGTTGTCAACTTTAAATTACT-T-TTCAGTACTTT   122
cox2  ATTACTTTAGAAATTGTAAATATAGAGTATTTTATTAAATGATTAAACCA-AATAAACTAATTTTAAAAGT-A---AACATAT-A--TAAATTT-TCTATTTTTAATAAATAAATT-A-TACTT-   113
trnD  -----------------------------------------------------------------AAAAGTTAGTTAATATATAACATAAAATTGTCAATCTTAAATAACTATATTCA-TAGTAC    59
                        cox2
                                                                              trnD
```

2.1.3 trnF-nad5 26

```
                        trnF                                                        nad5
R     ATTATTATTTTTAATTACCTTAACACCTTCAACGTTACGCTCTA-TTTAAGCTACTTAGAT-TAAA-TAAATATA-A-TAAT-AACTATAAATAAAATTAAATTAA-TAT-TAAAAACTAAAC-TTAC   119
trnF  -TAAAGGTTATTAACCACCTTAATATCTTCAGCATTATGCTCTAATTTAAGCTATTTAAAT------------------------------------------------------------------    60
nad5  -----------------------------------TAAGATATACTTTTACATGTTTATATATACATTATATATACATTAATAAACT-TA-TTAAAATAAAAATAATTATATAACAATTTAATAGTAC    91
                        trnF
                                                                                    nad5
```

2.1.4 trnG-trnE 15

```
                                    trnG                                                                         trnE
R     --ATTTAAAATTTTAAACCTTTTGCTTGGAAAACAAATATACTATAAATATACTAATAAAATATTATTCTTA-ATATAA-TATATTA-ATATCCCTAGAA-ATTGAAAATCTCTTGTGC-AC-A-TAC   119
trnG  TTATTTAATATTTTAAACCCTTTGTTTGGAAAACAAACATACTTTT--TATACTAATAAAGTA-TATACATATATATAACAATCT-------------------------------------------    82
trnE  ----------------------------------------------------------------------TATTTTTAA-TATCTAAGATATTC-TAAAAGATTGAAAATCTAATGTGCTAGTATTAC    56
                                    trnG
                                                                                                      trnE

      trnE
R     AC--   121
trnG  ----    82
trnE  ACTA    60
      trnE
```

2.1.5 trnW-trnQ 10

```
                                    trnW                                                                      trnQ
R     --AAATTTATATATTTATTATAAACCTTG-AAAGCTTAAAGTTTTAAATTAACCTAAGATCTTACAAAAAAAGGATTTGAACCTTTTCCTTAAAGATCAAAACTTTATATGCCCAT-ACA-C--   117
trnW  TTAAATTTATTAATTTATTAAAGACTTTGAAAATCCTCA-GTTTT--TTTAACTTAAAATCTTA------------------------------------------------------------    61
trnQ  ------------------------------------------------------TAAAACATTACAAAAG--GGTTTTGAAC-TCCTCCTTAAACTCCAAAAATTTATATGCTCATTACACCAC    67
                                    trnW
                                                                                                     trnQ
```

2.1.6 trnY-trnW 5

```
                              trnY                                                        trnW
R     A-AGCA--TTAT-ACTTATTCATAGATTTACAATCTACCGACTTTAATTTTCGACCACCTCATACAAGATCTAAATTTATATATTTATTATAAACCTTGAAAG-CTTAAAGTTTTA-AA-TTAACC   119
trnY  TGAGAAAGTAATAACTTATACATAGATTTACAATCTACCGACTTCAA-T--CGACCACCCCATAAAA-----------------------------------------------------------    64
trnW  --------------------------------------------------------------TACAAGATTTAAATTTATTAATTTATTAAAGACTTTGAAAATCCTCAGTTTTTTTAACTTAA--    62
                              trnY
                                                                                 trnW
```

2.1.7 trnR-trnN 3

```
                              trnR                                                    trnN
R     AGAAATGAATTA-TCATTTATGGTTTCGACCCATAATTTTGGTGTTTAACACCCTTAT-TTCAACAAAAAGCCAAAT-AGCGGCATTTAATTGTTAATTAAAAAAATGTAATT-T-ATTACTT   118
trnR  A-AA-TGATTTAATCATATATGATTTCGGCTCATACTT-AGATGTCACACATCCTTGT-----------------------------------------------------------------    55
trnN  -------------------------------------------------------TGTGTACAGTGGAAAGCCAAATGAGAGGCCTTTAACTGTTAATTAAATTATTGTAATTATGATTAC--    66
                              trnR
                                                                              trnN
```

2.1.8 rrnS-trnM 3

```
                              rrnS                                                          trnM
R     -TATTAAACCTAACATATATTATCTAATAAAA-ACTAATATTATAACCAGAATCAAATTA-ATTAATAA-AAAGATTTTTAAAAATCTATTTTTGGGGTATGAACCCAATAGCTTACA-T-TTAG--   119
rrnS  ATAATATATAT-TTAT-TATTGACCAATTAAACACTAAT-T-AT-ACC----TCAATTTATATT-ATAA----------------------------------------------------------    59
trnM  ------------------------------------------------------------------TAATAAAGAAATC-ATATATTTATTTTCGGGGTATGAACCCAATAGCTTAAAGTTTTAGCT    60
                                rrnS
                                                                                   trnM
```

2.1.9 trnM-trnC 1

trnM *trnC*

```
R      ATTTTTAAAAATCTATTTTTGGGGTATGAACCCAATAGCTTACA-TT-TAGCTTACTTTATT-AAGTTTTAACATAAT-ATGTATCCATGAATTTGCAATTCATTATTTTTAAATTAAACTAC-  119
trnM   AATCATATAT-T-TATTTTCGGGGTATGAACCCAATAGCTTAAAGTTTTAGCTGACTTTATTTA------------------------------------------------------------  62
trnC   ---------------------------------------------------------------AAACTTCAATATATTAATATATCCCCAAATCTGCAATTTGTTATTTT-AATTTAAACTATA  60
```

trnM *trnC*

2.1.10 trnP-nad1 -1

trnP *nad1*

```
R      AATCATTTTTATTTGATCAATAACTTCCAATGTTATTATTTT-ATTTTAAACTATTTTCTATTACAAATTTAAAAATCTAA-CAATTATAATTATTAATATAATAATACATAAAACAGAAG-  119
trnP   -ATAAGTATTCCTTAATCACCAACTTCCAATGTTAATATTTTAATTTTAAACTATTTTTT--------------------------------------------------------------  59
nad1   -------------------------------------------------------------TTATATATTCAA-AATATAAGCCAATATCACTAAAAACATTAAAATATTCAATACTCTTGG  60
```

trnP *nad1*

2.1.11 nad3-trnS1 -17

nad3 *trnS1*

```
R      TATTAATTTTACTTCTTGGACTTTATCATGAATGAAATCAAGGATCATTAAATTGACTATAAAAGTAATTAAAATTTTTTTTAAAAAGAAGCTGCTAACTTTTTTTTGAGCA--T-TT--TG--A-A  119
nad3   TA--A-TTTTACTTATAGGGCTGCTCCACGAATGAAACCAAGGCTCTTTAAACTGA--ATAAAA---------------------------------------------------------------  59
trnS1  ---------------------------------------------------------------------------------TAAAATAAAGCTGCTAACTTTATTTTGAGCAGCTCATACTGTTCTA  46
```

nad3 *trnS1*

2.2 NC_015310-NC_023250

Avg ovsize: -23
LCA: Unioninae-subfamily

2.2.1 trnH-trnA 55

```
                                        trnH
R     -GAGAGTGCTACAACCTATTTATAGAACCACAACCTATCGTTTTTTTTAAACTACTCATTAC-C-C-CCATTATA-AATATTCTATTTATT-AT-TTTTACCAATA-ATAAACTATAAATAA-AATTA   119
trnH  CGA-AAAGATCTTTCCCATCTATAGAACCACAATCTATTGTTTTCCTTAAACTACTCGTTACTCTCTCTAATATAGAATCT-CAATT-ACT-AT-TCTTCACAAAATCTTTAATA-ACTAAAGTATTC   122
trnA  ----------------------------------------------------------------------------AAACTTCCATTTACTTATATTTTAATAATA-CTA-ACTA-ACTCAA-AAATC    48
                                        trnH

                                            trnA
R     TTA--TAA-AT-A-A-T---AGTTCTATACTTTAACAAAAAGATTTGATTTGCATTCAACCATTGAGATATTACTCTAG-   189
trnH  TTA-----------------------------------------------------------------------------   125
trnA  TTACCTAAGATGAGACTACAAGTTCTATACTTTAATCAAAAGACTTGATTTGCAATCAGCCATTGAGTGATACCCCAACA   128
                                   trnA
```

2.2.2 trnM-trnW 41

```
                                       trnM                                                                  trnW
R     GTAAGCTAATAACTA-AGCTGTTGGGCTCATAACCCAAAAATAGAAACTCTCTTCCTACTATC-TCA-CTT-TAC-CTTAAGGAATTTAAGTTAAATA--AAACT-AATAGCCTTCAAAGCTTTAAAT   120
trnM  -TAAGCTAACCTCTAAAGCTGTTGGGCTCATAACCCAAAAATAGACACATTCTTCCCACTAAGAACCCCTTCTACACCTCAAACAAAAAA-TCAAAAAGTAAAATCAATAGCCT--------------   112
trnW  -------------------------------------------------------------------------ACACTACAGAGATTTAAGTTAAACA--A-ACT-AATAGCCTTCAAAGCTTTAATT    51
                                       trnM
                                                                                                  trnW
```

```
        trnW
R       GATCTAAAATCAA--T  134
trnM    ----------------  112
trnW    GACACCAAGTCAATCT   67
        trnW
```

2.2.3 trnS2-trnS1 23

```
        trnS2                                                                trnS1
R       GACCTCGGAGAAGATTTGCCTTGAAATCAAAAAGAAAGTGTTCGACTC-ACTTATCCTTTTTCTGGAAAGGTAGCCGAGCTAATATGTGGCTTCTAACTACATAAAGAGAGGTTTAACTCCTTC  123
trnS2   GACCTCGGAGAATATTTGCCTTGAAATCAAACAGAAAGTGTTCGACTC-ACTTATCCTTTTATAG-----------------------------------------------------------   64
trnS1   -------------------------------------------CAACCTACCTAAACTTATAAAGAGAA-GTAGCCGAGCTAATATGAGGCTTCTAACCACATAAAGAGAGGTTTAACTCCTT-   80
        trnS2
                                                                     trnS1
```

2.2.4 cox2-nad3 4

```
        cox2                                                                      nad3
R       AAGCTCAGAACTCAAACCACTATAACTATCTTGAAACCCTAATTGACCCCAAAAGCTCATTTT-T-TATTTTATTTTTATCCGCCATTATATCTCCCACTCTAATGAACCCTCACGCCACTCGTGAAC  126
cox2    CAACTCAAAACTTAAAACTCTAAAACTATCCTGAAACCCTAACTGACCCCAAAAGCTCATTCACTCCATACTACCATTAACCGCCCC-----------------------------------------   87
nad3    -----------------------------------------------------------------------------------CCCCTACCCCTCCCACTCTAAAGAACCCTCTCGCCACTCATGAAC   45
        cox2
                                                                                      nad3
```

```
                  nad3
R       TACTCCACCAAACAAT-A   143
cox2    ------------------    87
nad3    AACCCCCCCAAATAACAA    63
                  nad3
```

2.2.5 trnS1-trnE 3

```
                                 trnS1                                                                   trnE
R       GCCGAGCTAATATGTGGCTTCTAACTACATAAAGAGAGGTTTAACTCCTTCCACCTTTCTTTTAGCTAATTAAGTGTTAGGTTTTATGCACATTGATTTTTCACGTCAAAAGAGCATT-TTA-G-T   123
trnS1   GCCGAGCTAATATGAGGCTTCTAACCACATAAAGAGAGGTTTAACTCCTTTCATTTCTCTCTAAACTA----------------------------------------------------------    68
trnE    -----------------------------------------------------------------CTAATTAAGTGTTCA-CT-TACGCACATTGACTTTTCACGTCAAAAGAGCATACTAATGCA    59
                                 trnS1                                                                   trnE
```

2.2.6 nad3-trnH -17

```
                                 nad3
R       ACAT-AATAATACAA-ACCCAAGCAAGACAGCTACCAAAACCCTTAACACCACTTTTTCCATATCACCTTACAA--AGGTAAAATTTAATGTTTAATACACCAAA-ACCTAAAACCCTTAATGCCT-C   122
nad3    -CACCAACAACACAAGACCCAA-TACTAAAGATAACAAAATTCTTACTATAACTTTTTCCATTT-ACCTAATAACTAGATAACA--------------------------------------------    81
trnH    -----------------------------------------------------------------------------------------------------CAAATACC-AAAACTCACATTTCATTC    26
                                 nad3
```

```
                                  trnH
R      TAACACTAATGAGAGTGCTACAACCTATTTATAGAACCACAACCTATCGTTTTTTTTAAACTA--C   186
nad3   -------------------------------------------------------------------   81
trnH   CAAC-CTAACGAAAA-GATCTTTCCCATCTATAGAACCACAATCTATTGTTTTCCTTAAACTACTC   90
                              trnH
```

2.2.7 trnE-nad2 -275

```
                         trnE
R      --GTGTTAGGTTTTATGCACATTGATTTTTCACGTCAAAAGAGCATTTTAGTGCACTTAGCTATTTAAAAGTTCGACCAAAACTTT-AAG--TAATCTTTAACCAAAAACTTCAAAAGCTACTATCCA   123
trnE   AAGTGTTCA--CTTACGCACATTGACTTTTCACGTCAAAAGAGCATACTAATGCACTTAGCTACCCAAAAACACACACCCCCAACTCACCAGCAAAATCTTTA---------------------------   99
nad2   --------------------------------------------------------------------------------------------------------------------------------   0
                        trnE

R      TACTTATACTCTCTTTCTTAATTGTCCTATGCCTAACCTTAAGTATTCCCATATTTATCCTTTCTAACGAACCAAATCTTACTAACCAAACACTATGTTCAATAGACCTTAACAATCAACCTCCACAA   251
trnE   --------------------------------------------------------------------------------------------------------------------------------   99
nad2   --------------------------------------------------------------------------------------------------------------------------------   0

                                                                                                                                     nad2
R      CCCAAAACAGATAATTACCATATTACTGCCAGATCAGGGAACACAGACTTGACCAAACCAAACCAAGCATCCAGCGTCAGAAAAAAGTAATGACCATAACTACTACTTTCAACTTTTTTATAAATGAA   379
trnE   --------------------------------------------------------------------------------------------------------------------------------   99
nad2   ------------------------------------------------------------------------------------------------------------------------TAAATGAA   8
                                                                                                                                    nad2
```

nad2

```
R     ATCTCCAAACAAACTCCTATTTATATTTCTTATAGTTAGAAGCACTTGCATAGT-   433
trnE  -------------------------------------------------------   99
nad2  ATCGCCACACAAATTCTTATTCCTATCCCTAATAATCAGAAGCACTTGCATAGTA   63
```

nad2

2.3 NC_005144-NC_005298

Avg ovsize: -27

LCA: Elopomorpha-superorder

2.3.1 nad6-trnE 44

nad6 *trnE*

```
R     AGCTACAGCAACAAGA-CCAAGAACCAACCCAATTAAAAATAAAAAAATGAAATAAG-TCACAATTTCTACCCGGATTTTAACCGAGACCAATGACTTGAAAAACCACCGCTGTTATTCAA-   119
nad6  AGATACACCCAAAACACCCAACAA-CAGCACCGCTAAAA-GAGAATAATTAAGTAAGTTCATAAATCTTATAAGGATTCAACCTAATACCACAAATGTGATAA-------------------   101
trnE  -----------------------------------------------------------CATAGTTTTTACCCAGATTTAAACTGAGACCGATGATATGAAAAACCACTGATGTAATTCAAC   63
```

nad6

trnE

2.3.2 trnK-atp8 10

```
                        trnK                                      atp8
R     ACAGGGA--ATAGCGTTAGCCTTTTAAGCTAAAGAATGGTGACTCCCAACCACCCATAGTGACATGCCTCAATTAAACCCCGCCCCATGACTTGCCATT-TTATTATTCTCTTGACTAATATT-  120
trnK  -CTGAAATGACAGCACTGGCCTTTTAAGCCAGA-AATGGTGGCTCACAATCACCCTTAAT-AGATGCCGCA-----------------------------------------------------   68
atp8  -------------------------------------------------------------ACATGCCGCAGCTGAACCCAGACCCTTGATTTATAATCCTTATCGT-CGCATGGGTCGCCTTT   62
                        trnK                                      atp8
```

2.3.3 trnW-trnA 3

```
                        trnW                                      trnA
R     -TAGCATTAAGACCAAAAGCCTTCAAAGCTTTAAGCAGGAGTGAAAATCTCCTAGTTTTTGA-TAAGGCTTACGGGACTCTAACCCATATTTTATGAATGCAACTCAAACGCTTTAATTAAG-  120
trnW  ATAAGAGCA-GACCTAAAACCTTCAAAGTTTTAAGCAGGGGTGGAAATCCCCTAGCCTTTGACTAA---------------------------------------------------------   65
trnA  ---------------------------------------------------------------TAAGACCTGCAAGACTTTATCTTGCATAATCTGAATGCAACCCAGATACTTTAATTAAGA   60
                        trnW                                      trnA
```

2.3.4 trnQ-trnM 2

```
                        trnQ                                      trnM
R     GGACTTGAACCCATCCTCAAGAGATCAAAACTCTTGGTGCTTCCACTACACCACTTCCTA-GTAAAGTCAGCTAATAAAGCTCTCGGGCCCATACCCCAAGAATGTTGGTTAAAATCC-T  118
trnQ  GGGGTTGAACCCTTTCCGAAGAACTCAAAACTCTCAGTGCTTCCATTACACCACCTTCTA------------------------------------------------------------   60
trnM  ----------------------------------------------------------TAAGTAAAGTCAGCTAATAAAGCTCTTGGGCCCATGCCCCTAGTATGTCGGTTAAAGTCCTT   62
                        trnQ                                      trnM
```

2.3.5 trnI-trnQ 2

```
                                   trnI                                                        trnQ
R     GAATATTAAAGGGC-CACTTTGATAGAGTGGATAATAAGGGTTAAACTCCCTTTGTTTC--CTAGAAAGAAGGGACTTGAACCCATCCTCAAGAGATCAAAACTCTTGGTGCTTCCACTA-   117
trnI  -AATGTCCCAGGGGGTATTTGATAGAAATACACCATGAGGGTTAAAGCCCCTCCGCTTCTTCTA---------------------------------------------------------    63
trnQ  --------------------------------------------------------------TAGAAAGAAGGGGGTTGAACCCTTTCCGAAGAACTCAAAACTCTCAGTGCTTCCATTAC    59
                                   trnI
                                                                                               trnQ
```

2.3.6 nad3-trnR 2

```
                                   nad3                                                        trnR
R     TTTTATTAACCCTCGGCCTAATTTACGAATGACTTCAAGGCGGC-CTTGAATGAGCTGAATAGATGGTTAGTCCAAGA-AAGATTACTGATTTCGACTTAGTAGATTATGGTTAAAGTCCA   119
nad3  CTT--GTAACACTAGGCTTCGCCTATGAATGA-GTCAAGGGGGGACTAGAGTGGGCCGAGTAG----------------------------------------------------------    60
trnR  -------------------------------------------------------------AGACTATTAGTATAATTTAAGACACCTGACTTCGACTCAGACCCTCATGGTTAAAATCCA    60
                                   nad3
                                                                                               trnR
```

2.3.7 cob-trnT 2

```
                                   cob                                                         trnT
R     TATTTCTT-GTATTCATCCCAATAGCAGGATGAGTTGAAAATAAAATAATTGAATCTAATTGCCCTAGTAGCTTAGACTAAAGCACCGGTCTTGTAAGCCG-GAGATTGAAGGTTAAA-ACC   119
cob   CACTT---------------------------------------------------------------------------------------------------------------------     5
trnT  ---TTATAAGGATTCAACCTAATACCACAAAT-GT-GATAAT--AATAA--GAATCTCGCT-T-C-AGTAGCTTAAA-TAAAGCATCGATCTTGTAAGTCGAAAGCCGGGTTCTTAAATTAC   109
      cob
                                                                                               trnT
```

2.3.8 trnS1-trnL1 -5

```
                                trnS1                                                     trnL1
R      CATGAAGCATT--AAGAACTGCTAATTCCTTAATTCCAAAGTTAAAATCCTTGGCTCCCTC-AAGTCTTTAAAGGATAATAGATCATCCATTGGTCTTAGGAACCAAAAACTCTTGGTGCAAC-   120
trnS1  -A-GACGTATTCCATGAGTTGCTAGCCCTTTTGAACCACGGTTGAAGCCCGTGGCCCCTTCGAA------------------------------------------------------------    62
trnL1  ---------------------------------------------------------------------TAAAGGATAATAGCACATCCATTGGCCTTAGGAGTCAAAAATTCTTGGTGCAACC    55
                                trnS1                                                          trnL1
```

2.3.9 nad1-trnI -5

```
                               nad1                                                       trnI
R      TTAGTTATTTGACACATAGCCCTTCCAATTGCCATAGCAGGACTTCCACCCCAAAC--TTAAGGGTACTGGAA--ATA-TGCCTGAATATTAAAGGGCC-ACTTTGATAGAGTGGATAATAAGGGTTA   122
nad1   CTATTGATCTGTAACTTATCTCTAATTACAGCACTAGCCGGACT-CC-CCCCAAACAATTAA------------------------------------------------------------------    60
trnI   -------------------------------------------------------------------CTGGAAGAATCGTGCCCGAATGTCCCAGGGGGTATTTTGATAGAATACACCATGAGGGTTA    61
                               nad1                                                          trnI
```

```
       trnI
R      AACT   126
nad1   ----    60
trnI   AAGC    65
       trnI
```

2.3.10 nad2-trnW -55

```
                                  nad2                                                     trnW
R     -TTACCACCACTGGCACACTCATACTATTACCTTTGACCCCAACAATGACAACAATGCTCTAAGAACTTAGGATAGCATTAAGACCAAAAGCCTTCAAAGCTTTAAGCAGGAGTGAAAAT--  119
nad2  GTTGCAACAAC---------------------------------------------------------------------------------------------------------------  11
trnW  ------------------------------------------------------------------CTTAGGATAAGAGC-AGACCTAAAACCTTCAAAGTTTTAAGCAGGGGTGGAAATCC  55
        nad2                                                                                      trnW
```

2.3.11 trnP-trnF -297

```
                                  trnP
R     GGAGAGTTTAACTCCTATCCTTGGCTCCCAAAGCTAAGATTTTA-ATTA-AACTACCCCGTG-C--TA-ACGCAC-A-C-CAAAAA--AATTC-T-AAAACC-TGCGGGTATGTACTATAATACATAC  114
trnP  AGA-A-TTAAACTCCCACCCCCGGCCCCCAAAGCCGGGATCCTCTATTAGAAC-ACCCTCTGACAATATATGTACTAATGCATACATGCATACATGCATATTTTGCATGTATGTACTA-G-TACATAT  123
trnF  --------------------------------------------------------------------------------------------------------------------------------  0
                                  trnP

R     TATGTATTATCTTACATAAGTTATGTATACCGGGACACATTGTGTAAAACAGTACATTTCCTTGTTTGAAC-AG-ACAAA-ACTCAATAATTCAACATAAATCCTGCAAAAGTAGTTAATGTGCAGTA  239
trnP  TATGCATAATTTTGCAT--GT-ATG--TACTAGTACATATTATGCATAA---T---TTTGCATGTATGTACTAGTACATATTATGCATAATTTTACATGTAT-GT--ACTAGTACATATTATGCATAA  237
trnF  --------------------------------------------------------------------------------------------------------------------------------  0

R     CCTTACATATTAACCCGATCAAGAAATTTAATTACAATCTATAAAGAAATAAACCATATTGTTTGGTAATAATAACGGATGTGGACATATAATACACAAATCGTTATCAACATGAACTCCTATTAACG  367
trnP  TTTTACATGT----------------------------------------------------------------------------------------------------------------------  247
trnF  --------------------------------------------------------------------------------------------------------------------------------  0

R     TAAAGAAATAGCTTGTCTCGACATAACTTATCATACCCATATTCATCGAAGTCCCCACATCCTTGCCAATATTAATTAAATTTGTAATAAGAAACCAGCAACGGTGATATCAGGTGAAATCGTTTATT  495
trnP  --------------------------------------------------------------------------------------------------------------------------------  247
trnF  --------------------------------------------------------------------------------------------------------------------------------  0
```

```
                                                                                  trnF
R     GAACGATCAGGGGCAATAATAGTAGGGGTTGCACAGAGTGAACTAGTCCTCGTAGCTTAA-ATTAAAGCATGGCACTGAAGATG-CTAAAATGAGCCCTCATAAGC   599
trnP  ----------------------------------------------------------------------------------------------------------   247
trnF  ---------------------------------------------------GTAGCTTAACACTAA-GCATGACATTGAAAATGTCAAGAAGGCGCTTTAAAACGC    54
                                                                            trnF
```

2.4 NC_013884-NC_005929

Avg ovsize: -31

LCA: Holothurioidea-class

2.4.1 trnA-trnW 23

```
                                   trnA                                                      trnW
R     --CCAGGAAAT-TC-CTAGATCTCATGCGTGCAAAGCAAGCGTTTTATAATAAACTAAAATCACA-AAGACTTAAGTTAA-AAGACTGTAAGCCTTCAAAGCTTAAAGCGTAGGTTAAACTCCTA   119
trnA  TTCCA--AAATATCTTTGGATCATTTGCATGCAAAGCAAATATTTT-T-ATAAACTAAAATCACC-AAAAATTAAACAAA-AACA----------------------------------------    79
trnW  --------------------------------------------------------------ACCGAAGATTTAAGTTAATAAAACTTTGAGCCTTCAAAGCTCACCACACAAATCAAAATTTTG    63
                                  trnA
                                                                                             trnW
```

2.4.2 trnV-trnM 22

```
                                           trnV                                                      trnM
R     CTGAGTTGCACAGGTATCAACTCCGTGTAAAAGAGACACTTTCTTCTTAGCTATCCCTTGTGACATTTATATAAATGCTTTAAGTAGAG-TAAGCTAAAAAAGCTTTCGGGCTCATATTCCGGAAATG   127
trnV  TCGAGTTGCACGAACCTTAACTCTGTGTAAAAGAGTCGCTTACTCCCTAGCTAT--ATTCTG-TATTTATATAAATAC--------------------------------------------------    75
trnM  --------------------------------------------------------ATTCT--TATTTATATAAACACGTAAAGTA-AGCTAA-CT--T-AAGCTTTTAGGCTCATATCCCAAGAATG    65
                                           trnV
                                                                                                     trnM
```

```
        trnM
R     GAGGCGAAAATC-CC   141
trnV  ---------------    75
trnM  GGAGATAAAAACTCC    80
        trnM
```

2.4.3 trnC-trnV 11

```
                                           trnC                                                      trnV
R     TTCTAAGGTGTATAACATATTTAATTGCAAATTAAAAGT-CGTCC-A-GAAGGACTAG-AACCTA-AATTCAAGGAAAC-CT--GAGTTGCACAGGTATCAACTCCGTGTAAAAGAGACACTTTCTTC   120
trnC  GG-T--G-TATTTTACATATTTAATTGCAAATTAAAAG------------------------------------------------------------------------------------------    34
trnV  ---------------------------CCAACTAAAAGCACAACCCACTAAATACTAGTCTAGTACAAATCTTCAAAACAGTTCGAGTTGCACGAACCTTAACTCTGTGTAAAAGAGTCGCTTACTCC   101
                  trnC
                                                                                                     trnV
```

```
        trnV
R       TTA-    123
trnC    ----     34
trnV    CTAG    105
        trnV
```

2.4.4 trnP-trnQ 10

```
                                                      trnP                                                                   trnQ
R     -AGTTTAACAAAAATAGTTGTTTTGGGGACAACCGATATAAGGATCTT-CT-TGTGCTTCTGAGGTAAAA-AGTTATGACCTTCTATCAAGGGAATCAAAGACCCTCATTTTTCGTTAA-   115
trnP  TAGTTTATTAA-AATAGTTATTTTGGGGATAACAGATACA-G-ACCTAGCCCTGTGTTTCTGAAATAAA---------------------------------------------------    66
trnQ  -----------------------------------------------------------CTGAAA-AGATGAGAAACGACCTCATATCAAGGGTATCAAAATCCCTCATTTTTCATTAAA    60
                                                      trnP
                                                                                                 trnQ
```

2.4.5 trnQ-trnN 7

```
                                              trnQ                                                               trnN
R     AAGTTATGACCTTCTATCAAGGGAATCAAAGACCCTCATTTTTCGTTAAACTATACCTC-A--TGAGTTGAAGCTGA-ACTGTAGCATTTGGCCGTTAACCAAAAGGATGGAGGTTATTAACC   119
trnQ  GAGAAACGACCTCATATCAAGGGTATCAAAATCCCTCATTTTTCATTAAAATA----------------------------------------------------------------------    53
trnN  ----------------------------------------------TAAAACAAATCTTTAAATGGGTTGAAGCTGAAACAATAGCATTTGGCCGTTAACCAAAAGGATGAAGGTTAGTCCCT    77
                                          trnQ
                                                                                                      trnN
```

2.4.6 trnL1-trnA 5

```
R      AAGA-AGTTAATGCAGAAGGTTTAGGTCCTTCTATCAAGGGTGCAATTCCTTTTCTAGACTGTGGTTTCCAGGAAAT-TCCT-AGATCTCATGCGTGCAAAGCAAGCGTTTT-ATAATAAAC   118
trnL1  AAAATAGT-AATGCATTAGATTTAGGTTCTATTATTAAAGGTGCAAATCCTTTTTTAAACT-------------------------------------------------------------    60
trnA   --------------------------------------------------------AAACTGTGATTTCCA--AAATATCTTTGGATCATTTGCATGCAAAGCAAATATTTTTATAAACTAA    64
```

2.4.7 rrnS-trnE 5

```
R      -TCAAACGAGTAGAAAAGTCGTAACATAGTAGGTGTACCGGA-AGGT-GCCACCTGGAAAAACGCCCTTCTAGTTTACCTTAAAATAAAGGCTTTTCAAGCCTTAGAGCCAAGTGAAA-AACT--   119
rrnS   ATTAAA--ATTA-AACAC-AGAAACTTCCAAAGTCT-CAGTTCAGGTTTACACCTATAATAA---------------------------------------------------------------    57
trnE   ---------------------------------------------------------AAAAACTGC--TAAAGTTTAG-A-AAAACGAAGGCTTTTCAAGCCTTAGACCCAGGTTAAACCCCTGG    64
```

2.4.8 trnW-trnC 2

```
R      AAGTTAA-AAGACTGTAAGCCTTCAAAGCTTAAAGCGTAGGTTAAACTCCTACAGTCTTTGGGTTCTAAGGTGTATA--ACATATTTAATTGCAAATTAAAAGTCGTCCAGAAGGACT-AGAA   119
trnW   -AGTTAATAAAACTTTGAGCCTTCAAAGCTCACCACACAAATCAAAATTTTGTAATCTTTGGG------------------------------------------------------------    62
trnC   -------------------------------------------------------------GGTTCTAAGGTGTATTTTACATATTTAATTGCAAATTAAAAGTTA-CTAAAAAAATTTAGT-    60
```

2.4.9 trnR-nad4l 2

```
                             trnR                                                     nad4l
R      -TAGTTTAAAAAACATGAAGTTTCGGCCTTCAAATTCTTAGTTTAAATCTAAGATCT-TCCAATGAACATATTATCCGCTATCTTATTTTCATTATTTTTCTAGGAGTCATAGGAATAA-T   119
trnR   TTAGTTTAATAAACATAAAGCTTCGACCTTTAAATTCTTAGTA-AAGCCTAAGATCTTTTTAA-----------------------------------------------------------   62
nad4l  -------------------------------------------------------------AATGAACATACTAACAAGAATTTTATTCTCCCTTTTCTTCCTCGGGGTTTTAGGAGTGGTT   61
                            trnR                                                     nad4l
```

2.4.10 trnE-trnT -6

```
                              trnE                                                     trnT
R      --TTTACCTTAAAATAAAGGCTTTTCAAGCCTTAGAGCCAAGTGAAAAACTTGGGAAGAGTAGGCCTTAAAAGTTTACGAAAAACAGCTAGTCTTGTAAACTAGGAGAGAAGGTTTAACTC---C--   120
trnE   AGTTTA-G-AAAAACGAAGGCTTTTCAAGCCTTAGACCCAGGTTAAACCCCTGGTAGGAGT------------------------------------------------------------------   59
trnT   -------------------------------------------------------------------TATAAG--TAGGATACAAACTTAGTCTTGTAAACTAAGTTAGAAAGTTAAACTCTTTCAT   58
                             trnE                                                      trnT
```

2.4.11 trnT-trnP -422

```
                             trnT
R      TTTACGAAAAACAGCTAGTCTTGTAAACTAGGAGAGAAGGTTTAACTCCTTCTTAAGGCTTTTAGCCTCTTTTTTCCGTTCCCCCTACACCCCCTCCTACATCTATCTCCAACCGTATGCTCTTAATC   128
trnT   AGTAGGATACAAACTTAGTCTTGTAAACTAAGTTAGAAAGTTAAACTCTTTCATATAGCT--------------------------------------------------------------------   60
trnP   --------------------------------------------------------------------------------------------------------------------------------   0
                             trnT
```

```
R     ATAGTATTATTTCCACATTAACATATATTTCTTTTTTTACTCCTTATATTACTCTTAGCTTCCAAGTAATGGCATATCATAGTCTCTTCTAATCTTTCTTAATGTAGTAACAGCCTTCTCCCTTTAGG  256
trnT  --------------------------------------------------------------------------------------------------------------------------------  60
trnP  --------------------------------------------------------------------------------------------------------------------------------  0

R     GGTTGTCTTTTCAAGTTCGACTTTGGTAATTGTTATACTTCTTTATTTTGCTCTCATGTTAATTTCCTCAAGTTCTAAATATATTCACTACACTCTGTTTTAGCTGGGTTTGTCTCGGTAAGTTCCGC  384
trnT  --------------------------------------------------------------------------------------------------------------------------------  60
trnP  --------------------------------------------------------------------------------------------------------------------------------  0

                                                                                                                        trnP
R     TGGGCCAACATCATGTTTAAAATTATAGACGTTAAGTCTTTTCATTTATATAAATAGTTATATTTAAATATTAATAATTATCTAAGCTTTCCCTATTCAGAAGCTAGTTTAACAAAAATAGTTGTTTT  512
trnT  --------------------------------------------------------------------------------------------------------------------------------  60
trnP  --------------------------------------------------------------------------------------------------AGAAACTAGTTTATTAAAA-TAGTTATTTT  29
                                                                                                                        trnP

                  trnP
R     GGGGACAACCGATATA-AGG-ATCTTCT-TG  540
trnT  -------------------------------  60
trnP  GGGGATAACAGATACAGACCTAGC-CCTGTG  59
                  trnP
```

2.5 NC_015120-NC_008124

Avg ovsize: -47

LCA: Gadiformes-order

2.5.1 trnM-nad2 14

```
                    trnM                                                                     nad2
R     -GCTAAATTAAGCTCTCGGGCCCATACCCCGAACATGTTGGTTAAATTCCTTCCTCTGCTGCTATGA-GTCC-C-TTTGCCCTCTCTATTTTTTCATTA-A-GCCTCGGATTGGGCACTACCCTTAC-   121
trnM  AGCTAAA-TAAGCTTTTGGGCCCATACCCCAAACATGACGGGTAAAATCCTTC---------------------------------------------------------------------------   52
nad2  ---------------------------------------GATTAAATTC-TTCGACCCTTTCCAGAATGCCCGCGATTTCAATC-CTATTCTTT-ATTACAAGCTTAATTCTAGGAACTGCAATAACT   86
                    trnM
                                                                                             nad2
```

2.5.2 trnW-trnA 8

```
                    trnW                                                                     trnA
R     -GATGGACCAGACCAAGAGCCTTCAAAGCTCTCAGCGGAGGTGAGAACCTCCAGCCCCT--G---ATAAAATCTGCGGGATACTACCCCACATCTCCTGCATGCAAAACAGACACTTTAATTAAG-   120
trnW  GGATA-AGGAGACCAAGGGCCTTCAAAGCCTTTAGTGGAGGTTAAATCCTCCAGTTTCTGAGCGCATATTAATCT----------------------------------------------------   74
trnA  ------------------------------------------------------------------TAAAATCTGCGGGACATTACCCCACATCTTCTGCATGCAAAACAGACACTTTAATTAAGC   60
                    trnW
                                                                                             trnA
```

2.5.3 nad6-trnE 7

```
                    nad6                                                                     trnE
R     AGCTACTGAAATTACACCAACAATTATCCCAA-CT-AA--AAAA-GACAACATAAGATATGCCATAAATTCCTGCCCGGGCTCTCACCAGGACCAATGACTTGAAAAACCACCGTTGT-ATTCAAC   120
nad6  AACTACAGAAACCACCCCCAAAATTATTCCAATCAGAATTAAAATTAAAACATAAGACATCATTTTTATT--------------------------------------------------------   70
trnE  ---------------------------------------------------------------ATATATTTCTGCCCGGAATTTAACCGGAACCTACGACTTGAAAAACCGCCGTTGTAATTCAAC   63
                    nad6
                                                                                             trnE
```

2.5.4 trnI-trnQ 5

trnI *trnQ*

```
R     CTGAAT-TAAAGGACCACTTTGATAGAGTGAATCATAAGGGTTAAACTCCCTTCAGCTCCTTAGAAAAAGGGGATTTGAACCCATCCTTA-GGAGATCAAAACTCCTAGTGCTTCCACTA-   118
trnI  -TGAAACTAAAGGGCCACTTTGATGTAGTGGATCATGAGGGCTAAAATCCCTTCAACACC-TAAAA-------------------------------------------------------   64
trnQ  -------------------------------------------------------------TAGAAAAAAGGGATTTGAACCC-TCCCTATGGAACTCAAAATTCCATGTGCTACCTCTAC   59
```

trnI *trnQ*

2.5.5 trnL2-nad1 1

trnL2 *nad1*

```
R      GCCCGGACAGTGCATGAGGCCTAAGCCCTCCCTACAGAGGTTCAATTCCTCTCTCTAACTATGATCAACACCTTGATCACCCATGTTCTCAACCCCCTCATCTATATGGTCCC-AGTA--CT-   119
trnL2  AACAGGTTAATGCGTAAGACCTAAGCCCTTAATAAAGAGGTTCAAGTCCTCTCTCTAA-TA--------------------------------------------------------------   60
nad1   ------------------------------------------------------------ATGATC-AC-CCTA-CTAAACTATGTAATTATACCACTAACCTTTATGGTACCTATTCTTCTT   60
```

trnL2 *nad1*

2.5.6 trnH-trnS1 1

trnH *trnS1*

```
R      GTTTAATGAAG-ACATTAGATTGTGATTCTAAAAATAGAGGTTAAACCCCTCTAACCCACCGAGGAAGGCCCG-A-TGGCAATGAAGACTGCTAATTATCACCCCCTTGGTTAAACCCCAAG   119
trnH   -TTTAACGAAAAACAACAGATTGTGATTCTGTAAACAGAGGTTCAACCCCTCTAATTCACCG------------------------------------------------------------   61
trnS1  -------------------------------------------------------------GAAAAAGGGCCGGACTGGCAATAAAAGCTGCTACTTTTTAATACTTTGGTTTGACTCC-AG   60
```

trnH *trnS1*

2.5.7 trnC-trnY 1

```
                                    trnC                                                  trnY
R      AGTCCCGGTAGGCGTTAGCCTACTTCTTC-AGATTTGCAATCTGACGTG-TGACACCCCAGGGCTTACG-GACTTGACAGGAAGAGGGCTCAAACCTCTGTATGTGGGGTTACAATCCACCGCTTA-C   124
trnC   TGA-CAG--A-GCTTTAATCTGTGT-TTCTAGATTTGCAATCTAACGTGATAATACTCCAAGACTT--------------------------------------------------------------   61
trnY   -----------------------------------------------------------------TAAGTGAAGTGATAGGAAGAGGGGTTGAACCTCTGTCTGTGGAGCTACAATCCACCGCCTAAC   63
                                    trnC
                                                                                                  trnY
```

```
       trnY
R      TCAGCC   130
trnC   ------   61
trnY   TCAGCC   69
       trnY
```

2.5.8 trnS1-trnL1 0

```
                                     trnS1                                                 trnL1
R       CCCGA-TGGCAATGAAGACTGCTAATTATCACCCCCTTGGTTAAACCCCAAGGCTCCCTCGAGCTCCTAAAGGATAATAGCTCATCCGTTGGTCTTAGGAACCAAAAACTCTTGGTGCAA--C   120
trnS1   CG-GACTGGCAATAAAAGCTGCTACTTTTTAATACTTTGGTTTGACTCCAGGGCT--------------------------------------------------------------------   54
trnL1   -------------------------------------------------------CCCACG-GCTTCTAAAGGATAAAAG-TCTTCCGCTGGTCTTAGGAACCCGTAATTCCTGGTGCAAATC   66
                                  trnS1
                                                                                            trnL1
```

2.5.9 nad1-trnI -5

```
                                 nad1                                                                  trnI
R      CTAGTGATTTGGCACCTCTCACTCACCACT-GCATTCGCCGGGCTCCCACCCCAAGCCTAGCAACGGAGTTGTGCCTG-AATTAAAGGACCACTTTGATAGAGTGAATCATAAGGGTTAAACTCC  123
nad1   TTTATAATCTGACATCTGTCAATTTCTATTAGCCTAAGT-GGCCTCCCACCTCAGATCTA-----------------------------------------------------------------   59
trnI   -----------------------------------------------------------------GGTGCTGTGCCTGAAACTAAAGGGCCACTTTGATGTAGTGGATCATGAGGGCTAAAATCC   60
                                nad1
                                                                                                       trnI
```

2.5.10 rrnL-trnL2 -27

```
                                 rrnL                                                       trnL2
R      CCACTGACACCTAATAAAGTGGAA-AAGGGGGCTTAAAAAGGCCCCAGAAAATATGGGCATGTTACAGTGGCAGAGCCCGGACAGTGCATGAGGCCTAAGCCCTCCCTACAGAGGTTCAA--  119
rrnL   TTAAATTTACCTAG-AAGGTAGCAGAAGTTGGC-----------------------------------------------------------------------------------------   32
trnL2  ------------------------------------------------------------TATTAGAGTGGCAGAACA-GGTTAATGCGTAAGACCTAAGCCCTTAATAAAGAGGTTCAAGT   61
                    rrnL
                                                                                            trnL2
```

2.5.11 trnT-trnP -529

```
                                trnT
R      GCACAACAGAGCGCCGGCCTTGTAAGTCGGAGGTCGGAGGCTAAATTCCTCCCTAGCGCTAAACACGAGCAGCACAATTGCCTCCGAGCTCTGCCCAACCCACTTTCCTACAAAACCTTAAGAGTCTT  128
trnT   GTAATTCAGAGCACCAGTCTTGTAAACTGGGGGTCAAAGGTTAAACTCCTTTCTAGTGCTAA------------------------------------------------------------------   62
trnP   --------------------------------------------------------------------------------------------------------------------------------    0
                                trnT
```

```
R      AACCCCGCAGGTCCCAAACCCCGAGCTCTGCCCAACTACTTTTCCTGCAAAAGGTCCCGACTAAAAAATCCTACCACAGACCCCCCCCCTTTTTAAACCCGCCCTGCCCAGCCCACTTTCGTCAGGAA   256
trnT   --------------------------------------------------------------------------------------------------------------------------------    62
trnP   --------------------------------------------------------------------------------------------------------------------------------     0

R      GACCCGGACCCAAAAAAATCTTCATCTTTCCCCCCCCCCCTTTTTTTAACCCCCCCCCCCCCCCAAAGCCGGACCCAAAAAAATCTTCATCTTTCCCCCCCCCCCTTTTTTTAACCCCCCCCCAAAGC   384
trnT   --------------------------------------------------------------------------------------------------------------------------------    62
trnP   --------------------------------------------------------------------------------------------------------------------------------     0

R      CGGACCCAAAAAATCTTCATCCTTTTTTAACCCCCCCCTTTTTTACCCGCTTACCCGCTTACCCGCTTACCCGCTTACCCGCTTACCCGCTTACCCGCTTACCCGCTTACCCGCTTACCCGCTTACCC   512
trnT   --------------------------------------------------------------------------------------------------------------------------------    62
trnP   --------------------------------------------------------------------------------------------------------------------------------     0

                                                                                                                 trnP
R      GCTTACCCGCTTACCCGCTTACCCGCTTACCCGCTTACCCGCTTACCCGCTTACCCGCTTACCCGCTTACCCGCTTACCCTCAGAAAGAAGAGACTTAAACTCCCATCACTAACTCCCAAAGCTAGCA   640
trnT   --------------------------------------------------------------------------------------------------------------------------------    62
trnP   -------------------------------------------------------------------------------CTCAGAAAGAGGAGATTTTAACTCCCACCCCTGGTTCCCAAAACCAGAA    49
                                                                                                                 trnP

          trnP
R      TTTTATTTTAAA   652
trnT   ------------    62
trnP   TTCTAAATTTAA    61
          trnP
```

3 Inversion

3.1 NC_023223-NC_023222

Avg ovsize: 4

LCA: Bagridae-family

3.1.1 trnH-trnS1 6

```
                                          trnH                                                          trnS1
R      TTTAACACAAAACATTAGATTGTGGTTCTAAAAATAGAGGTTAAATTCCTCTTATCCA-CCGAAAGAGGCCCGA-GGCAGTAAGGACTGCTAATCCTTATAACCACAGTTAAACTCCGTGG   119
trnH   TTTAACACAAAACATTAGATTGTGGTTCTAAAAATAGAGGTTAAACTCCTCTTATCCA-CC------------------------------------------------------------    60
trnS1  -------------------------------------------------------CCAGCCGAAAGAGGCCCGACGGCAGTAAGGATTGCTAATCCCTATTTCCATGGTTAAACTCCATGG    66
                                          trnH                                                          trnS1
```

3.1.2 trnS1-trnL1 2

```
                                  trnS1                                                       trnL1
R      -C-GA-GGCAGTAAGGACTGCTAATCCTTATAACCACAGTTAAACTCCGTGGCTCATTCACCCAGCTTCTAAAGGATAATAGTTCATCCGTTGGTCTTAGGAACCAAAAACTCTTGGTGCAAC-   120
trnS1  CCCGACGGCAGTAAGGATTGCTAATCCCTATTTCCATGGTTAAACTCCATGGCTCATTCA----------------------------------------------------------------    60
trnL1  ----------------------------------------------------------CAGCC-GCTTCTAAAGGATAATAGTTCATCCGTTGGTCTTAGGAACCAAAAACTCTTGGTGCAACT    65
                                  trnS1                                                       trnL1
```

3.2 NC_006893-NC_013571

Avg ovsize: 2

LCA: notFound-notFound

3.2.1 trnI-trnQ 3

```
                                   trnI                                                      trnQ
R     TCTGACAAAAGAGTTACTTTGATAGAGTAAATCATAGAGGTTTAAATCCTCTTATTTCTAGAGCTATAGGAATTGAACCTACCCTTGAGAAATCAAAAATCTCCGTGCTACCATTT-   116
trnI  TCTGATAAAAGAGTTACTTTGATAGAGTAAATAATAGAGGTTTAAGCCCTCTTATTTCTA---------------------------------------------------------    60
trnQ  ---------------------------------------------------------CTAGAACAATAGGAATCGAACCTATACCTGAGAATCCAAAGTTCTCCGTGCTACCCGTTA    60
                                   trnI
                                                                                            trnQ
```

3.2.2 trnQ-trnM 1

```
                                   trnQ                                                          trnM
R     -ATT-GAACCTACCCTTGAGAAATCAAAAATCTCCGTGCTACCATTTTTACACTAAACTCTAGTAAGGTAAGCTAATTTAAGCTATCGGGCCCATACCCCGAAAATGTCGG-TTAATACC-T   118
trnQ  GAATCGAACCTATACCTGAGAATCCAAAGTTCTCCGTGCTACCCGT-T-ACACCCCATTCTA------------------------------------------------------------    60
trnM  -------------------------------------------------------------AGTAAGGTCAGCTAATA-AAGCTATCGGGCCCATACCCCGAAAATGTTGGTTTAAACCCTT    60
                                   trnQ
                                                                                                 trnM
```

3.3 NC_006081-NC_012463

Avg ovsize: 2

LCA: Pancrustacea-subphylum

3.3.1 rrnL-trnV 9

```
                   rrnL                                              trnV
R      TTTATAAACTAACTAAAACTTAATAGATAAGTTTTTATT-TCAAAATAAATCGATTCGCACGAT-ATCTTTTCAACGTAAGTGA-AATGCTTAACTAGTCAAGCTCTATTTTG   110
rrnL   A--ATAAAA-AATTATTTTTTATAAAATAA-TTAAAATAATTAAAATAA----------------------------------------------------------------    45
trnV   ----------------------------------------TCAAATTAAATTGATTCGCACAATTAAATTATTAATGTAAATAATAATACTTA-CTA---AA-----A----G    60
                 rrnL
                                                                        trnV
```

3.3.2 trnL1-rrnL -5

```
                      trnL1                                                                  rrnL
R      TAAGAATTCAAACTCT-TATTTTTAGATCCTAAGTCTAATGCACTTTTCTGCCAACTTAACTAGTTAATAATAATCAACTAATAAACAAAATATTTGACTTATCAAATCCTTTCGTACT---A----   119
trnL1  TTTGTAGT-AAATACTACATAAATAAATTCTAAATTTATCACAATTTTCTGCCAAAATAGC------------------------------------------------------------------    60
rrnL   ------------------------------------------------------------------AATATTAATCAA-AAATTAA-AAATTATTTTAGGTACCTGGCCCTTTCGTACTGAGATACC    59
                      trnL1
                                                                                                 rrnL
```

3.4 NC_013836-NC_014703

Avg ovsize: 1

LCA: Cervus-genus

3.4.1 trnW-trnA 2

```
           trnW                                                          trnA
R     AGGTTAACCTAGACCAAGAGCCTTCAAAGCCCTAAGCAAGTACAATATACTTAACTCCTGATTAAGGATTGCAAGACCATATCTTACATCAA-TGAATGCAAATCAACCACTTTAATTAAGC 121
trnW  AGGTTAACCTAGACCAAGAGCCTTCAAAGCCCTAAGCAAGTACAATATACTTAACTCCTGAT------------------------------------------------------------  62
trnA  ------------------------------------------------------------AT-AAGGATTGCAAGACCATATCTTACATCAATTGAATGCAAATCAACCACTTTAATTAAGC  61
           trnW                                                          trnA
```

3.4.2 trnA-trnN 1

```
           trnA                                                          trnN
R     GCAAGACCATATCTTACATCAAT-GAATGCAAATCAACCACTTTAATTAAGCTAAATCCTCACTAGATTGGTGGGCTCCACCCCCACGAAACTTTAGTTAACAGCTAAACACCCTAATCAA- 120
trnA  -CAAGACCATATCTTACATCAATTGAATGCAAATCAACCACTTTAATTAAGCTAAATCCTCA------------------------------------------------------------  61
trnN  -------------------------------------------------------------ACTAGATTGGTGGGCTCCACCCCCACGAAACTTTAGTTAACAGCTAAACACCCTAATCAAC  61
           trnA                                                          trnN
```

3.5 NC_014347-NC_012571

Avg ovsize: 0

LCA: Panonychus-genus

3.5.1 trnK-cox2 1

```
                 trnK                                             cox2
R     TAAAAGAAAATTTTTCTATAGGTAACTTAAGAGGTTACAGCTT-TAATCAGCCATTTAACGTTTAATTTTTTAATCTAAAAACACTGATTGGTATAAATCTATGATTAATTCCACAAATTT-   120
trnK  --AAAGAAATATTTTTCTGTAAATAACTTAAGAGGTTACTGCTTCTACTCAGCCATTTAACGAT-----------------------------------------------------------    61
cox2  --------------------------------------------------------------TTAATTTTTTAATCTTAATACTCTAATAGGTATAAAACTATGATTAATACCACAAATTTC    60
                 trnK                                             cox2
```

3.5.2 atp8-trnK -1

```
                 atp8                                             trnK
R     TAAACTTAAAATTAAATAAAATAAAAATATTATTCAATTCATAGGAGAAACTTGATAAATTCGTTAAAAGAAAATTTTTCTATAGGTAACTTAAGAGGTTACAGCTT-TAATCAGCCATT-   119
atp8  TAAACTTAAAATAATAAATAATATGAATAATAACCAGTTTATAGGAGAAATTTGATATATT------------------------------------------------------------    61
trnK  --------------------------------------------------------------GTTAAAAGAAATATTTTTCTGTAAATAACTTAAGAGGTTACTGCTTCTACTCAGCCATTT    59
                 atp8                                             trnK
```

3.6 NC_023222-NC_023223

Avg ovsize: -52

LCA: Bagridae-family

3.6.1 trnH-trnS1 -51

```
                                    trnH                                                              trnS1
R      TTTAACACAAAACATTAGATTGTGGTTCTAAAAATAGAGGTTAAACTCCTCTTATCCACCGAAAGAGGCCCGACGGCAGTAAGGATTGCTAATCCCTATTTCCATGGTTAAACTCCAT-G   119
trnH   TTTAACACAAAACATTAGATTGTGGTTCTAAAAATAGAGGTTAAATTCCTCTTATCCACC------------------------------------------------------------    60
trnS1  ---------------------------------------------------------------------------------------------------------------ACTGCCTCG     9
                                    trnH
                                                                                                                      trnS1
```

3.6.2 trnS1-trnL1 -54

```
                                    trnS1                                                                 trnL1
R      CCCGACGGCAGTAAGGATTGCTAATCCCTATTTCCATGGTTAAACTCCATGGCTCATTCACCAGCCGCTTCTAAAGGATAATAGTTCATCCGTTGGTCTTAGGAACCAAAAACTCTTGGTGCAAC-   125
trnS1  GCC-ACGG-AGT------------------------------------------------------------------------------------------------------------------    10
trnL1  ------------------------------------------------------------------GCTTCTAAAGGATAATAGTTCATCCGTTGGTCTTAGGAACCAAAAACTCTTGGTGCAACT    60
            trnS1
                                                                                                   trnL1
```

3.7 NC_013571-NC_006893

Avg ovsize: -56

LCA: notFound-notFound

3.7.1 trnI-trnQ -55

```
R     TCTGATAAAAGAGTTACTTTGATAGAGTAAATAATAGAGGTTTAAGCCCTCTTATTTCTAGAACAATAGGAATCGAACCTATACCTGAGAATCCAAAGTTCTCCGTGCTACCCGTT   116
trnI  TCTGACAAAAGAGTTACTTTGATAGAGTAAATCATAGAGGTTTAAATCCTCTTATTTCTA--------------------------------------------------------    60
trnQ  -------------------------------------------------------------------------------------------------------------------T     1
```

3.7.2 trnQ-trnM -58

```
R     GAATCGAACCTATACCTGAGAATCCAAAGTTCTCCGTGCTACCCGTTACACCCCATTCTAAGTAAGGTCAGCTAATA-AAGCTATCGGGCCCATACCCCGAAAATGTTGGTTTAAACCCT   119
trnQ  AAA---------------------------------------------------------------------------------------------------------------------     3
trnM  -------------------------------------------------------------GTAAGGTAAGCTAATTTAAGCTATCGGGCCCATACCCCGAAAATGTCGGTTAATACCTT    59
```

3.8 NC_012571-NC_014347

Avg ovsize: -58

LCA: Panonychus-genus

3.8.1 trnK-cox2 -57

```
     trnK (R 1-60)                                                cox2 (R 61-119)
R    AAAGAAATATTTTCTGTAAATAACTTAAGAGGTTACTGCTTCTACTCAGCCATTTAACGATTAATTTTTTAATCTTAATACTCTAATAGGTATAAAACTATGATTAATACCACAAATTT-   119
trnK AAA---------------------------------------------------------------------------------------------------------------------     3
cox2 ------------------------------------------------------------TTAATTTTTTAATCTAAAAACACTGATTGGTATAAATCTATGATTAATTCCACAAATTTC    60
     trnK (1-3)                                                   cox2 (61-120)
```

3.8.2 atp8-trnK -60

```
     atp8 (R 1-60)                                                trnK (R 61-120)
R    TAAACTTAAAATAATAAATAATATGAATAATAACCAGTTTATAGGAGAAATTTGATATATTCGTTAAAAGAAATATTTTCTGTAAATAACTTAAGAGGTTACTGCTTCTACTCAGCCATT   120
atp8 TAAACTTAAAATTAAATAAAATAAAAATATTATTCAATTCATAGGAGAAACTTGATAAAT------------------------------------------------------------    60
trnK ------------------------------------------------------------------------------------------------------------------------     0
     atp8 (1-60)
```

3.9 NC_014703-NC_013836

Avg ovsize: -59

LCA: Cervus-genus

3.9.1 trnW-trnA -59

```
                                  trnW                                                      trnA
R     AGGTTAACCTAGACCAAGAGCCTTCAAAGCCCTAAGCAAGTACAATATACTTAACTCCTGATTAAGGATTGCAAGACCATATCTTACATCAATTGAATGCAAATCAACCACTTTAATTAAG  121
trnW  AGGTTAACCTAGACCAAGAGCCTTCAAAGCCCTAAGCAAGTACAATATACTTAACTCCTGAT-----------------------------------------------------------  62
trnA  -------------------------------------------------------------------------------------------------------------------------  0
                                  trnW
```

3.9.2 trnA-trnN -60

```
                                  trnA                                                      trnN
R     CAAGACCATATCTTACATCAATTGAATGCAAATCAACCACTTTAATTAAGCTAAATCCTCACTAGATTGGTGGGCTCCACCCCCACGAAACTTTAGTTAACAGCTAAACACCCTAATCAA-  120
trnA  -------------------------------------------------------------------------------------------------------------------------  0
trnN  ------------------------------------------------------------ACTAGATTGGTGGGCTCCACCCCCACGAAACTTTAGTTAACAGCTAAACACCCTAATCAAC  61
                                                                                            trnN
```

3.10 NC_012463-NC_006081

Avg ovsize: -60

LCA: Pancrustacea-subphylum

3.10.1 trnL1-rrnL -59

```
                                  trnL1                                                     rrnL
R      TTTGTAGTAAATACT-ACATAAATAAATTCTAAATTTATCACAATTTTCTGCCAAAATAGCTAATATTAATCAAAAATTAAAAATTATTTTAGGTACCTGGCCCTTTCGTACTGAGATAC  119
trnL1  TAAGAATTCAA-ACTCTTATTTTTAGATCCTAAGTCTAATGCACTTTTCTGCCAACTTAAC-----------------------------------------------------------  60
rrnL   ------------------------------------------------------------------------------------------------------------------------  0
                                  trnL1
```

3.10.2 rrnL-trnV -61

```
R     --AATAAAAAATTATTTTTTA--TAAAATAATTAAAATAATTAAAATAATAATTTAATAATTTATCAAATTAAATTGATTCGCACAATTAAATTATTAATGTAAATAATAATACTTACTAAAA  119
rrnL  TTAGTACGAAAGGATTTGATAAGTCAAATATTTTGTTT-ATTAGT-TGATTA-TTATTAACT-------------------------------------------------------------   59
trnV  ---------------------------------------------------------------------------------------------------------------------------    0
```

3.11 NC_006922-NC_009683

Avg ovsize: -399

LCA: Agamidae-family

3.11.1 trnt-trnp 3

```
R     TAGCTTAAACACC--ATAGAGCGTTAGTTTTGTAAACTAAAGACGGGAACACCCGCAGATCATCAAAAGAGGACTATAAGCCCGATCCCTGGCCCCCAAAGCCAACATTTTAACTAAACTA---  119
trnt  --GCTTAAGCTCCTAATAAAGCATTAGACTTGTAAAGTAAAGACGGGA-CACCC-CTAA-GATCA-----------------------------------------------------------   60
trnp  --------------------------------------------------------------TCAAAAGAAGGTGGTGA-CC-TATTGCTGGCATCCAAAGCCAGTGTTTTGTTTAAACTATCT   60
```

3.11.2 trnP-trnF -802

```
                                            trnP
R     -GAGGACTATAAGCCCGATCCCTGGCCCCCAAAGCCAACATTTTAACTAAACTATCTTTTGTTTTTCACCTGACTAAATATTACTAATATGCACCAAAATAAATATTCCCCAGGCTCACCCCCATTCA   127
trnP  AGA-TAGTTTAAACA-AAACACTGGCTTTGGATGCCAGCAATAGGTCACCACCTTCTTTTG-------------------------------------------------------------------    59
trnF  --------------------------------------------------------------------------------------------------------------------------------     0
                                            trnP

R     TTTACTATCGTCCGGTACCCCCCCTACCCCCCCCATTGCTGAAAGAGTAAAAAATCACACATACGTATATGTATAATCCCCATTATTTAGTACTAAATAATGTAACCTTATGCCCTATGTATATCGTGC   255
trnP  --------------------------------------------------------------------------------------------------------------------------------    59
trnF  --------------------------------------------------------------------------------------------------------------------------------     0

R     ATTAATTTTTTTGCCCCATGCATATCATCATAGACATTCCCCTCTTAATCATACTAACTACATGTCATTATATAAGGTACAGTATACCCTTCACCCCATGGATATCAAGCATGCACATATCTGCTTAA   383
trnP  --------------------------------------------------------------------------------------------------------------------------------    59
trnF  --------------------------------------------------------------------------------------------------------------------------------     0

R     TCTGACATAACCCTTGCCCAACCAATCAACATTCTATATGTACCCCTCCGCGAGAGATCAGCAATCCTTGCATGAAAGGTTTATCATGTCTAGCTTCAGGCTCCTATATCGAGGTTGCATCACAACTG   511
trnP  --------------------------------------------------------------------------------------------------------------------------------    59
trnF  --------------------------------------------------------------------------------------------------------------------------------     0

R     TACTTTCCAAGGCCTCTGGTTGTTACTTCAGGCACATCACATCCAACTCCGCCCACGGTTATCTTTCCAAGACCTATGGTTGATGGTGTATTACATCTCACCCATGACCCCCCATTCCTTGCCCTTCC   639
trnP  --------------------------------------------------------------------------------------------------------------------------------    59
trnF  --------------------------------------------------------------------------------------------------------------------------------     0

R     GGCATTTGGTACTTTTTATCTATTTTCAACTCTCTGTACATTTCAAGTGCCCAGCAATTACAGTTGCACCTAGTCCATATATTAAAGTGTGTACCCCGCTATATATCCCATGTATTACAATCTTTTAA   767
trnP  --------------------------------------------------------------------------------------------------------------------------------    59
trnF  --------------------------------------------------------------------------------------------------------------------------------     0
```

```
                                                                                                                trnF
R     TGCTCGTAGGACATAAAACTTATAAAAAACAACCAATTTGACCAGAAATACCGAGAACAACAAACATTCATCAAACATATGTTGTAACCCCGTCATTGTAGCTTACCACCAAAGCATAGTGCTGAAGA   895
trnP  --------------------------------------------------------------------------------------------------------------------------------   59
trnF  -----------------------------------------------------------------------------------------------TTGTAGCTTAATTT-AAAGCCTAGTGTTGAAAA   32
                                                                                                                  trnF
```

```
        trnF
R     CACTAAGACGAGCCTTA--CTCCA   917
trnP  ------------------------   59
trnF  CACTAAAACGAGCCAAACGCTCCA   56
        trnF
```

3.12 NC_023228-NC_015076

Avg ovsize: -456

LCA: Clupeocephala-order

3.12.1 trnQ-trnI -9

```
                                   trnQ                                                                     trnI
R     TTATGGAGTGCACAAAGAGTTTTGATCTCTTA-AGTTGAGGTTCGAATCCTC-CTTTCCTATATATTATCCCCAGGAGCTATGTCTGA---ATAAAGAGTCACTTTGATAGAGTGAAGCACGTAGATG   123
trnQ  GTAGCGGGAGCACTCAGAGTTTTGATCTCTGCGGGCTG-GGTTCAAATCCCGGCTTT-CTAAAGA---------------------------------------------------------------   63
trnI  --------------------------------------------------------------------------GGAACTGTGCCTGACCGCTTAAGGGGCACTTTGATAGAGTGACTAAAGGGGGT-   53
                                   trnQ
                                                                                                         trnI
```

```
        trnI
R     AAAACCCT   131
trnQ  --------    63
trnI  TGAACTC-    60
        trnI
```

3.12.2 nad1-trnQ -904

```
      nad1
R     TTCGTGATAGTAAC-CCTAATAATTC-TAGTTATAACAGCAGGTATTCCTCCAATAGACTAATATTTTACATATATGTAATATTTTACATATATGTAATATTTTACATATATGTAATATTTTGCATAT   126
nad1  TTCGTCGT-CTGACACCT-TTCCTTCATTGTCAGCACTGCCGCACTTCCCCC----------------------------------------------------------------------------    50
trnQ  --------------------------------------------------------------------------------------------------------------------------------     0
      nad1

R     ATGTAATATTTTACATATATGTAATATTTTACATATATGTAATATTTTACATATATGTAATATTTTACATATATGTAATATTTTACATGTATGTAATATTTTACATGTATGTAATAGAACATAATACA   254
nad1  --------------------------------------------------------------------------------------------------------------------------------    50
trnQ  --------------------------------------------------------------------------------------------------------------------------------     0

R     TTCCACTCATAAACTTACTAATAGATGTATTAAACGCCCATAGGTATATCATATATTATATATCAGTATATCACCTACATAGTAAATTAATAACCATACATTGGATTTCAAGAATCAAGCTCCTTACA   382
nad1  --------------------------------------------------------------------------------------------------------------------------------    50
trnQ  --------------------------------------------------------------------------------------------------------------------------------     0

R     TTCTCAAGATCTTATCTTGAGAGAATAGTATTCTCTCTCTACTTTATGCTAGTATGCGGGATCCCATCATGTACACCTCCTACTTATCTAGTCAGTTCTTATTTTATAACATACCTTGCATGCTGTTA   510
nad1  --------------------------------------------------------------------------------------------------------------------------------    50
trnQ  --------------------------------------------------------------------------------------------------------------------------------     0

R     ATAAAGTTCTACACATACTTGACATGAAGTGTTCTCCAGGGGATAAGGGGTTTTCCTCTCTCCCTTGTTGACTCGACTAGTGTCTGCTTAGAAGAATATATCTGGGTAACTCATAACCAATTTCATCT   638
nad1  --------------------------------------------------------------------------------------------------------------------------------    50
trnQ  --------------------------------------------------------------------------------------------------------------------------------     0
```

```
                                                                                                                                  
R     ATTACTACAAAAGACATACAGACATTTATTGGAACACTACATAAGTCCTATAAACATACATTTTTACTGTCTTTATATCCGCCCGCCTTAATACAAACTATAGTCCTATACACAGTAATAGAAATCCC   766
nad1  --------------------------------------------------------------------------------------------------------------------------------   50
trnQ  --------------------------------------------------------------------------------------------------------------------------------   0

R     GGACTCTTTAATTTACCTCAGTGATTTCGACATAAAAAATTTTCCATCTTACATCTCTCAAAGAAGCAACAAATATTAAACAAAATTTACTAAGCCTAAAAATTTTTTGAGATTATTCGATTATAAAA   894
nad1  --------------------------------------------------------------------------------------------------------------------------------   50
trnQ  --------------------------------------------------------------------------------------------------------------------------------   0

                                                                                      trnQ
R     AAATCAAAAAACAAGCTACTAAAATATCACAAACCAAGTAACTTTGAAAATTTAGGAAATAGTGTTAT-G-GAGTGCACAAAGAGTTTTGATCTCTTAAGT-TGAGGTTCGAAT--   1005
nad1  --------------------------------------------------------------------------------------------------------------------   50
trnQ  ------------------------------------------------------------AGTGGTGTAGCGGGAGCACTCAGAGTTTTGATCTCTGCGGGCTG-GGTTCAAATCC   55
                                                                                        trnQ
```

3.13 NC_015076-NC_023228-revcomp

Avg ovsize: -467

LCA: Clupeocephala-order

3.13.1 nad1-trnq -12

```
                              nad1                                                             trnq
R     TTCGTCGTC-TGACACCTT-TCCTTCATTGTCAGCACTGCCGCACTTCCCCCTCAATTTAGGAGATTA-GAAAGCCGGGATTTGAACC-CAGCCCGCAGAGATCAAAACTCTGAGTGCTCCCGCTA-   122
nad1  TTCGTGATAGTAAC-CCTAATAATTC-TAGTTATAACAGCAGGTATTCCTCC---------------------------------------------------------------------------   50
trnq  ----------------------------------------------------------------AATAGGAAAGG-AGGATTCGAACCTCAACTT-AAGAGATCAAAACTCTTTGTGCACTCCATAA   61
                          nad1
                                                                                                trnq
```

3.13.2 trnq-trni -923

```
                                                  trnq
R     GGATTTGAACC-CAGCCCGCAGAGATCAAAACTCTGAGTGCTCCCGCTA-CACCACTCTCTGAATTGTTACCGCCAGGCTCTGCCCCGCCCGGACGTCCTCCCTTTCTGGTGATTAATATATACTATG 126
trnq  -GATTCGAACCTCAACTTA-AGAGATCAAAACTCTTTGTGCACTCCATAACACTATTTCCT------------------------------------------------------------------- 59
trni  -------------------------------------------------------------------------------------------------------------------------------- 0
                                                  trnq

R     TATTTACACCATAAATTTATTTCAGGTACTATCAAGAAACCATTAATACTAAGAAACACCAGGTACCCTATAAGATTTATCAACTTAGTGTGGAATAAATGAATATTTGATCATTAATCCCCATAATC 254
trnq  -------------------------------------------------------------------------------------------------------------------------------- 59
trni  -------------------------------------------------------------------------------------------------------------------------------- 0

R     TTATATGAATGCATATTACTCTCTATCAACATCCCTAATTCTACAAATATAATGCGCAGTAAGAAATCAGCAACCCATATCTAATTGCATATCATGAATGATAGGGTCAGGGACTTACGGTGTGGGGG 382
trnq  -------------------------------------------------------------------------------------------------------------------------------- 59
trni  -------------------------------------------------------------------------------------------------------------------------------- 0

R     TTACACAGAATGAACTATTACTGGCATCTGGTTCCTATTTCAGGGCCATACTAGTATATTCCCCCATCAACTGAATTAAATTTGCATAAGTTAATGGTGTAGGACTAACGGTTCTTTACTCCCCATGC 510
trnq  -------------------------------------------------------------------------------------------------------------------------------- 59
trni  -------------------------------------------------------------------------------------------------------------------------------- 0

R     CAAGCGTTCTCTATGCGACATCTGGTTCTTTTTATTTCCGGGTCACTTTCAAATTGCATTTGGCGACTCCTTCCTAATGTTATTAGCGAAGGTGGCACTACACTTTGCCTGAGTAAACAACTGTAACA 638
trnq  -------------------------------------------------------------------------------------------------------------------------------- 59
trni  -------------------------------------------------------------------------------------------------------------------------------- 0

R     CTCTATAGACTAAAATAGAAGACTTGCATAAGTAATATCAAGTACATAAGGTTATTCCTTAATCATCTATCTACTCGTAGAATGCCCCGGGGTGTATGGTTAAAGGAATTTGCTCGGCAAACCCCCCC 766
trnq  -------------------------------------------------------------------------------------------------------------------------------- 59
trni  -------------------------------------------------------------------------------------------------------------------------------- 0

R     TACCCCCCCTTAATCCGAAAGAACTTTGTTGTCTCCTGTCAAACCCCTAAACCAGGAAAAATTCAGACAGATCGTCACTGGGTTAAAATGTGAAATGAAGGTATGTGAGTATAATAATATAAAAAAT 894
trnq  -------------------------------------------------------------------------------------------------------------------------------- 59
trni  -------------------------------------------------------------------------------------------------------------------------------- 0
```

```
                                                                                                                          trni
R     TCATGCCCAAAGCCCCCTCTTTACCTGCAATTCTTCGGCCGAACCCCCCCCATTTTCCCCCTTGGGCCGAAGGATTCTTGTTTAAGGCGGAACTGTGCCTGACCGCTTAAGGGGCACTTTGATAGAGT 1022
trnq  -------------------------------------------------------------------------------------------------------------------------------- 59
trni  ----------------------------------------------------------------------------------------GGAGCTATGTCTGAA---TAAGAGTCACTTTGATAGAGT 37
                                                                                                                      trni
```

```
            trni
R     GACTAAAGGGGGTTGA-A--CT- 1041
trnq  ----------------------- 59
trni  GAAGCACGTAGATGAAAACCCTA 60
            trni
```

3.14 NC_009683-NC_006922

Avg ovsize: -753

LCA: Agamidae-family

3.14.1 trnT-trnP 2

```
                         trnT                                                      trnP
R     --GCTTAAGCTCCTAATAAAGCATTAGACTTGTAAAGTAAAGACGGGA-CACCCCTAAGATCAAAAGATAGTTTAAACAAAACACTGGCTTTGGATGCCAGCAATAGGTCACCACCTTCT- 117
trnT  TAGCTTAAACACC--ATAGAGCGTTAGTTTTGTAAACTAAAGACGGGAACACCCGCA-GATCA---------------------------------------------------------- 60
trnP  -------------------------------------------------------------CAAAAGATAGTTTAGTTAAAATGTTGGCTTTGGGGCCAGGGATCGGGCTTATAGTCCTC 60
                         trnT
                                                                                         trnP
```

3.14.2 trnP-trnF -1508

trnP

```
R     AGATAGTTTAAACAAAACACTGGCTTTGGATGCCAGCAATAGGTCACC-AC-CTTCTTTTGAAAAAAGCAAAAAAAAACACAAAAACAAAACAGGCCTTAATGTCTGTTTTGTTTTATGTGTATATTAC   126
trnP  A--TAGTTTAGTTAAAATGTTGGCTTTGGGGGCCAGGGATCGGGCTTATAGTCCTCTTTTGA------------------------------------------------------------------    60
trnF  --------------------------------------------------------------------------------------------------------------------------------     0
```

trnP

```
R     ATAAAATTATTTACCACATGAATATTTATGTAATACATCAATATATATCTAATACATTAATACATATATATATATATAATACATTAGTACATATATGTCTATAGTACATTATTTTTTTTGCCCCATAA   254
trnP  --------------------------------------------------------------------------------------------------------------------------------    60
trnF  --------------------------------------------------------------------------------------------------------------------------------     0

R     TATATTCTTTAGTACCACCACTCTGACTATTACAGTCAATCTTGCTTAACCAACCGGTTATTTATTGATTTCACACCTCACGAGAACCGACCAATCCTTGCTTGCGTGCTATTCCTCACTAGTCTCGT   382
trnP  --------------------------------------------------------------------------------------------------------------------------------    60
trnF  --------------------------------------------------------------------------------------------------------------------------------     0

R     GGGTCATCAATCGAGGTTGCAACACAACTTGCTCTTTCCAAGACCTACGGGCGTTTAGTCAGGCACTTCTAACCCTTAATCATAACCACGTCCTCCTTTCCAAGGCCTGTAGTTGCAGCTTATCCTTG   510
trnP  --------------------------------------------------------------------------------------------------------------------------------    60
trnF  --------------------------------------------------------------------------------------------------------------------------------     0

R     CTACCTACATACCATGATTGCACAACCCTTGGCCGCCCCGCATTCAGCTTTTTTTTAGCGTCTCAGCCTCACCAACACCAGTGGTCGCTACCGATCCATTGTAGGTGGGTACCATATATCTCAATCTT   638
trnP  --------------------------------------------------------------------------------------------------------------------------------    60
trnF  --------------------------------------------------------------------------------------------------------------------------------     0

R     TTATTCCCTCCGACTTCATTTTGGGGCATTTCATTTATGCTTGTTAGTCATAAAAATCGCAAAAAAATTGCCAAATTTGTCCGAAATTCAACTAGAACAAAGGACAAACTAAAAAACAAACAAACAACC   766
trnP  --------------------------------------------------------------------------------------------------------------------------------    60
trnF  --------------------------------------------------------------------------------------------------------------------------------     0

R     AAACGTTTGTTTTTAACCTAAAATCATCTAAAACAACGGATTTTTTAAAAAACCAACAAATTCCGTGTTTGTCAAAGATTCAACTAGAACAACGAACAAACAAACAAATGAACAAACAAATGAACACA   894
trnP  --------------------------------------------------------------------------------------------------------------------------------    60
trnF  --------------------------------------------------------------------------------------------------------------------------------     0
```

```
R     AATGATTAAACAAACAAACAAACAAACATTTGTTGTTAGCTCAAATTTTTCTAGAAAAATTAATAAACAAACTAAACAAACAAACGAACAAACATTTGTTGTTAGCTCAAATTTTTCTAGAAAAATTA  1022
trnP  --------------------------------------------------------------------------------------------------------------------------------    60
trnF  --------------------------------------------------------------------------------------------------------------------------------     0

R     ATAAACAAACTAAACAAACAAACGAACAAACATTTGTTGTTAGCTCAAATTTTTCTAGAAAAATTAATAAACAAACTAAACAAACAAACGAACAAACATTTGTTGTAGCTCAAATTTTTCTAGAAAAT  1150
trnP  --------------------------------------------------------------------------------------------------------------------------------    60
trnF  --------------------------------------------------------------------------------------------------------------------------------     0

R     TAATAAACAAACTAAACAAACAAACGAACGAACAAACATTTGTTGTTAGCTCAAATTTTTCTAGAAAAATTAATAAACAAACTAAACAAAGTTTGTTATAGCTAGTTTATCTAGACGAACACACTCCG  1278
trnP  --------------------------------------------------------------------------------------------------------------------------------    60
trnF  --------------------------------------------------------------------------------------------------------------------------------     0

R     CCGCATATACTTTTTAGAATTAATTTTTTTATTTTTTATAAAAATTTTTAATATATATATATATTTTTATAAAAACTTTTTAGATATATATATATATATATATTTTTTTTATAAAAAAATATTTAAT  1406
trnP  --------------------------------------------------------------------------------------------------------------------------------    60
trnF  --------------------------------------------------------------------------------------------------------------------------------     0

R     ATATATATATATATATTTTTTATTTTTTTATAAAAATTTTTAATATATATATATATATATTTTTTTATTTTTTTATAAAAATTTTTAATATATATATATATTTTTATAAATATTTATTTTTATTACAC  1534
trnP  --------------------------------------------------------------------------------------------------------------------------------    60
trnF  --------------------------------------------------------------------------------------------------------------------------------     0

                                                   trnF
R     ATTCAACCGCCAAACCTCTAAAACACAAACGTACTTGTAGCTTAATTT-AAAGCCTAGTGTTGAAAACACTAAAACGAGCCAAACGCTCC  1623
trnP  ------------------------------------------------------------------------------------------    60
trnF  ----------------------------------TTGTAGCTTACCACCAAAGCATAGTGCTGAAGACACTAAGACGAGCCTTACTCCAA    56
                                                   trnF
```
